# Supplementary material for: Separable roles of the DNA damage response kinase Mec1ATR and its activator Rad24RAD17 during meiotic recombination
Source: PLoS Genet. 2024 Dec 9;20(12):e1011485. doi: 10.1371/journal.pgen.1011485 (PMC11658708; doi:10.1371/journal.pgen.1011485)
Supplement: S1 Fig — The method is based on that described previously [39,40]. Notable differences are that mismatches are tolerated during the initial alignment to preserve reads containing variants from both parents; the alignment is carried out against both parental genomes, and genotype calls reconciled; and the variant table includes both SNPs and indels. Additionally, we introduce the technique of sequencing msh2Δ tetrads and using heteroduplex calls to reconstruct an ersatz octad. (PDF) [file pgen.1011485.s001.pdf]

Figure S1

Sequence genomic DNA from each member of a tetrad or octad

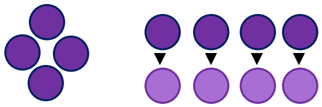

Align DNA to both parental genomes (S288c and SK1).
Alignment is tolerant of mismatches

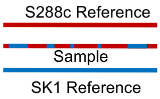

Genotype reads for each sample. Resolve separate parental calls into a binary signal

Pos	Call1	Call2	Final
1	c	c	c
2	c	–	c
3	c	c	c
4	k	–	k
5	h	h	h
6	k	c	–

c = S288c
k = SK1
h = heteroduplex
– = discarded

Combine members of an octad/tetrad

Pos	1a	1b	2a	2b	3a	3b	4a	4b
1	c	c	k	k	k	k	c	c
2	c	c	k	k	k	k	h	h
3	c	c	h	h	k	k	c	c
4	k	k	c	c	k	k	c	c
5	k	k	c	c	k	k	c	c

In *msh2Δ* tetrads, resolve heteroduplex calls

Pos	1a	1b	2a	2b	3a	3b	4a	4b
1	c	c	k	k	k	k	c	c
2	c	c	k	k	k	k	k	c
3	c	c	k	c	k	k	c	c
4	k	k	c	c	k	k	c	c
5	k	k	c	c	k	k	c	c

Segment chromosomes into segregation pattern changes based on binarised calls

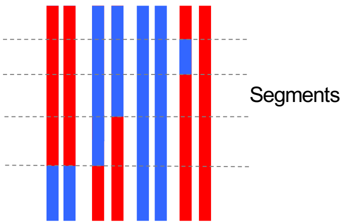

Group segregation pattern changes into events:
CO if reciprocal exchange occurs
NCO if not.

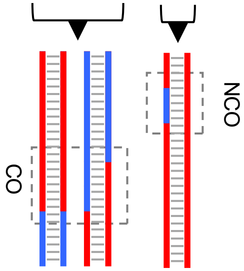

Manually examine events to categorize complex events
