# Supplementary figures and images for: Separable roles of the DNA damage response kinase Mec1ATR and its activator Rad24RAD17 during meiotic recombination
Source: PLoS Genet. 2024 Dec 9;20(12):e1011485. doi: 10.1371/journal.pgen.1011485 (PMC11658708; doi:10.1371/journal.pgen.1011485)

# Figure S2

**A**

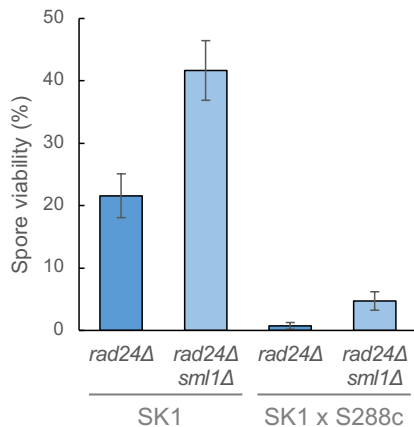

**B**

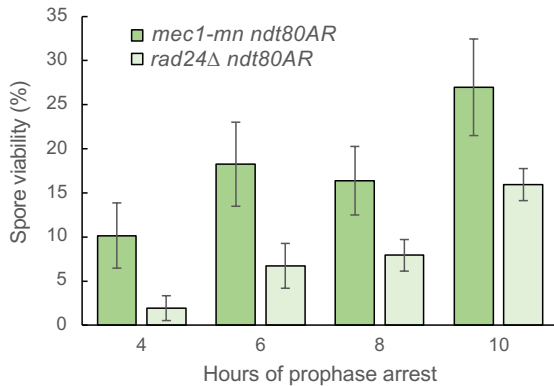

Supplement: S2 Fig — All error bars are standard error of the mean. A) Rescue of rad24Δ spore viability by sml1Δ. The spore viability of both SK1 and SK1xS288c hybrid strains with a deletion of RAD24 is improved by the additional deletion of SML1. B) The effect of increasing prophase length on the spore viability of hybrid mec1-mn and rad24Δ yeast. Hybrid SK1xS288c strains are arrested in prophase and released after 4–10 hours using an inducible NDT80 system ‘ndt80AR’. Both Mec1 and Rad24 mutants display an improvement in spore viability when prophase is extended (compare with non-arrested viabilities shown in Fig 1C), and the level of improvement correlates with the length of the arrest. (PDF) [file pgen.1011485.s002.pdf]

Figure S3

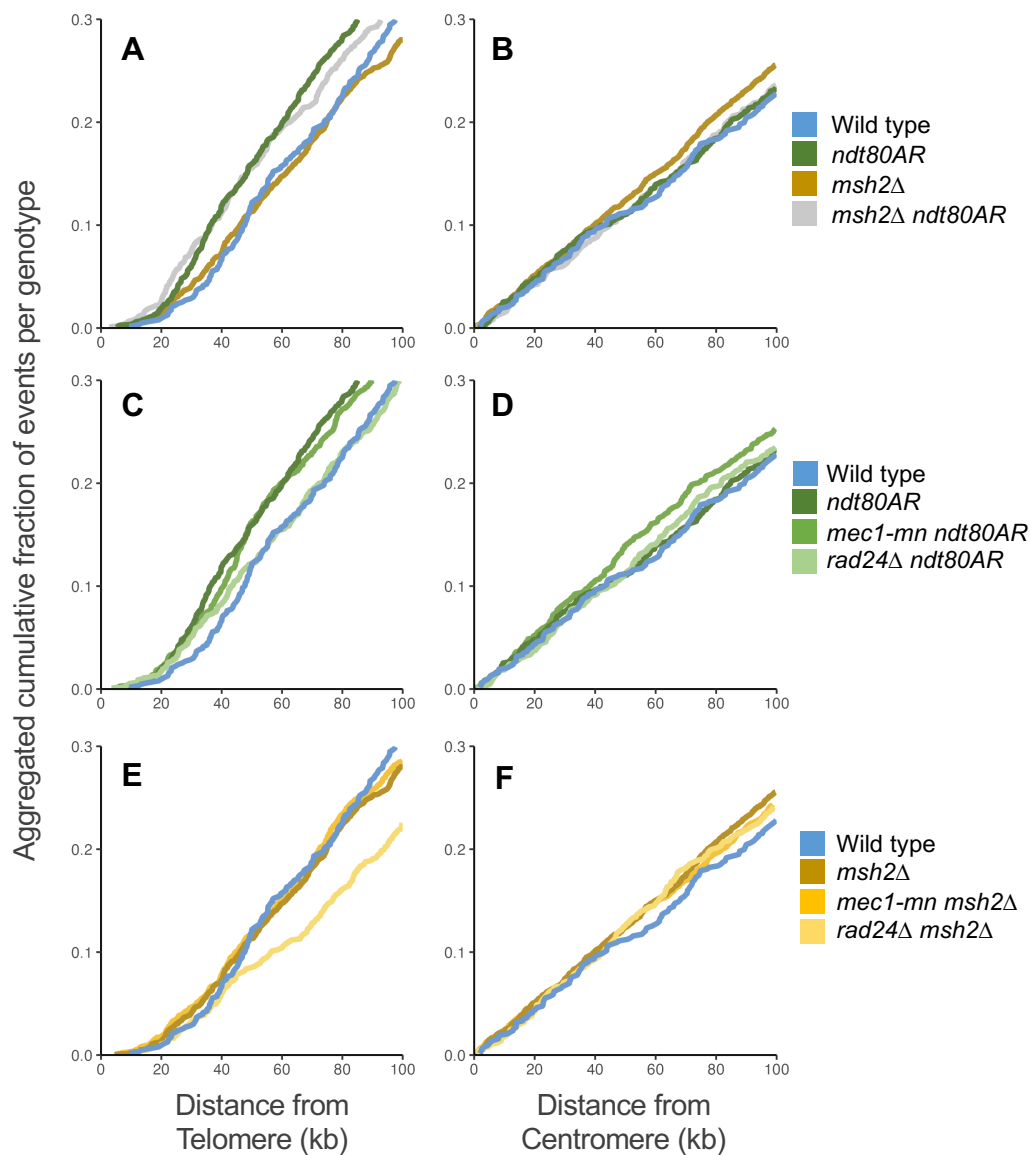

Supplement: S3 Fig — The cumulative fraction of all recombination events plotted against the distance from the nearest telomere (A,C,E) or centromere (B,D,F), for the indicated strains. (PDF) [file pgen.1011485.s003.pdf]

Figure S4

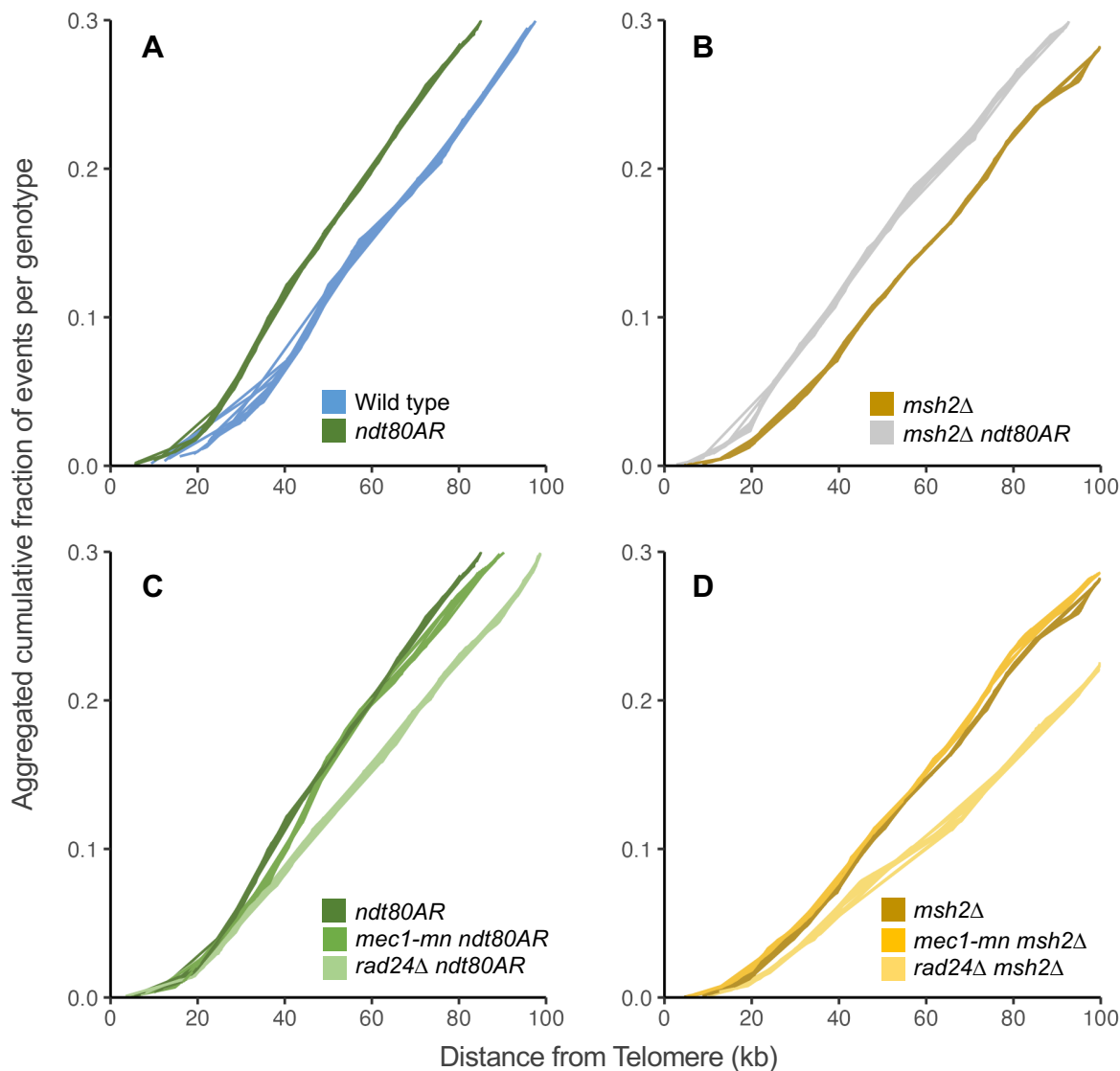

Supplement: S4 Fig — The cumulative fraction of all recombination events plotted against the distance from the nearest telomere, stratified by chromosome, for the indicated strains. Each chromosome is represented by a single line, coloured by strain. (PDF) [file pgen.1011485.s004.pdf]

Figure S5

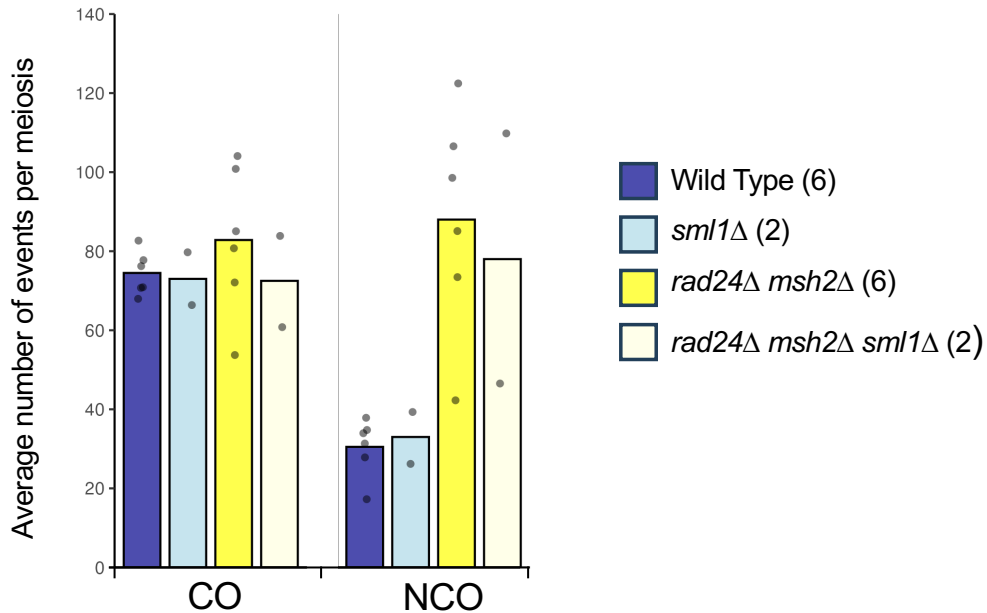

Supplement: S5 Fig — The mean counts of CO and NCO events per tetrad are shown (including both single and multi-DSB events). All error bars are standard error of the mean. Event count differences between sml1Δ and SML1 versions of strains were tested by two-tailed T-test, and found to be insignificant. (PDF) [file pgen.1011485.s005.pdf]

Figure S6

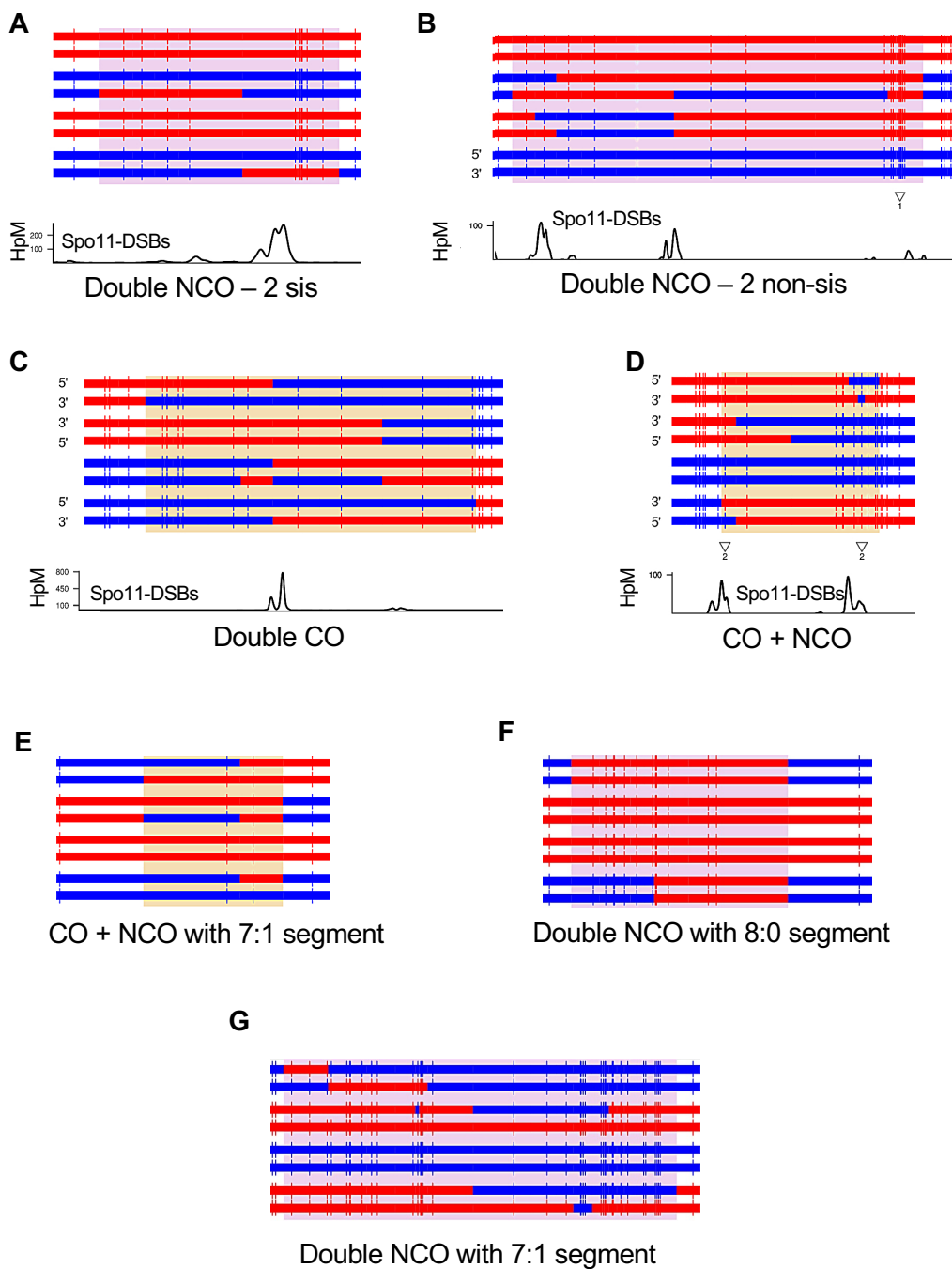

Supplement: S6 Fig — All example images are from msh2Δ octads. (A-G) A representative set of multi-DSB events taken from msh2Δ octads. Horizontal lines represent the eight strands of DNA present during recombination, while vertical lines are SNP/indel locations, with the S288C and SK1 alleles coloured red and blue, respectively. The orange/purple background highlights the CO/NCO event region respectively; events containing both a CO and an NCO are also coloured orange. The bottom part of panels A-D shows the counts of immunoprecipitated Spo11-FLAG oligos for each position, using S288C coordinates [62] smoothed using a 101bp hann window. A) Double noncrossover affecting two sister chromatids. B) Double non-crossover affecting two non-sister chromatids; this is determined by the occurrence of a small region of double-exchange, which could potentially be compatible with a very close double CO. C) Double CO event; this is determined by the double reciprocal exchange. D) CO and NCO event; this is determined by the occurrence of the NCO on a separate chromatid to the CO event. E) CO and NCO event containing a region of 7:1 segregation. F) Double noncrossover affecting two sister chromatids, and containing a region of 8:0 segregation. G) Double NCO containing a region of 7:1 segregation. (PDF) [file pgen.1011485.s006.pdf]

Figure S7

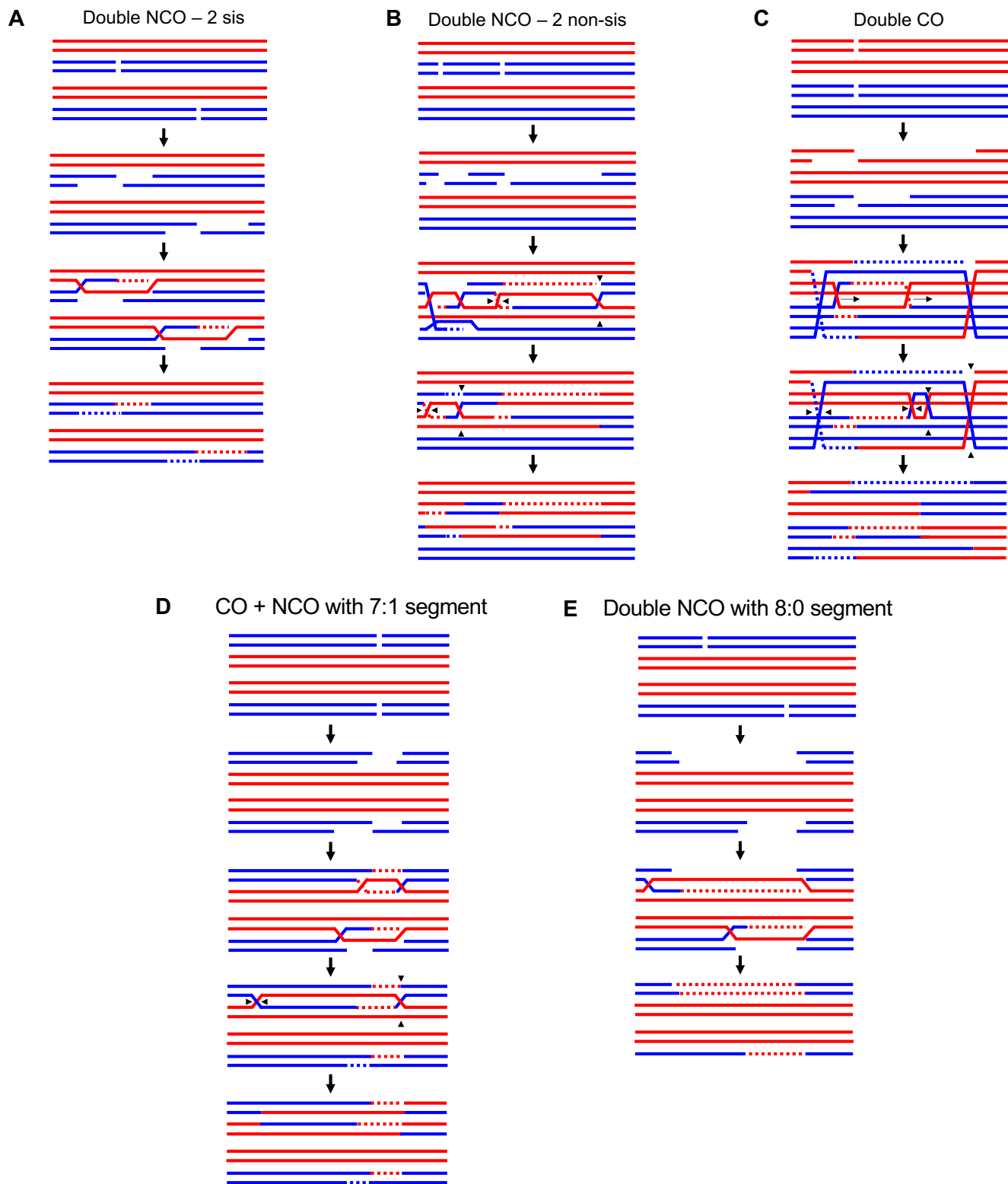

Supplement: S7 Fig — A) Double NCO on two sister chromatids formed by two independent SDSA reactions (S6A Fig). B) Double NCO on two non-sister chromatids. A second event needs to be invoked to account for the 6:2 conversion on the right: either another DSB + SDSA or a nick repair during resolution (S6B Fig). C) Double CO. Determined by the double reciprocal exchange (S6C Fig). For CO + NCO (S6D Fig), typical DSBR with CO resolution as in S9D Fig plus an additional SDSA with nick translation. D) CO + NCO with 7:1 segment (S6E Fig). E) Double NCO with 8:0 segment. Two independent SDSA reactions initiated on two sister chromatids but with processing of both ends yielding a gap. The short hDNA tracts are not detected due to low SNP density. (S6F Fig). Double NCO with a 7:1 segment (S6G Fig) caused by multiple DSBs: The first causing a two-sided NCO as in S10C Fig on chr 1, the second causing SDSA with one nick translation on chromosome 4, the third causing SDSA with template switching on chromosome 2. (PDF) [file pgen.1011485.s007.pdf]

# Figure S8

**A**

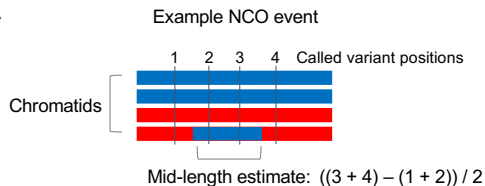

## CO Events

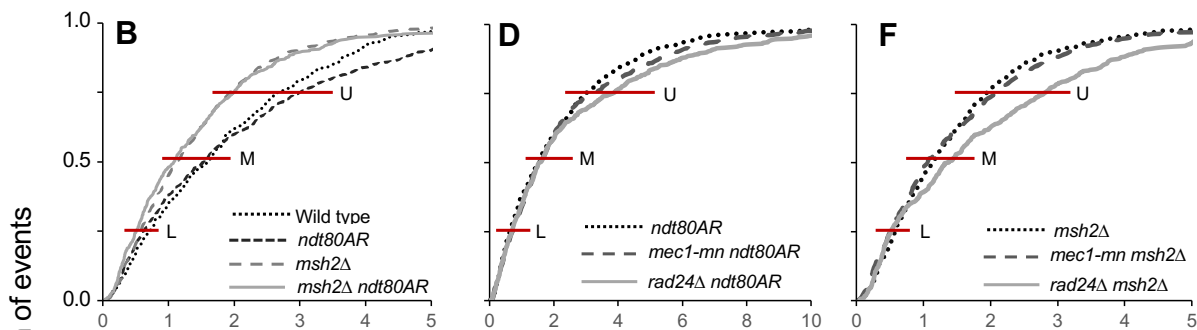

## NCO Events

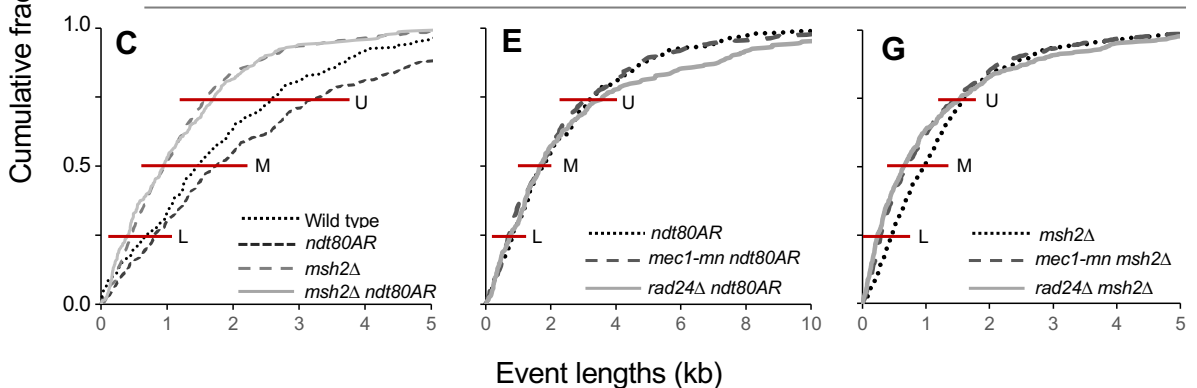

Supplement: S8 Fig — A) Event mid-length calculation is defined as the distance between the midpoints of the intermarker intervals surrounding the recombination event. Multi-DSB events are omitted from these analyses. B-G) Distribution of mid-lengths of the strand transfers associated with CO (B,D,F) and NCO (C,E,G) events in the indicated strains. Red horizontal lines indicate the values of the Upper Quartile (U), Median (M) and Lower Quartile (L) summarised in Fig 5. (PDF) [file pgen.1011485.s008.pdf]

Figure S9

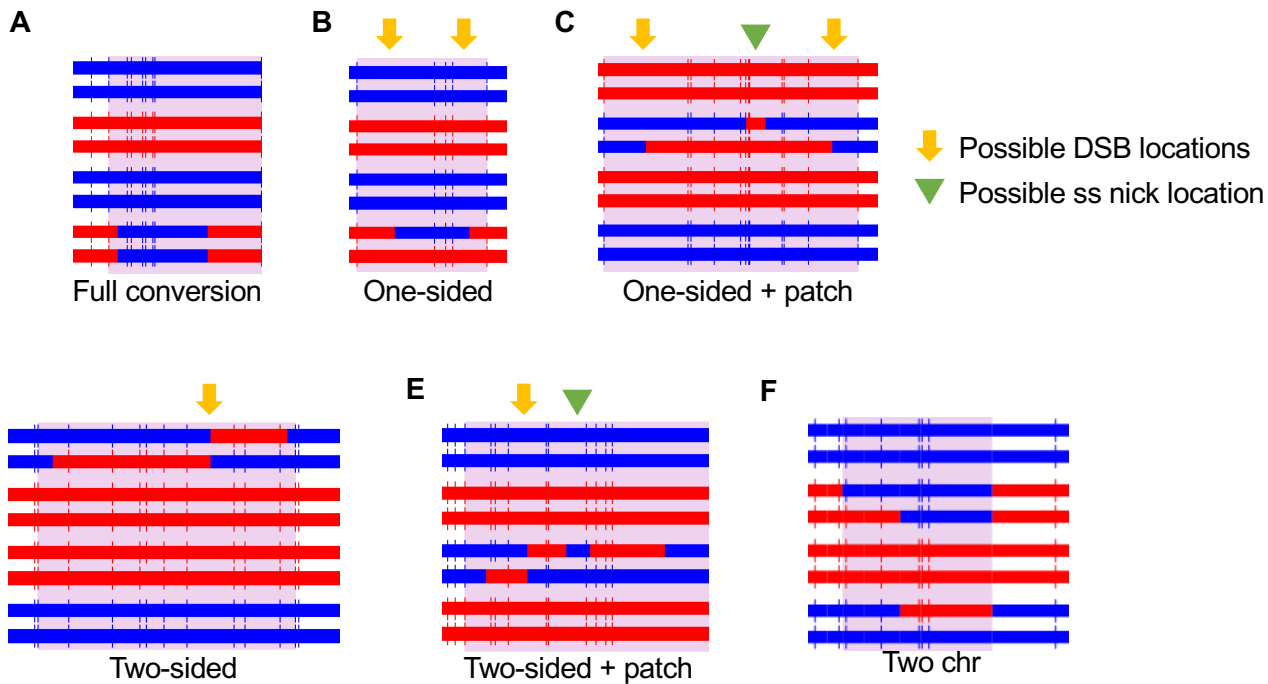

Supplement: S9 Fig — All example images are from msh2Δ octads. (A-F) A representative set of NCO events taken from msh2Δ octads. Horizontal lines represent the eight strands of DNA present during recombination, while vertical lines are SNP/indel locations, with the S288C and SK1 alleles coloured red and blue, respectively. The purple background highlights the NCO event region. Categories are named as in [40]. The term ‘Two-Sided’ refers occurrence of strand transfer patterns on both sides of the putative DSB location. A lack of hDNA on both sides of the event (‘One-Sided’) may be caused by an absence of markers, or may be due to a peculiarity of the repair process e.g. template switching. A) Noncrossover with a single full-conversion tract. B) One-sided noncrossover with a half-conversion tract. C) One-sided noncrossover with a half-conversion tract and an internal patch of full conversion. D) Two-sided noncrossover with two half-conversion tracts (trans hDNA) affecting the same chromatid. E) Two-sided noncrossover with two half-conversion tracts affecting the same chromatid and separated by a restoration tract. F) Noncrossover that affects two non-sister chromatids. (PDF) [file pgen.1011485.s009.pdf]

Figure S10

**A**

Full conversion

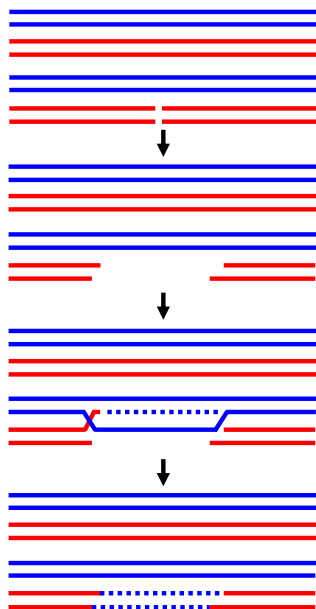

**B**

One-sided

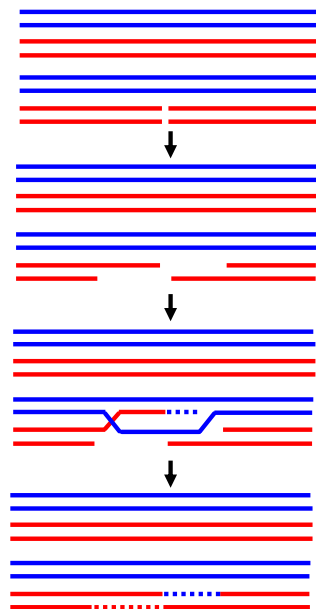

**C**

Two-sided

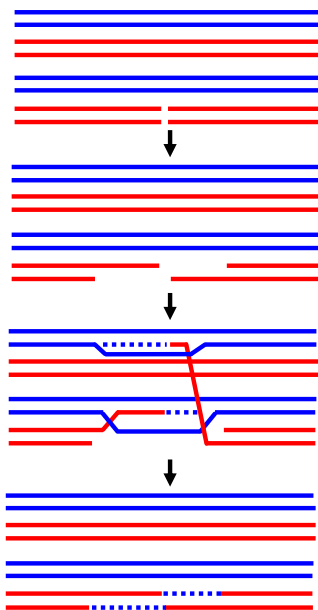

**D**

Two chr

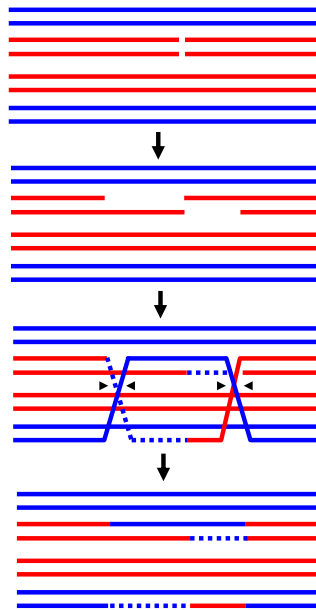

Supplement: S10 Fig — A) Full conversion. The short hDNA tracts near the edges of the gap escape detection due to SNP density (S9A Fig). B) Synthesis dependent strand annealing (SDSA). Results in a one-sided NCO (S9B Fig) or one-sided + patch if there is a nick translation within the converted strand of an hDNA tract (S9C Fig). C) Double SDSA. Results in a two-sided NCO (S9D Fig) or two-sided + patch if there is a nick translation within the converted strand of an hDNA tract (S9E Fig). Can also arise from dHJ dissolution and nicked dHJ disassembly. D) DSB repair with NCO resolution resulting in an NCO that affects two non-sister chromatids (S9F Fig). (PDF) [file pgen.1011485.s010.pdf]

Figure S11

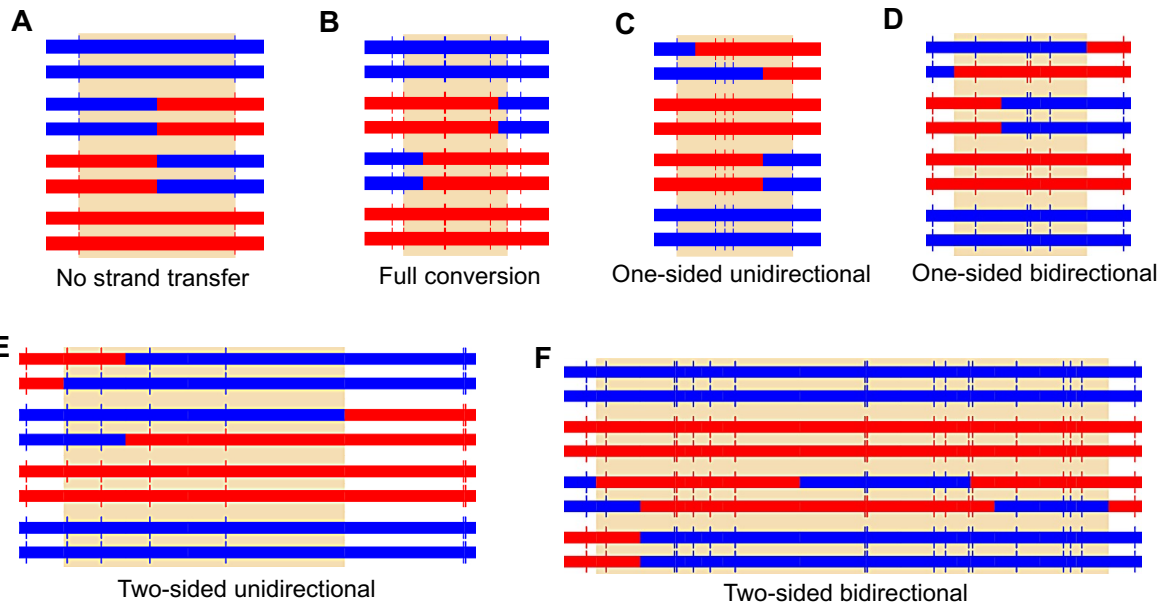

Supplement: S11 Fig — All example images are from msh2Δ octads. (A-F) A representative set of CO events taken from msh2Δ octads. Horizontal lines represent the eight strands of DNA present during recombination, while vertical lines are SNP/indel locations, with the S288C and SK1 alleles coloured red and blue, respectively. The orange background highlights the CO event region. Categories are named as in [40]. The term ‘Two-’ or ‘One-Sided’ refers to the occurrence of strand transfer patterns on one or both sides of the putative DSB location. The term ‘Directionality’ refers to whether markers from only one parent are converted, or both. Bidirectionality may be caused by multiple DSBs/nicking, or junction migration. ‘Sym 4:4’ refers to symmetrical hDNA, which may originate from HJ branch migration. A) Crossover with no detectable strand transfer. B) Crossover with a single full-conversion tract. C) One-sided, unidirectional crossover with a single half-conversion tract. D) One-sided, bidirectional crossover. E) Two-sided, unidirectional crossover with trans hDNA tracts on the two recombining chromatids. F) Two-sided, bidirectional crossover with trans hDNA tracts on one chromatid only. (PDF) [file pgen.1011485.s011.pdf]

Figure S12

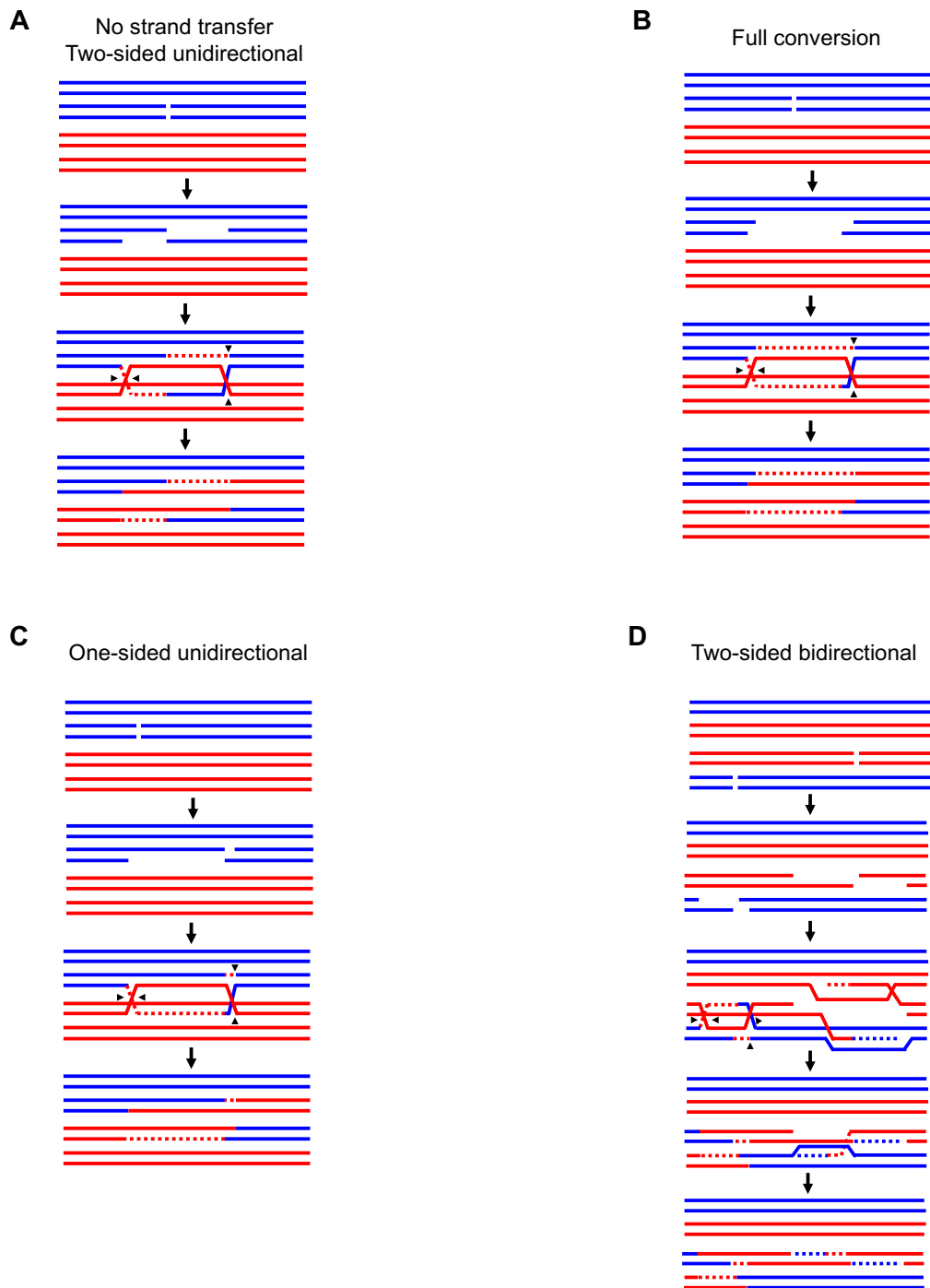

Supplement: S12 Fig — A) DSBR CO resolution. If SNP density is not high enough, hDNA tracts are missed (no strand transfer, S11A Fig). If SNP density is high enough, hDNA tracts are detected (two-sided unidirectional, S11E Fig). B) DSBR CO resolution resulting in full conversion (S11B Fig). Both ends are processed. Short hDNA tracts not picked by the SNP density. C) DSBR CO resolution. Asymmetry in the positioning of the two HJ with respect to the initiating DSB event yeilding asymmetric hDNA tract (one long one short) (S11C Fig). The short hDNA tract is not detected due to low SNP density. There are many complex combinations of events that can give rise to one-sided bidirectional transfer (S11D Fig), so this had been omitted. D) Two-sided bidirectional event caused by two double-strand breaks, resulting in two-sided bidirectional transfer (S11F Fig). (PDF) [file pgen.1011485.s012.pdf]

Figure S13

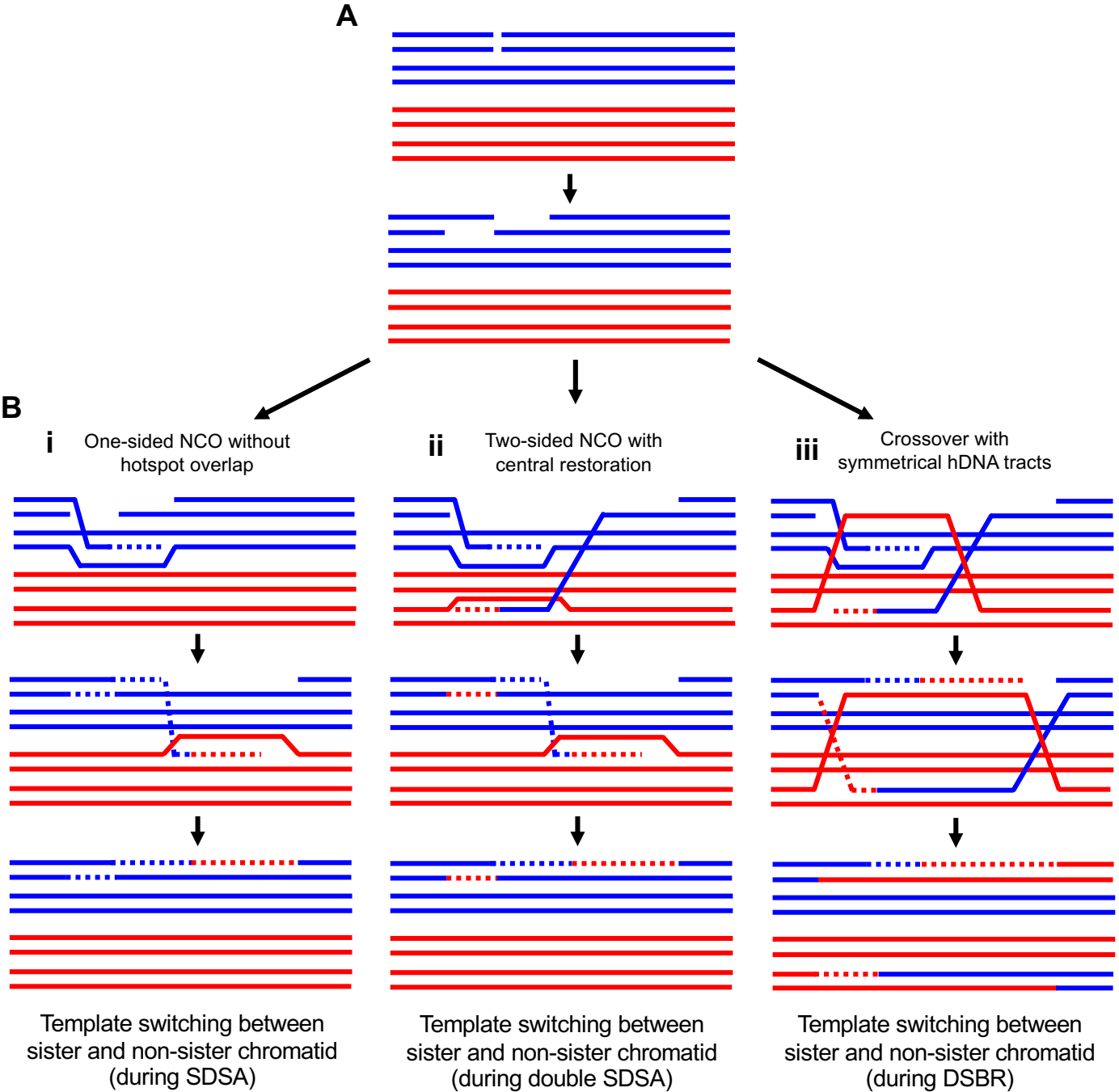

Supplement: S13 Fig — A) DSB and gap widening, common steps giving rise to single-DSB events. B) differential formation of single-DSB events. i) One-sided NCO without hotspot overlap (Fig 6D). ii) Two-sided NCO with central restoration (Fig 6E). iii) Crossover with symmetrical hDNA tracts (Fig 6F). (PDF) [file pgen.1011485.s013.pdf]
