# Supplementary material for: Separable roles of the DNA damage response kinase Mec1ATR and its activator Rad24RAD17 during meiotic recombination
Source: PLoS Genet. 2024 Dec 9;20(12):e1011485. doi: 10.1371/journal.pgen.1011485 (PMC11658708; doi:10.1371/journal.pgen.1011485)
Supplement: S14 Fig — Whole chromosome recombination patterns were mapped in hybrid strains by detection of SK1 (blue) or S288c (red) markers. The region of interest is plotted as eight horizontal lines corresponding to the eight strands of DNA present in the original hybrid diploid, with vertical tick marks indicating called variant positions. A) partial chromosome conversions on Chr 4, 7 and 10 in a PCLB2-MEC1 +10 h tetrad (TCMN6); B) A duplication of S288c Chr 8 in a PCLB2-MEC1 msh2Δ tetrad (TCMM4). Raw reads indicate the frequency of reads containing SK1 or S288c type polymorphisms detected at each position; these are translated into binary calls, which can only be SK1 or S288c. (PDF) [file pgen.1011485.s014.pdf]

Figure S14

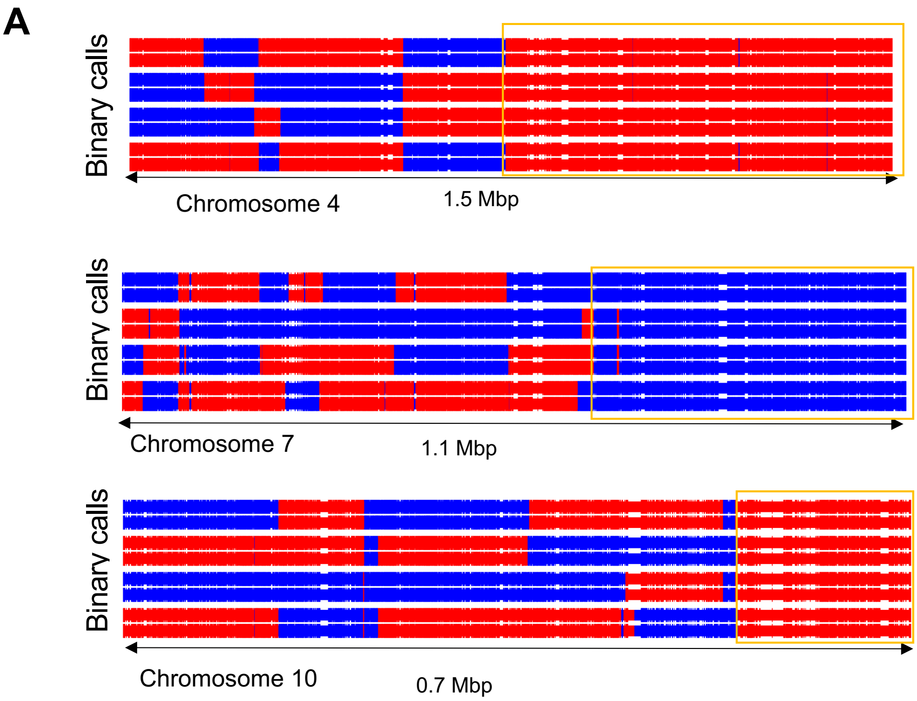

Two spores contain chromosomes where a substantial portion has been converted to the other parental genotype, suggesting a premeiotic recombination event.

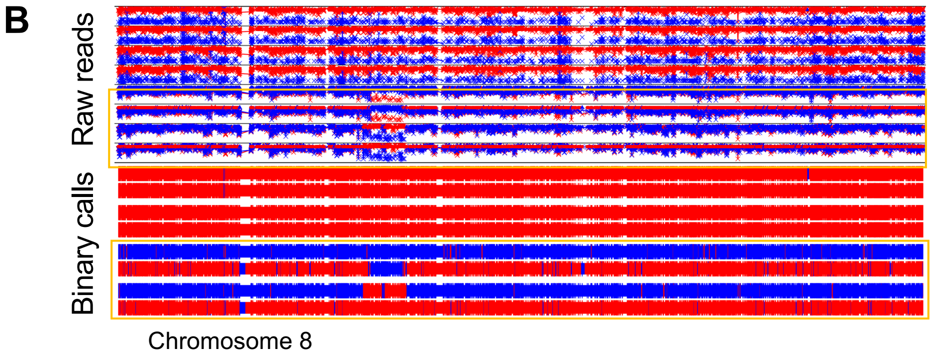

Presence of heteroduplex reads along whole length of chromosome, indicating that two spores have a copy of each parental chromosome, suggesting premeiotic duplication.
