# Supplementary material for: Separable roles of the DNA damage response kinase Mec1ATR and its activator Rad24RAD17 during meiotic recombination
Source: PLoS Genet. 2024 Dec 9;20(12):e1011485. doi: 10.1371/journal.pgen.1011485 (PMC11658708; doi:10.1371/journal.pgen.1011485)
Supplement: S1 Table — Relevant homozygous diploid genotype (column 1), haploid strain numbers used to create each diploid strain (column 2), or SK1 diploid strain where applicable, genetic background (column 3), and number of tetrads dissected (column 4) are indicated. Mean spore viability (column 5) was scored as the percentage of dissected spores that show visible growth after 48 hours incubation at 30°C. To estimate how well the measurement represents the viability of the population, 95% confidence intervals were calculated (column 6). Viability pattern (columns 7–11) refers to the percentage of dissected tetrads that displayed 4, 3, 2, 1 or 0 viable spores. The colour scale used in column 5 indicates the mean spore viability reported for each sample: (Green > Yellow > Red; indicating High > Medium > Low). For all recombination mapping experiments using ndt80AR arrest-release, cultures were sporulated for 8 hours prior to oestradiol induction. (PDF) [file pgen.1011485.s015.pdf]

Table S1

S. cerevisiae spore viability measurements

Genotype	Cross	Background	Tetrads dissected	Mean spore viability	95% confidence interval	Viability pattern 4	3	2	1	0
WT	MJ512xMJ513	SK1	64	96.9	2.13	92.19	3.13	4.69	0.00	0.00
WT	MJ600xMJ631	S288c	72	96.9	2.01	88.89	9.72	1.39	0.00	0.00
WT	MJ600xMJ513	SK1xS288c	197	82.0	2.68	53.81	27.41	14.21	2.03	2.54
msh2Δ	MC25xMC26	SK1	110	80.9	3.67	60.00	12.73	18.18	9.09	0.00
msh2Δ	MC49xMC51	S288c	105	77.9	3.97	47.62	26.67	18.10	4.76	2.86
msh2Δ	MC49xMC26	SK1xS288c	149	73.0	3.56	40.27	25.50	21.48	11.41	1.34
ndt80AR 8h	MJ846	SK1	40	94.4	3.57	82.50	12.50	5.00	0.00	0.00
ndt80AR 10h	MJ43xMC42	SK1xS288c	86	70.4	4.83	36.05	25.58	25.58	9.30	3.49
mec1-mn	VG120xVG121	SK1	130	58.5	4.24	21.54	32.31	15.38	20.00	10.77
mec1-mn	MC152xMC153	S288c	110	77.3	3.92	50.00	24.55	17.27	0.91	7.27
mec1-mn	VG120xMC153	SK1xS288c	153	6.4	1.94	1.31	0.65	4.58	9.15	84.31
mec1-nm msh2Δ	MC163xMC164	SK1	88	49.2	5.22	19.32	23.86	14.77	18.18	23.86
mec1-nm msh2Δ	MC171xMC172	S288c	111	63.1	4.49	30.63	25.23	19.82	14.41	9.91
mec1-nm msh2Δ	MC163xMC172	SK1xS288c	176	28.8	3.35	3.41	10.80	17.61	34.09	34.09
mec1-mn ndt80AR 3h	MC2xMC3	SK1	88	49.2	5.22	12.50	28.41	18.18	25.00	15.91
mec1-mn ndt80AR 6h	MC2xMC3	SK1	86	54.1	5.27	19.77	23.26	23.26	20.93	12.79
mec1-mn ndt80AR 8h	MC2xMC3	SK1	84	57.7	5.28	17.86	30.95	25.00	16.67	9.52
mec1-mn ndt80AR 10h	MC2xMC3	SK1	86	48.6	5.28	12.79	19.77	30.23	23.26	13.95
mec1-mn ndt80AR 4h	MC2xMC198	SK1xS288c	64	10.2	3.70	1.56	1.56	3.13	23.44	70.31
mec1-mn ndt80AR 6h	MC2xMC198	SK1xS288c	63	18.3	4.77	0.00	6.35	14.29	25.40	53.97
mec1-mn ndt80AR 8h	MC2xMC198	SK1xS288c	87	16.4	3.89	2.30	4.60	9.20	24.14	59.77
mec1-mn ndt80AR 10h	MC2xMC198	SK1xS288c	63	27.0	5.48	4.76	6.35	19.05	31.75	38.10
rad24Δ	MC17xMC18	SK1	132	21.6	3.51	7.58	5.30	17.42	5.30	64.39
rad24Δ	MC19xMC20	S288c	106	46.7	4.75	12.26	17.92	29.25	25.47	15.09
rad24Δ	MC17xMC20	SK1xS288c	240	0.7	0.54	0.00	0.00	0.00	2.92	97.08
rad24Δ msh2Δ	MC105xMC107	SK1	107	21.6	3.88	7.58	5.30	17.42	5.30	64.39
rad24Δ msh2Δ	MC202xMC203	S288c	102	28.2	4.37	2.94	11.76	18.63	28.43	38.24
rad24Δ msh2Δ	MC105xMC203	SK1xS288c	462	5.9	1.07	1.30	2.38	8.87	21.00	66.45
rad24Δ ndt80AR 4h	MJ835xMC89	SK1xS288c	91	1.9	1.41	0.00	0.00	2.20	3.30	94.51
rad24Δ ndt80AR 6h	MJ835xMC89	SK1xS288c	93	6.7	2.54	0.00	4.30	1.08	11.83	82.80
rad24Δ ndt80AR 8h	MJ835xMC89	SK1xS288c	218	7.9	1.79	0.46	0.92	4.59	17.89	76.15
rad24Δ ndt80AR 10h	MJ835xMC89	SK1xS288c	269	15.9	1.82	1.86	2.60	13.38	17.84	64.31
rad24Δ ndt80AR 4h	MJ848	SK1	44	31.8	6.88	4.55	18.18	20.45	13.64	43.18
rad24Δ ndt80AR 6h	MJ848	SK1	66	42.8	5.97	9.09	15.15	36.36	16.67	22.73
rad24Δ ndt80AR 8h	MJ848	SK1	84	59.2	5.42	15.48	26.19	28.57	25.00	4.76
rad24Δ ndt80AR 10h	MJ848	SK1	59	69.1	5.90	35.59	22.03	28.81	10.17	3.39
rad24Δ sml1Δ	MC101xMC103	SK1	102	41.7	4.78	18.63	13.73	21.57	7.84	38.24
rad24Δ sml1Δ	MC101xMC61	SK1xBY474	196	4.7	1.48	0.51	0.51	4.08	7.14	87.76
mus81Δ	MC214xMC215	SK1	110	55.7	4.64	17.27	27.27	25.45	20.91	9.09
mlh3Δ	MC254xMC255	SK1	110	89.1	2.91	81.82	3.64	9.09	0.00	5.45
mus81Δ mlh3Δ	MC271xMC272	SK1	107	59.6	4.65	26.17	28.04	19.63	10.28	15.89
mec1-mn mus81Δ	MC243xMC242	SK1	108	20.4	3.80	1.85	3.70	14.81	33.33	46.30
mec1-mn mlh3Δ	MC303xMC304	SK1	117	49.6	4.50	22.22	14.53	23.08	19.66	20.51
mec1-mn mus81Δ mlh3Δ	MC333xMC334	SK1	94	21.3	4.10	1.06	8.51	15.96	23.40	51.06
rad24Δ mus81Δ	MC222xMC223	SK1	110	19.3	3.69	0.00	7.27	20.91	13.64	58.18
rad24Δ mus81Δ msh2Δ	MC292xMC297	SK1XS288c	31	0.0	0.00	0.00	0.00	0.00	0.00	100.00
rad24Δ mlh3Δ	MC247xMC248	SK1	109	20.6	3.80	6.42	3.67	13.76	18.35	57.80
rad24Δ mus81Δ mlh3Δ	MC257xMC258	SK1	98	7.9	2.67	1.02	0.00	9.18	9.18	80.61
rad24Δ mus81Δ ndt80AR 4h	MC288xMC289	SK1	89	5.9	2.45	1.12	0.00	4.49	10.11	84.27
rad24Δ mus81Δ ndt80AR 8h	MC288xMC289	SK1	108	9.7	2.79	0.00	1.85	2.78	27.78	67.59
mus81Δ ndt80AR 4h	MC286xMC287	SK1	128	34.0	4.10	6.25	10.94	23.44	31.25	28.13
mus81Δ ndt80AR 8h	MC286xMC287	SK1	110	33.4	4.41	3.64	17.27	21.82	23.64	33.64
zip3Δ	MC322xMC313	SK1xS288c	106	46.2	4.75	13.21	18.87	31.13	13.21	23.58
zip3Δ msh2Δ	MC326xMC317	SK1xS288c	177	35.9	3.53	7.34	12.99	28.25	18.64	32.77
