# Supplementary material for: Separable roles of the DNA damage response kinase Mec1ATR and its activator Rad24RAD17 during meiotic recombination
Source: PLoS Genet. 2024 Dec 9;20(12):e1011485. doi: 10.1371/journal.pgen.1011485 (PMC11658708; doi:10.1371/journal.pgen.1011485)
Supplement: S2 Table — For each individual meiosis, values are given for the number of single CO and NCO events, the identity of chromosomes without a CO or NCO event, the number of multi-DSB events (either dCO, dNCO or CO + NCO), and the number of individual events containing an 8:0 or 7:1 segment (not an exclusive category; all such events are also considered to be multi-DSB events of some kind). An increase in the formation of multi-DSB events suggests a loss of CO interference and/or cis/trans DSB interference. The occurrence of chromosomes without COs suggests a loss of CO assurance; COs are not spread out evenly between chromosomes. Many of these chromosomes are also missing NCOs, meaning they could not have compensated for the lack of CO events. The occurrence of 7:1 or 8:0 segregation patterns within an event suggests a loss of trans DSB interference. Example images for multi-DSB and 8:1/7:1 segment events are shown in S6 Fig. 7:1 segments cannot be called in MSH2 strains (NA) due to lack of heteroduplex strand information. * mec1-mn msh2Δ 1 and mec1-mn ndt80AR 6 were not used for any analysis except event lengths, due to large mitotic duplication events (see Methods and S14 Fig). rad24Δ msh2Δ #6 and mec-mn msh2Δ #3 underwent haplotype resequencing (Methods) to reproduce a full octad (see Fig 5A). (PDF) [file pgen.1011485.s016.pdf]

Table S2

Meiosis	Single COs	Single NCOs	Double COs	Double NCOs	CO and NCO	Chromosomes without a CO	Chromosomes without an NCO	8:0 segregation	7:1 segregation
Wild type 1	65	32	0	2	3	Chr 1,6	Chr 1,3,6	1	NA
Wild type 2	80	37	0	1	3	Chr 1	Chr 6	0	NA
Wild type 3	72	32	0	2	4	-	Chr 2,3	1	NA
Wild type 4	68	30	0	2	3	-	Chr 1,5,6,12	1	NA
Wild type 5	70	28	0	0	1	Chr 1	Chr 3,5,8	0	NA
Wild type 6	75	16	1	1	2	-	Chr 6,7,9,10,11	4	NA
msh2Δ 1	109	91	0	3	7	-	-	0	0
msh2Δ 2	94	117	0	1	6	-	-	0	0
msh2Δ 3	90	83	0	6	5	-	-	0	0
msh2Δ 4	93	82	1	3	10	-	-	0	1
msh2Δ 5	98	77	0	1	0	-	Chr 6	0	0
msh2Δ 6	113	60	1	2	1	-	Chr 1,7	1	0
msh2Δ 7	88	121	0	4	10	-	-	0	1
msh2Δ 8	98	96	0	4	9	-	Chr 6	0	2
msh2Δ 9	103	79	0	1	7	-	-	0	0
ndt80AR 1	66	30	0	0	4	-	-	1	NA
ndt80AR 2	82	35	1	1	6	-	Chr 3	1	NA
ndt80AR 3	107	55	2	3	6	-	Chr 3	5	NA
ndt80AR 4	103	59	1	2	3	-	-	3	NA
ndt80AR 5	106	41	0	2	2	-	-	1	NA
ndt80AR 6	89	49	1	6	2	-	Chr 5	2	NA
ndt80AR 7	74	39	1	1	0	-	Chr 2,13	0	NA
ndt80AR 8	98	52	1	4	8	-	-	3	NA
msh2Δ ndt80AR 1	82	56	2	5	6	-	Chr 3	1	1
msh2Δ ndt80AR 2	106	94	1	4	14	-	-	1	2
msh2Δ ndt80AR 3	91	72	0	2	6	-	-	0	0
msh2Δ ndt80AR 4	80	94	0	3	1	-	-	0	0
msh2Δ ndt80AR 5	84	79	0	1	3	-	-	0	0
msh2Δ ndt80AR 6	95	99	2	3	17	-	-	1	4
mec1-mn 1	58	24	2	4	6	Chr 2	Chr 2	8	NA
mec1-mn 2	82	59	3	11	14	Chr 1	Chr 1	20	NA
*mec1-mn msh2Δ 1 **	59	134	1	4	7	Chr 8	Chr 8	1	1
mec1-mn msh2Δ 2	122	172	0	16	23	-	-	4	6
mec1-mn msh2Δ 3 #	109	119	0	8	27	-	Chr 1	1	3
mec1-mn msh2Δ 4	116	152	3	16	23	-	-	3	5
mec1-mn msh2Δ 5	131	126	2	18	27	-	-	5	4
mec1-mn msh2Δ 6	118	214	3	25	30	-	-	5	10
mec1-mn ndt80AR 1	155	88	2	9	37	-	-	21	NA
mec1-mn ndt80AR 2	89	37	1	4	6	-	-	9	NA
mec1-mn ndt80AR 3	109	54	2	6	22	-	-	15	NA
mec1-mn ndt80AR 4	87	60	3	3	13	-	Chr 14	7	NA
mec1-mn ndt80AR 5	112	71	2	17	35	-	-	32	NA
*mec1-mn ndt80AR 6**	59	34	6	3	4	Chr 1	Chr 3,4	11	NA
rad24Δ msh2Δ 1	50	42	0	0	4	Chr 5,15	Chr 5	0	0
rad24Δ msh2Δ 2	68	76	1	9	16	Chr 1	Chr 1,6	2	4
rad24Δ msh2Δ 3	90	110	0	12	14	-	-	2	5
rad24Δ msh2Δ 4	76	96	1	10	4	Chr 6,8	-	0	0
rad24Δ msh2Δ 5	88	69	1	5	12	Chr 8	-	0	1
rad24Δ msh2Δ 6 #	66	91	0	8	6	-	Chr 3	1	1
rad24Δ ndt80AR 1	112	60	3	12	28	-	-	17	NA
rad24Δ ndt80AR 2	117	38	3	7	16	-	-	7	NA
rad24Δ ndt80AR 3	83	42	6	8	12	Chr 6	Chr 1,3,6	7	NA
rad24Δ ndt80AR 4	128	81	2	13	23	-	-	13	NA
rad24Δ ndt80AR 5	79	32	0	6	8	Chr 6	Chr 6	6	NA
rad24Δ ndt80AR 6	85	56	4	22	20	-	-	6	NA
rad24Δ sml1Δ	25	6	2	2	5	Chr 1,5,8,11,12,14	Chr 1,5,7,8,9,11,12,14,15	8	NA
rad24Δ msh2Δ sml1Δ 1	70	97	0	13	14	Chr 3	-	2	1
rad24Δ msh2Δ sml1Δ 2	58	40	0	6	3	Chr 3,8	Chr 1,3	1	0
sml1Δ 1	66	40	0	1	1	-	Chr 1,12	2	NA
sml1Δ 2	80	26	0	1	2	-	Chr 1,9,14	2	NA
zip3Δ 1	61	76	1	7	4	Chr 3	-	2	NA
zip3Δ 2	43	84	0	6	3	Chr 12	-	4	NA
zip3Δ 3	47	86	0	5	8	Chr 1	-	2	NA
zip3Δ 4	35	55	0	1	9	Chr 6	Chr 5	2	NA
zip3Δ msh2Δ 1	24	296	0	17	10	Chr 1	-	0	3
zip3Δ msh2Δ 2	24	294	0	10	1	Chr 9,16	-	1	4
zip3Δ msh2Δ 3	27	289	0	20	7	Chr 15	-	0	3
zip3Δ msh2Δ 4	28	376	1	32	10	-	-	2	3
zip3Δ msh2Δ 5	21	368	0	33	14	Chr 3,4	-	2	10
zip3Δ msh2Δ 6	27	304	0	15	6	Chr 11	-	0	5
