# Supplementary material for: Separable roles of the DNA damage response kinase Mec1ATR and its activator Rad24RAD17 during meiotic recombination
Source: PLoS Genet. 2024 Dec 9;20(12):e1011485. doi: 10.1371/journal.pgen.1011485 (PMC11658708; doi:10.1371/journal.pgen.1011485)
Supplement: S3 Table — All strains displayed are haploid, and were mated immediately prior to sporulation and tetrad dissection. (PDF) [file pgen.1011485.s017.pdf]

Table S3

Strain	Background	Mat	Genotype	Sequenced sample name
MJ513	SK1	a	*ho::LYS2 lys2Δ leu2 arg4Δ*	WT (TW)
MJ600	S288c	α	*ade8Δ*	WT (TW)
MJ43	SK1	α	*ho::LYS2 lys2Δ arg4Δ leu2Δ::hisG trp1Δ::hisG his4XΔ::LEU2 nuc1Δ::LEU2 PGAL1-NDT80::TRP1 ura3::pGPD1-GAL4(848)-ER::URA3*	*ndt80AR* (TN)
MC42	S288c	a	*ade8Δ ndt80Δ::KanMX*	*ndt80AR* (TN)
MC26	SK1	α	*ho::LYS2 lys2Δ ura3Δ arg4 leu2 msh2Δ::KanMX*	*msh2Δ* (OM)
MC49	S288c	a	*ade8Δ msh2Δ::KanMX*	*msh2Δ* (OM)
MC298	SK1	a	*ho::LYS2 lys2Δ ura3Δ arg4 leu2 trp1Δ::hisG ura3Δ::PGPD1-GAL4(848)-ER::URA3 PGAL1-NDT80::TRP1 msh2Δ::KanMX*	*msh2Δ ndt80AR* (OMN)
MC300	S288c	α	*ade8Δ ndt80Δ::KanMX msh2Δ::KanMX*	*msh2Δ ndt80AR* (OMN)
MC105	SK1	a	*ho::LYS2 lys2Δ ura3Δ arg4 leu2 rad24Δ::HphMX4 msh2Δ::KanMX*	*rad24Δ msh2Δ* (TRM)
MC203	S288c	α	*ade8Δ rad24Δ::HphMX4 msh2Δ::KanMX*	*rad24Δ msh2Δ* (TRM)
MC101	SK1	a	*ho::LYS2 lys2Δ ura3Δ arg4 leu2 rad24Δ::HphMX4 sml1Δ::URA3*	*rad24Δ sml1Δ* (TRS)
MC61	BY4742	α	*lys2Δ his4 ura3Δ leu2Δ met15Δ sml1Δ::URA3 rad24Δ::HphMX4*	*rad24Δ sml1Δ* (TRS)
MC163	SK1	a	*ho::LYS2 lys2Δ ura3Δ arg4Δ leu2Δ::hisG nuc1Δ::LEU2 PCLB2-MEC1::KanMX msh2Δ::KanMX*	*mec1-mn msh2Δ* (TCMM)
MC172	S288c	α	*ade8Δ PCLB2-MEC1::KanMX4 msh2::HphMX4*	*mec1-mn msh2Δ* (TCMM)
MC2	SK1	a	*ho::LYS2 lys2Δ arg4Δ leu2Δ::hisG his4XΔ::LEU2 nuc1Δ::LEU2 trp1Δ::hisG ura3Δ::PGPD1-GAL4(848)-ER::URA3 PCLB2-MEC1::KanMX PGAL1-NDT80::TRP1*	*mec1-mn ndt80AR* (TCMN)
MC198	S288c	α	*ade8Δ PCLB2-MEC1::KanMX4 ndt80Δ::KanMX*	*mec1-mn ndt80AR* (TCMN)
MJ835	SK1	α	*ho::LYS2 lys2Δ arg4Δ leu2Δ::hisG trp1Δ::hisG his4XΔ::LEU2 nuc1Δ::LEU2 ura3Δ::PGPD1-GAL4(848)-ER::URA3 PGAL1-NDT80::TRP1 rad24Δ::hphMX*	*rad24Δ ndt80AR* (TRN)
MC89	S288c	a	*ade8Δ rad24Δ::HphMX4 ndt80Δ::KanMX*	*rad24Δ ndt80AR* (TRN)
MC190	SK1	α	*ho::LYS2 lys2Δ ura3Δ arg4 leu2 msh2Δ::Kan rad24Δ::HphMX4 sml1Δ::URA3*	*rad24Δ msh2Δ sml1Δ* (TRMS)
MC193	BY4741	a	*ura3Δ his4Δ leu2Δ met15Δ msh2Δ::Kan rad24Δ::HphMX4 sml1Δ::URA3*	*rad24Δ msh2Δ sml1Δ* (TRMS)
MC37	BY4742	α	*ura3Δ his4Δ leu2Δ met15Δ sml1Δ::URA3*	*sml1Δ* (TS)
MC113	SK1	a	*ho::LYS2 lys2Δ ura3Δ arg4 leu2 sml1Δ::URA3*	*sml1Δ* (TS)
MC313	S288c	a	*ade8Δ zip3Δ::HphMX4*	*zip3Δ* (TZ)
MC322	SK1	α	*ho::LYS2 lys2Δ ura3Δ arg4 leu2 zip3Δ::HphMX4*	*zip3Δ* (TZ)
MC317	S288c	a	*ade8Δ msh2Δ::Kan zip3Δ::HphMX4*	*zip3Δ msh2Δ* (TMZ)
MC326	SK1	α	*ho::LYS2 lys2Δ ura3Δ arg4 leu2 msh2Δ::Kan zip3Δ::HphMX4*	*zip3Δ msh2Δ* (TMZ)
