# Supplementary material for: Separable roles of the DNA damage response kinase Mec1ATR and its activator Rad24RAD17 during meiotic recombination
Source: PLoS Genet. 2024 Dec 9;20(12):e1011485. doi: 10.1371/journal.pgen.1011485 (PMC11658708; doi:10.1371/journal.pgen.1011485)
Supplement: S4 Table — All strains displayed are diploid. (PDF) [file pgen.1011485.s018.pdf]

Table S4

Strain	Background	Genotype
MJ512xMJ513	SK1	*ho::LYS2/' lys2Δ/' leu2/' arg4/' ura3Δ/URA3*
MJ600xMJ631	S288c	*ade8Δ/'*
MC25xMC26	SK1	*ho::LYS2 lys2Δ/' arg4/' leu2/' msh2Δ::KanMX6/'*
MC51xMC49	S288c	*ade8Δ/' msh2Δ::KanMX6/'*
MC214xMC215	SK1	*ho::LYS2/' lys2Δ/' ura3Δ/' arg4/' leu2/' mus81Δ::KanMX6/'*
MC230xMC231	SK1	*ho::LYS2/' lys2Δ/' ura3Δ/' arg4/' leu2/' mus81Δ::KanMX6/' msh2Δ::KanMX6/'*
MC254xMC255	SK1	*ho::LYS2/' lys2Δ/' ura3Δ/' arg4/' mlh3Δ::KanMX6/'*
MC271xMC272	SK1	*ho::LYS2/' lys2Δ/' ura3Δ/' arg4/' mlh3Δ::KanMX6/' mus81Δ::KanMX6/'*
MC17xMC18	SK1	*ho::LYS2/' lys2Δ/' arg4/' leu2/' rad24Δ::HphMX4/'*
MC19xMC20	S288c	*ade8Δ/' rad24Δ::HphMX4/'*
MC107xMC105	SK1	*ho::LYS2/' lys2Δ/' ura3Δ/' arg4/' leu2/' rad24Δ::HphMX4/' msh2Δ::KanMX6/'*
MC202xMC203	S288c	*ade8Δ/' rad24Δ::HphMX4/' msh2Δ::KanMX6/'*
MC223xMC222	SK1	*ho::LYS2/' lys2Δ/' ura3Δ/' arg4/' leu2/' mus81Δ::KanMX6/' rad24::hphMX/'*
MC247xMC248	SK1	*ho::LYS2/' lys2Δ/' ura3Δ/' arg4/' rad24Δ::HphMX4/' mlh3Δ::KanMX6/'*
MC257xMC258	SK1	*ho::LYS2/' lys2Δ/' ura3Δ/' arg4/' rad24Δ::HphMX4/' mus81Δ::KanMX6/' mlh3Δ::KanMX6/'*
MC101xMC103	SK1	*ho::LYS2/' lys2Δ/' ura3Δ/' arg4/' leu2/' rad24Δ::HphMX4/' sml1Δ::URA3/'*
MC59xMC61	BY474	*his4/' ura3Δ/' leu2Δ/' met15Δ/' sml1Δ::URA3/' rad24Δ::HphMX4/'*
VG120xVG121	SK1	*ho::LYS2/' lys2Δ/' ura3Δ/' arg4Δ/' leu2Δ::hisG/' his4XΔ::LEU2/' nuc1Δ::LEU2/' ade2-bgl/' PCLB2-MEC1::KanMX6/'*
MC152xMC153	S288c	*ade8Δ/' PCLB2-MEC1::KanMX6/'*
MC163xMC164	SK1	*ho::LYS2/' lys2Δ/' ura3Δ/' arg4Δ/' leu2Δ::hisG/' nuc1Δ::LEU2/' PCLB2-MEC1::KanMX6/' msh2Δ::KanMX6/'*
MC171xMC172	S288c	*ade8Δ/' PCLB2-MEC1::KanMX6/' msh2::HphMX4/'*
MC243xMC242	SK1	*ho::LYS2/' lys2Δ/' ura3Δ/' arg4/' leu2/' mus81Δ::KanMX6/' PCLB2-MEC1::KanMX6/'*
MC2xMC3	SK1	*ho::LYS2/' lys2Δ/' arg4Δ/' leu2Δ::hisG/' his4XΔ::LEU2/' nuc1Δ::LEU2/' trp1Δ::hisG/' ura3Δ::PGPD1-GAL4(848)-ER::URA3/' PCLB2-MEC1::KanMX6/' PGAL1-NDT80::TRP1/'*
MJ848	SK1	*ho::LYS2/' lys2Δ/' arg4Δ/' leu2Δ::hisG/' trp1Δ::hisG/' his4XΔ::LEU2/' nuc1Δ::LEU2/' ura3Δ::PGPD1-GAL4(848)-ER::URA3/' PGAL1-NDT80::TRP1/' rad24Δ::hphMX/'*
MC288xMC289	SK1	*ho::LYS2/' lys2Δ/' arg4/' leu2/' trp1Δ::hisG/' his4XΔ::LEU2/' nuc1Δ::LEU2/' ura3Δ::PGPD1-GAL4(848)-ER::URA3/' PGAL1-NDT80::TRP/' mus81Δ::KanMX6/' rad24Δ::HphMX4/'*
MC286xMC287	SK1	*ho::LYS2/' lys2Δ/' arg4/' leu2Δ' trp1Δ::hisG/' his4XΔ::LEU2/' nuc1Δ::LEU2/' ura3Δ::PGPD1-GAL4(848)-ER::URA3/' PGAL1-NDT80::TRP/' mus81Δ::KanMX6/'*
MC292xMC297	SK1/S288c	*ho::LYS2/ho lys2Δ/LYS2 ade8Δ/ADE8 ura3Δ/URA3 arg4Δ/ARG4 leu2Δ/LEU2 msh2Δ::KanMX6/' mus81::KanMX6/' rad24::HphMX4/'*
MC307xMC309	SK1	*ho::LYS2/' lys2Δ/' ura3Δ/' arg4Δ/' leu2Δ::hisG/' trp1::hisG/' his4XΔ::LEU2/' nuc1Δ::LEU2/' 'ura3Δ::PGPD1-GAL4(848)-ER::URA3/' PGAL1-NDT80::TRP1/'rad24Δ::hphMX/' mlh3Δ::KanMX6/'*
MC303xMC304	SK1	*ho::LYS2/' lys2Δ/' ura3Δ/' arg4Δ/' leu2Δ::hisG/' his4XΔ::LEU2/' nuc1Δ::LEU2/' PCLB2-MEC1::KanMX6/' mlh3Δ::KanMX6/'*
MC333xMC304	SK1	*ho::LYS2/' lys2Δ/' ura3Δ/' arg4Δ/' leu2Δ::hisG/' his4XΔ::LEU2/' nuc1Δ::LEU2/' PCLB2-MEC1::KanMX6/' mlh3Δ::KanMX6/' mus81::hphMX/'*
MJ848	SK1	*ho::LYS2/'' lys2Δ/' argΔ/' leu2Δ::hisG/' trp1Δ::hisG/' his4XΔ::LEU2/' nuc1Δ::LEU2/' ura3Δ::PGPD1GAL4(848)-ER::URA3/' PGAL1-NDT80::TRP1/' rad24Δ::HphMX4/'*
MC4	SK1	*ho::LYS2/' lys2Δ/' arg4Δ/' leu2Δ::hisG/' his4XΔ::LEU2/' nuc1Δ::LEU2/' trp1Δ::hisG/' ura3Δ::pGPD1GAL4(848)-ER::URA3/' PCLB2-MEC1::Kan/' PGAL1-NDT80::TRP1/'*
