# Supplementary material for: Separable roles of the DNA damage response kinase Mec1ATR and its activator Rad24RAD17 during meiotic recombination
Source: PLoS Genet. 2024 Dec 9;20(12):e1011485. doi: 10.1371/journal.pgen.1011485 (PMC11658708; doi:10.1371/journal.pgen.1011485)
Supplement: S3 Data — Example event images for one of the meioses studied. (PDF) [file pgen.1011485.s021.pdf]

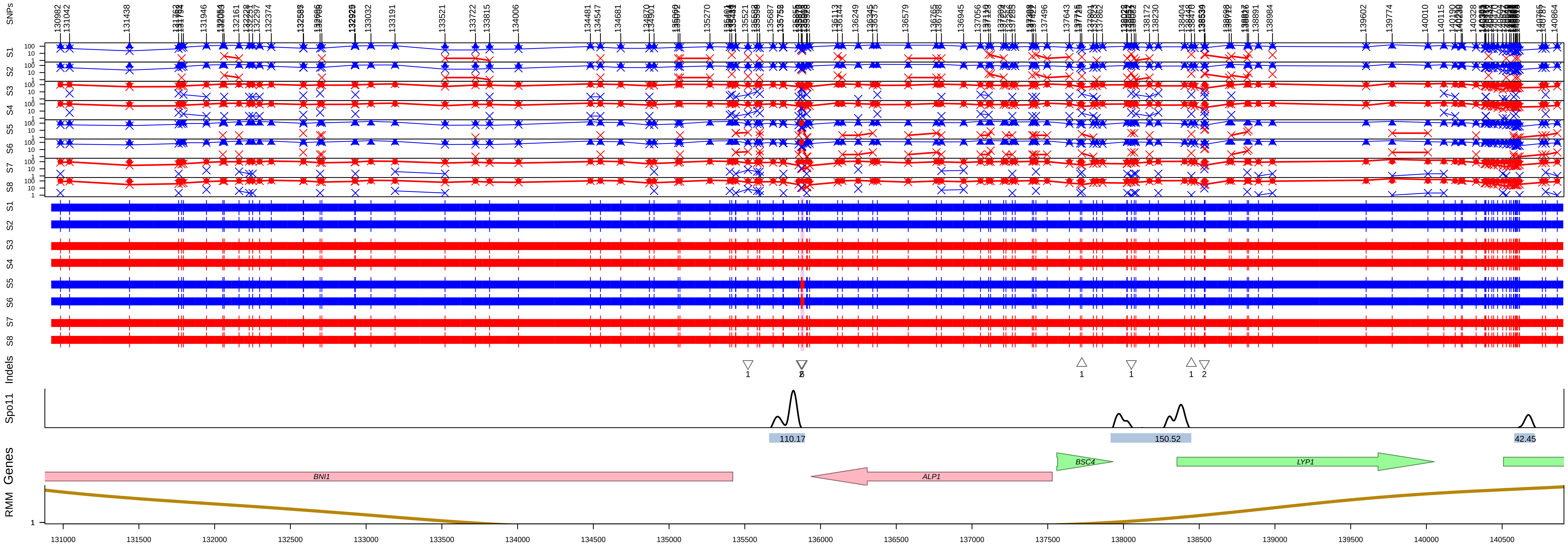

Genotype=TCMN6 Id=97 Chromo=14 Type=Full Conversion
Estimated: DSBs=1, COs=0, NCOs=1
Pattern=6:2
Mid Len=26bp Midpoint=135880.25 Variants=1
Red=S288c, Blue=SK1

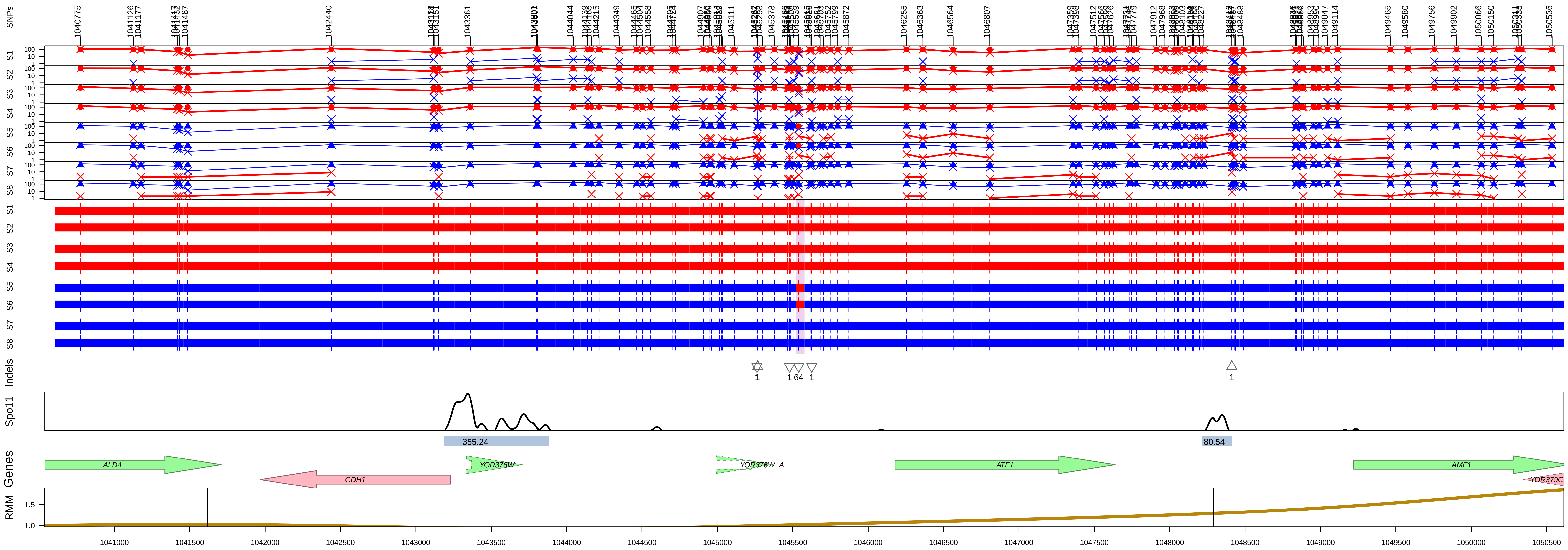

Genotype=TCMN6 Id=123 Chromo=15 Type=Full Conversion
Estimated: DSBs=1, COs=0, NCOs=1
Pattern=6:2
Mid Len=54bp Midpoint=1045550.25 Variants=1
Red=S288c, Blue=SK1

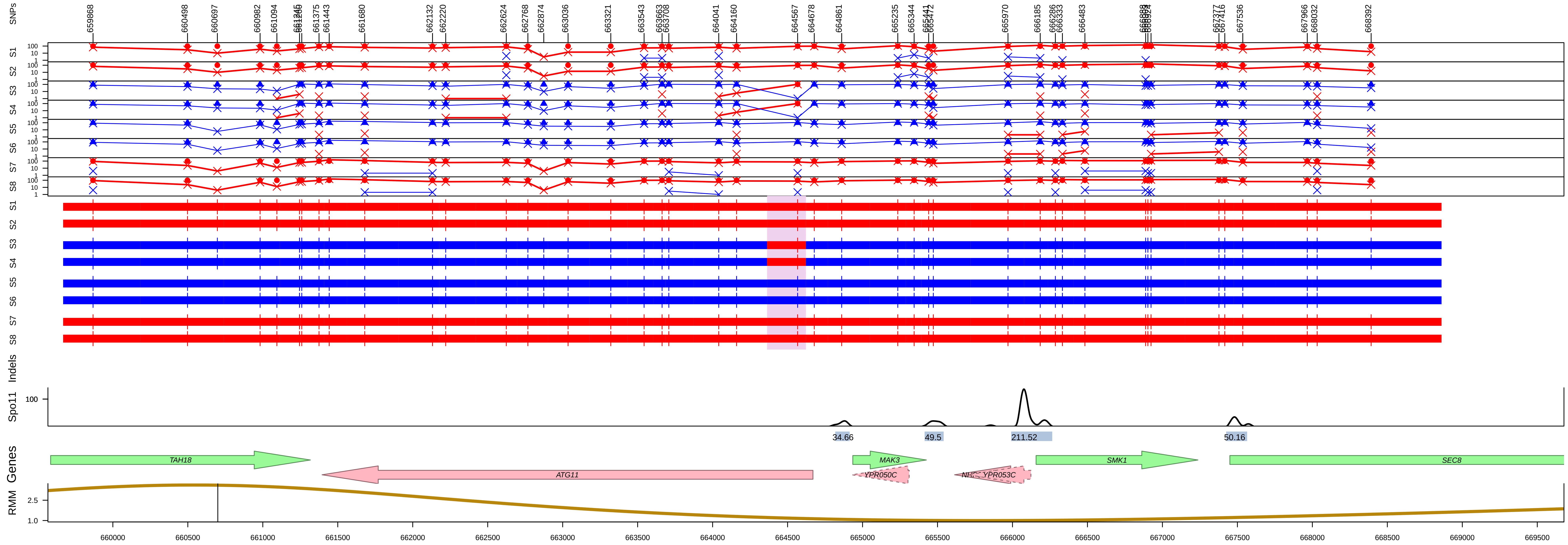

Genotype=TCMN6 Id=131 Chromo=16 Type=Full Conversion
Estimated: DSBs=1, COs=0, NCOs=1
Pattern=6:2
Mid Len=259bp Midpoint=664493 Variants=1
Red=S288c, Blue=SK1

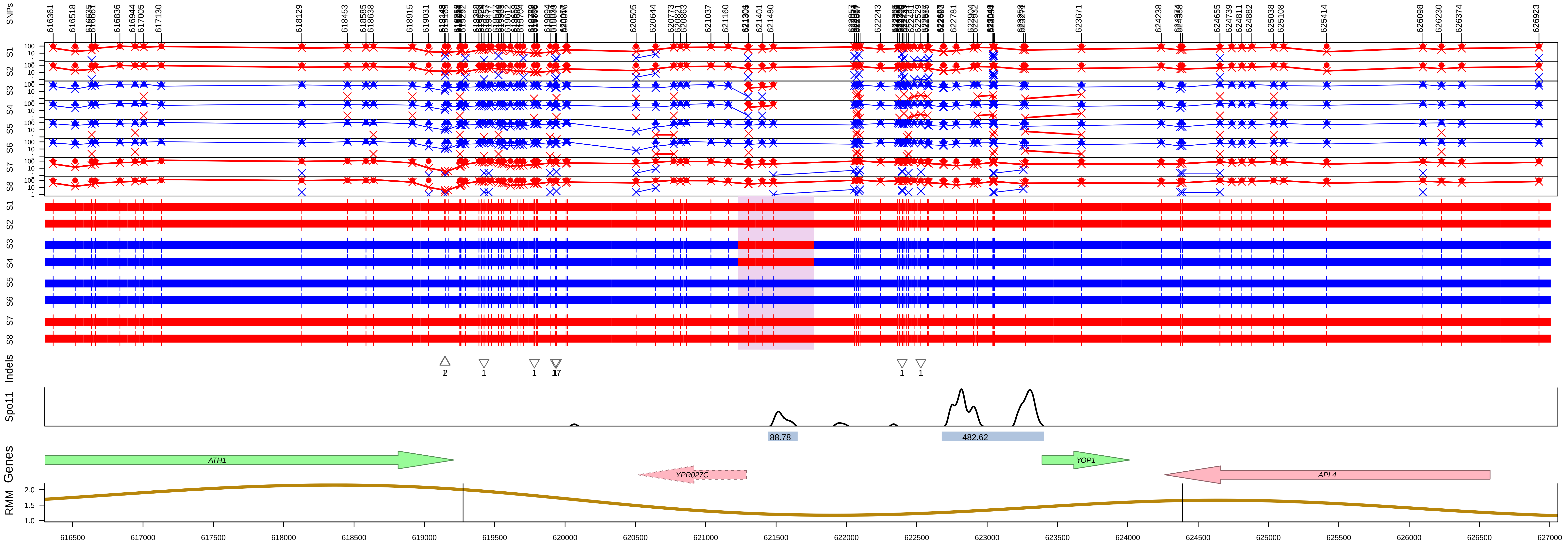

Genotype=TCMN6 Id=130 Chromo=16 Type=Full Conversion
Estimated: DSBs=1, COs=0, NCOs=1
Pattern=6:2
Mid Len=538bp Midpoint=621499.5 Variants=4
Red=S288c, Blue=SK1

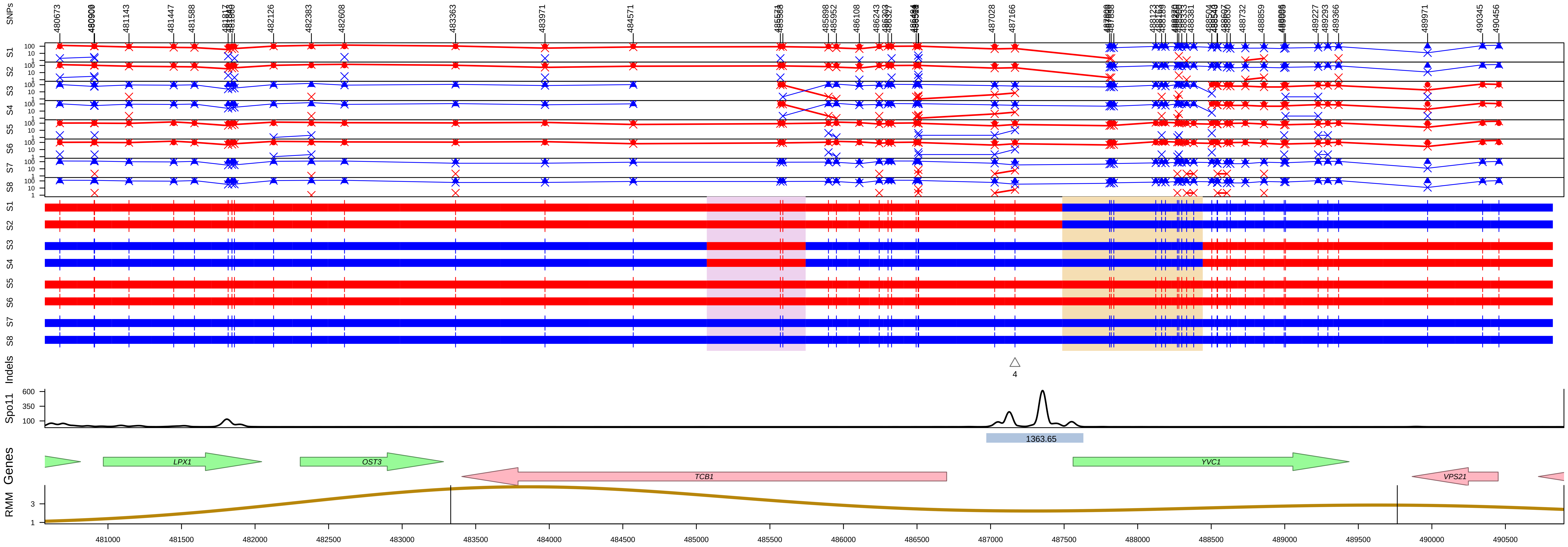

Genotype=TCMN6 Id=115 Chromo=15 Type=Full Conversion
Estimated: DSBs=1, COs=0, NCOs=1
Pattern=6:2
Mid Len=672bp Midpoint=485407 Variants=2
Red=S288c, Blue=SK1

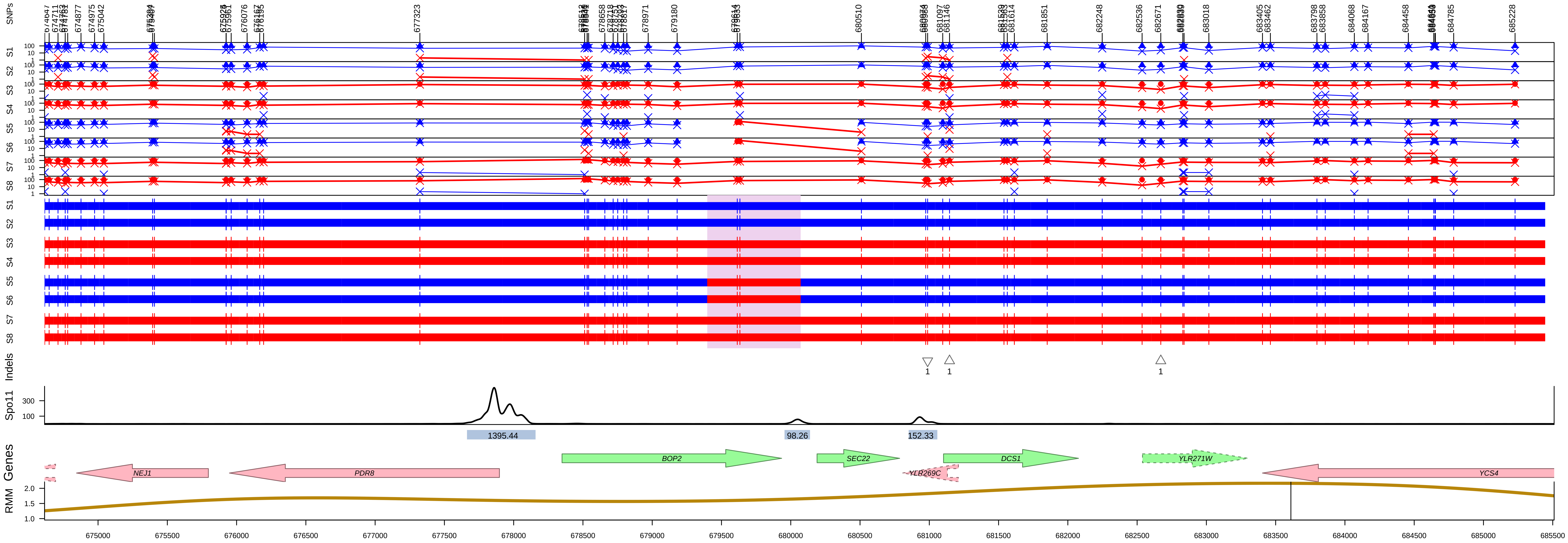

Genotype=TCMN6 Id=80 Chromo=12 Type=Full Conversion
Estimated: DSBs=1, COs=0, NCOs=1
Pattern=6:2
Mid Len=674bp Midpoint=679734.25 Variants=2
Red=S288c, Blue=SK1

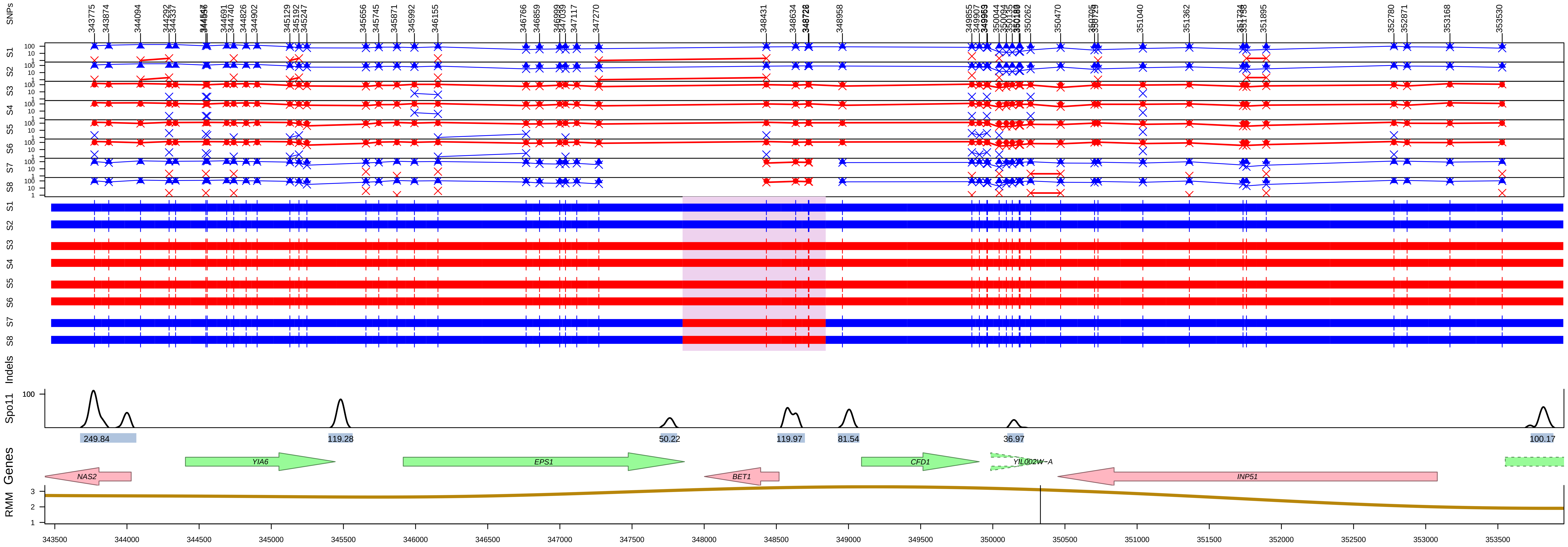

Genotype=TCMN6 Id=59 Chromo=9 Type=Full Conversion
Estimated: DSBs=1, COs=0, NCOs=1
Pattern=6:2
Mid Len=992bp Midpoint=348346.25 Variants=4
Red=S288c, Blue=SK1

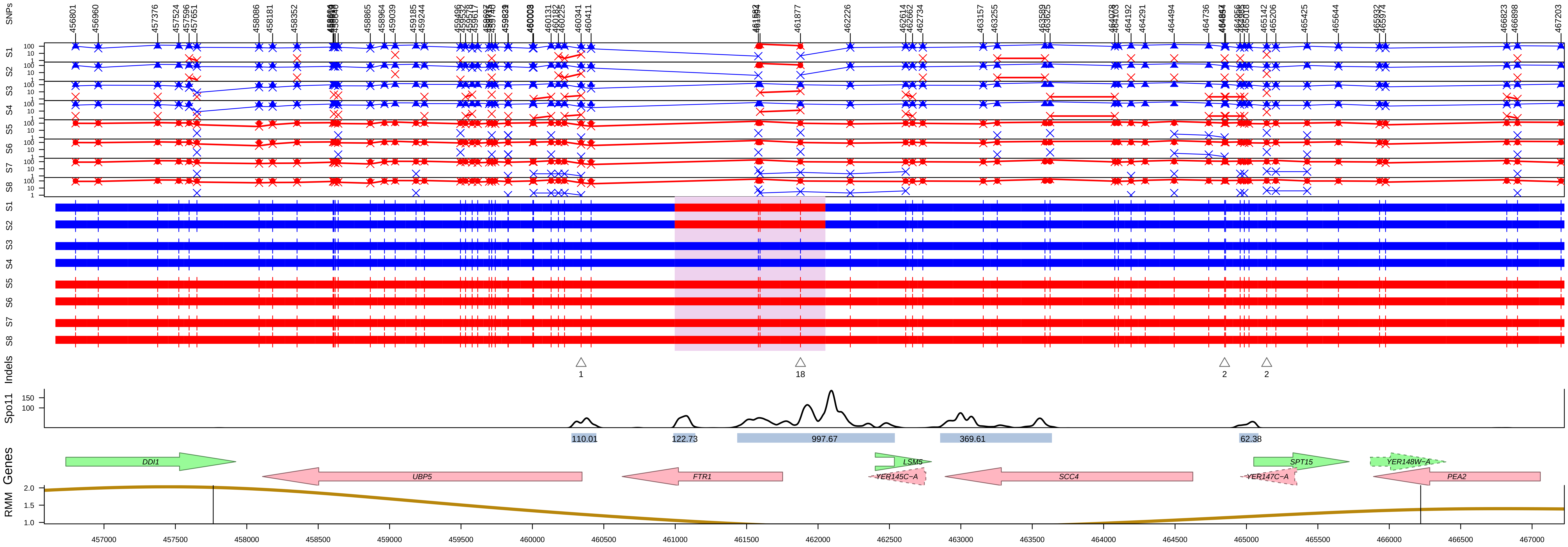

Genotype=TCMN6 Id=28 Chromo=5 Type=Full Conversion
Estimated: DSBs=1, COs=0, NCOs=1
Pattern=6:2
Mid Len=1055bp Midpoint=461524 Variants=3
Red=S288c, Blue=SK1

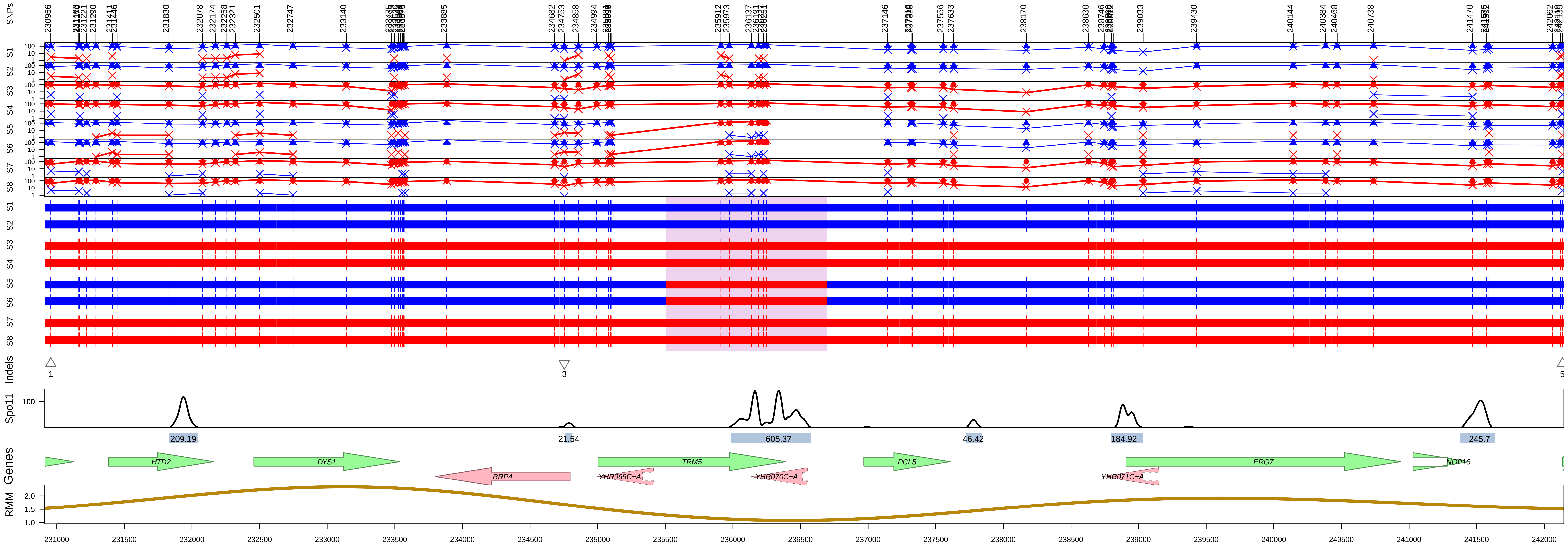

Genotype=TCMN6 Id=51 Chromo=8 Type=Full Conversion
Estimated: DSBs=1, COs=0, NCOs=1
Pattern=6:2
Mid Len=1193bp Midpoint=236102 Variants=6
Red=S288c, Blue=SK1

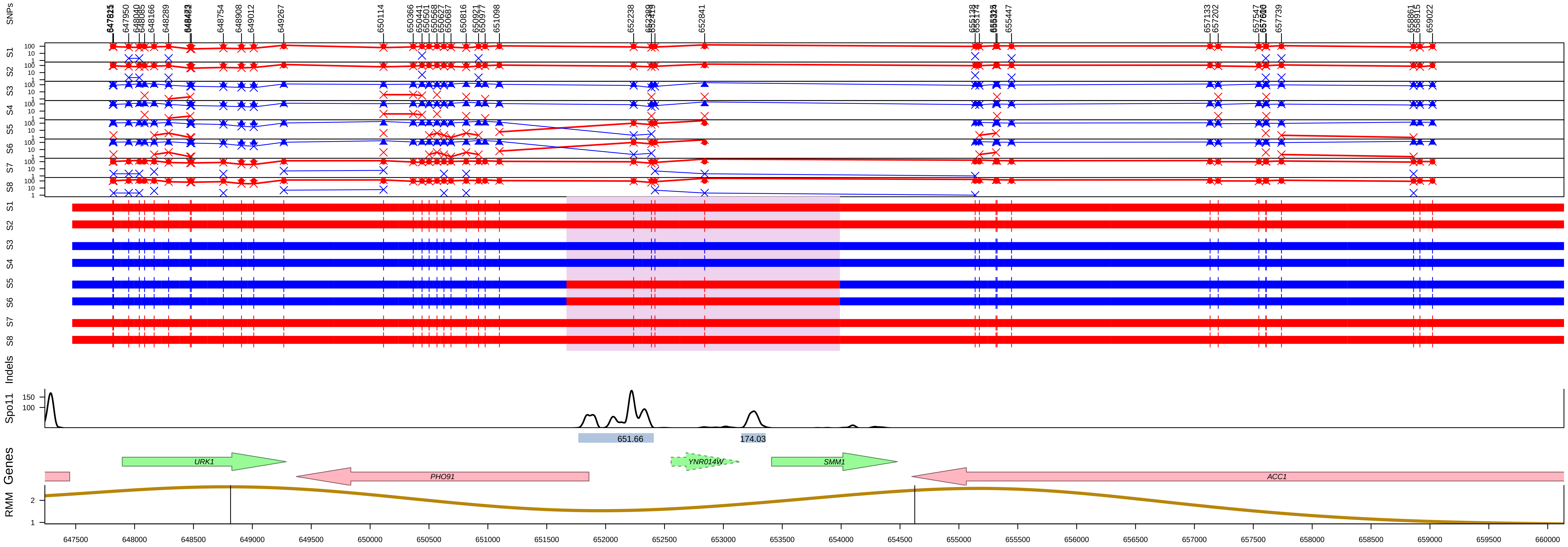

Genotype=TCMN6 Id=104 Chromo=14 Type=Full Conversion
Estimated: DSBs=1, COs=0, NCOs=1
Pattern=6:2
Mid Len=2322bp Midpoint=652828.75 Variants=4
Red=S288c, Blue=SK1

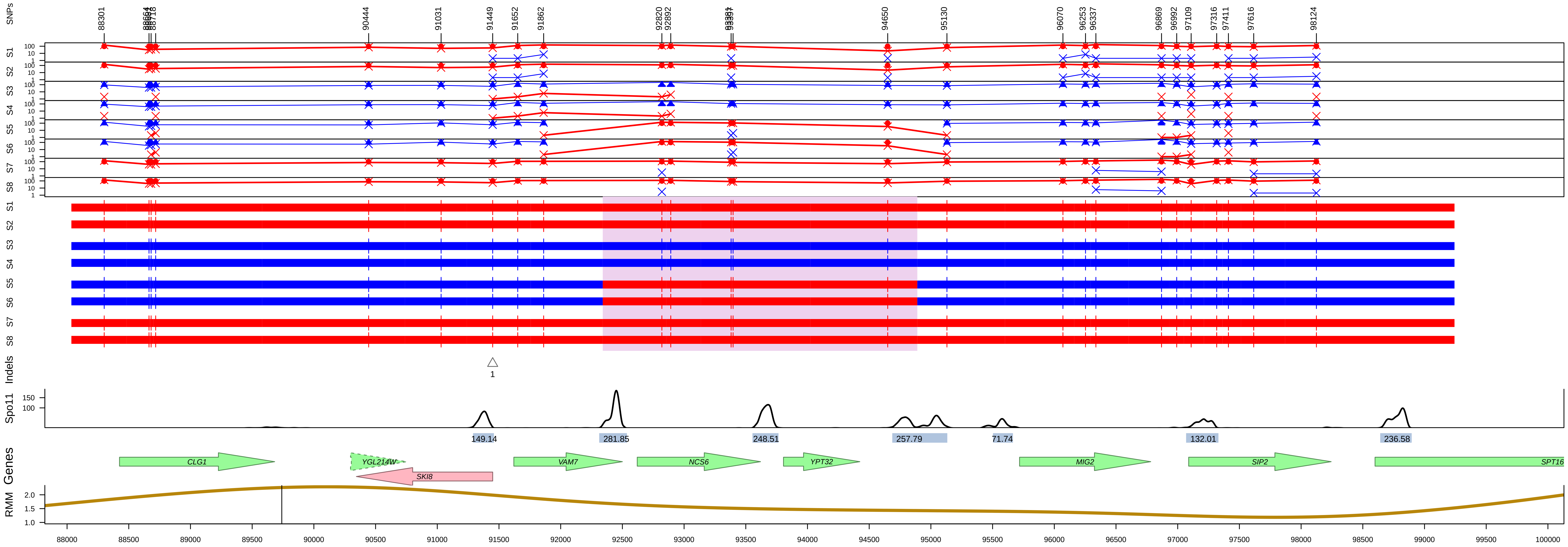

Genotype=TCMN6 Id=37 Chromo=7 Type=Full Conversion
Estimated: DSBs=1, COs=0, NCOs=1
Pattern=6:2
Mid Len=2549bp Midpoint=93615.5 Variants=5
Red=S288c, Blue=SK1

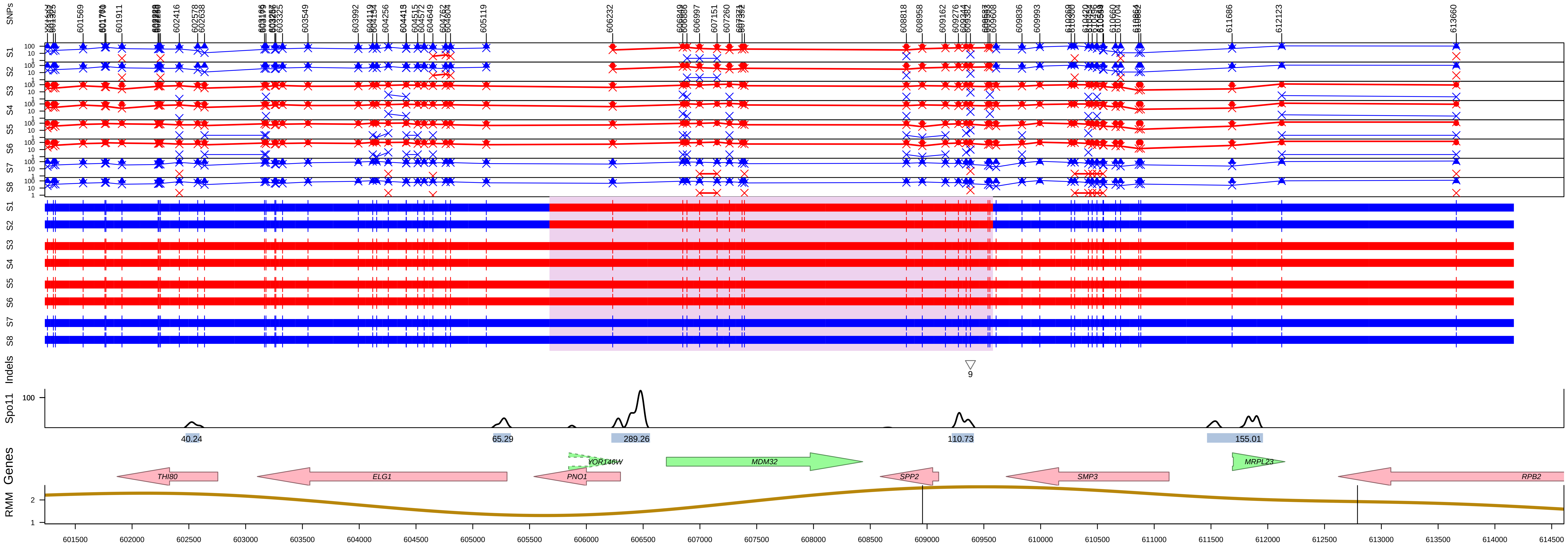

Genotype=TCMN6 Id=118 Chromo=15 Type=Full Conversion
Estimated: DSBs=1, COs=0, NCOs=1
Pattern=6:2
Mid Len=3905bp Midpoint=607628 Variants=16
Red=S288c, Blue=SK1

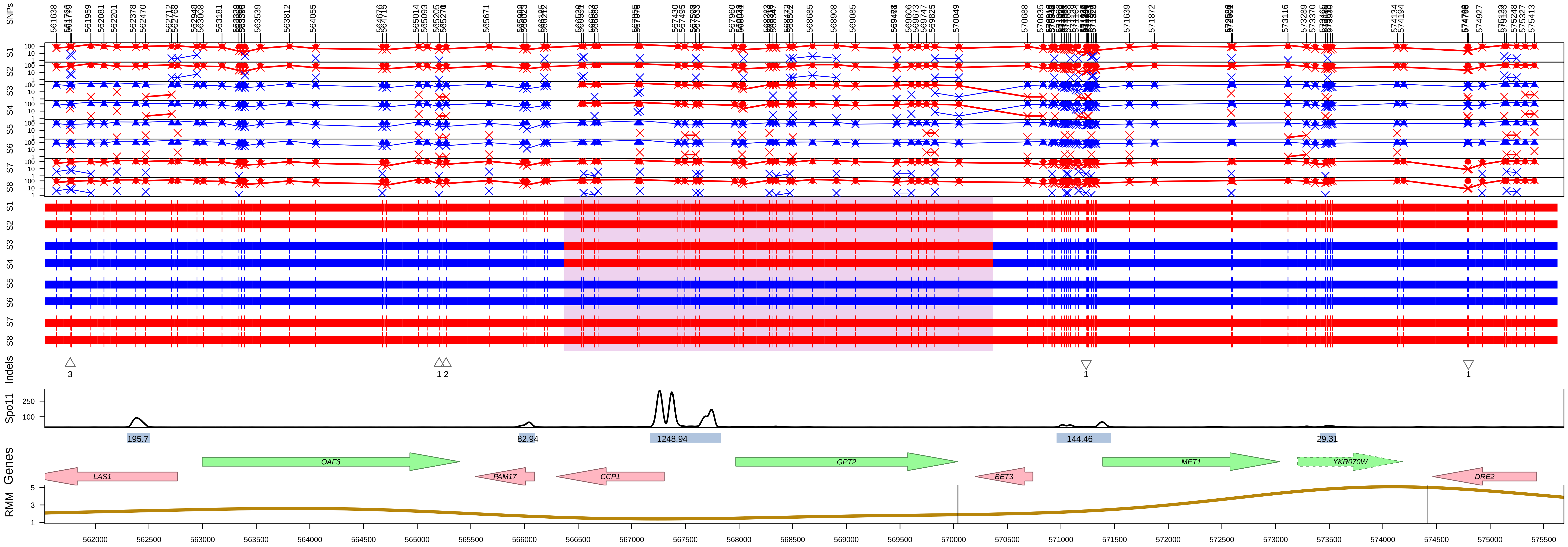

Genotype=TCMN6 Id=73 Chromo=11 Type=Full Conversion
Estimated: DSBs=1, COs=0, NCOs=1
Pattern=6:2
Mid Len=3998bp Midpoint=568369.75 Variants=29
Red=S288c, Blue=SK1

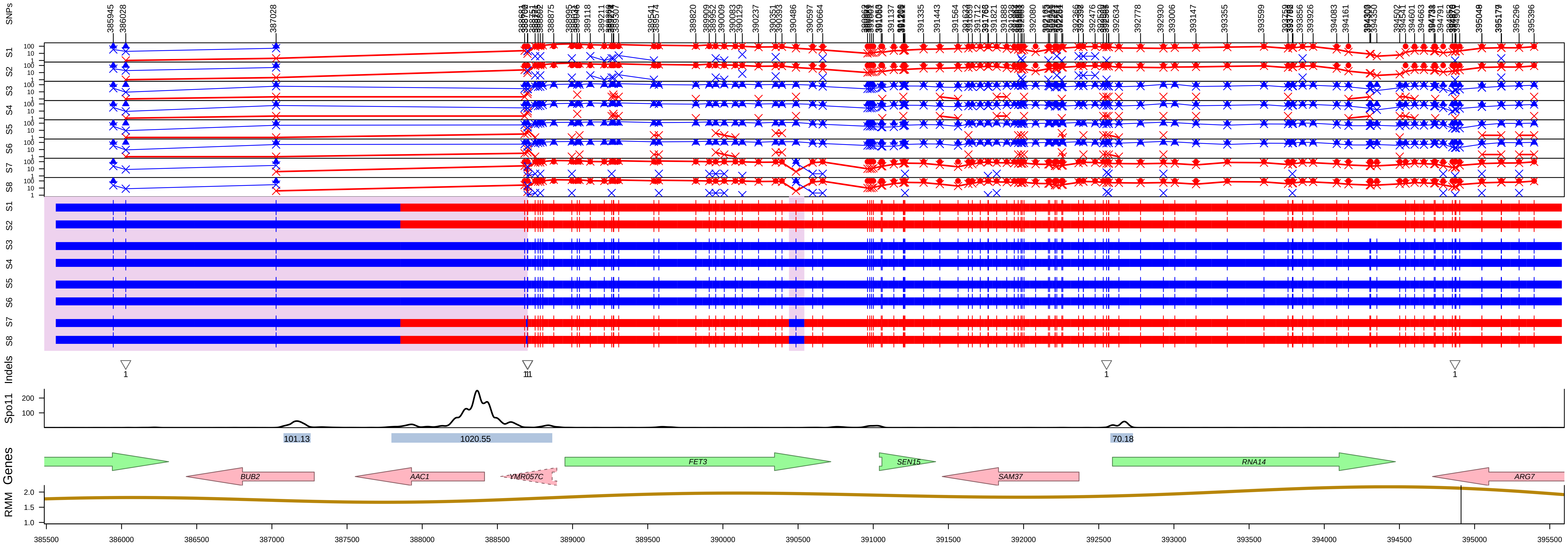

Genotype=TCMN6 Id=86 Chromo=13 Type=Full Conversion
Estimated: DSBs=1, COs=0, NCOs=1
Pattern=2:6
Mid Len=102bp Midpoint=390490.5 Variants=1
Red=S288c, Blue=SK1

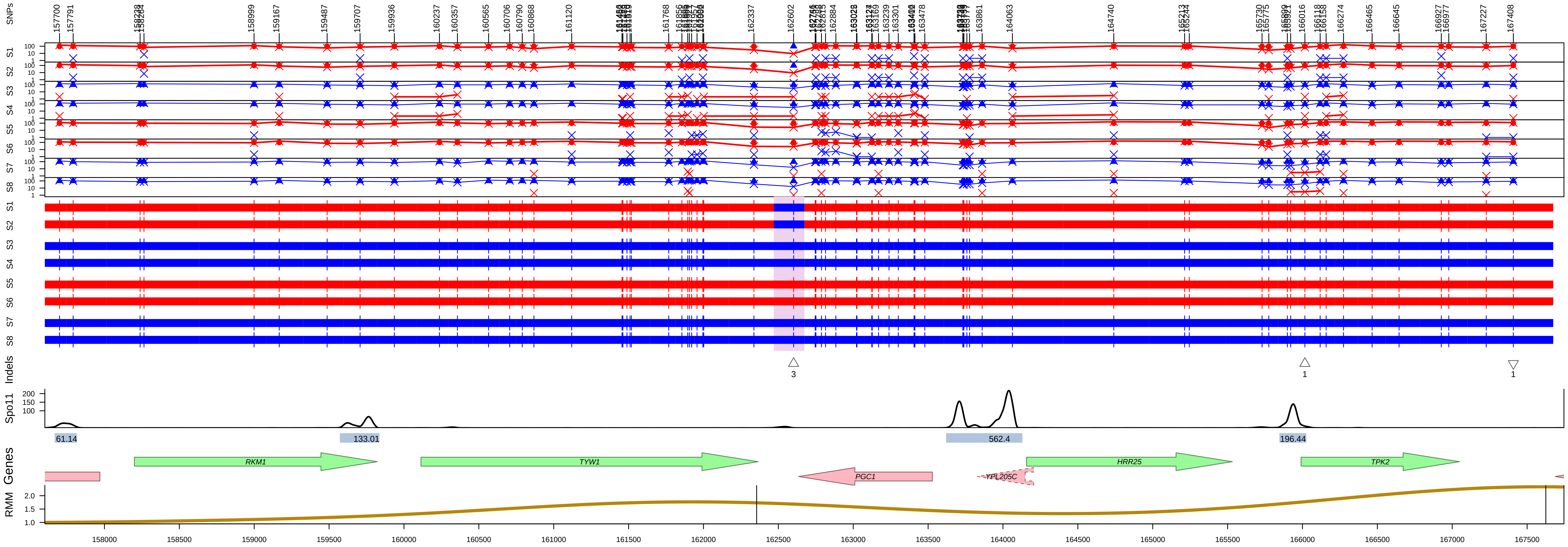

Genotype=TCMN6 Id=124 Chromo=16 Type=Full Conversion
Estimated: DSBs=1, COs=0, NCOs=1
Pattern=2:6
Mid Len=204bp Midpoint=162571.75 Variants=1
Red=S288c, Blue=SK1

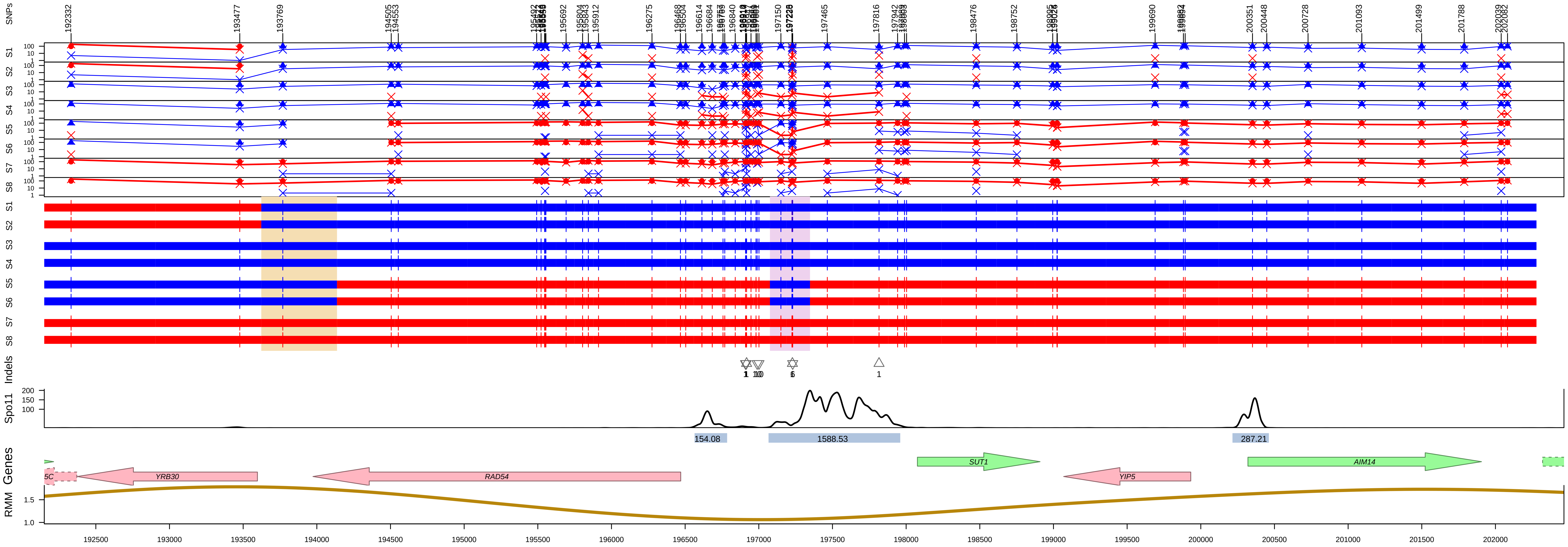

Genotype=TCMN6 Id=40 Chromo=7 Type=Full Conversion
Estimated: DSBs=1, COs=0, NCOs=1
Pattern=2:6
Mid Len=272bp Midpoint=197211.5 Variants=3
Red=S288c, Blue=SK1

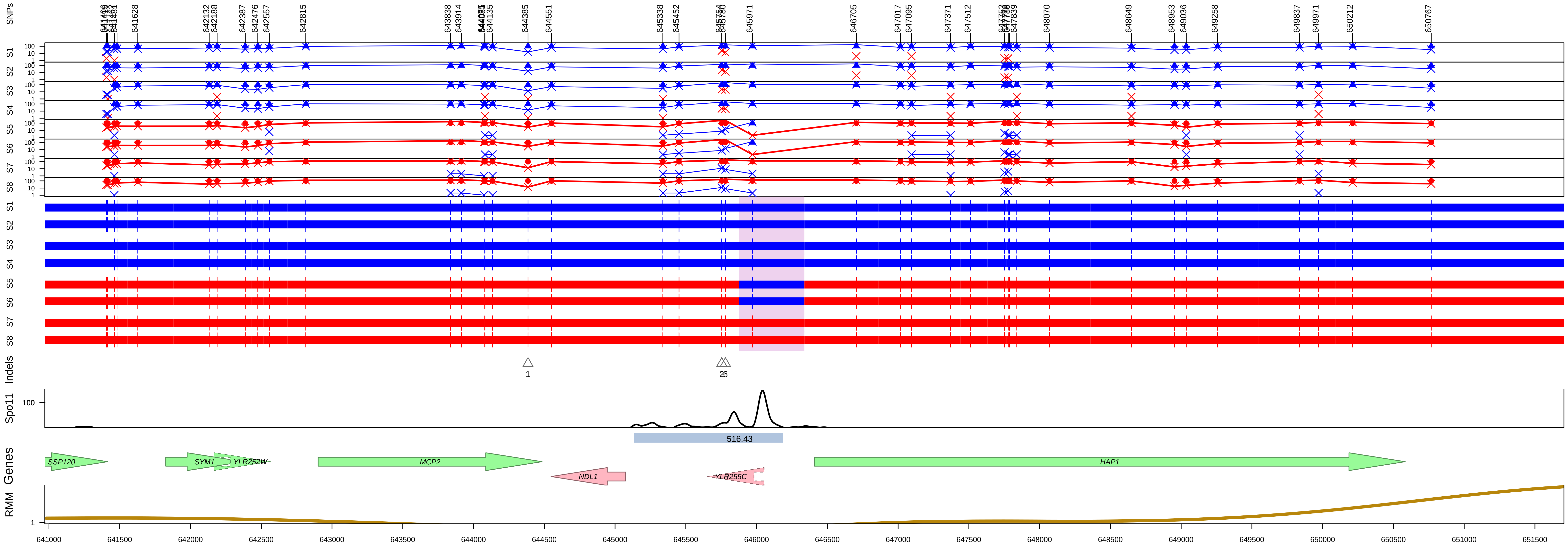

Genotype=TCMN6 Id=78 Chromo=12 Type=Full Conversion
Estimated: DSBs=1, COs=0, NCOs=1
Pattern=2:6
Mid Len=462bp Midpoint=646106.75 Variants=1
Red=S288c, Blue=SK1

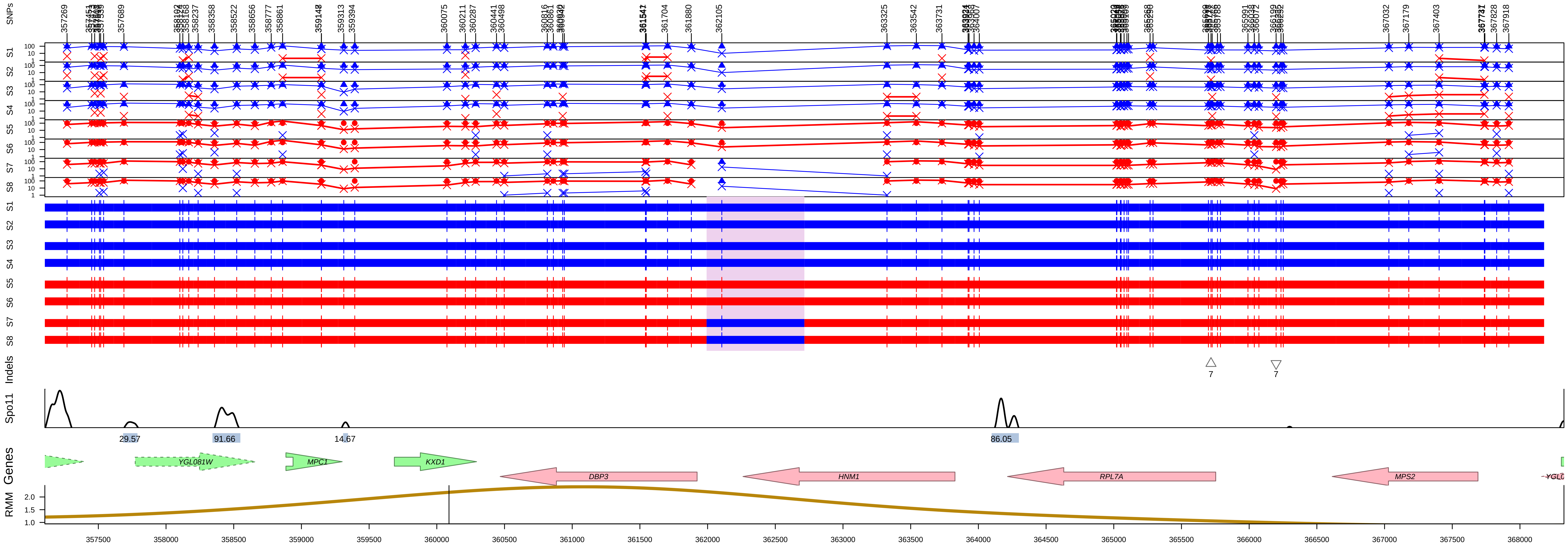

Genotype=TCMN6 Id=44 Chromo=7 Type=Full Conversion
Estimated: DSBs=1, COs=0, NCOs=1
Pattern=2:6
Mid Len=722bp Midpoint=362353.75 Variants=1
Red=S288c, Blue=SK1

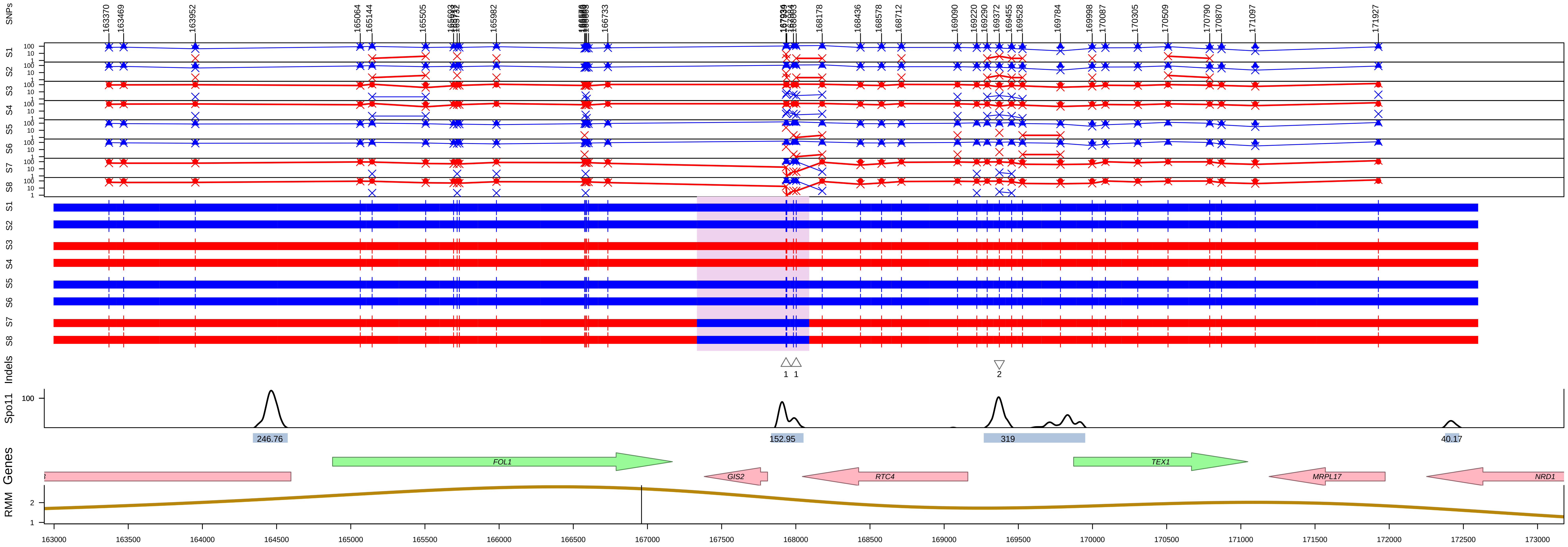

Genotype=TCMN6 Id=98 Chromo=14 Type=Full Conversion
Estimated: DSBs=1, COs=0, NCOs=1
Pattern=2:6
Mid Len=757bp Midpoint=167712 Variants=4
Red=S288c, Blue=SK1

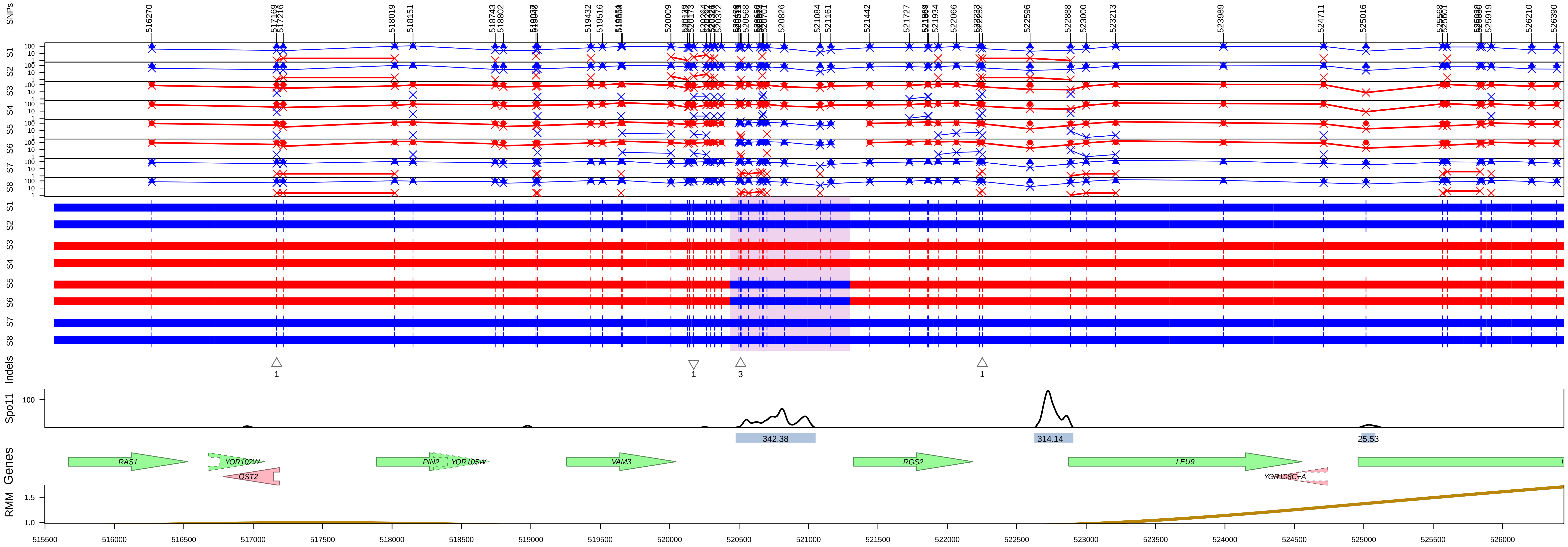

Genotype=TCMN6 Id=117 Chromo=15 Type=Full Conversion
Estimated: DSBs=1, COs=0, NCOs=1
Pattern=2:6
Mid Len=866bp Midpoint=520868.5 Variants=10
Red=S288c, Blue=SK1

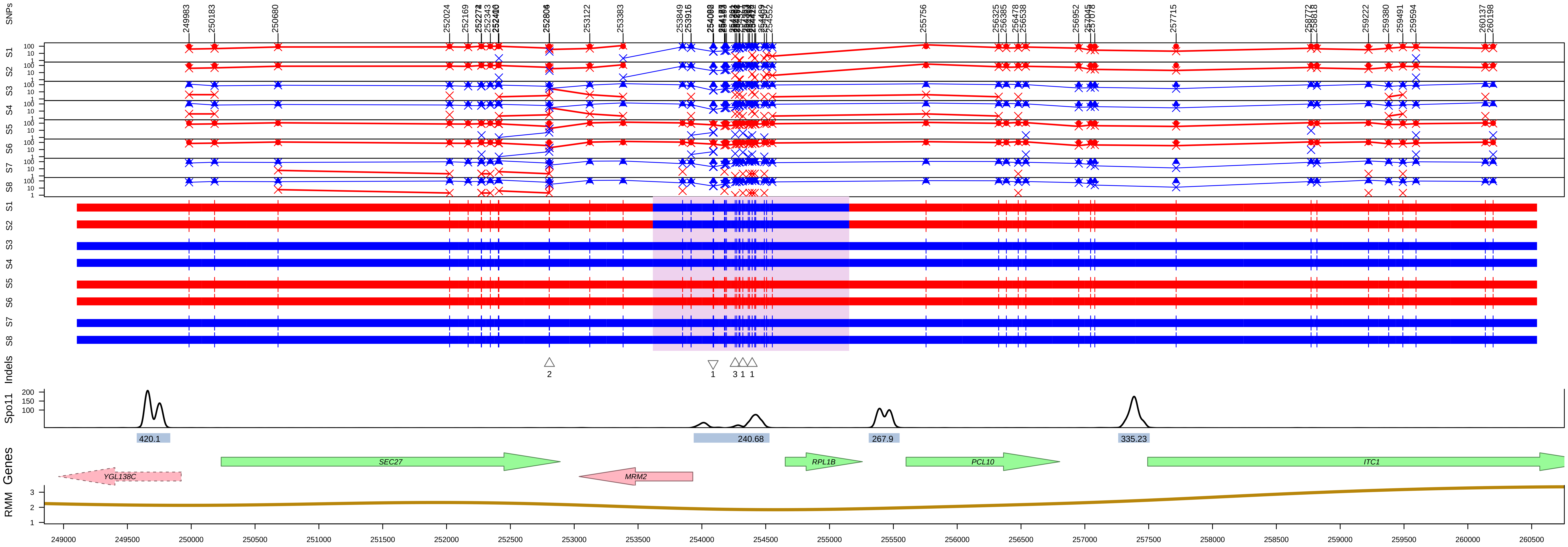

Genotype=TCMN6 Id=42 Chromo=7 Type=Full Conversion
Estimated: DSBs=1, COs=0, NCOs=1
Pattern=2:6
Mid Len=1538bp Midpoint=254385 Variants=21
Red=S288c, Blue=SK1

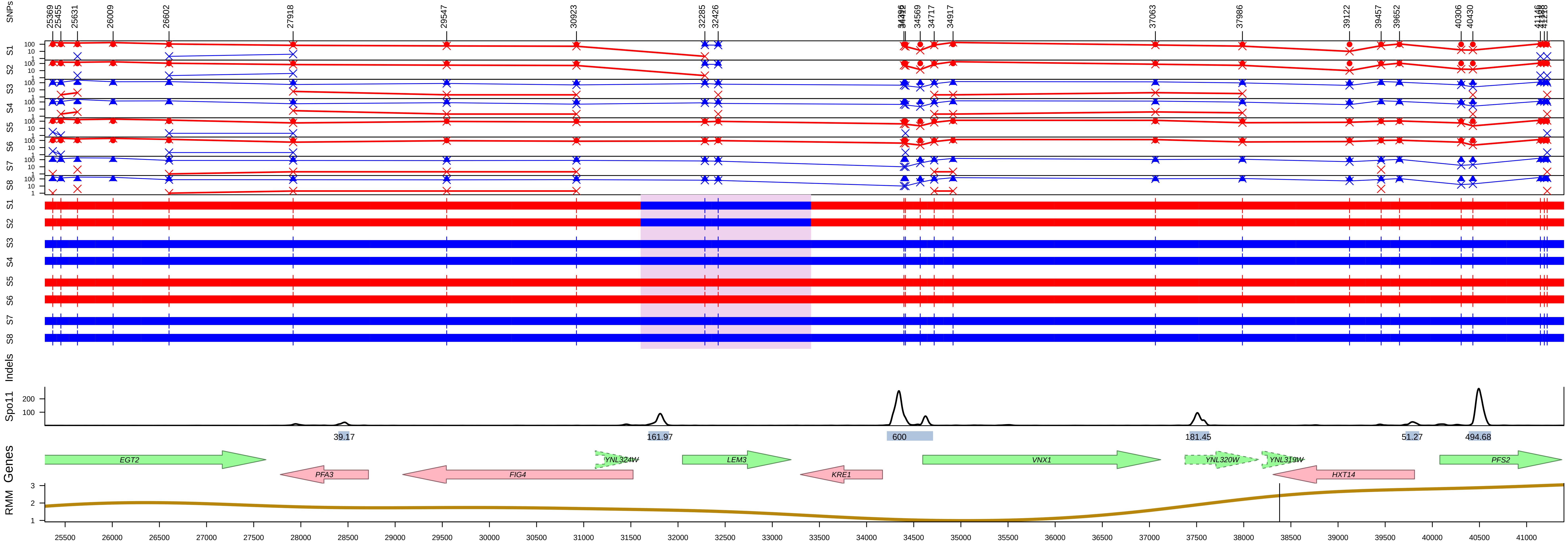

Genotype=TCMN6 Id=93 Chromo=14 Type=Full Conversion
Estimated: DSBs=1, COs=0, NCOs=1
Pattern=2:6
Mid Len=1807bp Midpoint=32507.5 Variants=2
Red=S288c, Blue=SK1

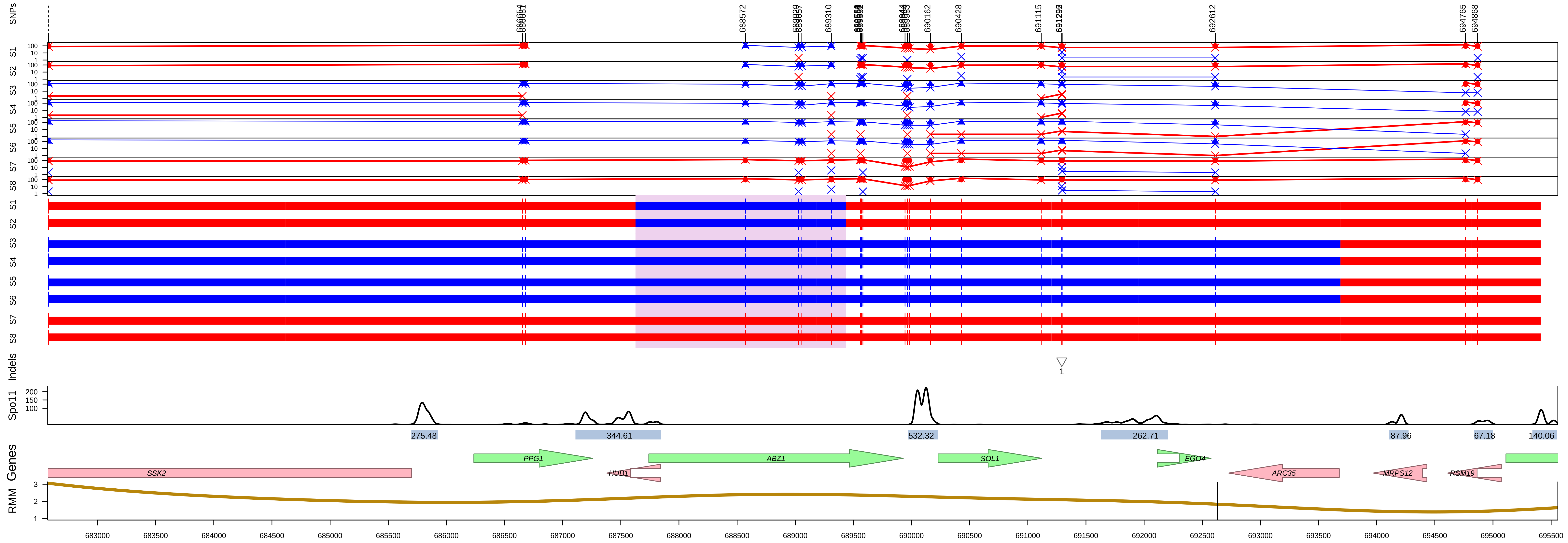

Genotype=TCMN6 Id=105 Chromo=14 Type=Full Conversion
Estimated: DSBs=1, COs=0, NCOs=1
Pattern=2:6
Mid Len=1808bp Midpoint=688530.25 Variants=4
Red=S288c, Blue=SK1

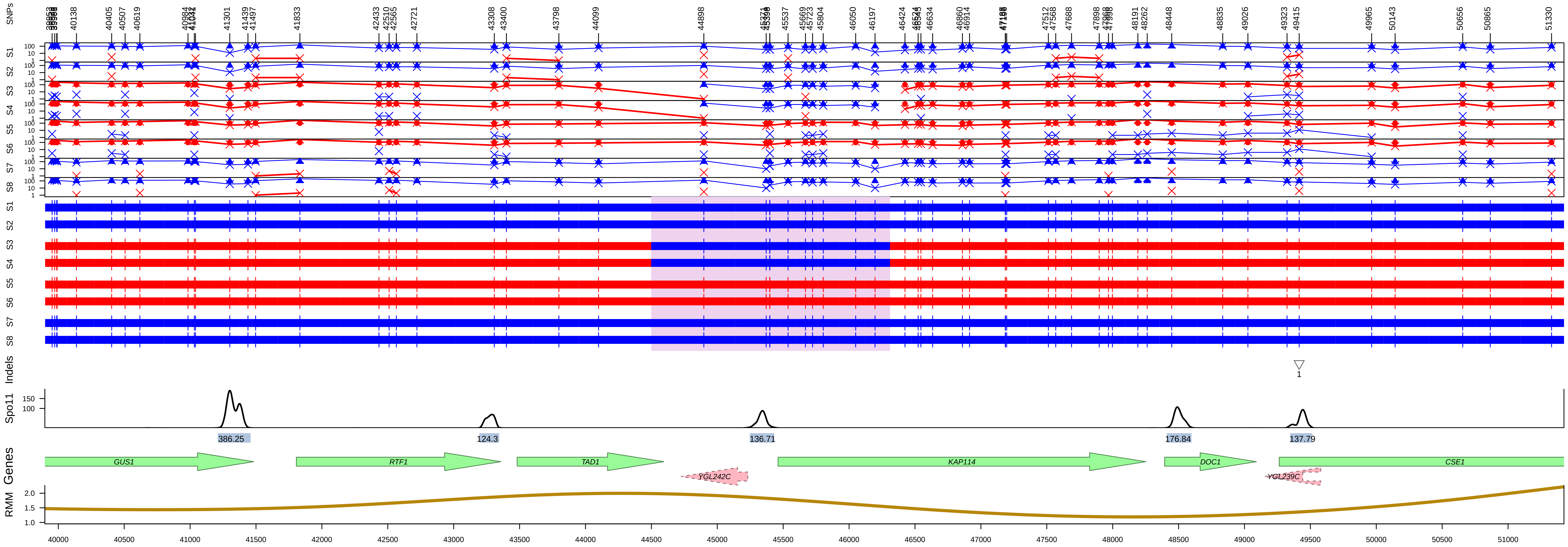

Genotype=TCMN6 Id=35 Chromo=7 Type=Full Conversion
Estimated: DSBs=1, COs=0, NCOs=1
Pattern=2:6
Mid Len=1812bp Midpoint=45404.5 Variants=10
Red=S288c, Blue=SK1

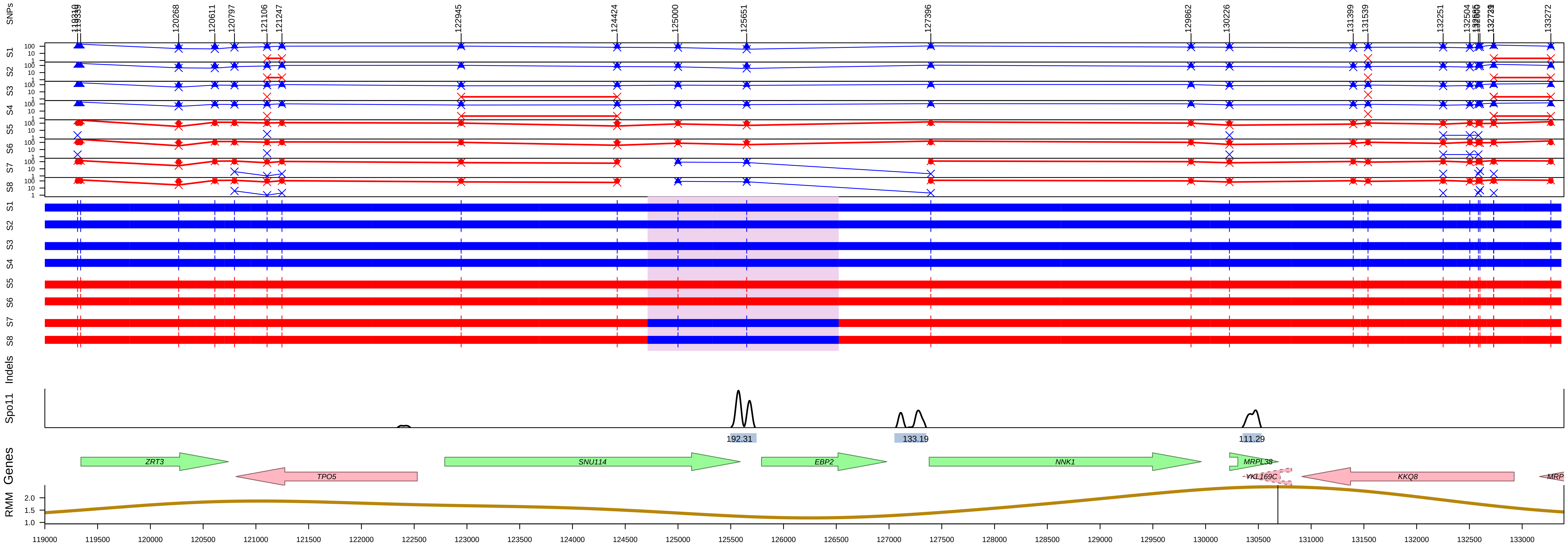

Genotype=TCMN6 Id=67 Chromo=11 Type=Full Conversion
Estimated: DSBs=1, COs=0, NCOs=1
Pattern=2:6
Mid Len=1812bp Midpoint=125617.75 Variants=2
Red=S288c, Blue=SK1

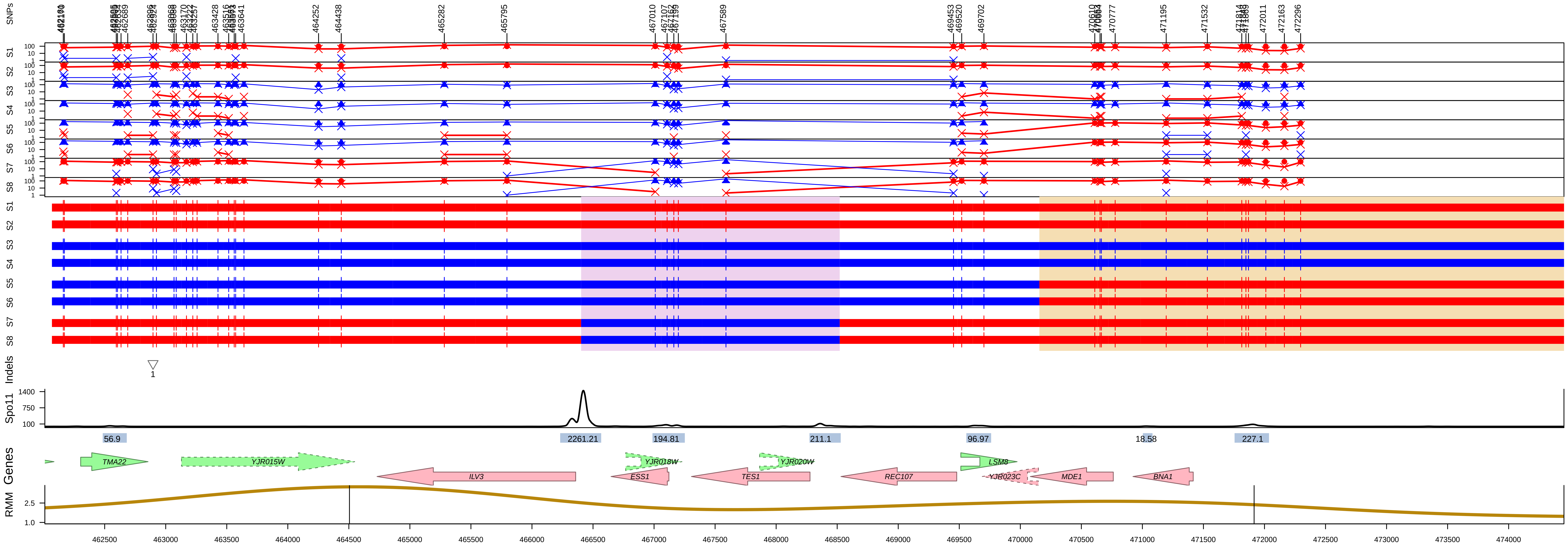

Genotype=TCMN6 Id=64 Chromo=10 Type=Full Conversion
Estimated: DSBs=1, COs=0, NCOs=1
Pattern=2:6
Mid Len=2118bp Midpoint=467461.75 Variants=5
Red=S288c, Blue=SK1

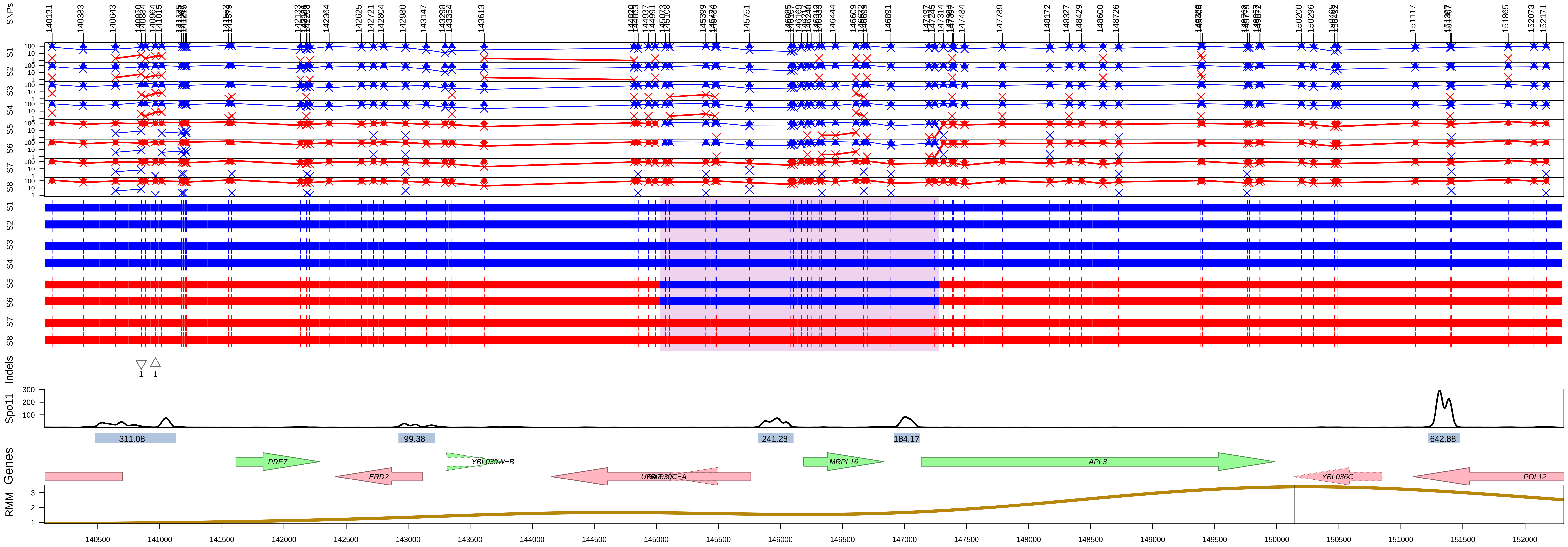

Genotype=TCMN6 Id=7 Chromo=2 Type=Full Conversion
Estimated: DSBs=1, COs=0, NCOs=1
Pattern=2:6
Mid Len=2248bp Midpoint=146155.75 Variants=20
Red=S288c, Blue=SK1

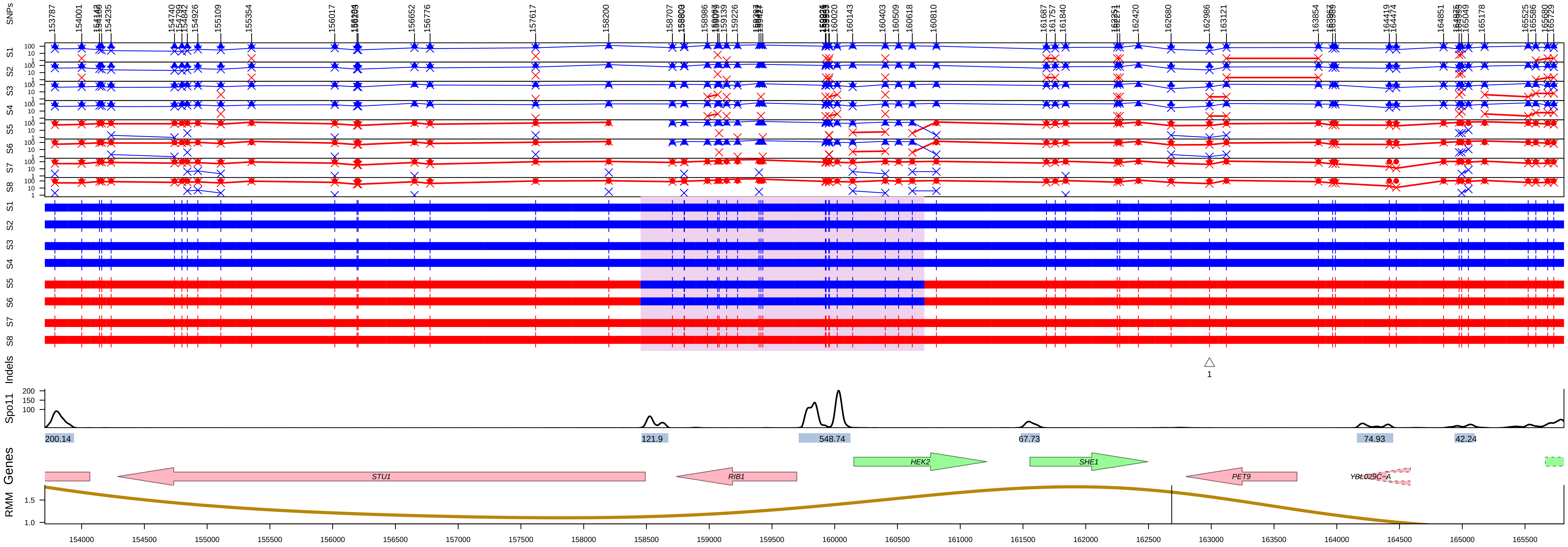

Genotype=TCMN6 Id=8 Chromo=2 Type=Full Conversion
Estimated: DSBs=1, COs=0, NCOs=1
Pattern=2:6
Mid Len=2260bp Midpoint=159583.75 Variants=20
Red=S288c, Blue=SK1

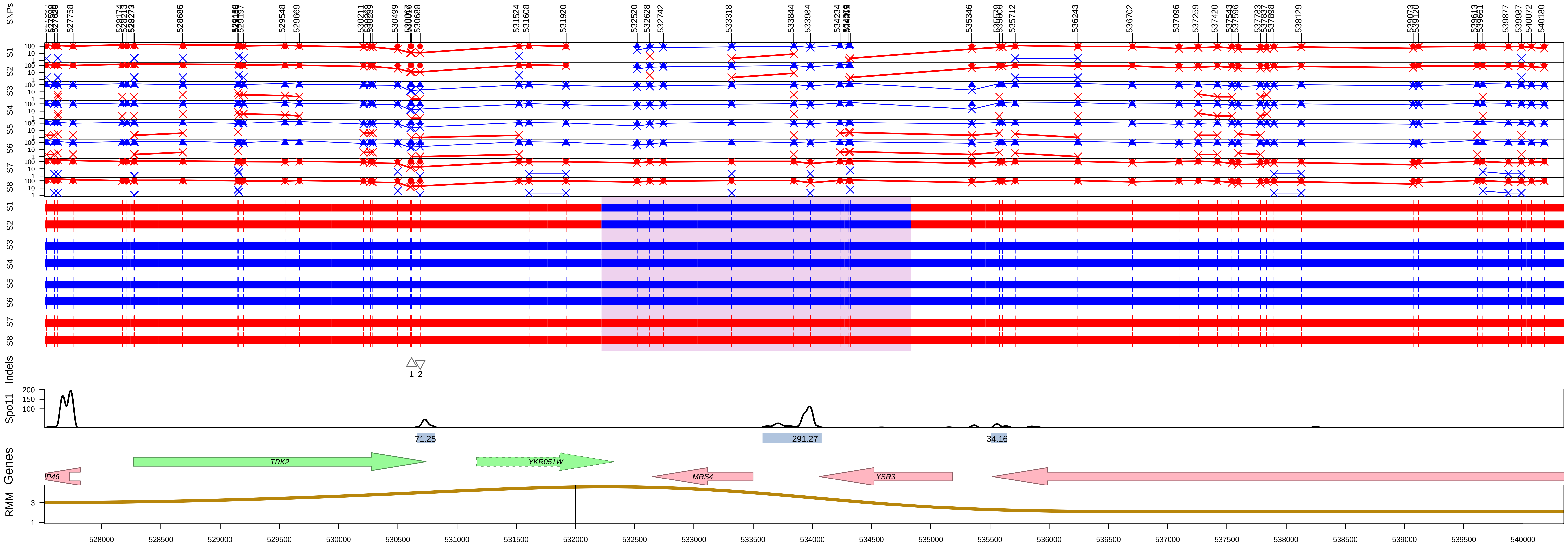

Genotype=TCMN6 Id=72 Chromo=11 Type=Full Conversion
Estimated: DSBs=1, COs=0, NCOs=1
Pattern=2:6
Mid Len=2612bp Midpoint=533526.25 Variants=9
Red=S288c, Blue=SK1

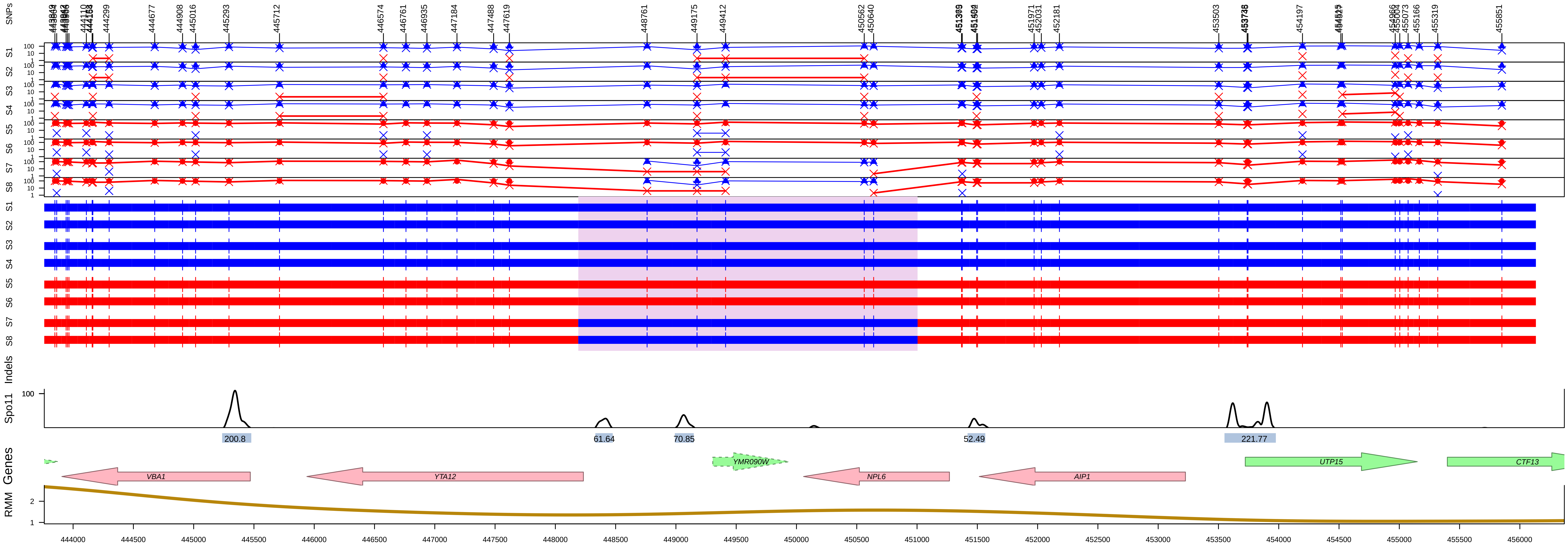

Genotype=TCMN6 Id=88 Chromo=13 Type=Full Conversion
Estimated: DSBs=1, COs=0, NCOs=1
Pattern=2:6
Mid Len=2814bp Midpoint=449597.25 Variants=5
Red=S288c, Blue=SK1

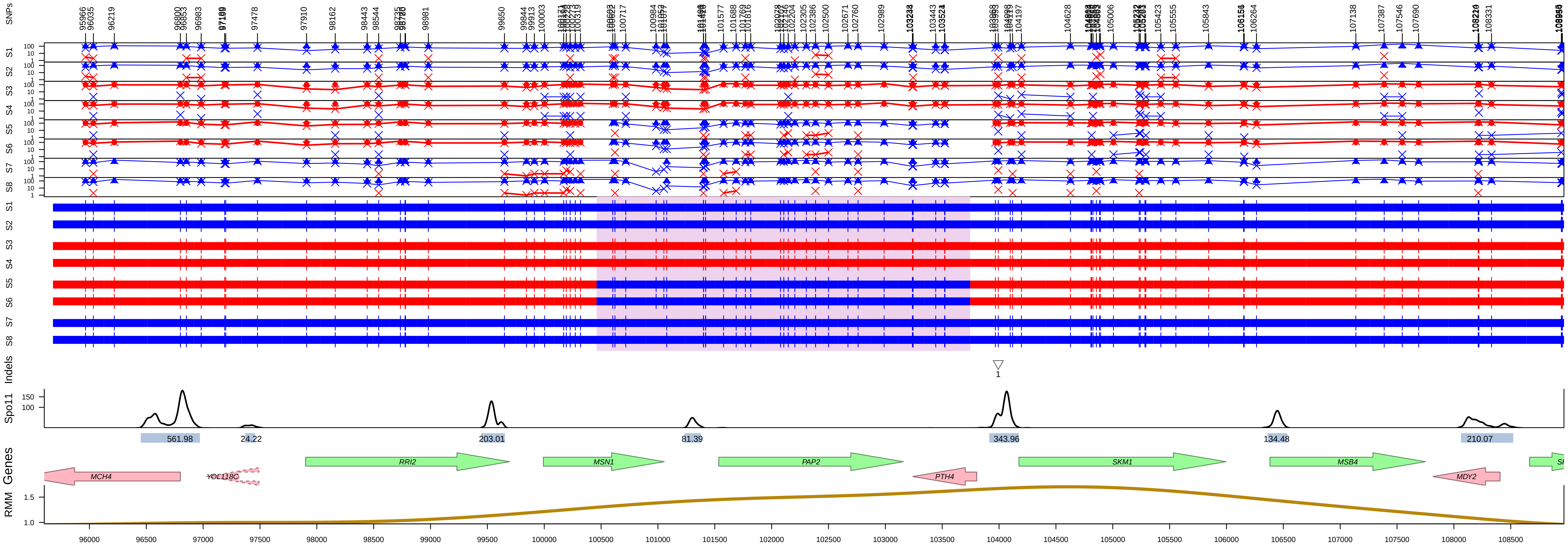

Genotype=TCMN6 Id=108 Chromo=15 Type=Full Conversion
Estimated: DSBs=1, COs=0, NCOs=1
Pattern=2:6
Mid Len=3285bp Midpoint=102103.5 Variants=26
Red=S288c, Blue=SK1

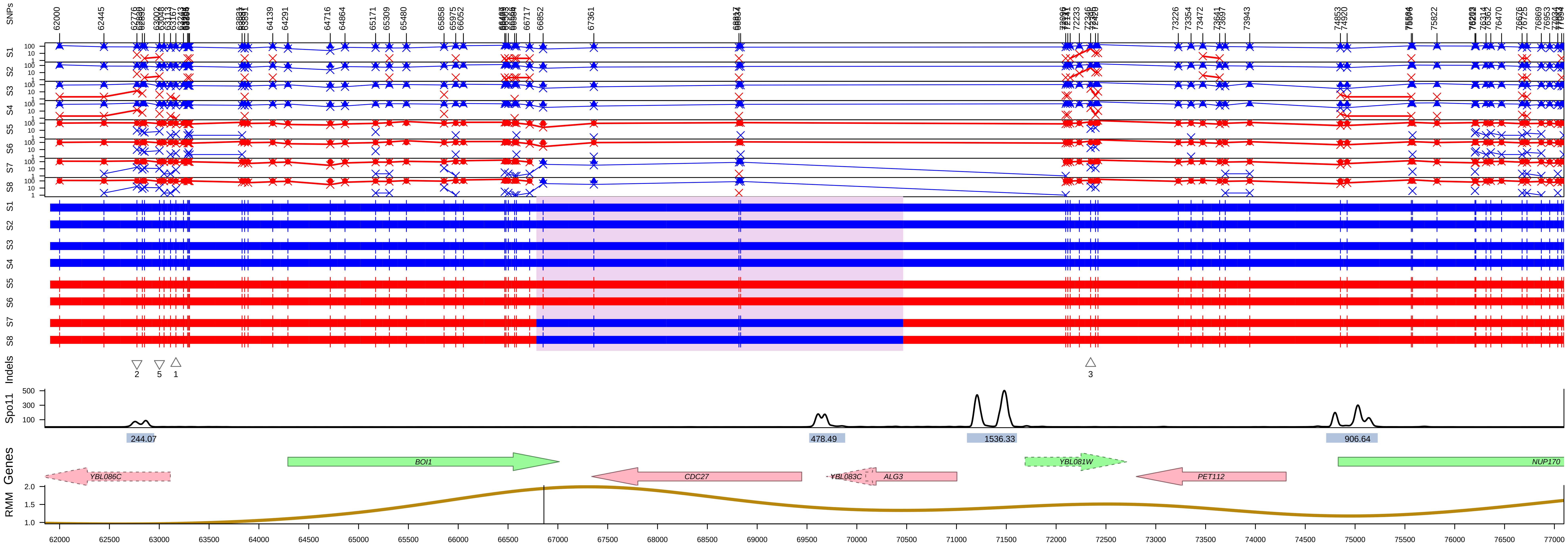

Genotype=TCMN6 Id=4 Chromo=2 Type=Full Conversion
Estimated: DSBs=1, COs=0, NCOs=1
Pattern=2:6
Mid Len=3680bp Midpoint=68624.75 Variants=4
Red=S288c, Blue=SK1

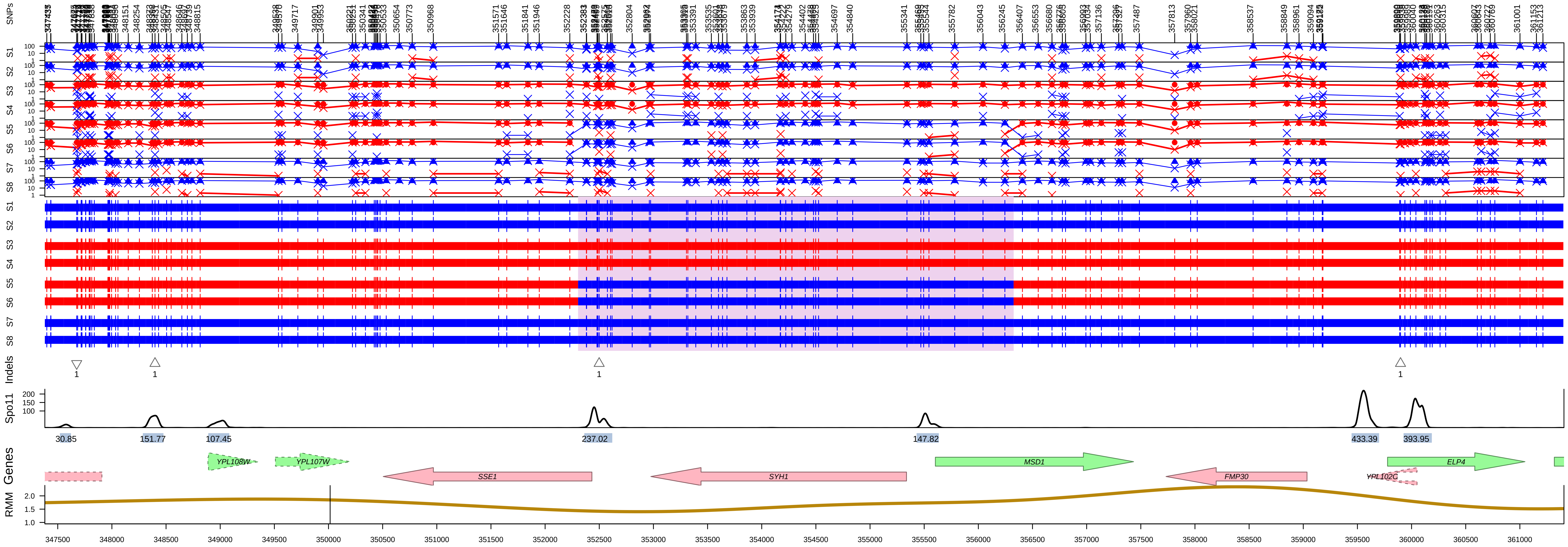

Genotype=TCMN6 Id=126 Chromo=16 Type=Full Conversion
Estimated: DSBs=1, COs=0, NCOs=1
Pattern=2:6
Mid Len=4022bp Midpoint=354315.25 Variants=37
Red=S288c, Blue=SK1

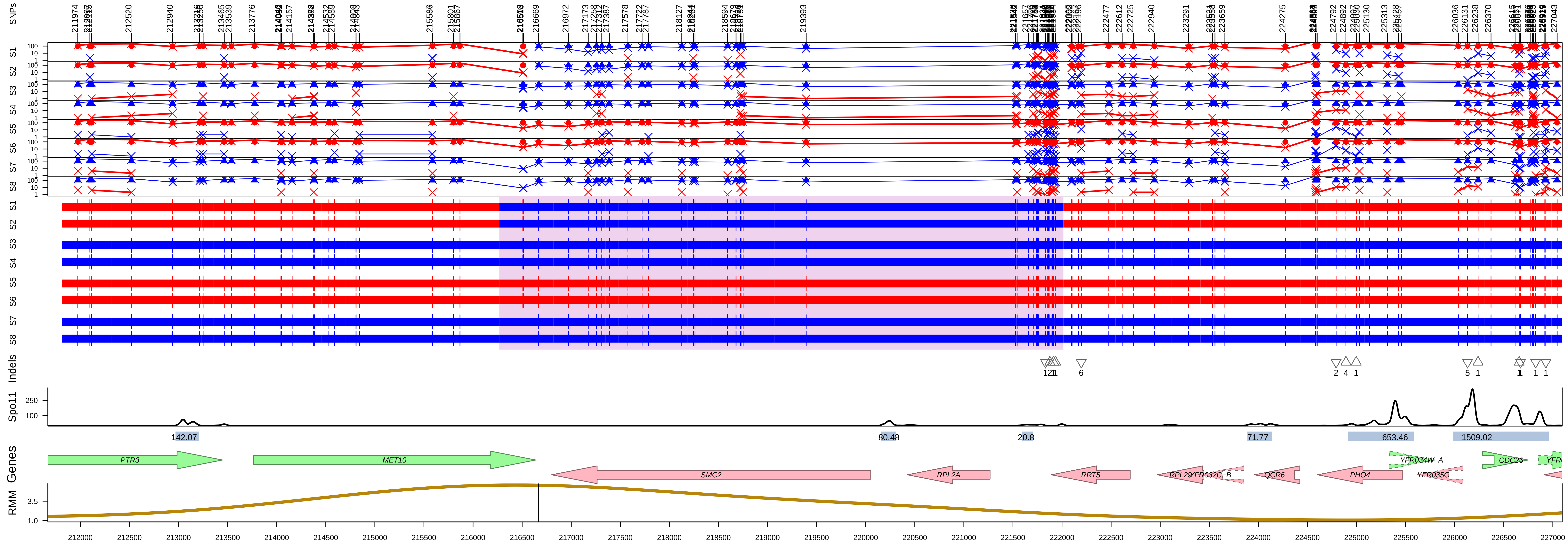

Genotype=TCMN6 Id=33 Chromo=6 Type=Full Conversion
Estimated: DSBs=1, COs=0, NCOs=1
Pattern=2:6
Mid Len=5746bp Midpoint=219141.25 Variants=36
Red=S288c, Blue=SK1

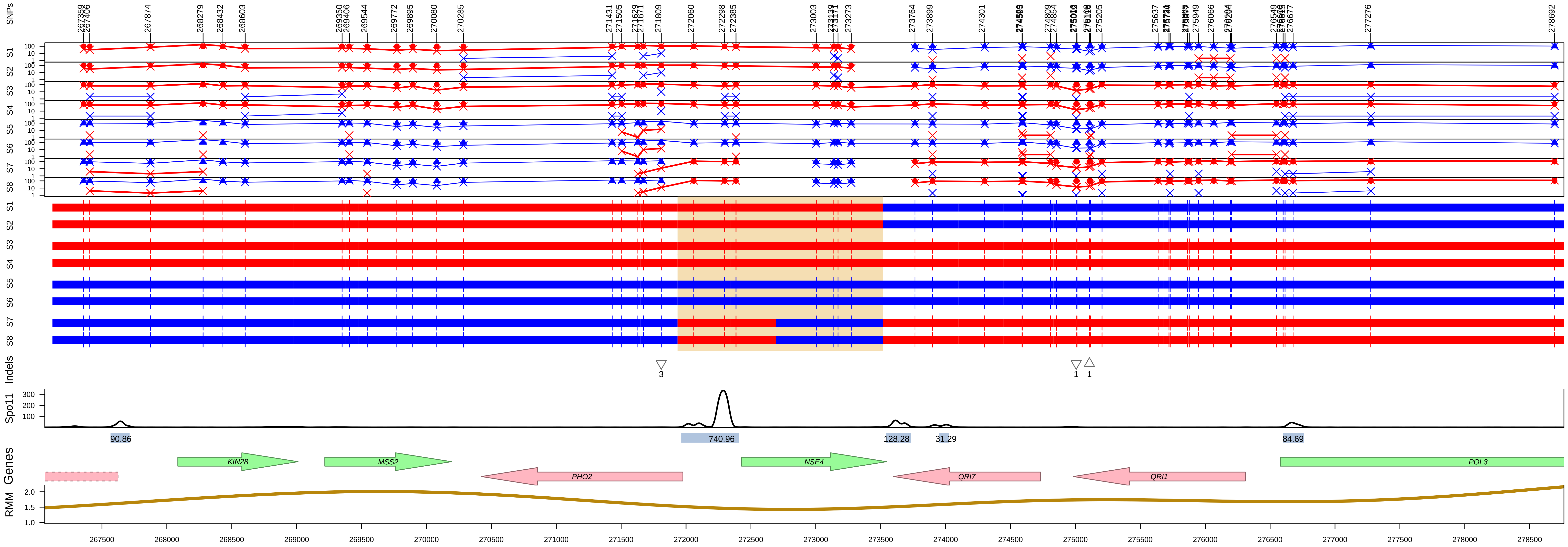

Genotype=TCMN6 Id=21 Chromo=4 Type=One−Sided Unidirectional
Estimated: DSBs=1, COs=1, NCOs=0
Pattern=(6:2)_(4:4)_(4:4aCO)
Mid Len=1584bp Midpoint=272726.5 Variants=7
Red=S288c, Blue=SK1

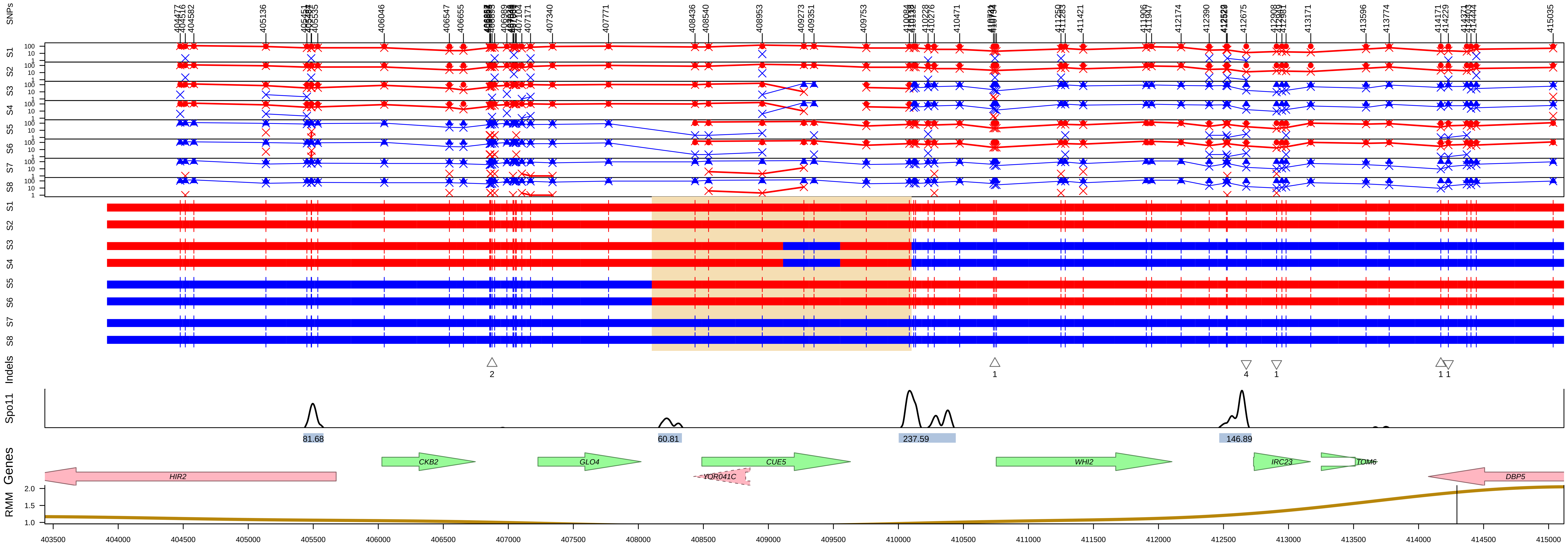

Genotype=TCMN6 Id=114 Chromo=15 Type=One−Sided Unidirectional
Estimated: DSBs=1, COs=1, NCOs=0
Pattern=(6:2)_(4:4aCO)_(6:2)_(4:4a)
Mid Len=1998bp Midpoint=409102.25 Variants=7
Red=S288c, Blue=SK1

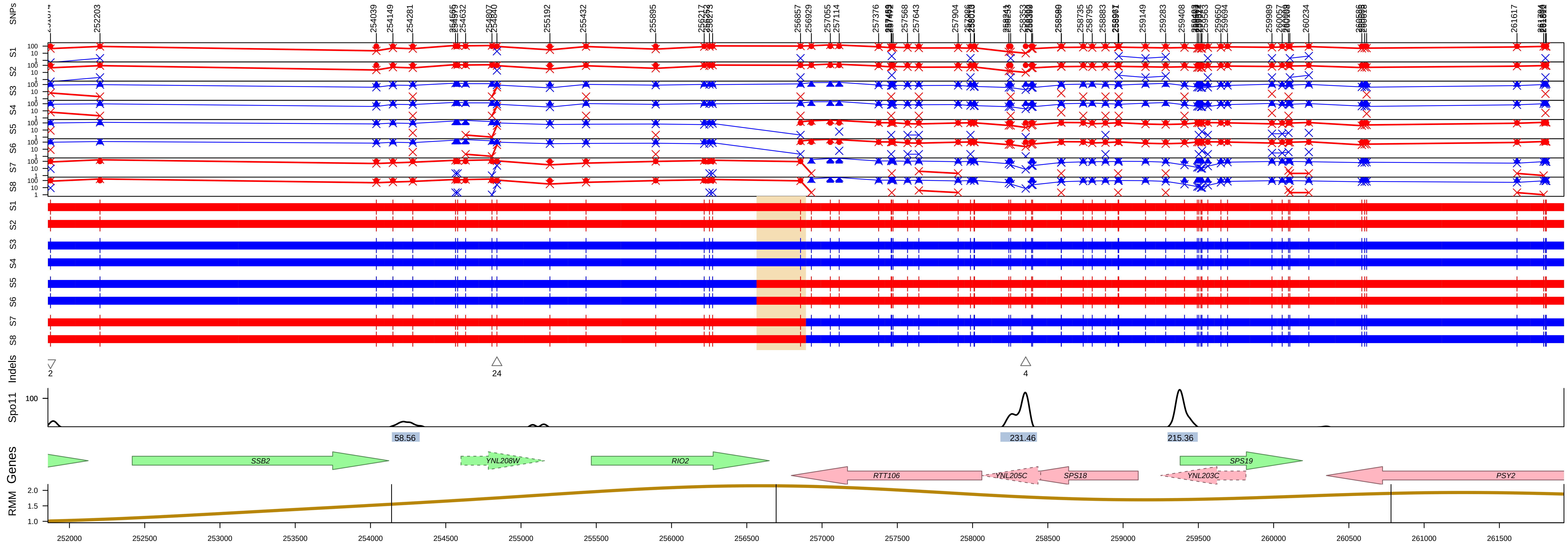
Genotype=TCMN6 Id=100 Chromo=14 Type=Full Conversion
Estimated: DSBs=1, COs=1, NCOs=0
Pattern=(6:2)_(4:4aCO)
Mid Len=328bp Midpoint=256729 Variants=1
Red=S288c, Blue=SK1

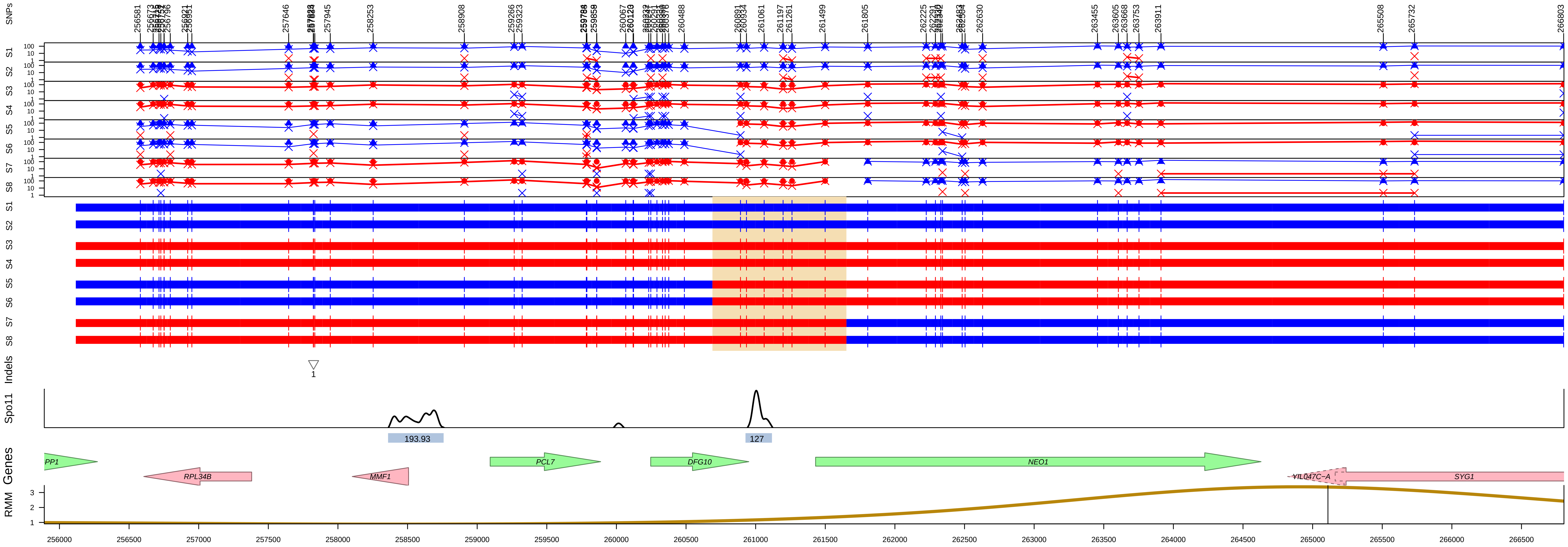

Genotype=TCMN6 Id=58 Chromo=9 Type=Full Conversion
Estimated: DSBs=1, COs=1, NCOs=0
Pattern=(6:2)_(4:4aCO)
Mid Len=962bp Midpoint=261170.75 Variants=6
Red=S288c, Blue=SK1

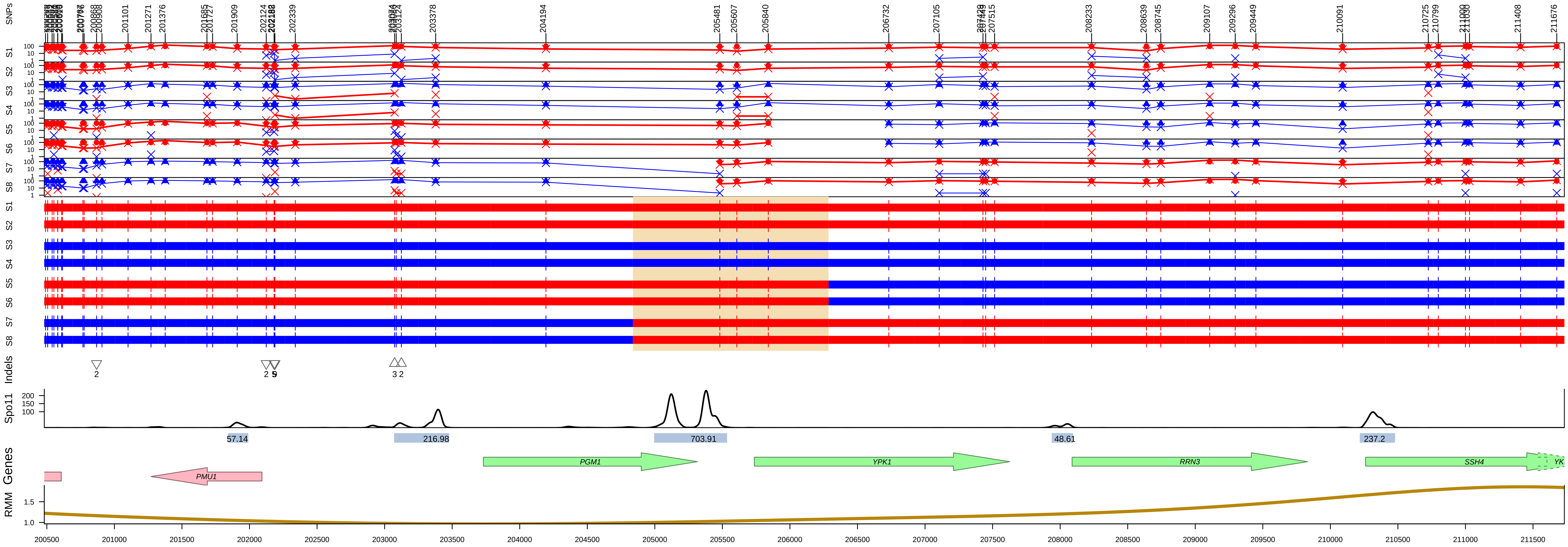

Genotype=TCMN6 Id=70 Chromo=11 Type=Full Conversion
Estimated: DSBs=1, COs=1, NCOs=0
Pattern=(6:2)_(4:4aCO)
Mid Len=1448bp Midpoint=205561.75 Variants=3
Red=S288c, Blue=SK1

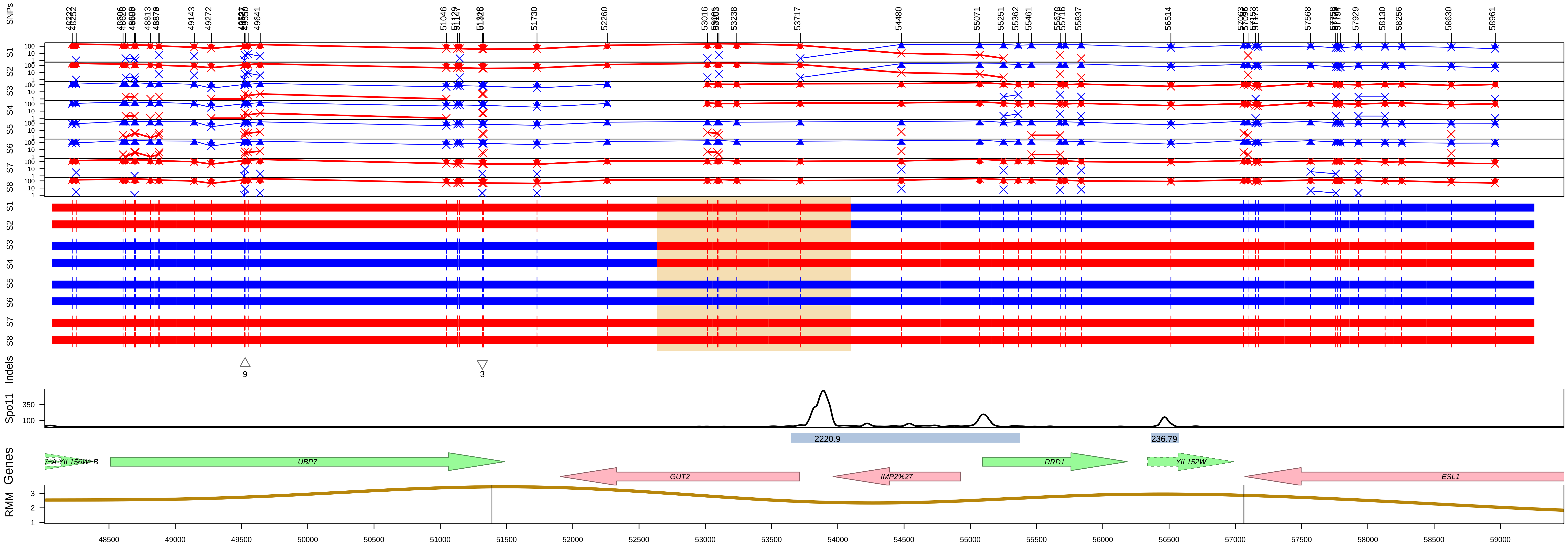

Genotype=TCMN6 Id=55 Chromo=9 Type=Full Conversion
Estimated: DSBs=1, COs=1, NCOs=0
Pattern=(6:2)_(4:4aCO)
Mid Len=1460bp Midpoint=53368.25 Variants=5
Red=S288c, Blue=SK1

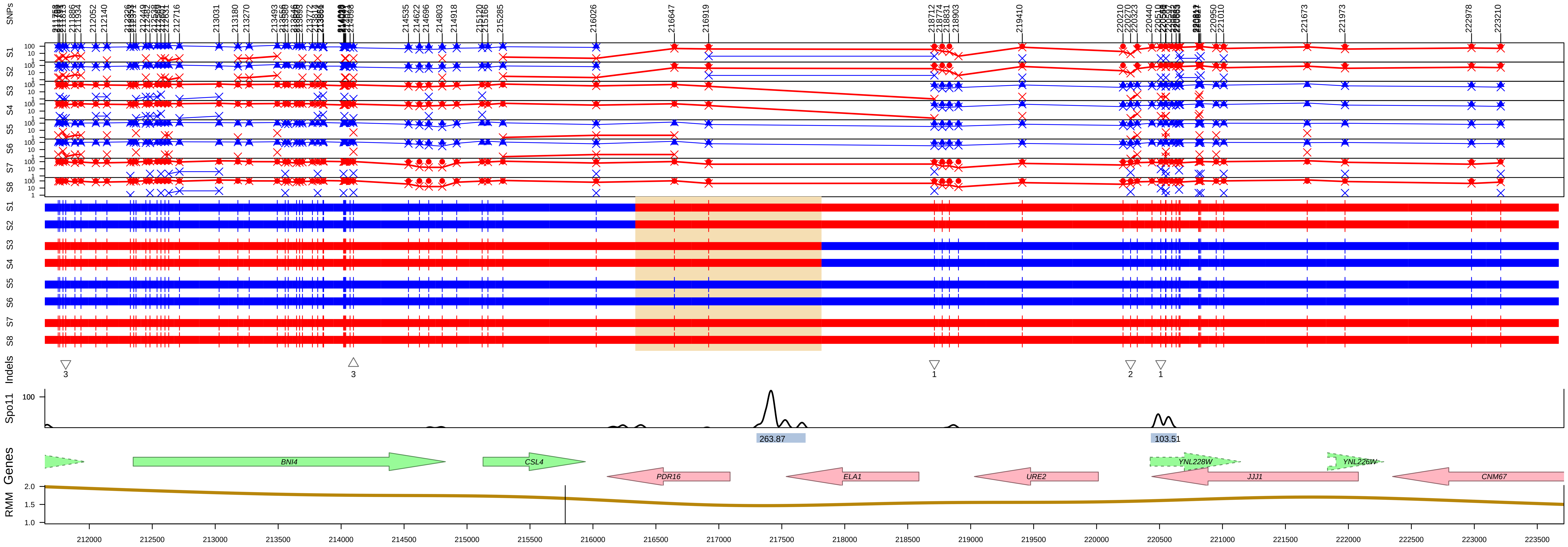

Genotype=TCMN6 Id=99 Chromo=14 Type=Full Conversion
Estimated: DSBs=1, COs=1, NCOs=0
Pattern=(6:2)_(4:4aCO)
Mid Len=1479bp Midpoint=217076 Variants=2
Red=S288c, Blue=SK1

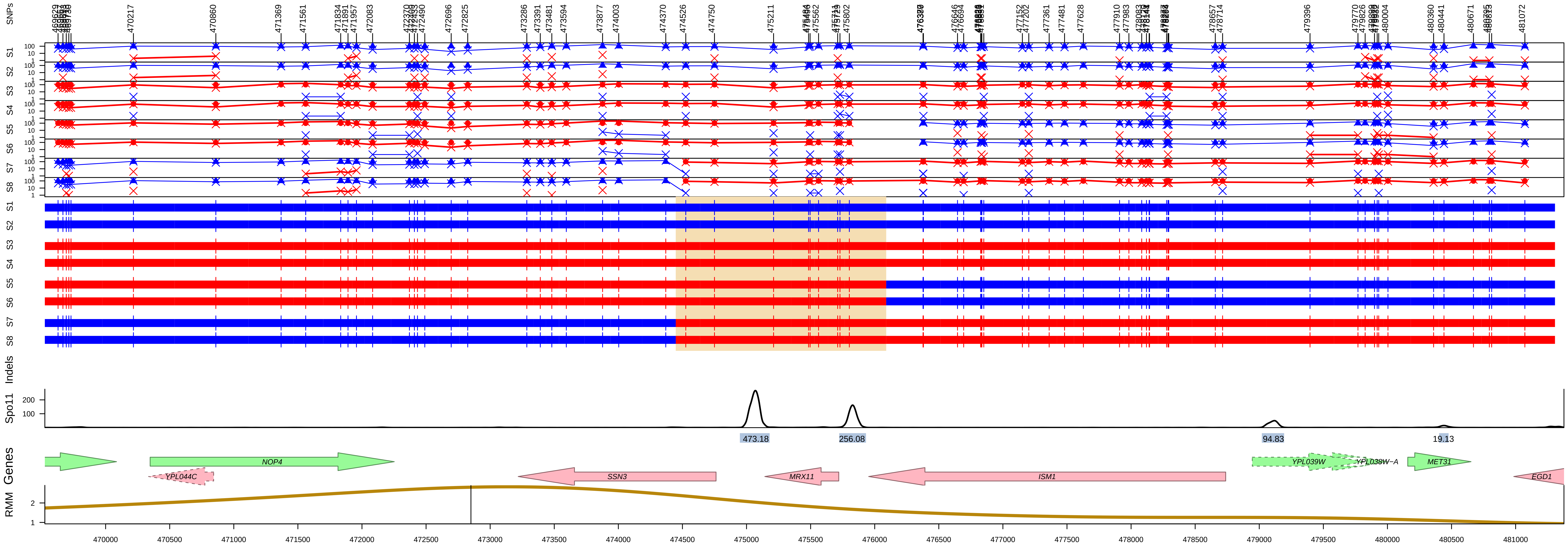

Genotype=TCMN6 Id=127 Chromo=16 Type=Full Conversion
Estimated: DSBs=1, COs=1, NCOs=0
Pattern=(6:2)_(4:4aCO)
Mid Len=1642bp Midpoint=475268.75 Variants=9
Red=S288c, Blue=SK1

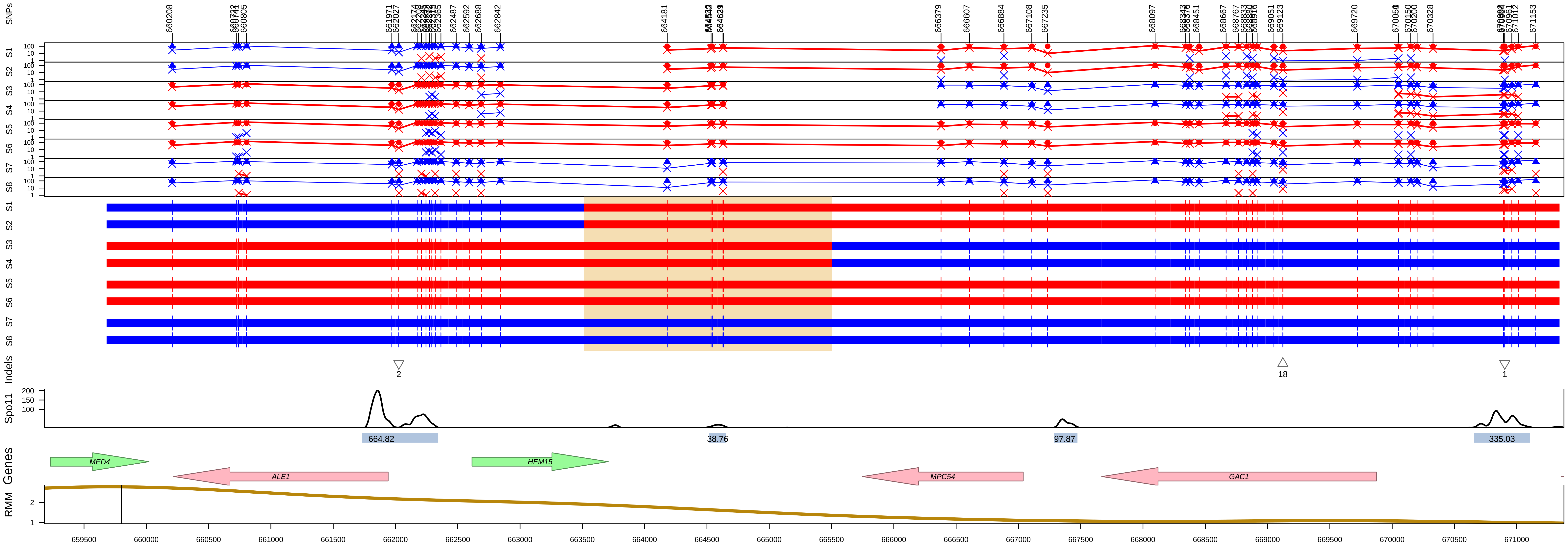

Genotype=TCMN6 Id=119 Chromo=15 Type=Full Conversion

Estimated: DSBs=1, COs=1, NCOs=0

Pattern=(6:2)_(4:4aCO)

Mid Len=1994bp Midpoint=664508.25 Variants=5

Red=S288c, Blue=SK1

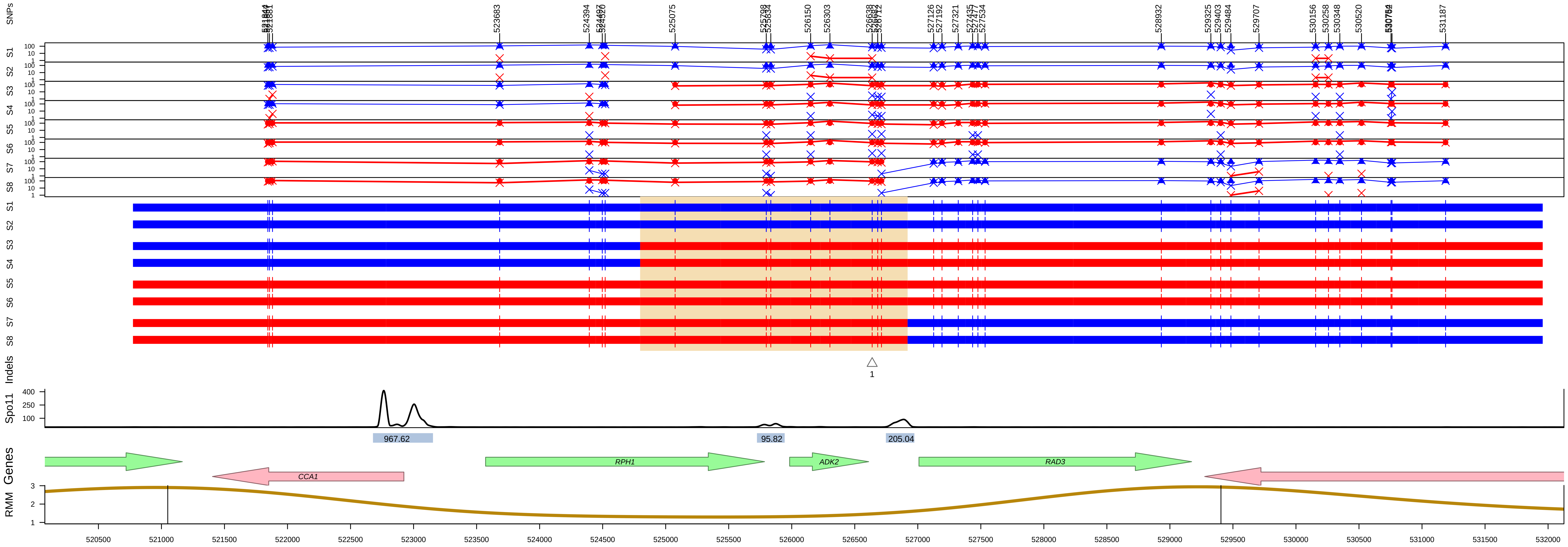

Genotype=TCMN6 Id=30 Chromo=5 Type=Full Conversion
Estimated: DSBs=1, COs=1, NCOs=0
Pattern=(6:2)_(4:4aCO)
Mid Len=2122bp Midpoint=525858.25 Variants=8
Red=S288c, Blue=SK1

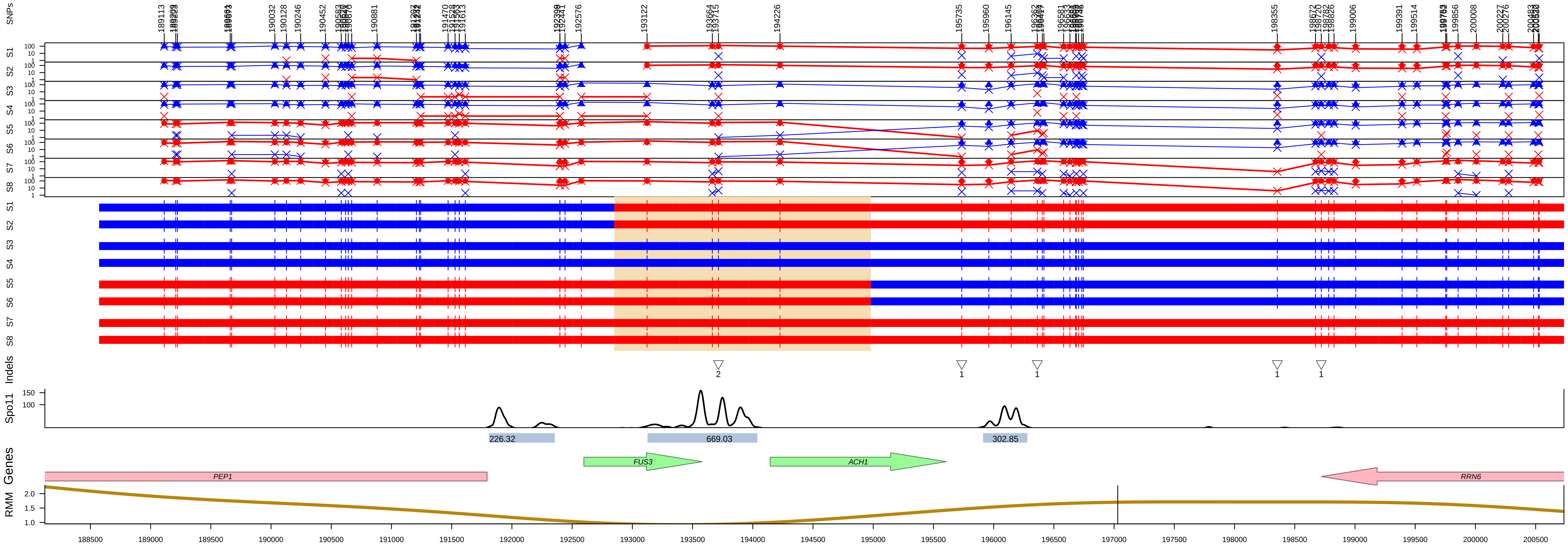

Genotype=TCMN6 Id=9 Chromo=2 Type=Full Conversion
Estimated: DSBs=1, COs=1, NCOs=0
Pattern=(6:2)_(4:4aCO)
Mid Len=2132bp Midpoint=193914.75 Variants=4
Red=S288c, Blue=SK1

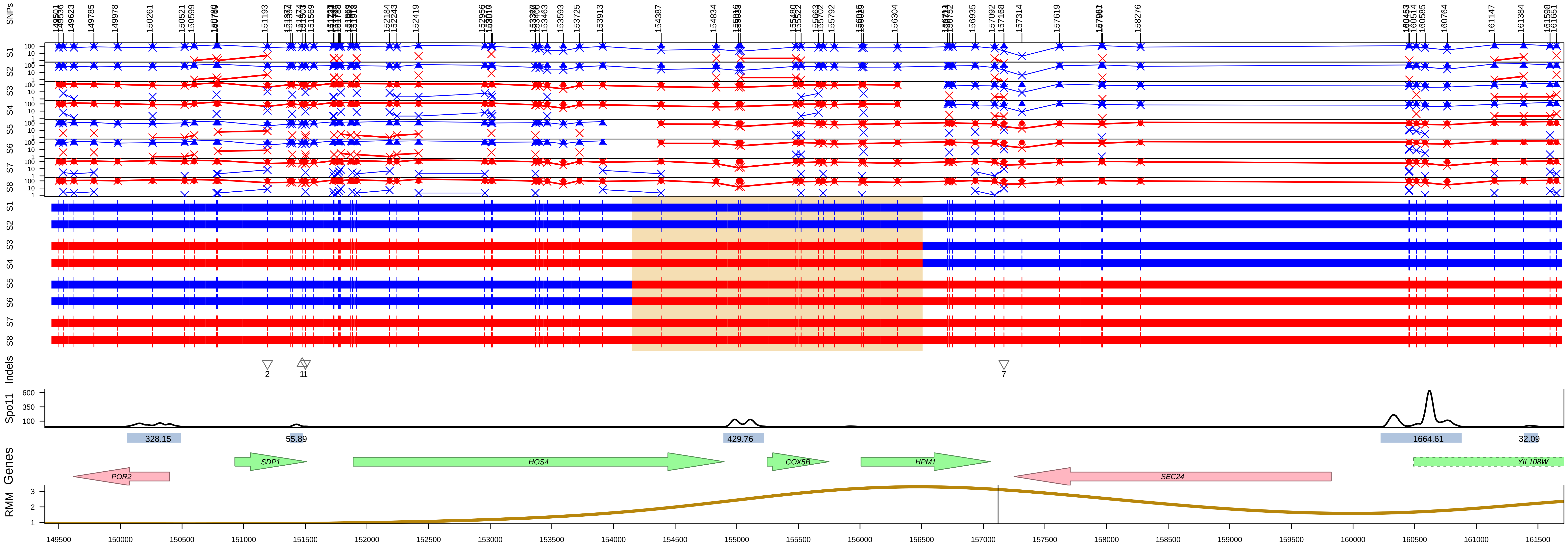

Genotype=TCMN6 Id=56 Chromo=9 Type=Full Conversion
Estimated: DSBs=1, COs=1, NCOs=0
Pattern=(6:2)_(4:4aCO)
Mid Len=2358bp Midpoint=155328.75 Variants=12
Red=S288c, Blue=SK1

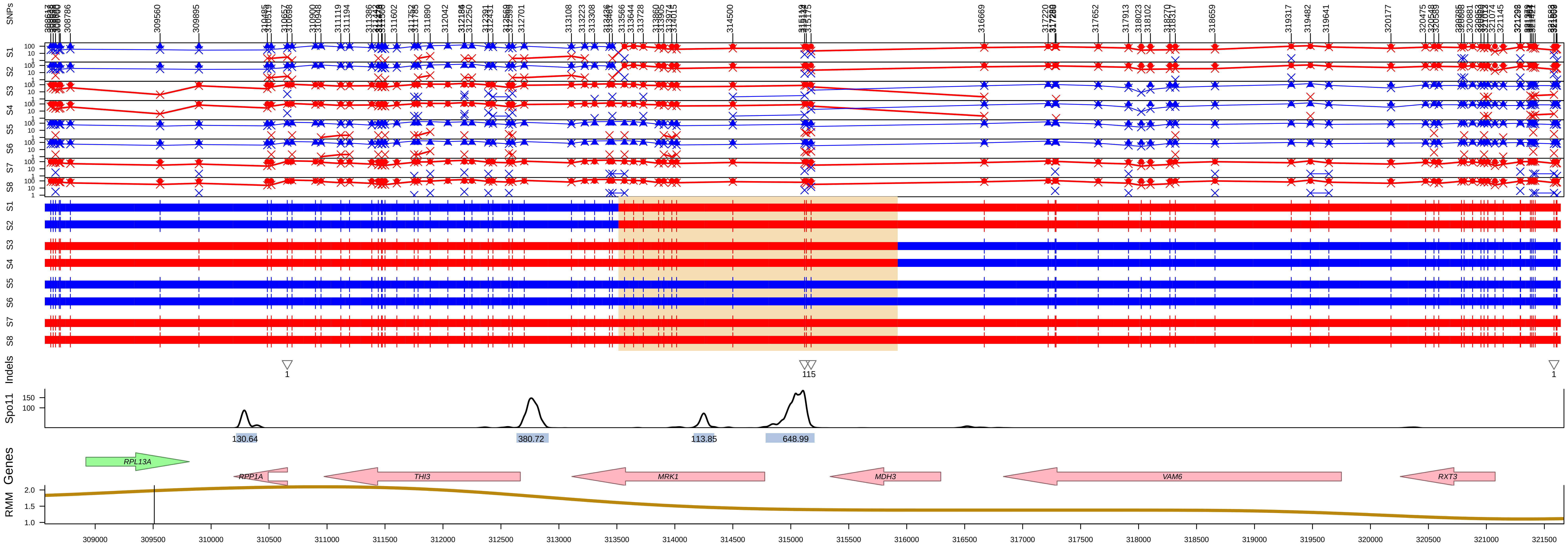

Genotype=TCMN6 Id=22 Chromo=4 Type=Full Conversion
Estimated: DSBs=1, COs=1, NCOs=0
Pattern=(6:2)_(4:4aCO)
Mid Len=2408bp Midpoint=314717.75 Variants=11
Red=S288c, Blue=SK1

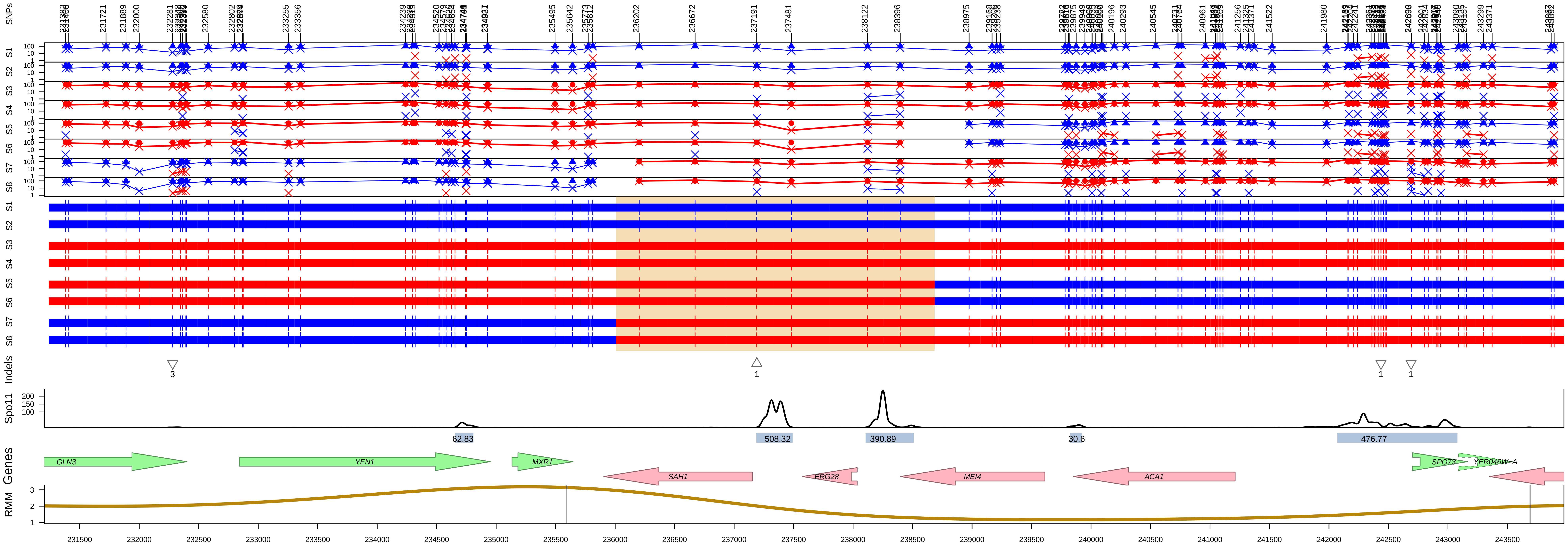

Genotype=TCMN6 Id=25 Chromo=5 Type=Full Conversion
Estimated: DSBs=1, COs=1, NCOs=0
Pattern=(6:2)_(4:4aCO)
Mid Len=2678bp Midpoint=237346.25 Variants=6
Red=S288c, Blue=SK1

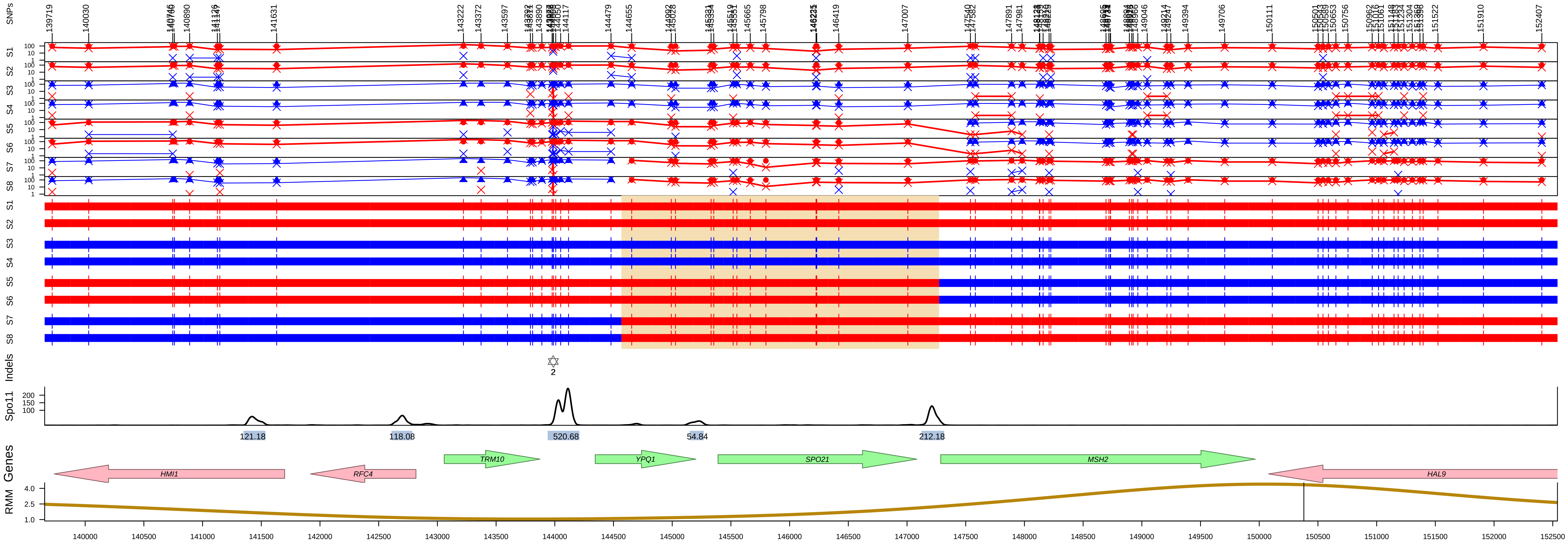

Genotype=TCMN6 Id=110 Chromo=15 Type=Full Conversion
Estimated: DSBs=1, COs=1, NCOs=0
Pattern=(6:2)_(4:4aCO)
Mid Len=2706bp Midpoint=145920.25 Variants=13
Red=S288c, Blue=SK1

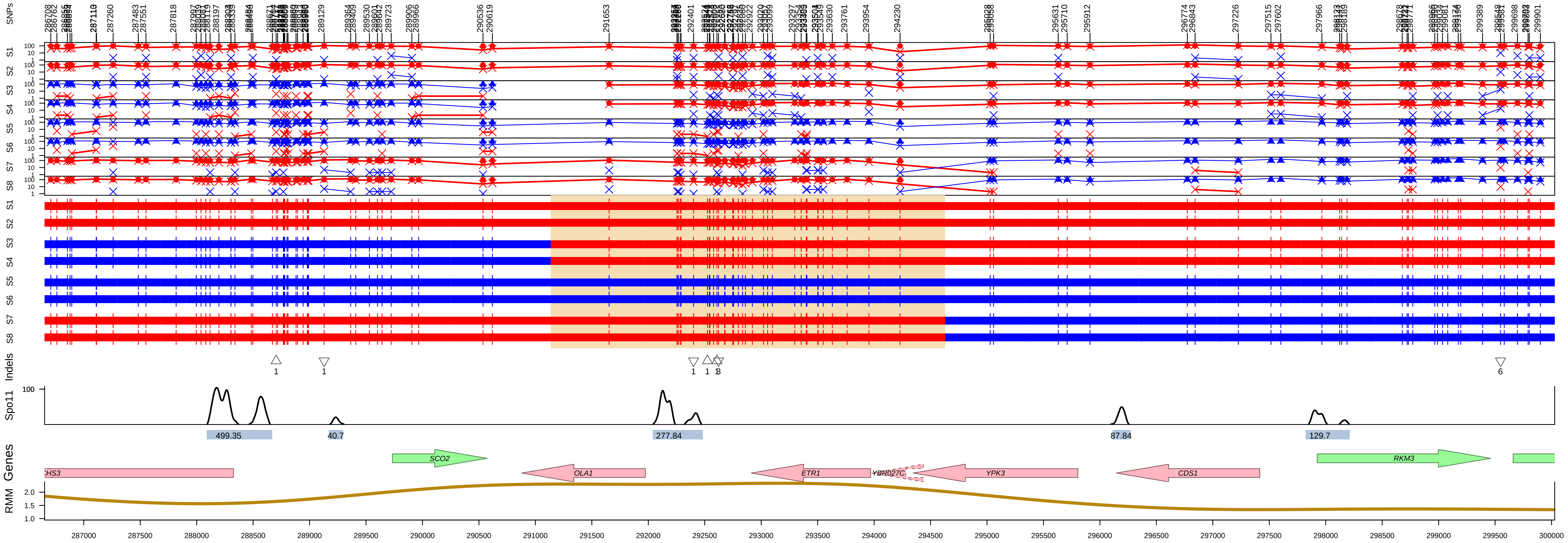

Genotype=TCMN6 Id=10 Chromo=2 Type=Full Conversion
Estimated: DSBs=1, COs=1, NCOs=0
Pattern=(6:2)_(4:4aCO)
Mid Len=3493bp Midpoint=292882.5 Variants=36
Red=S288c, Blue=SK1

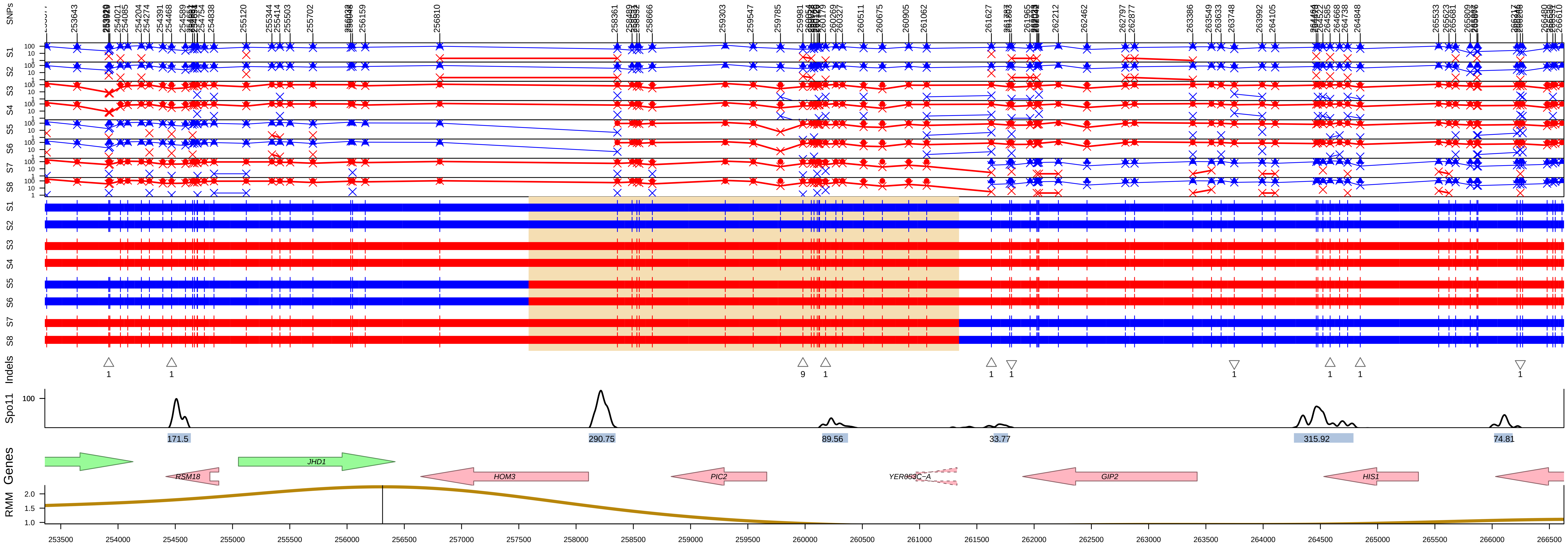

Genotype=TCMN6 Id=26 Chromo=5 Type=Full Conversion

Estimated: DSBs=1, COs=1, NCOs=0

Pattern=(6:2)_(4:4aCO)

Mid Len=3759bp Midpoint=259465 Variants=20

Red=S288c, Blue=SK1

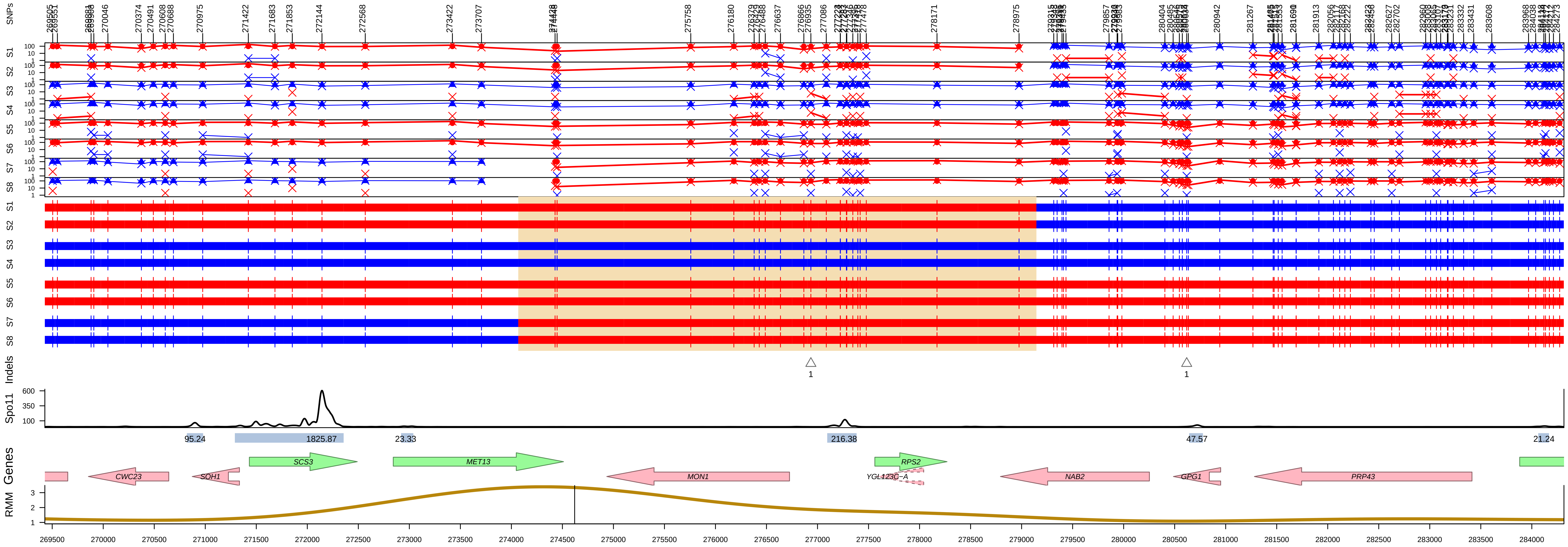

Genotype=TCMN6 Id=43 Chromo=7 Type=Full Conversion
Estimated: DSBs=1, COs=1, NCOs=0
Pattern=(6:2)_(4:4aCO)
Mid Len=5078bp Midpoint=276606.25 Variants=21
Red=S288c, Blue=SK1

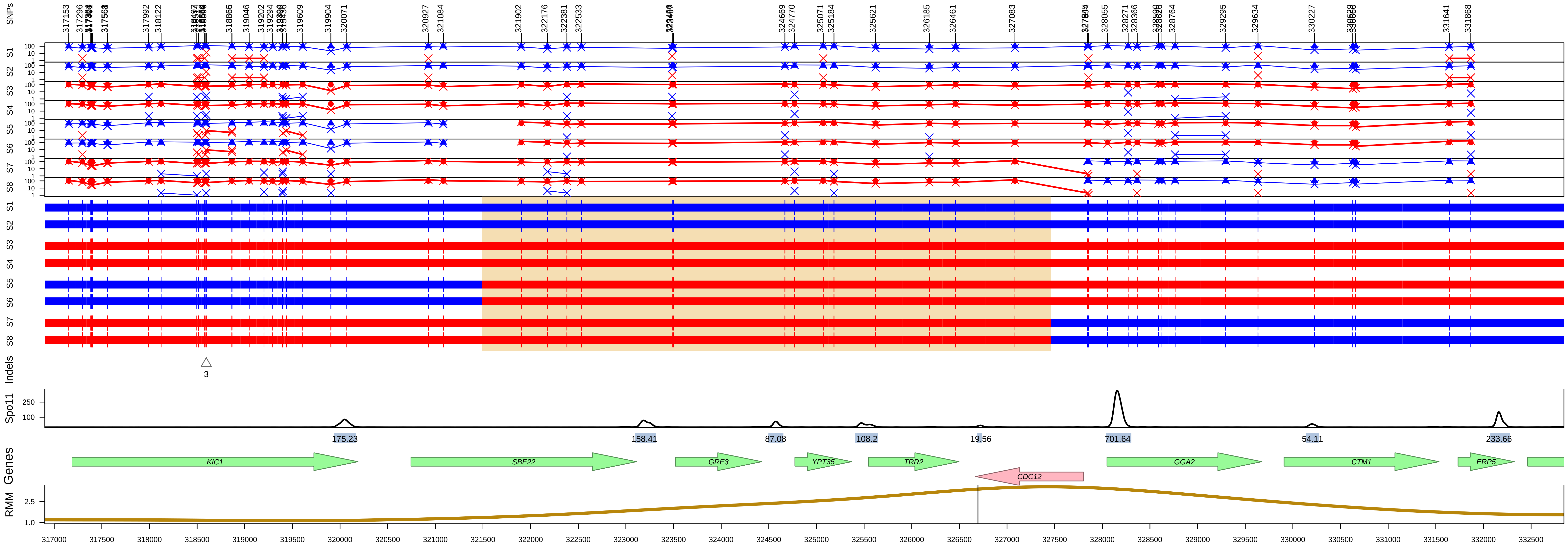

Genotype=TCMN6 Id=53 Chromo=8 Type=Full Conversion
Estimated: DSBs=1, COs=1, NCOs=0
Pattern=(6:2)_(4:4aCO)
Mid Len=5971bp Midpoint=324478.5 Variants=14
Red=S288c, Blue=SK1

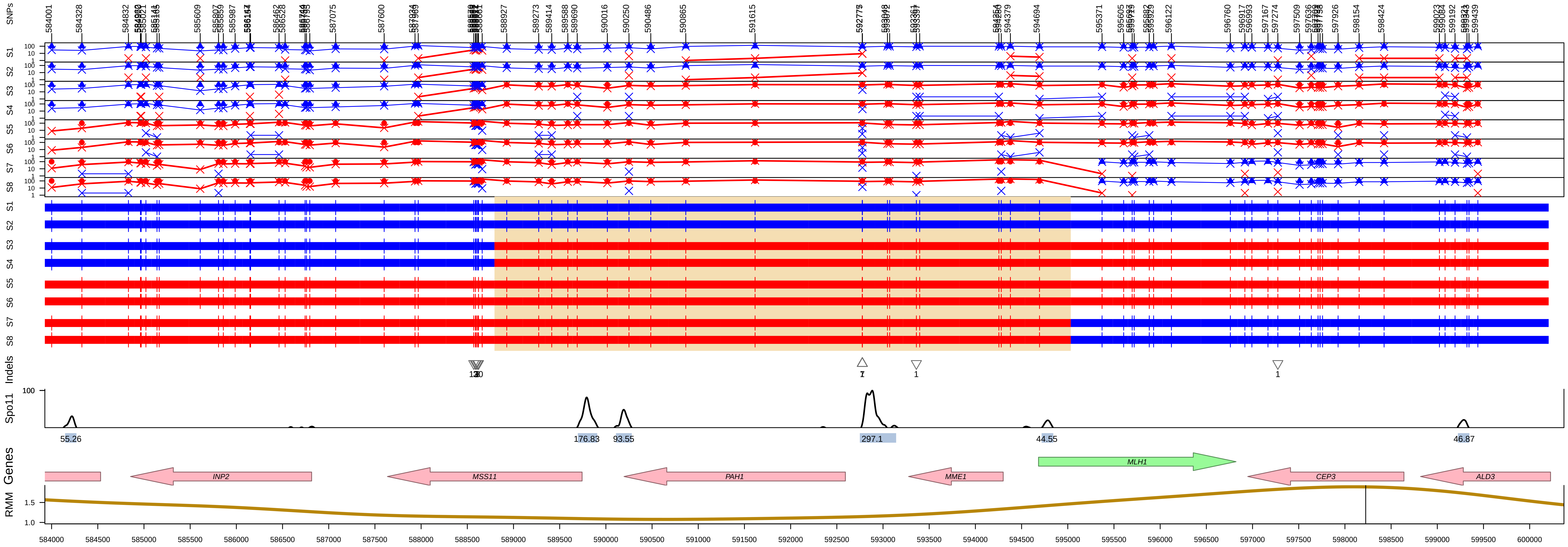

Genotype=TCMN6 Id=89 Chromo=13 Type=Full Conversion
Estimated: DSBs=1, COs=1, NCOs=0
Pattern=(6:2)_(4:4aCO)
Mid Len=6238bp Midpoint=591913.25 Variants=20
Red=S288c, Blue=SK1

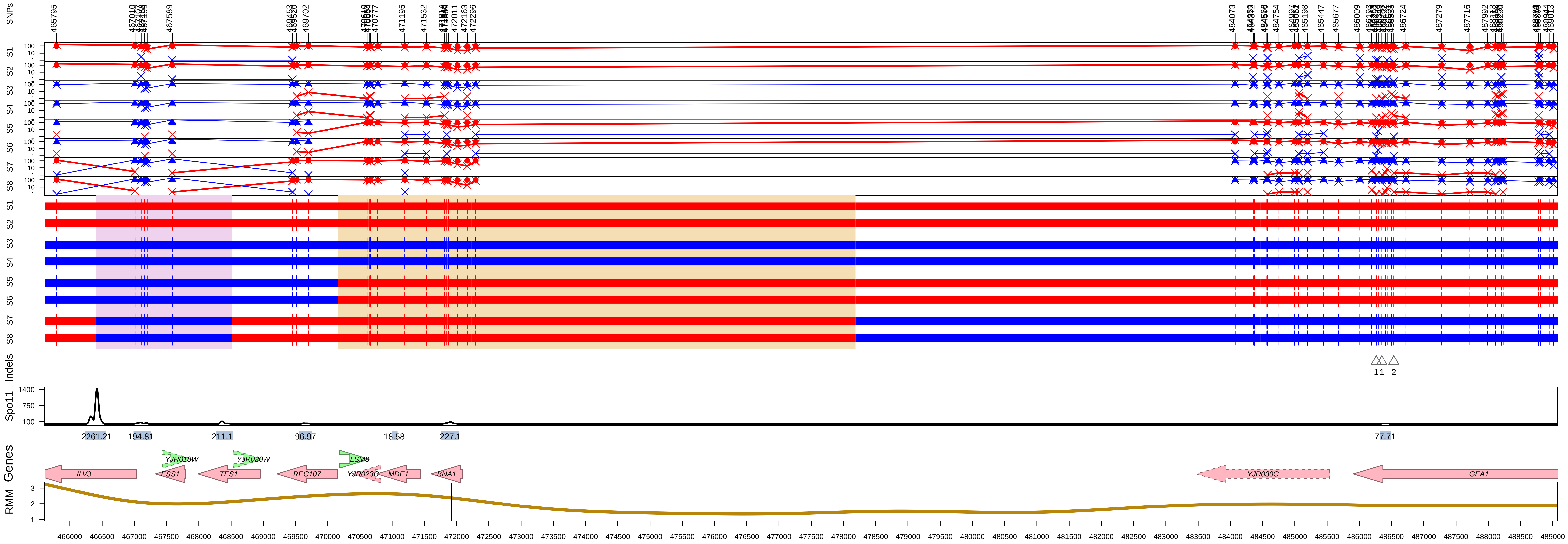

Genotype=TCMN6 Id=65 Chromo=10 Type=Full Conversion
Estimated: DSBs=1, COs=1, NCOs=0
Pattern=(6:2)_(4:4aCO)
Mid Len=8028bp Midpoint=474170.25 Variants=12
Red=S288c, Blue=SK1

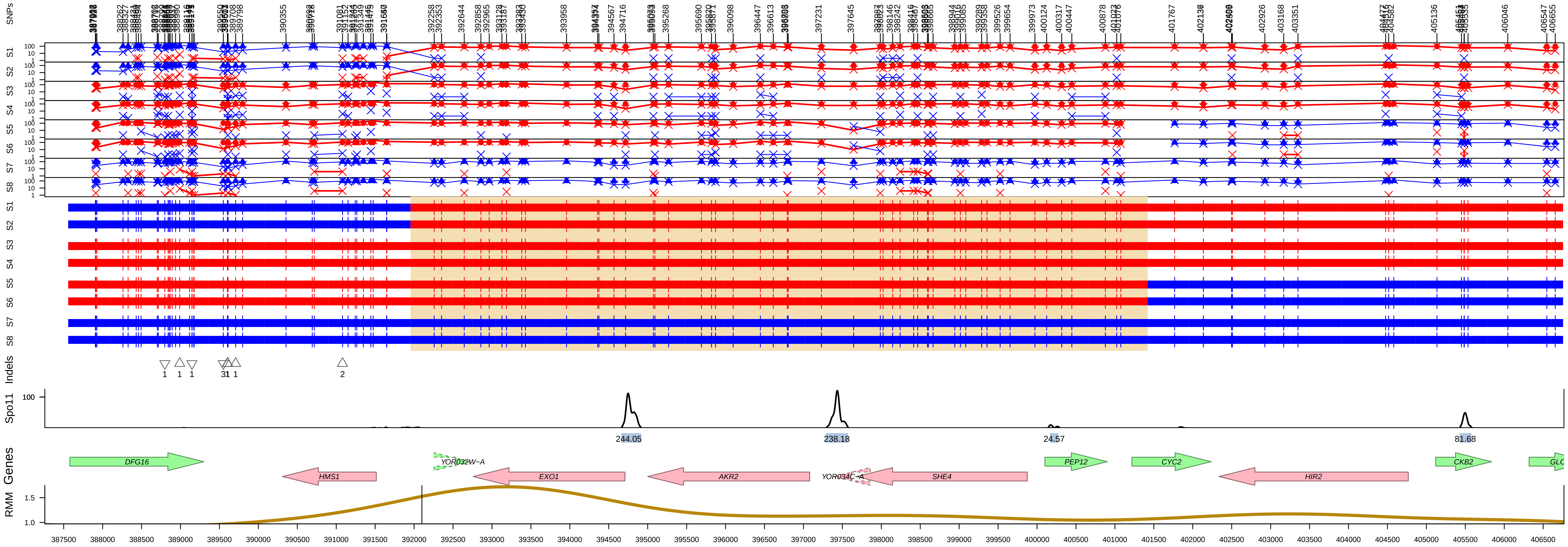

Genotype=TCMN6 Id=113 Chromo=15 Type=Full Conversion
Estimated: DSBs=1, COs=1, NCOs=0
Pattern=(6:2)_(4:4aCO)
Mid Len=9468bp Midpoint=396687.75 Variants=49
Red=S288c, Blue=SK1

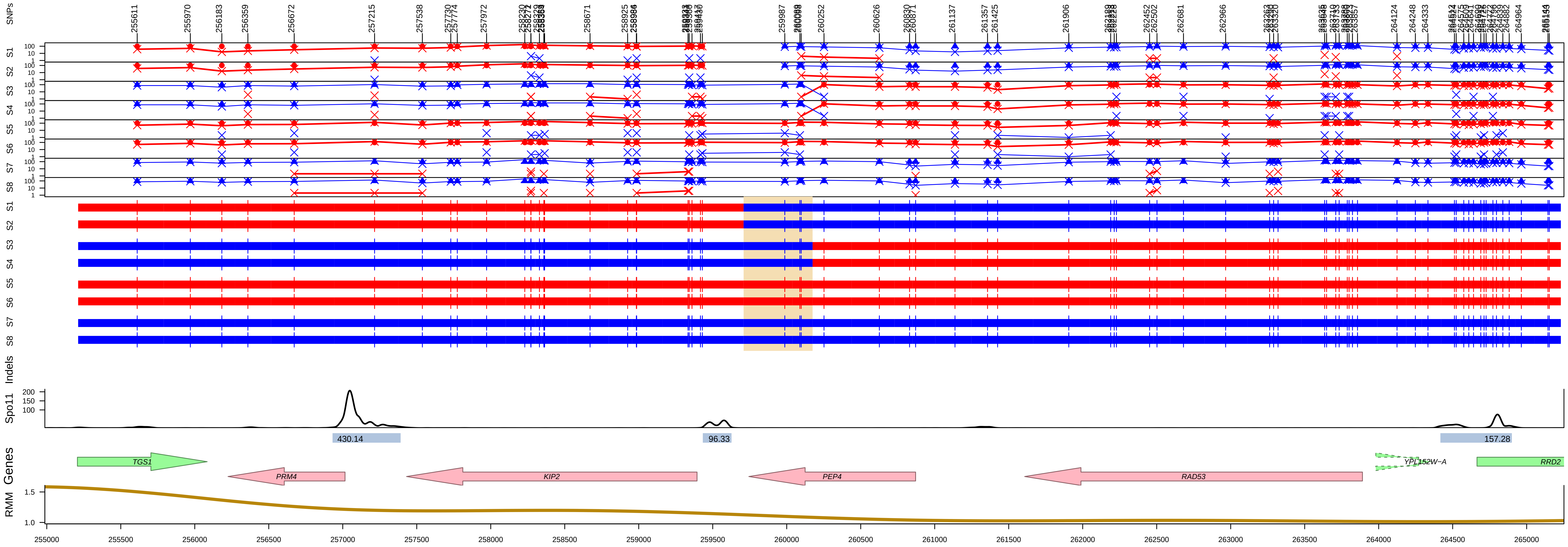

Genotype=TCMN6 Id=125 Chromo=16 Type=Full Conversion
Estimated: DSBs=1, COs=1, NCOs=0
Pattern=(2:6)_(4:4aCO)
Mid Len=466bp Midpoint=259941.75 Variants=3
Red=S288c, Blue=SK1

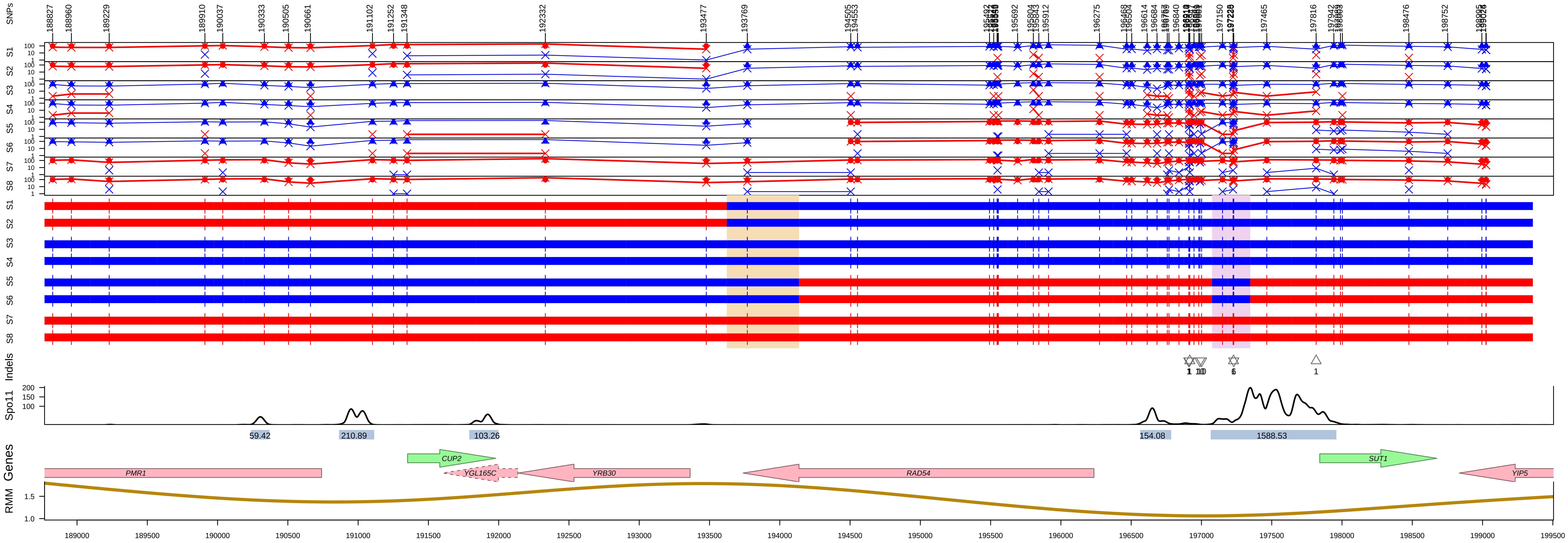

Genotype=TCMN6 Id=39 Chromo=7 Type=Full Conversion
Estimated: DSBs=1, COs=1, NCOs=0
Pattern=(2:6)_(4:4aCO)
Mid Len=514bp Midpoint=193880 Variants=1
Red=S288c, Blue=SK1

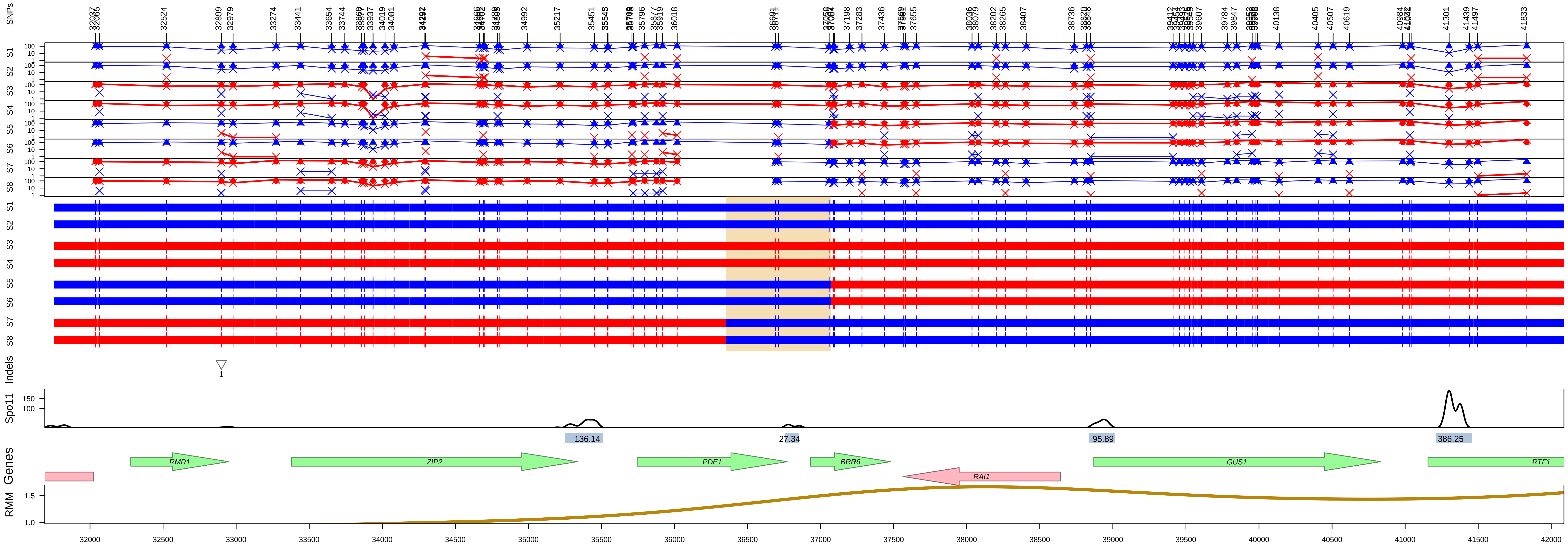

Genotype=TCMN6 Id=34 Chromo=7 Type=Full Conversion
Estimated: DSBs=1, COs=1, NCOs=0
Pattern=(2:6)_(4:4aCO)
Mid Len=718bp Midpoint=36713.5 Variants=3
Red=S288c, Blue=SK1

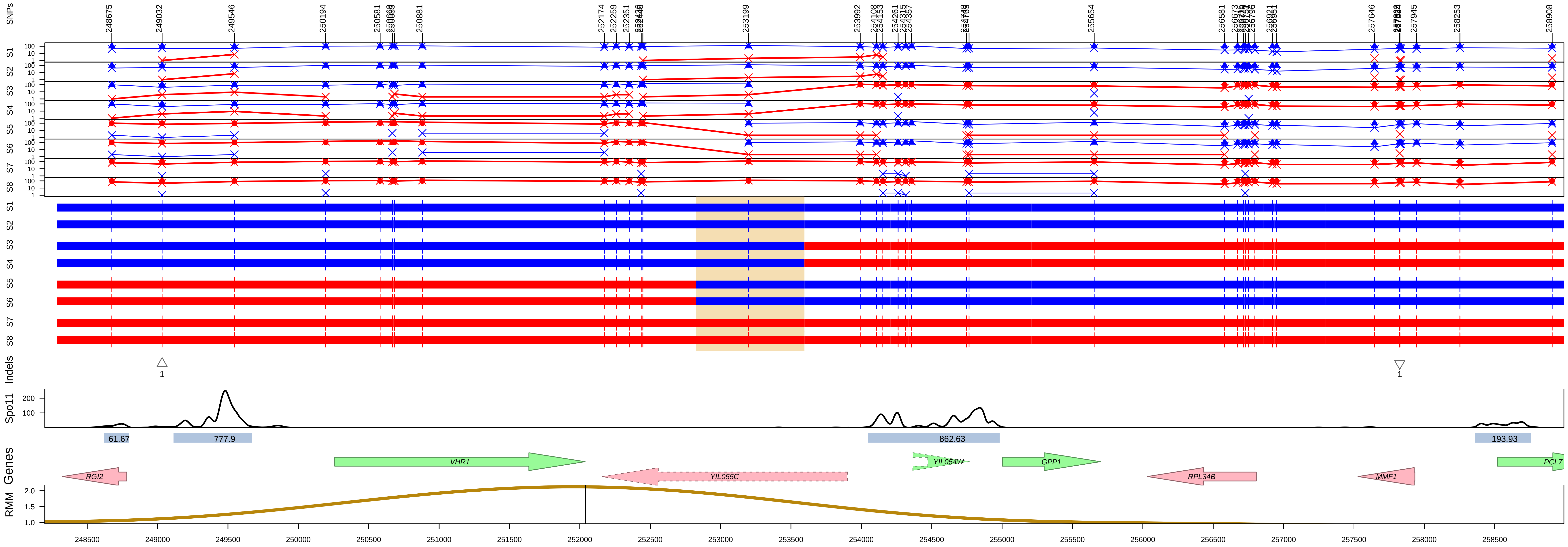

Genotype=TCMN6 Id=57 Chromo=9 Type=Full Conversion
Estimated: DSBs=1, COs=1, NCOs=0
Pattern=(2:6)_(4:4aCO)
Mid Len=772bp Midpoint=253209.5 Variants=1
Red=S288c, Blue=SK1

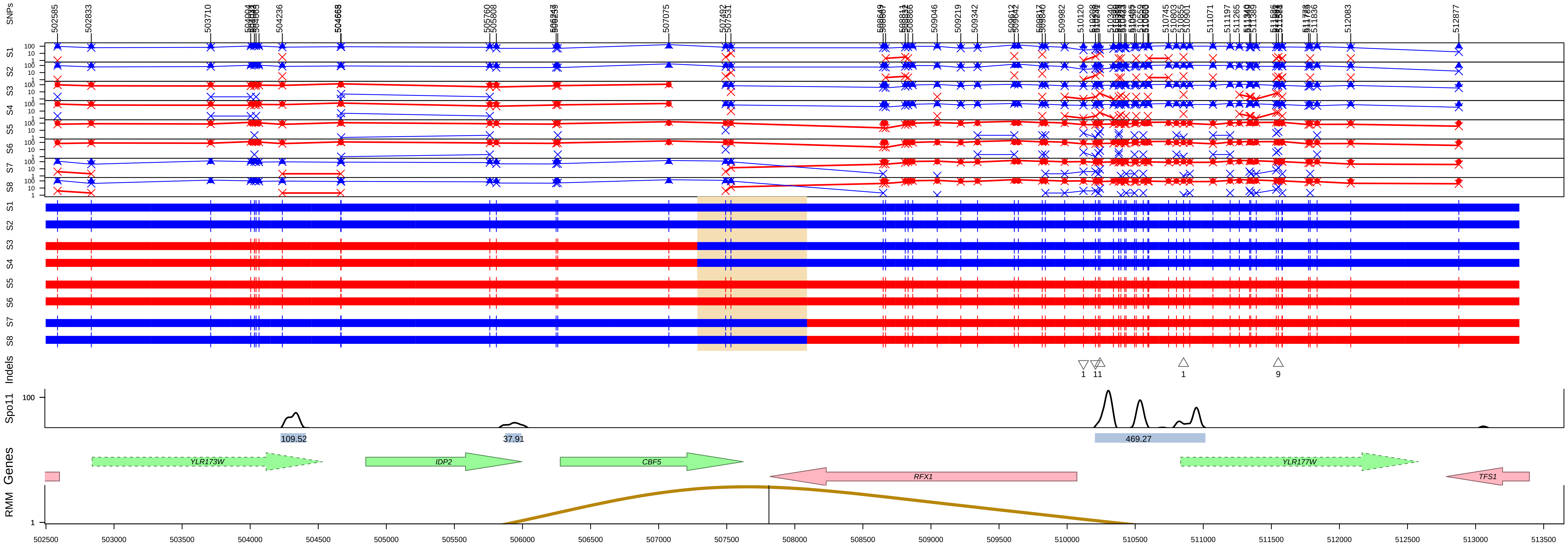

Genotype=TCMN6 Id=77 Chromo=12 Type=Full Conversion
Estimated: DSBs=1, COs=1, NCOs=0
Pattern=(2:6)_(4:4aCO)
Mid Len=806bp Midpoint=507686.75 Variants=2
Red=S288c, Blue=SK1

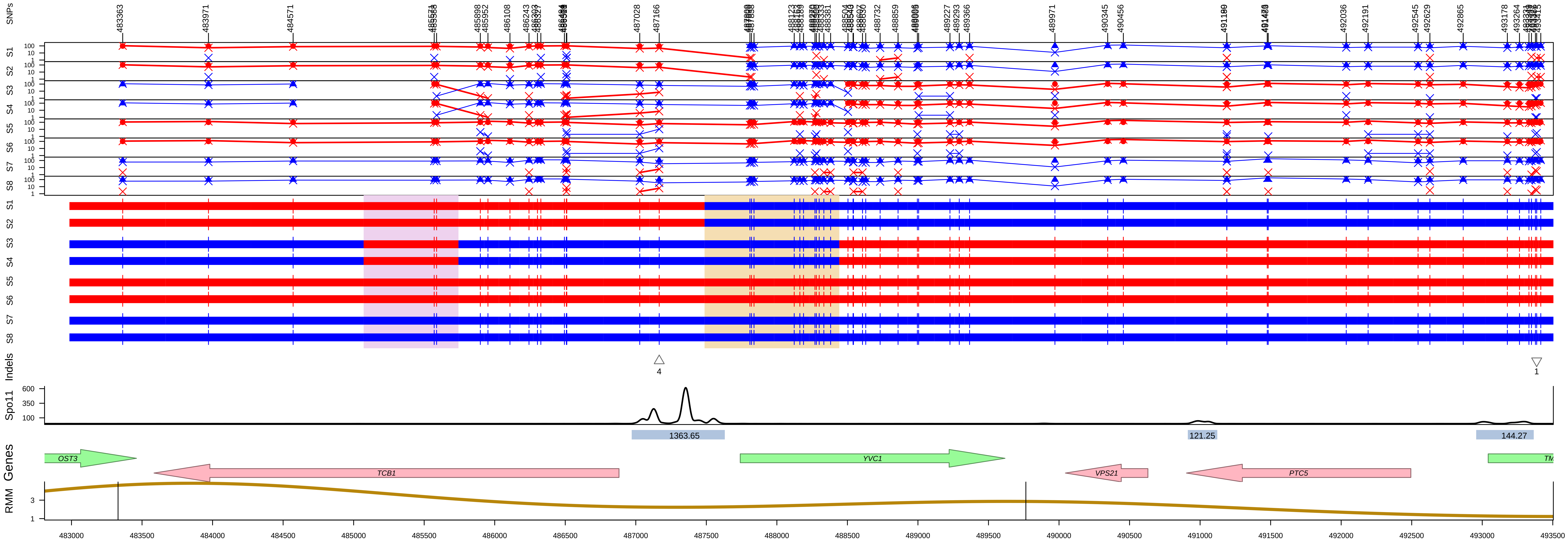

Genotype=TCMN6 Id=116 Chromo=15 Type=Full Conversion
Estimated: DSBs=1, COs=1, NCOs=0
Pattern=(2:6)_(4:4aCO)
Mid Len=955bp Midpoint=487965 Variants=11
Red=S288c, Blue=SK1

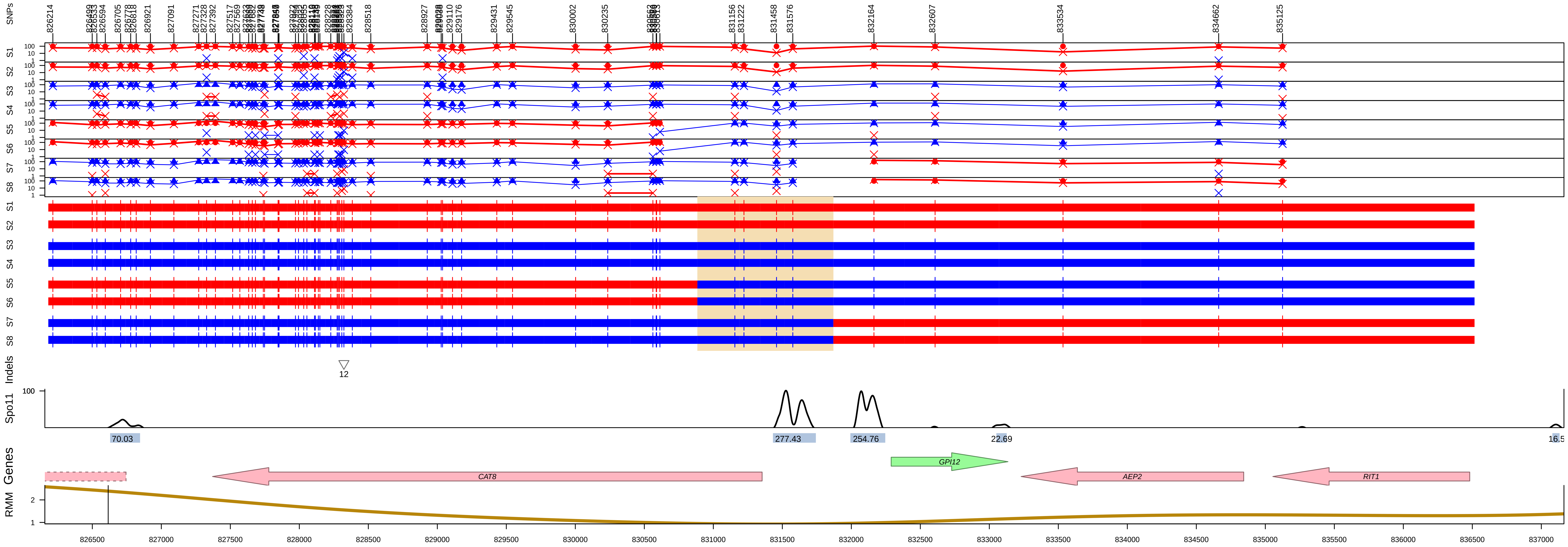

Genotype=TCMN6 Id=92 Chromo=13 Type=Full Conversion
Estimated: DSBs=1, COs=1, NCOs=0
Pattern=(2:6)_(4:4aCO)
Mid Len=986bp Midpoint=831377.25 Variants=4
Red=S288c, Blue=SK1

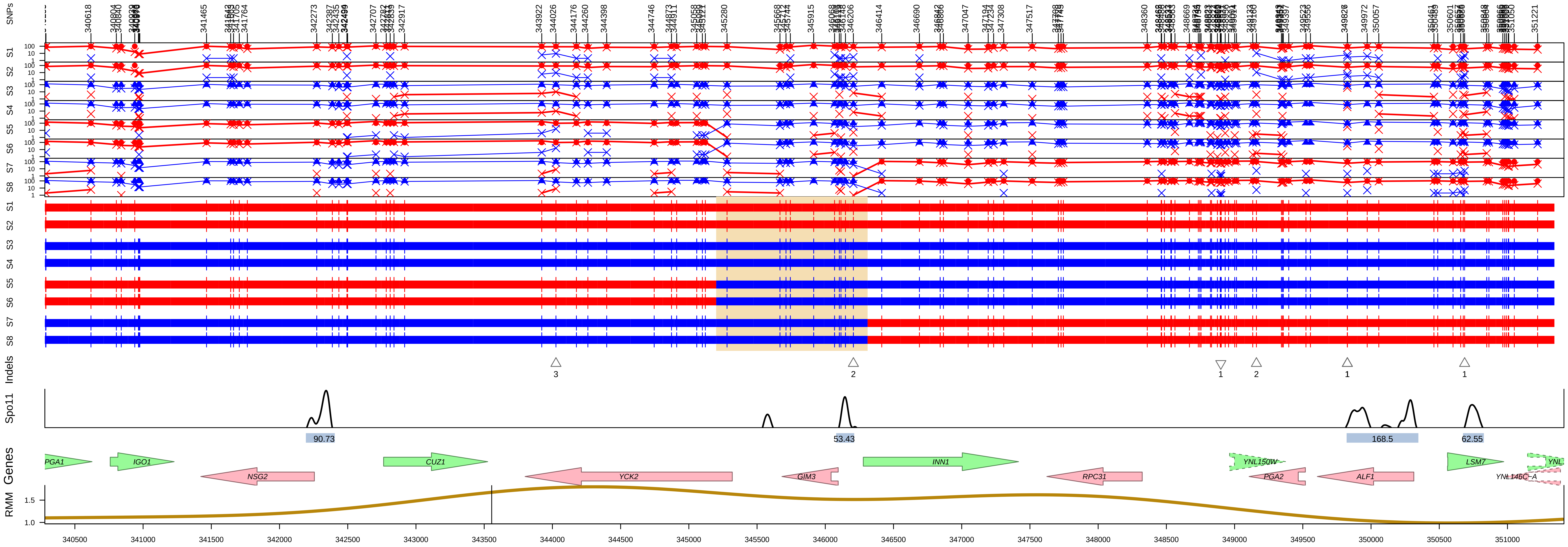

Genotype=TCMN6 Id=101 Chromo=14 Type=Full Conversion
Estimated: DSBs=1, COs=1, NCOs=0
Pattern=(2:6)_(4:4aCO)
Mid Len=1110bp Midpoint=345755.25 Variants=10
Red=S288c, Blue=SK1

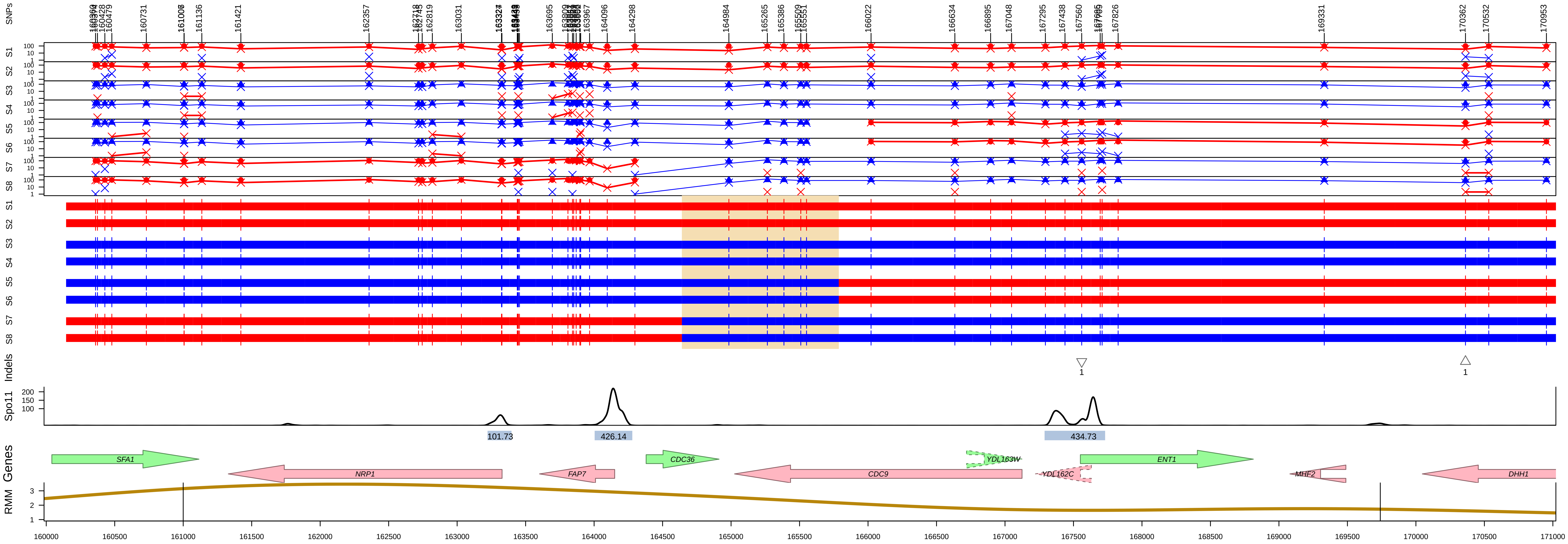

Genotype=TCMN6 Id=18 Chromo=4 Type=Full Conversion
Estimated: DSBs=1, COs=1, NCOs=0
Pattern=(2:6)_(4:4aCO)
Mid Len=1146bp Midpoint=165213.75 Variants=5
Red=S288c, Blue=SK1

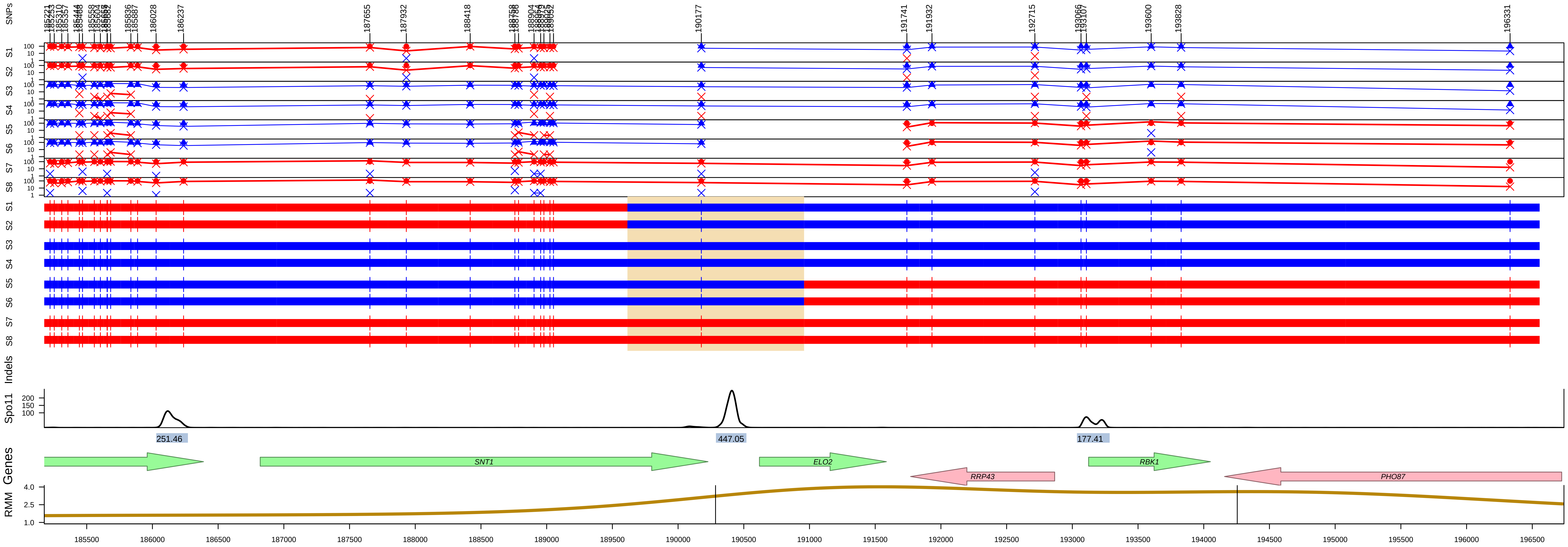

Genotype=TCMN6 Id=16 Chromo=3 Type=Full Conversion
Estimated: DSBs=1, COs=1, NCOs=0
Pattern=(2:6)_(4:4aCO)
Mid Len=1344bp Midpoint=190286.75 Variants=1
Red=S288c, Blue=SK1

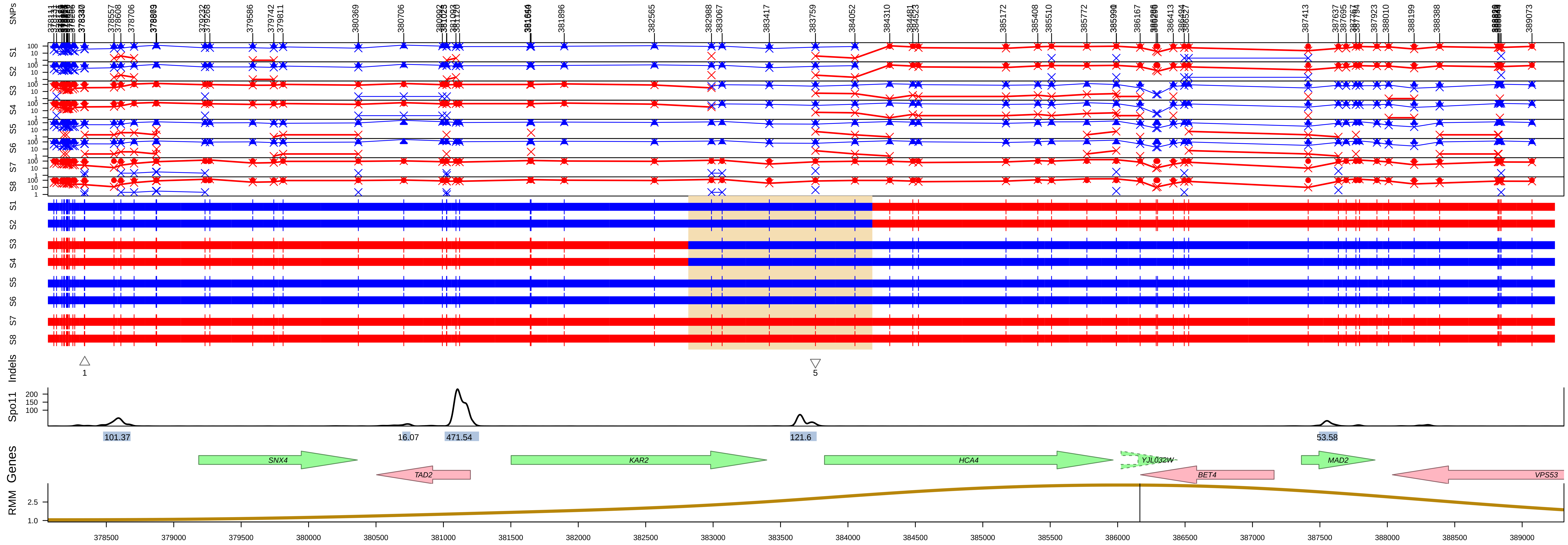

Genotype=TCMN6 Id=63 Chromo=10 Type=Full Conversion
Estimated: DSBs=1, COs=1, NCOs=0
Pattern=(2:6)_(4:4aCO)
Mid Len=1365bp Midpoint=383498.5 Variants=4
Red=S288c, Blue=SK1

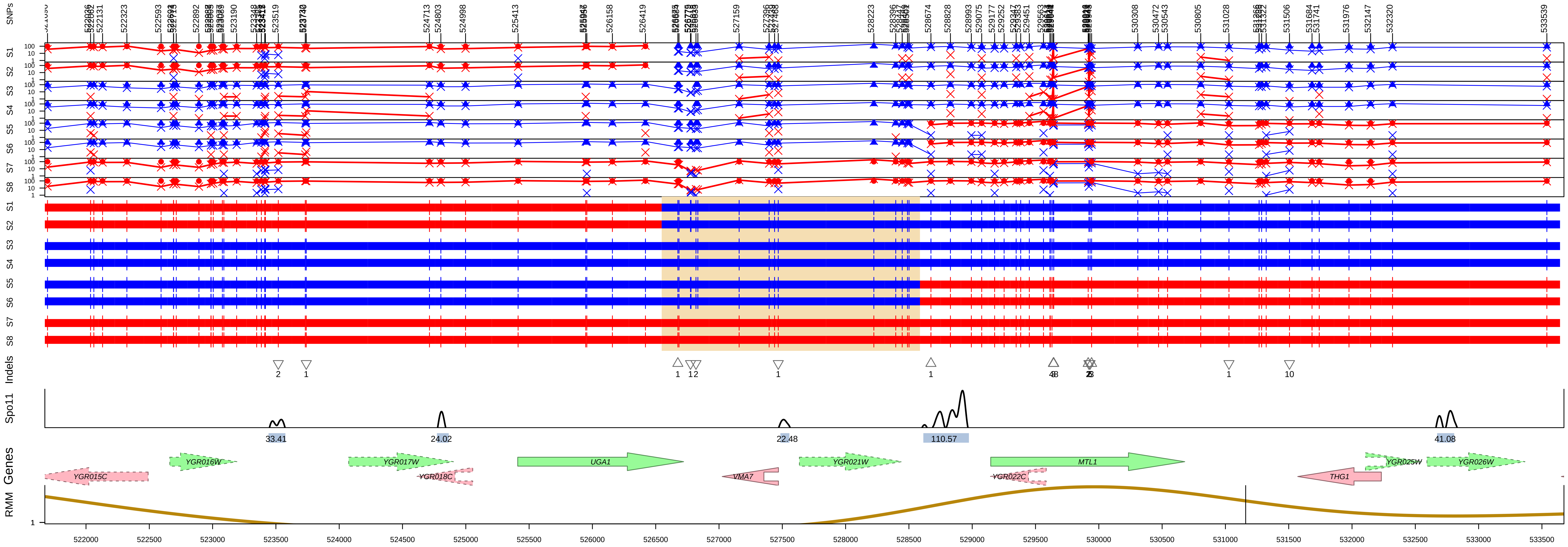

Genotype=TCMN6 Id=47 Chromo=7 Type=Full Conversion
Estimated: DSBs=1, COs=1, NCOs=0
Pattern=(2:6)_(4:4aCO)
Mid Len=2040bp Midpoint=527567.25 Variants=11
Red=S288c, Blue=SK1

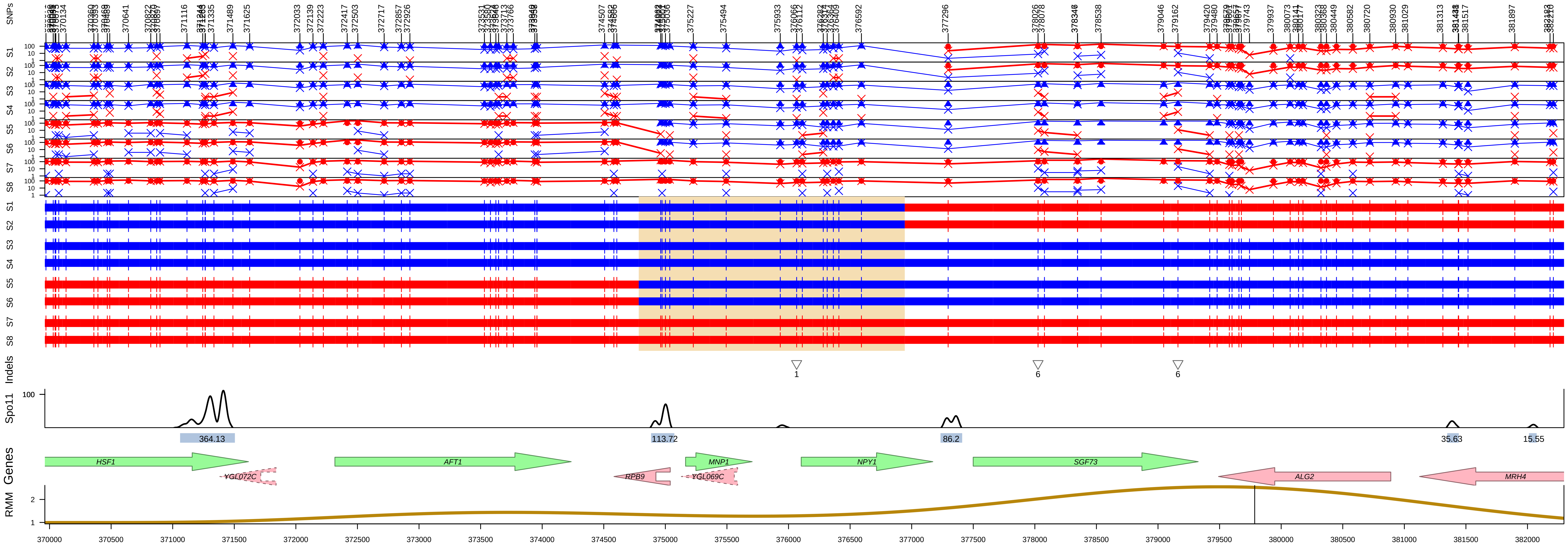

Genotype=TCMN6 Id=45 Chromo=7 Type=Full Conversion
Estimated: DSBs=1, COs=1, NCOs=0
Pattern=(2:6)_(4:4aCO)
Mid Len=2160bp Midpoint=375864 Variants=14
Red=S288c, Blue=SK1

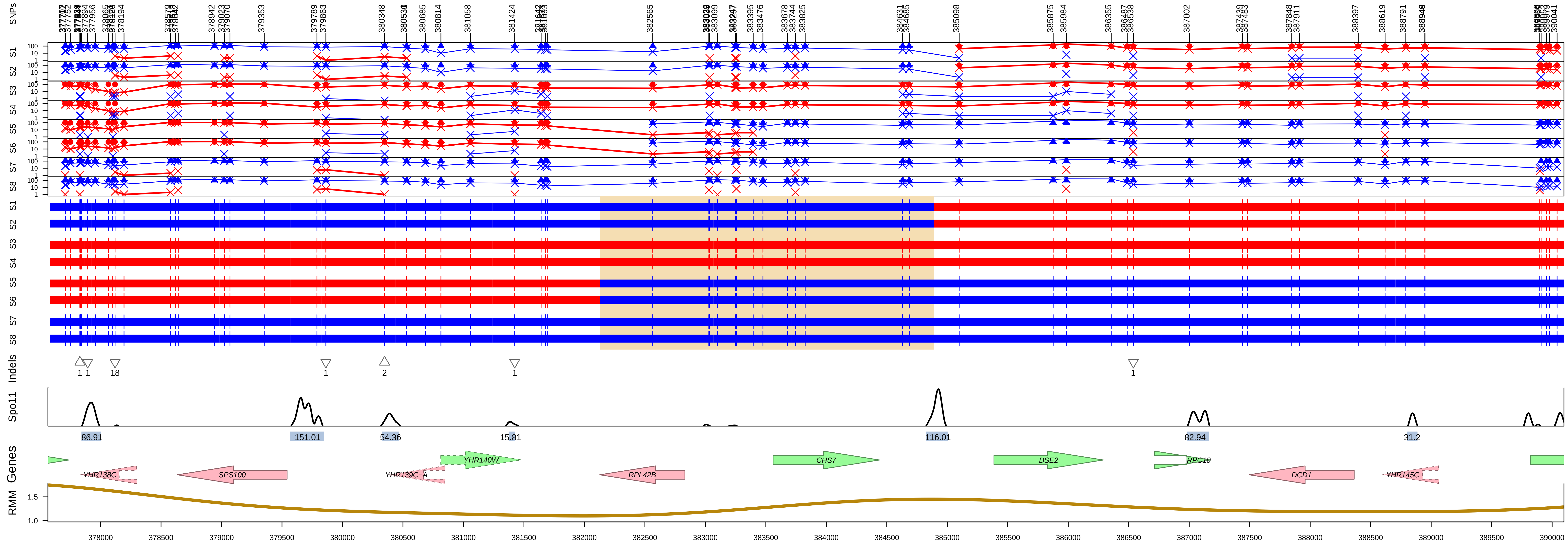

Genotype=TCMN6 Id=54 Chromo=8 Type=Full Conversion
Estimated: DSBs=1, COs=1, NCOs=0
Pattern=(2:6)_(4:4aCO)
Mid Len=2762bp Midpoint=383510.25 Variants=14
Red=S288c, Blue=SK1

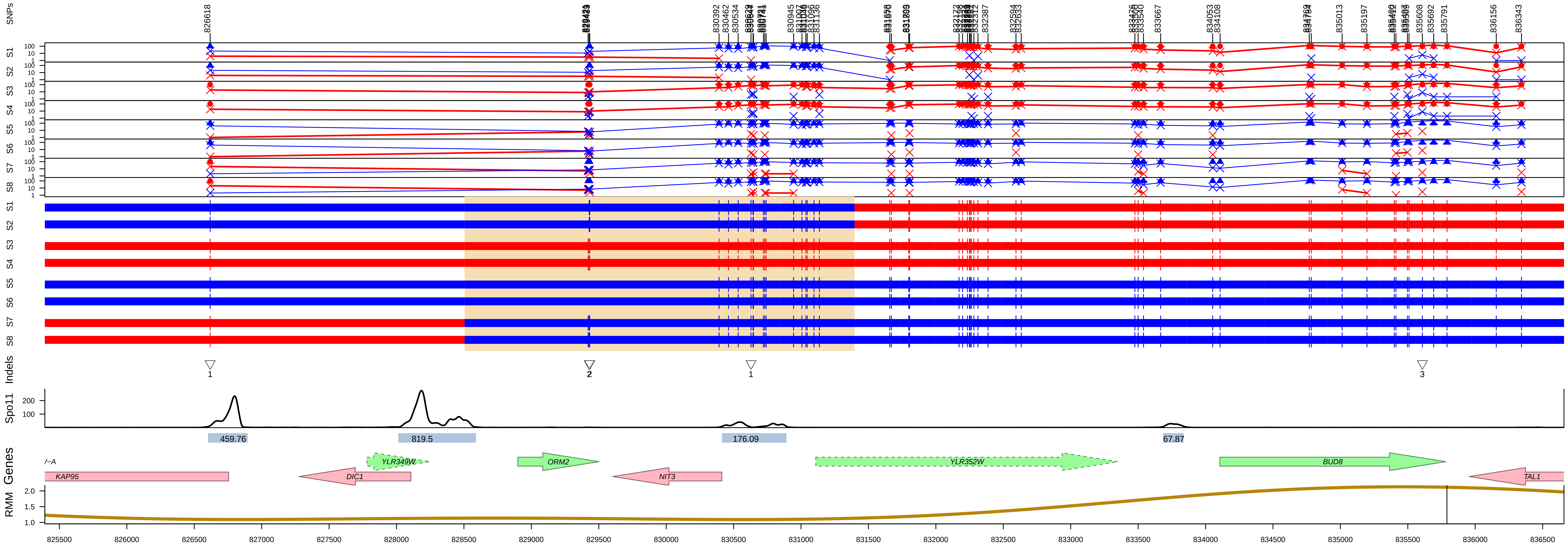

Genotype=TCMN6 Id=81 Chromo=12 Type=Full Conversion
Estimated: DSBs=1, COs=1, NCOs=0
Pattern=(2:6)_(4:4aCO)
Mid Len=2892bp Midpoint=829951 Variants=15
Red=S288c, Blue=SK1

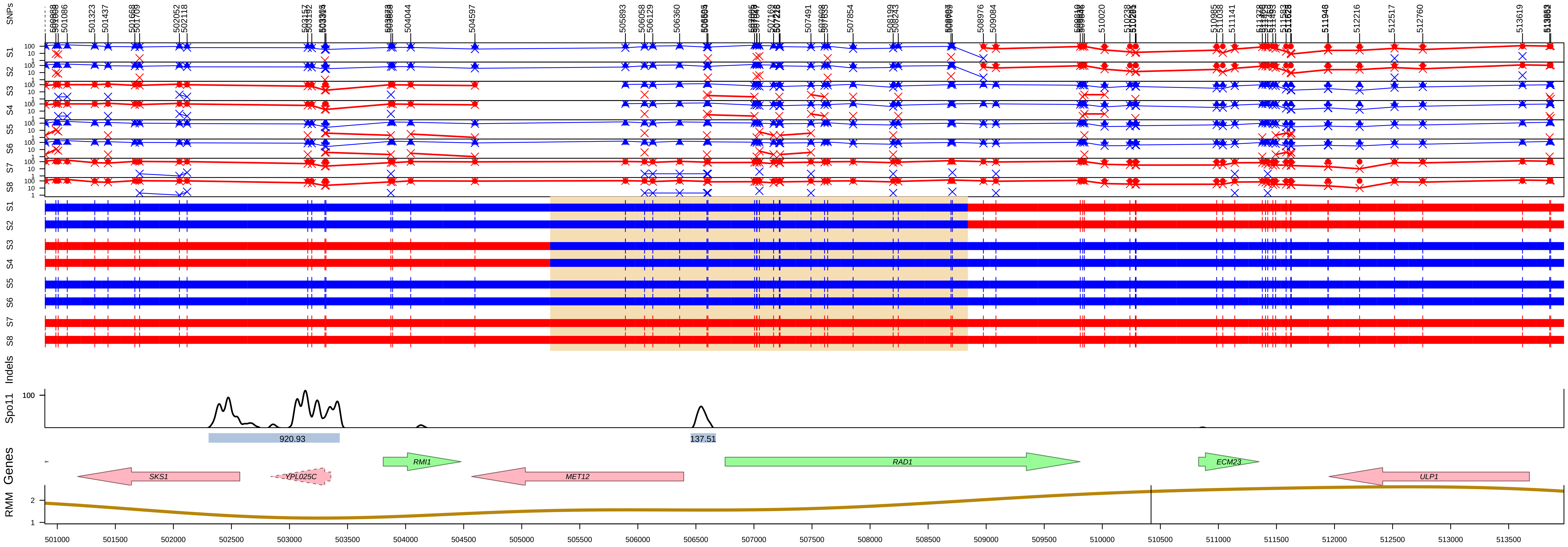

Genotype=TCMN6 Id=128 Chromo=16 Type=Full Conversion
Estimated: DSBs=1, COs=1, NCOs=0
Pattern=(2:6)_(4:4aCO)
Mid Len=3598bp Midpoint=507043.75 Variants=21
Red=S288c, Blue=SK1

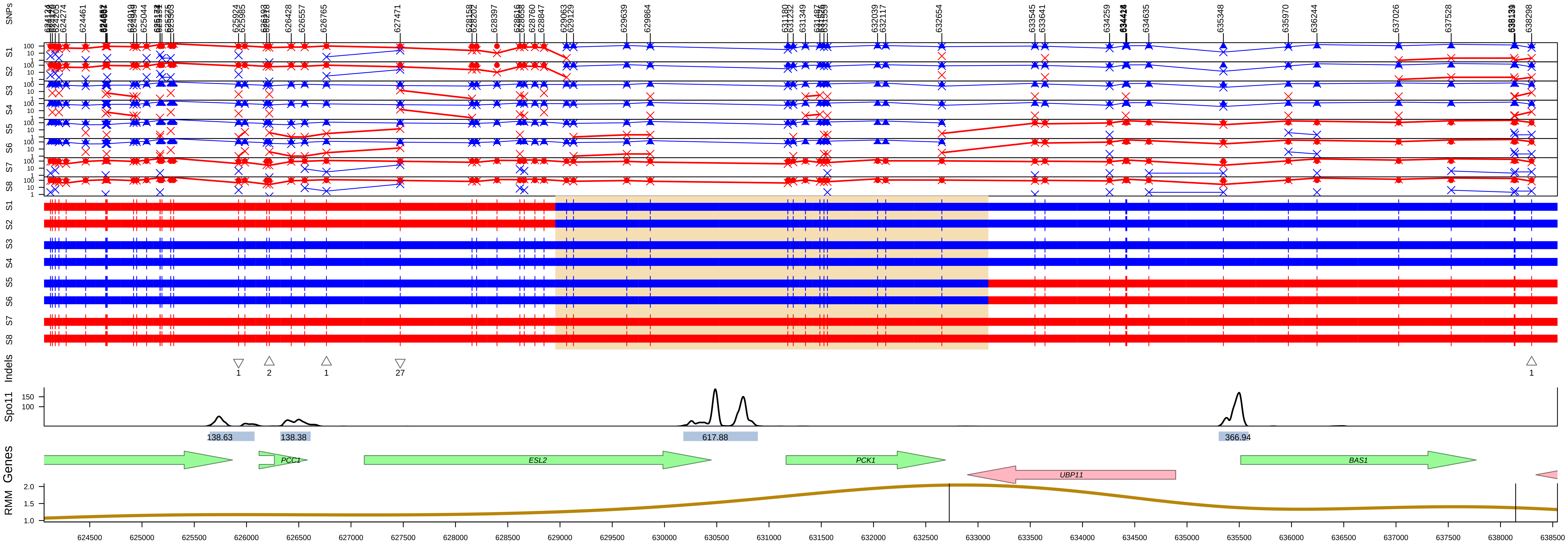

Genotype=TCMN6 Id=74 Chromo=11 Type=Full Conversion
Estimated: DSBs=1, COs=1, NCOs=0
Pattern=(2:6)_(4:4aCO)
Mid Len=4144bp Midpoint=631027.25 Variants=13
Red=S288c, Blue=SK1

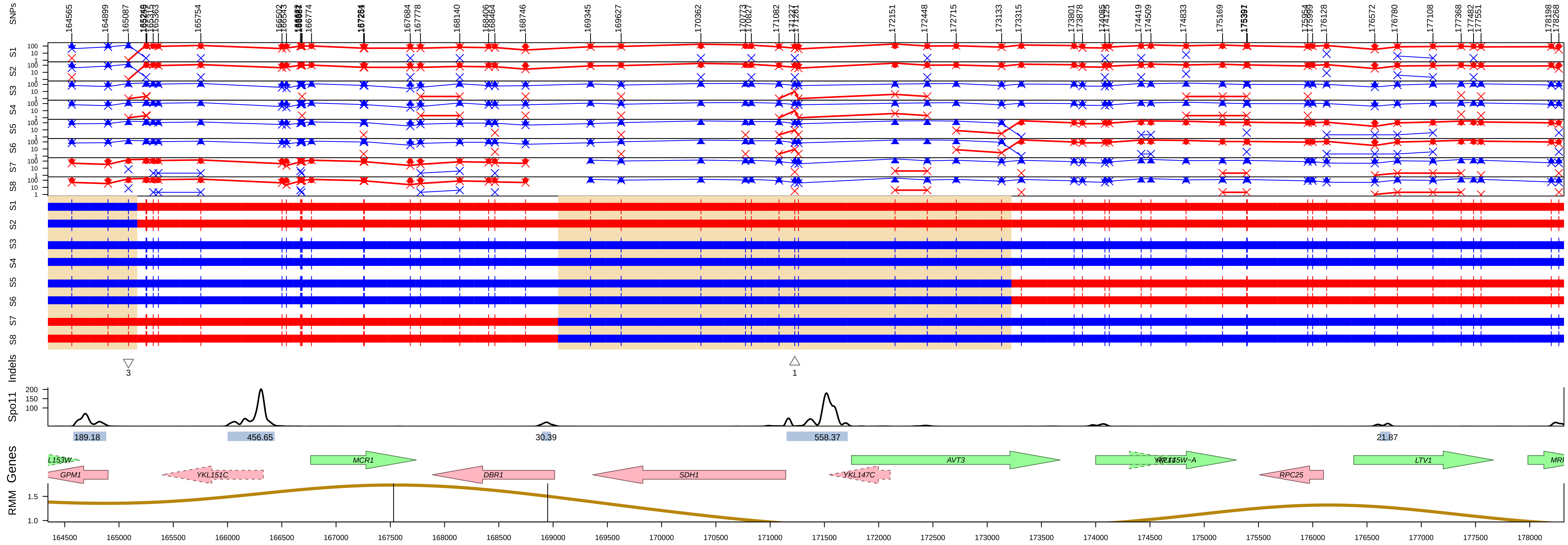

Genotype=TCMN6 Id=69 Chromo=11 Type=Full Conversion
Estimated: DSBs=1, COs=1, NCOs=0
Pattern=(2:6)_(4:4aCO)
Mid Len=4178bp Midpoint=171134.75 Variants=12
Red=S288c, Blue=SK1

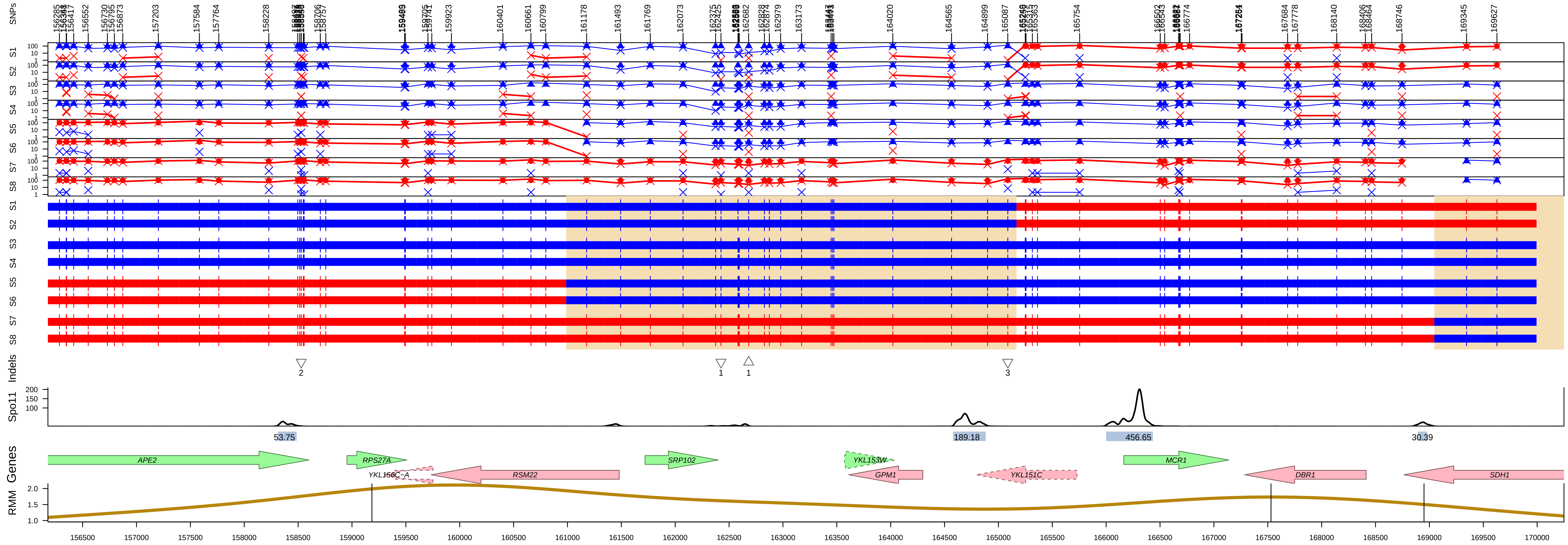

Genotype=TCMN6 Id=68 Chromo=11 Type=Full Conversion
Estimated: DSBs=1, COs=1, NCOs=0
Pattern=(2:6)_(4:4aCO)
Mid Len=4180bp Midpoint=163078.25 Variants=19
Red=S288c, Blue=SK1

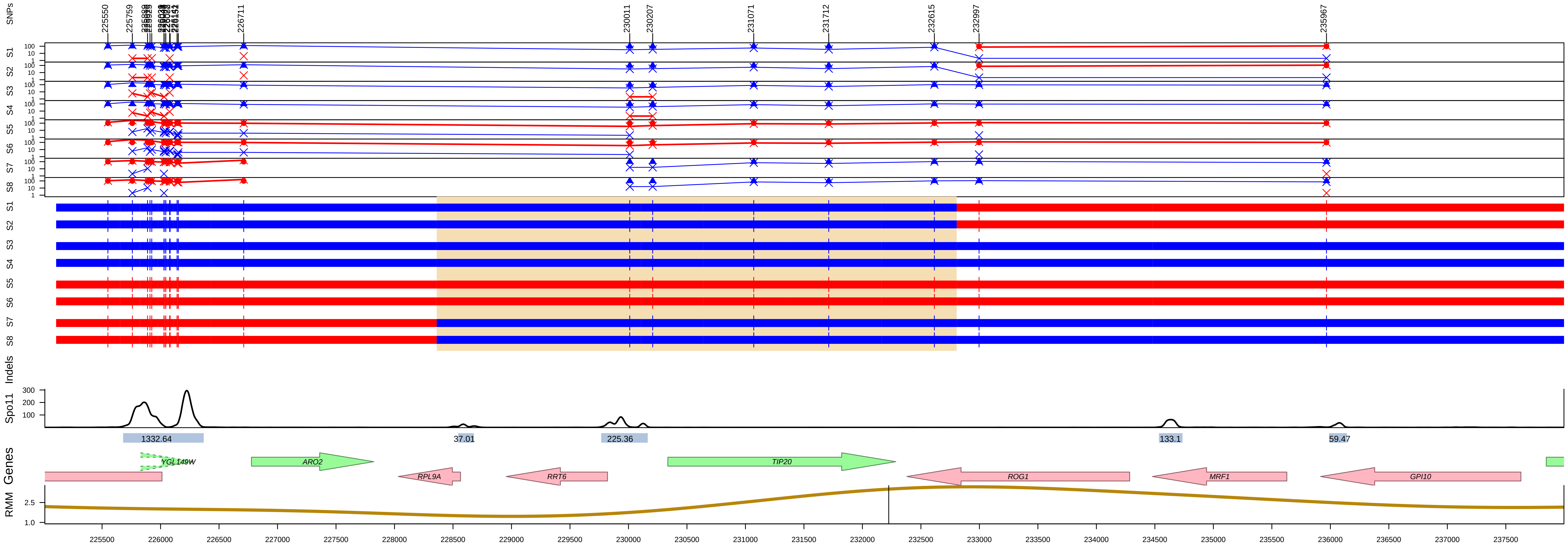

Genotype=TCMN6 Id=41 Chromo=7 Type=Full Conversion
Estimated: DSBs=1, COs=1, NCOs=0
Pattern=(2:6)_(4:4aCO)
Mid Len=4445bp Midpoint=230583.5 Variants=5
Red=S288c, Blue=SK1

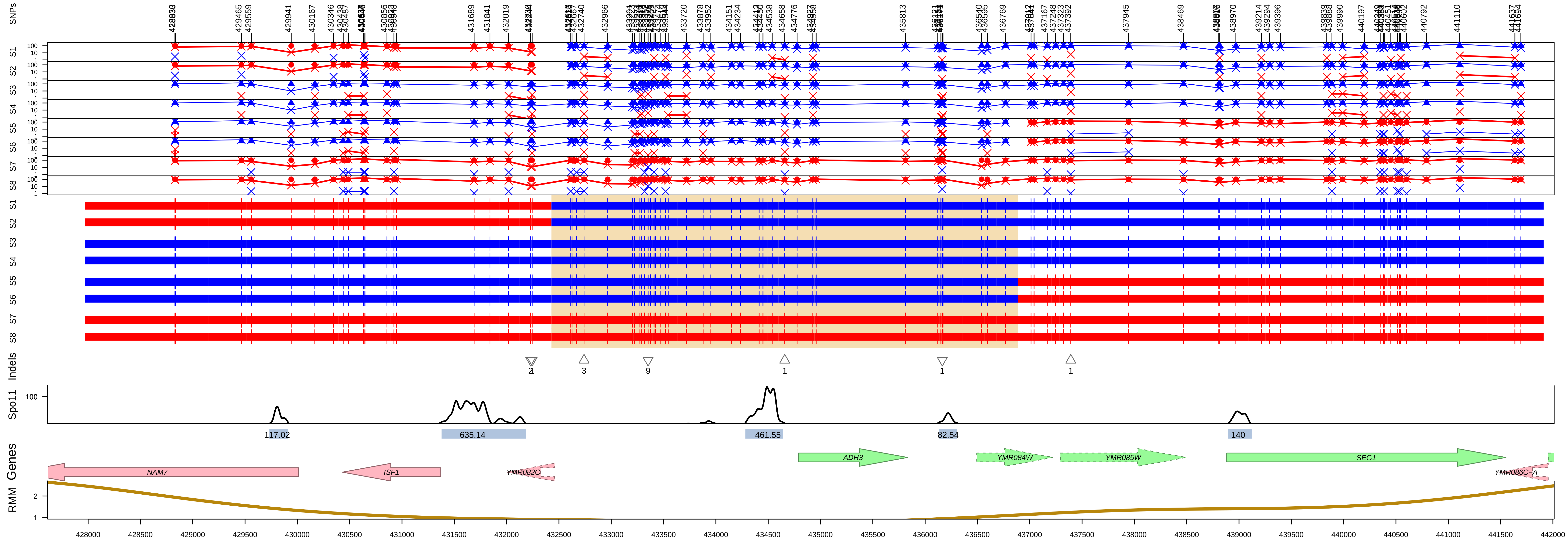

Genotype=TCMN6 Id=87 Chromo=13 Type=Full Conversion
Estimated: DSBs=1, COs=1, NCOs=0
Pattern=(2:6)_(4:4aCO)
Mid Len=4462bp Midpoint=434659.5 Variants=37
Red=S288c, Blue=SK1

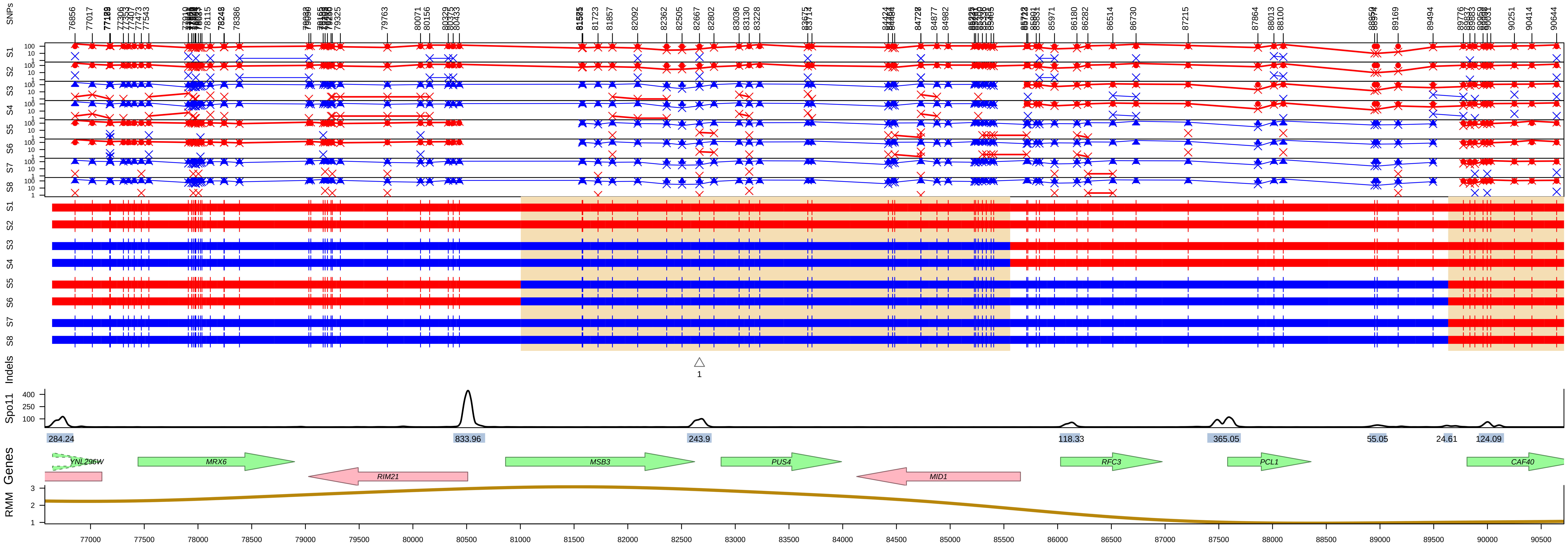

Genotype=TCMN6 Id=94 Chromo=14 Type=Full Conversion
Estimated: DSBs=1, COs=1, NCOs=0
Pattern=(2:6)_(4:4aCO)
Mid Len=4554bp Midpoint=83281.25 Variants=28
Red=S288c, Blue=SK1

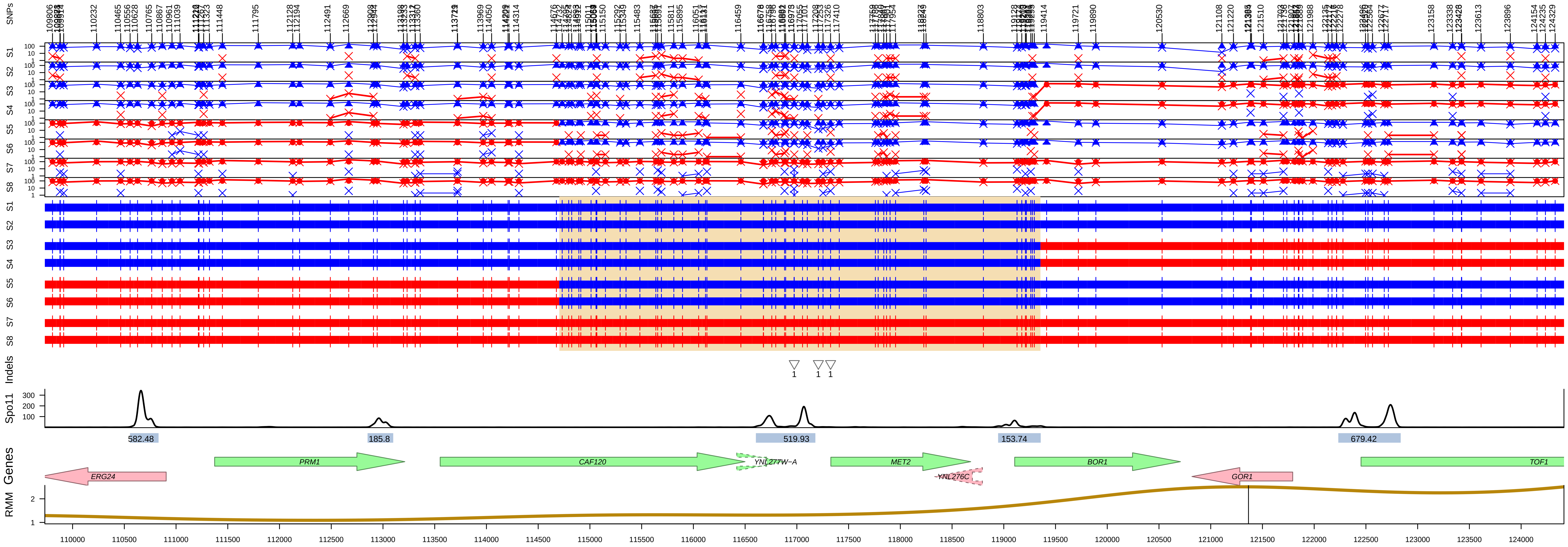

Genotype=TCMN6 Id=96 Chromo=14 Type=Full Conversion
Estimated: DSBs=1, COs=1, NCOs=0
Pattern=(2:6)_(4:4aCO)
Mid Len=4650bp Midpoint=117029.25 Variants=52
Red=S288c, Blue=SK1

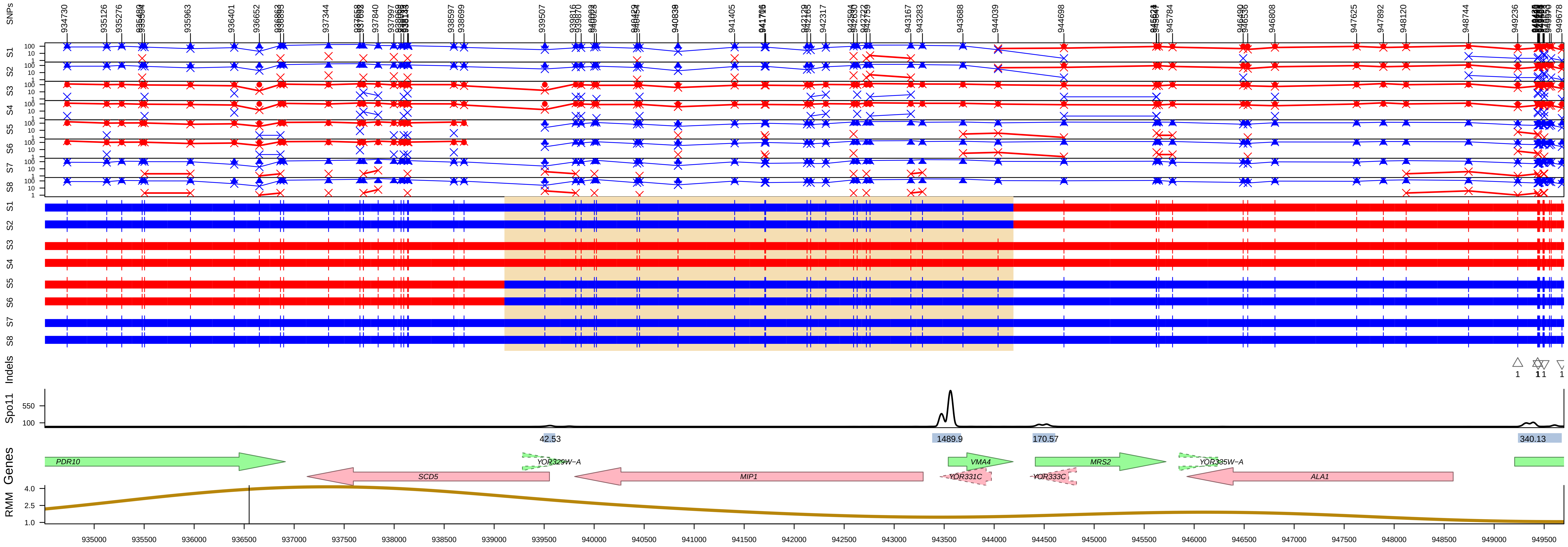

Genotype=TCMN6 Id=121 Chromo=15 Type=Full Conversion
Estimated: DSBs=1, COs=1, NCOs=0
Pattern=(2:6)_(4:4aCO)
Mid Len=5090bp Midpoint=941648 Variants=22
Red=S288c, Blue=SK1

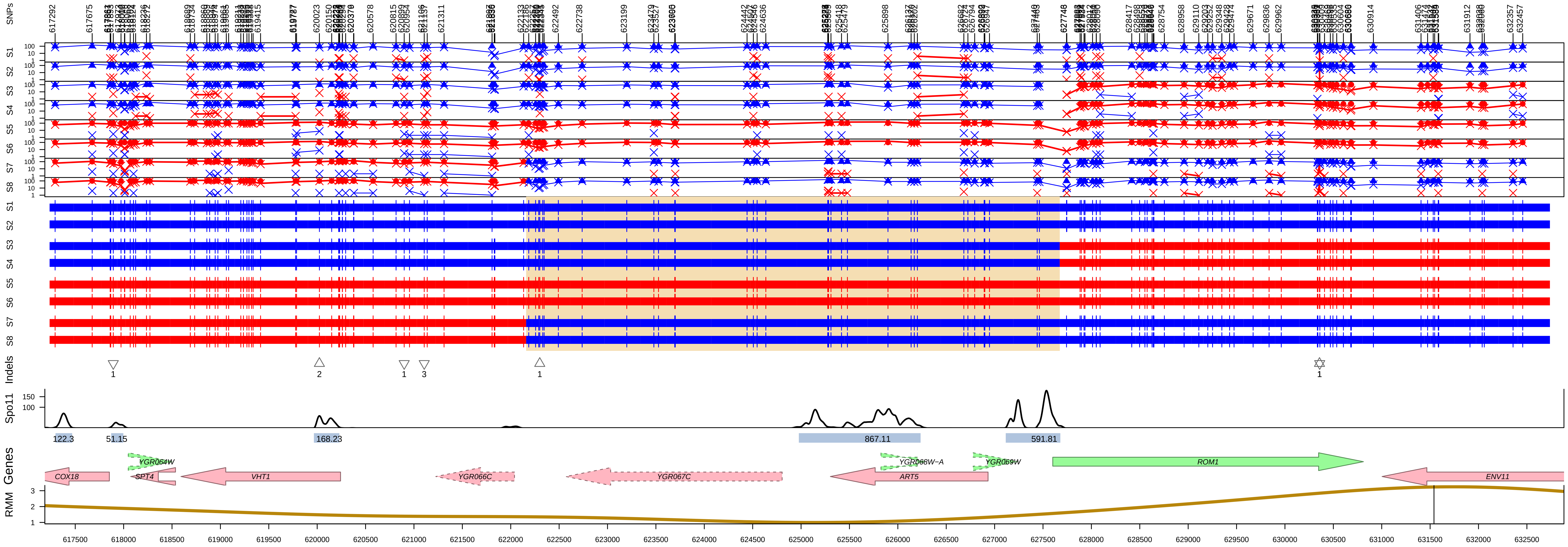

Genotype=TCMN6 Id=48 Chromo=7 Type=Full Conversion
Estimated: DSBs=1, COs=1, NCOs=0
Pattern=(2:6)_(4:4aCO)
Mid Len=5514bp Midpoint=624916.25 Variants=36
Red=S288c, Blue=SK1

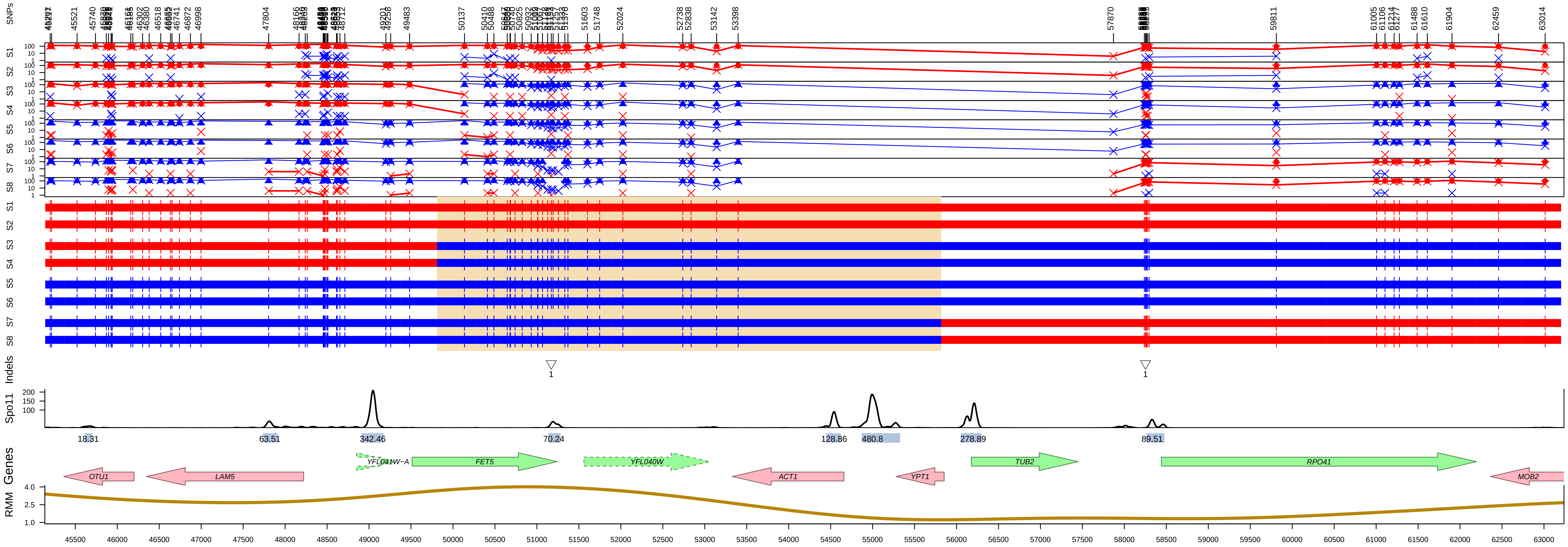

Genotype=TCMN6 Id=31 Chromo=6 Type=Full Conversion
Estimated: DSBs=1, COs=1, NCOs=0
Pattern=(2:6)_(4:4aCO)
Mid Len=6008bp Midpoint=52814 Variants=21
Red=S288c, Blue=SK1

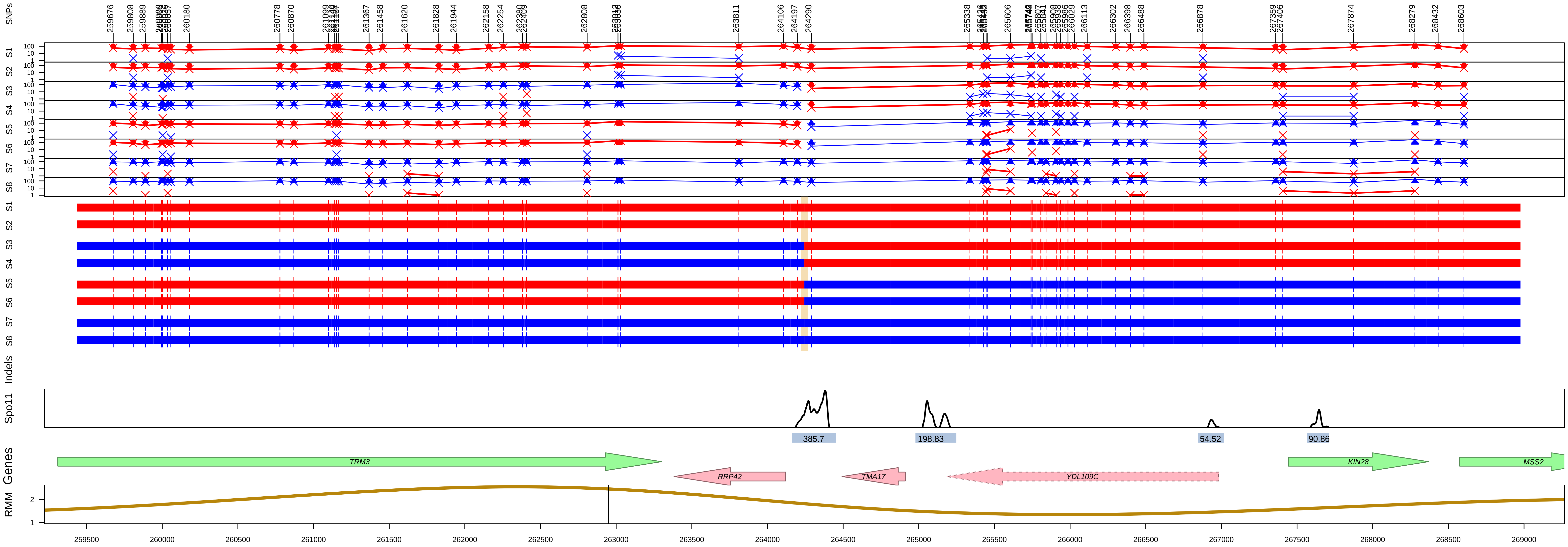

Genotype=TCMN6 Id=20 Chromo=4 Type=No Strand Transfer
Estimated: DSBs=1, COs=1, NCOs=0
Pattern=(4:4aCO)
Mid Len=46bp Midpoint=264243.5 Variants=0
Red=S288c, Blue=SK1

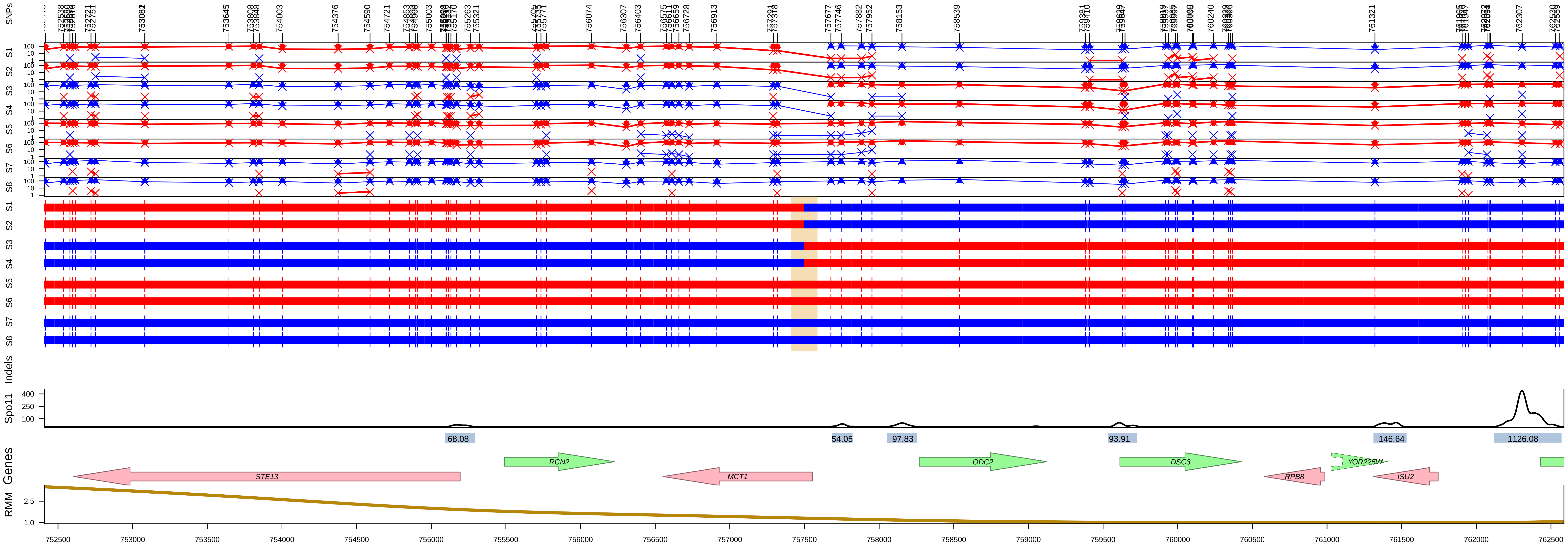

Genotype=TCMN6 Id=120 Chromo=15 Type=No Strand Transfer
Estimated: DSBs=1, COs=1, NCOs=0
Pattern=(4:4aCO)
Mid Len=179bp Midpoint=757497.5 Variants=0
Red=S288c, Blue=SK1

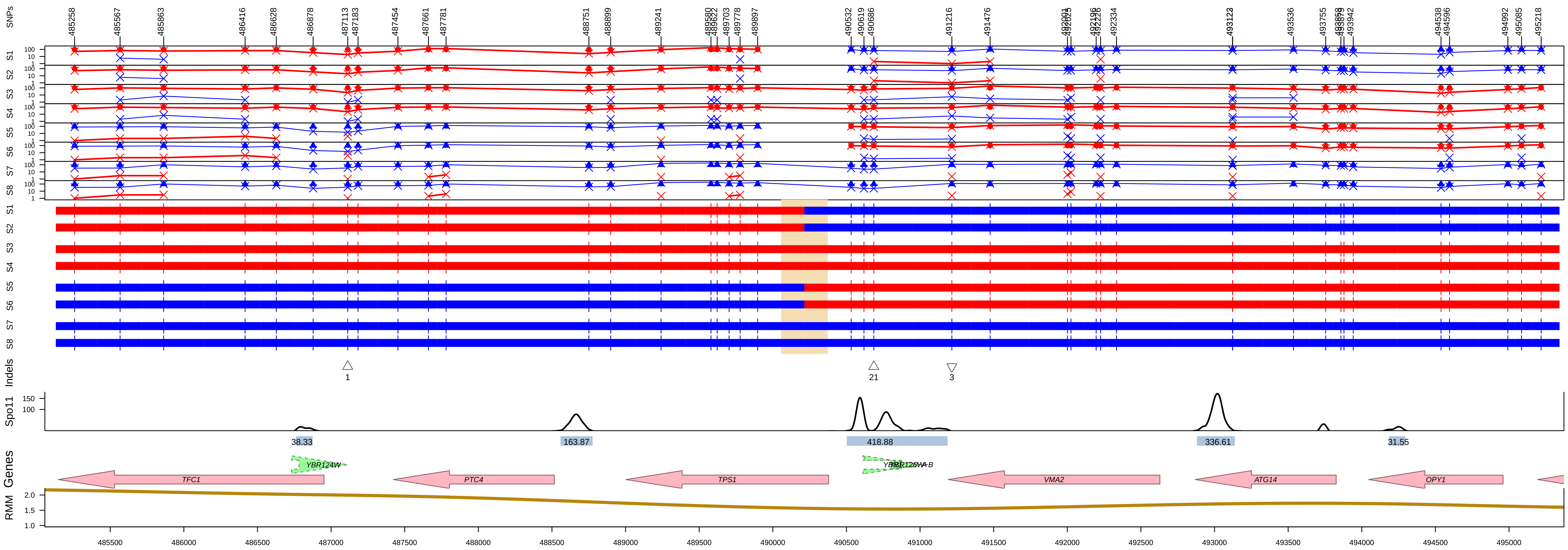

Genotype=TCMN6 Id=11 Chromo=2 Type=No Strand Transfer
Estimated: DSBs=1, COs=1, NCOs=0
Pattern=(4:4aCO)
Mid Len=317bp Midpoint=490214.5 Variants=0
Red=S288c, Blue=SK1

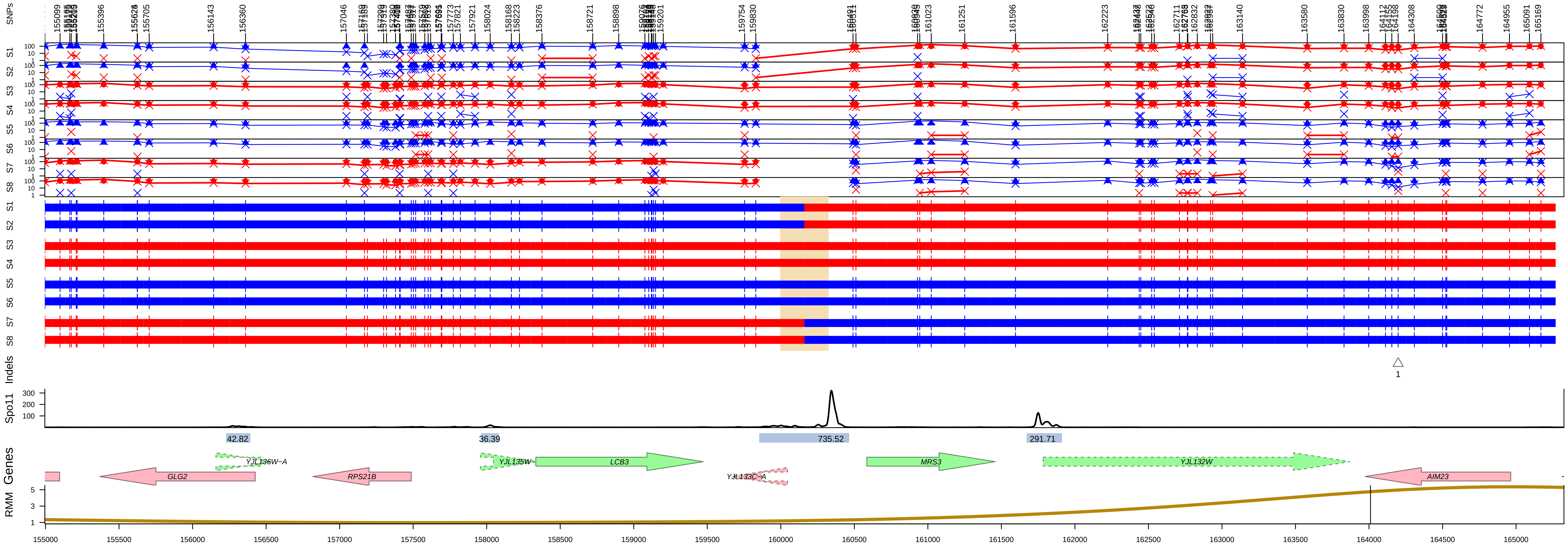

Genotype=TCMN6 Id=61 Chromo=10 Type=No Strand Transfer
Estimated: DSBs=1, COs=1, NCOs=0
Pattern=(4:4aCO)
Mid Len=330bp Midpoint=160160.5 Variants=0
Red=S288c, Blue=SK1

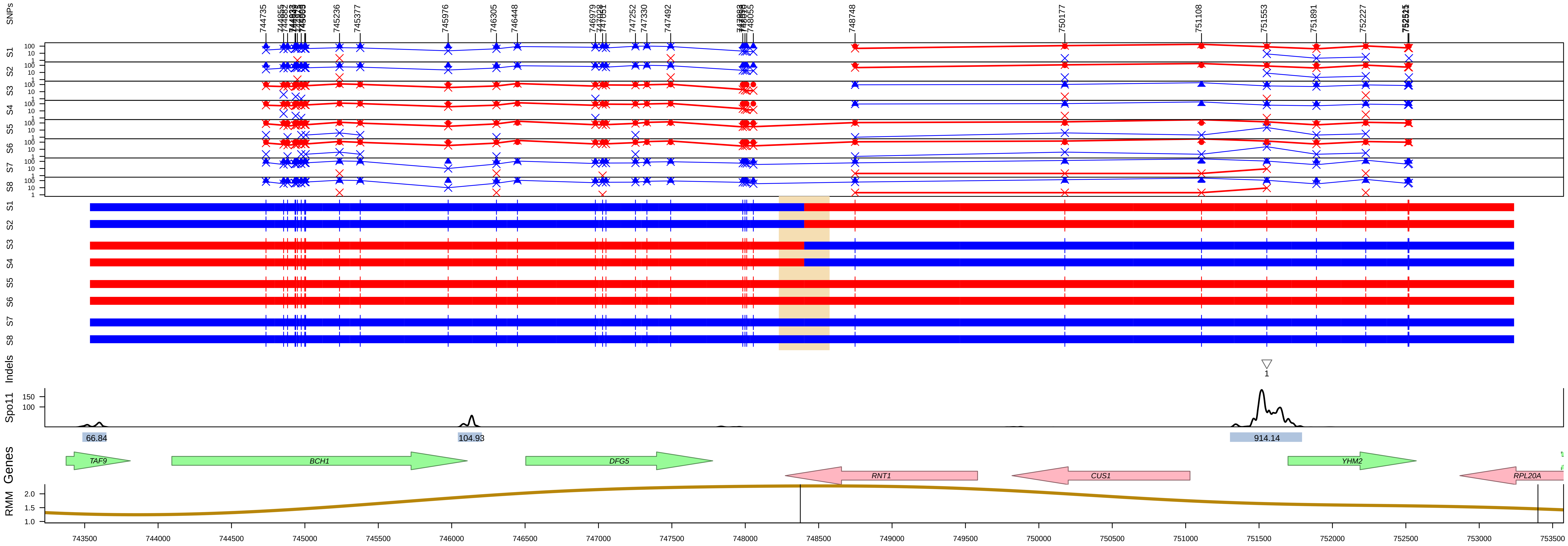

Genotype=TCMN6 Id=91 Chromo=13 Type=No Strand Transfer
Estimated: DSBs=1, COs=1, NCOs=0
Pattern=(4:4aCO)
Mid Len=346bp Midpoint=748401.5 Variants=0
Red=S288c, Blue=SK1

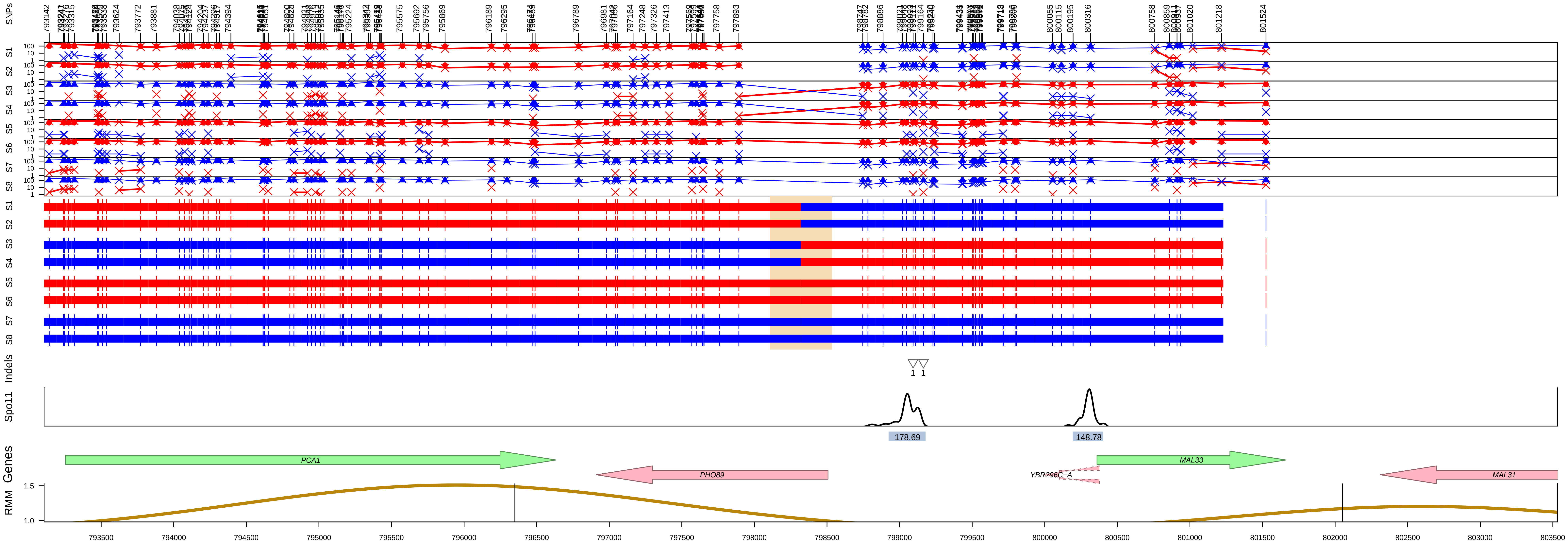

Genotype=TCMN6 Id=15 Chromo=2 Type=No Strand Transfer
Estimated: DSBs=1, COs=1, NCOs=0
Pattern=(4:4aCO)
Mid Len=426bp Midpoint=798320 Variants=0
Red=S288c, Blue=SK1

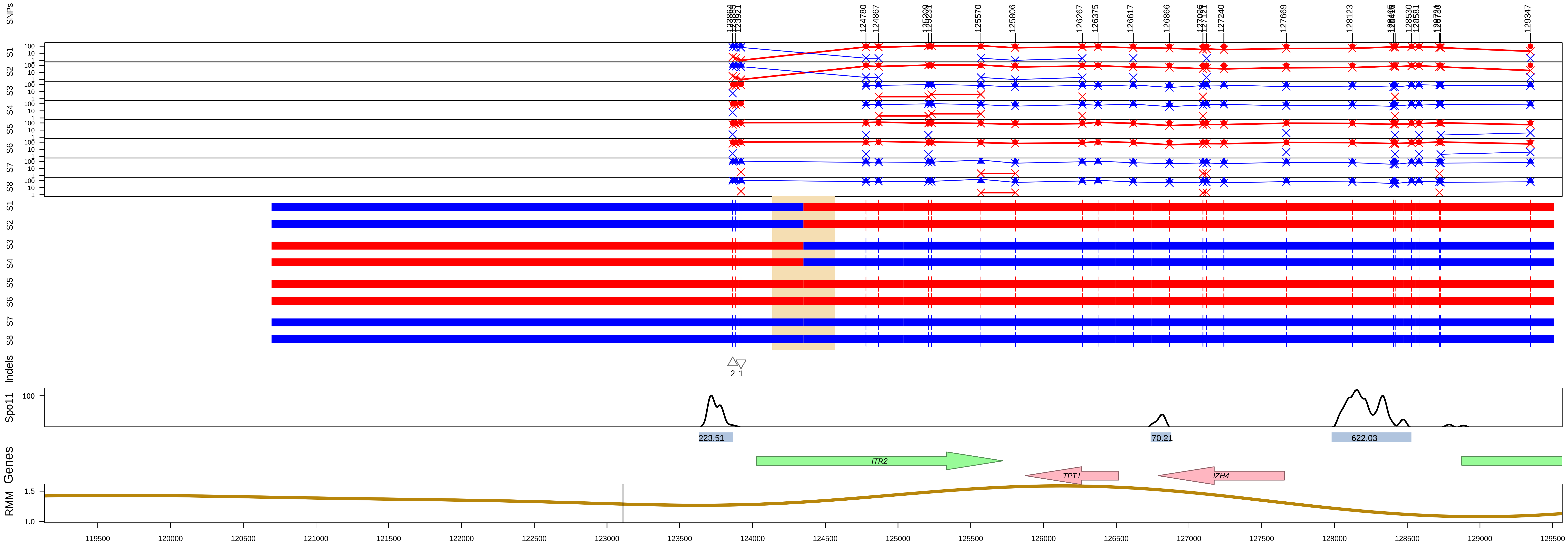

Genotype=TCMN6 Id=109 Chromo=15 Type=No Strand Transfer
Estimated: DSBs=1, COs=1, NCOs=0
Pattern=(4:4aCO)
Mid Len=429bp Midpoint=124350.5 Variants=0
Red=S288c, Blue=SK1

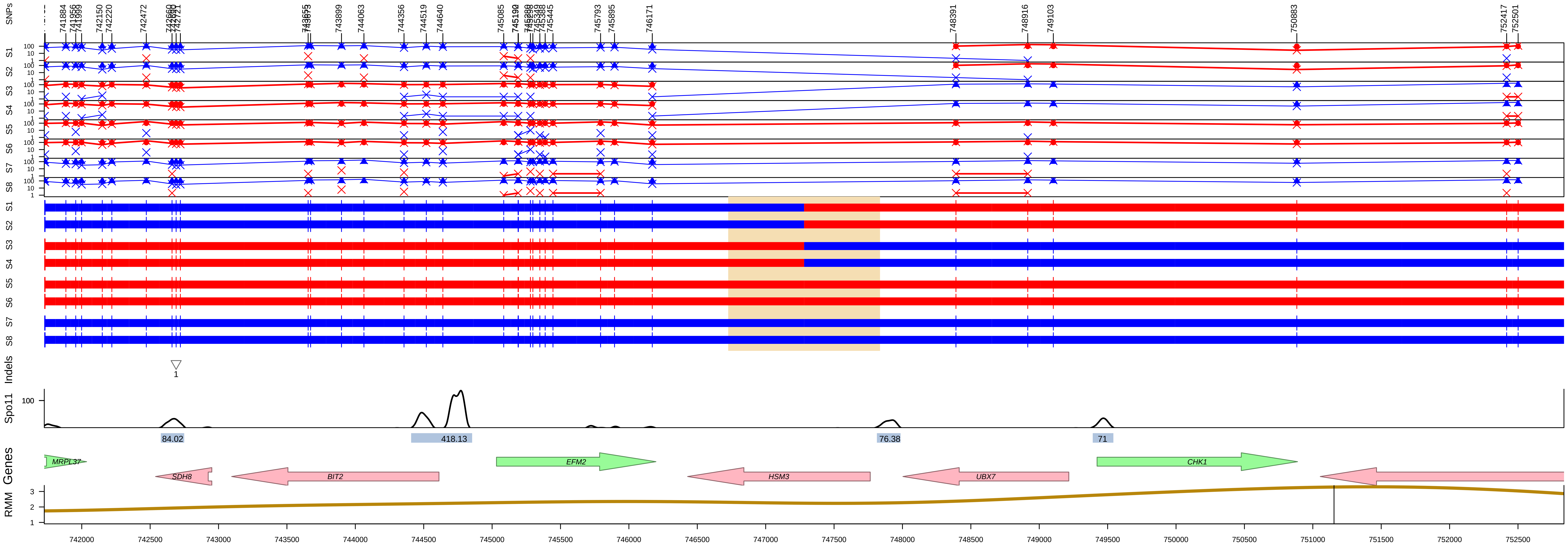

Genotype=TCMN6 Id=14 Chromo=2 Type=No Strand Transfer
Estimated: DSBs=1, COs=1, NCOs=0
Pattern=(4:4aCO)
Mid Len=1110bp Midpoint=747281 Variants=0
Red=S288c, Blue=SK1

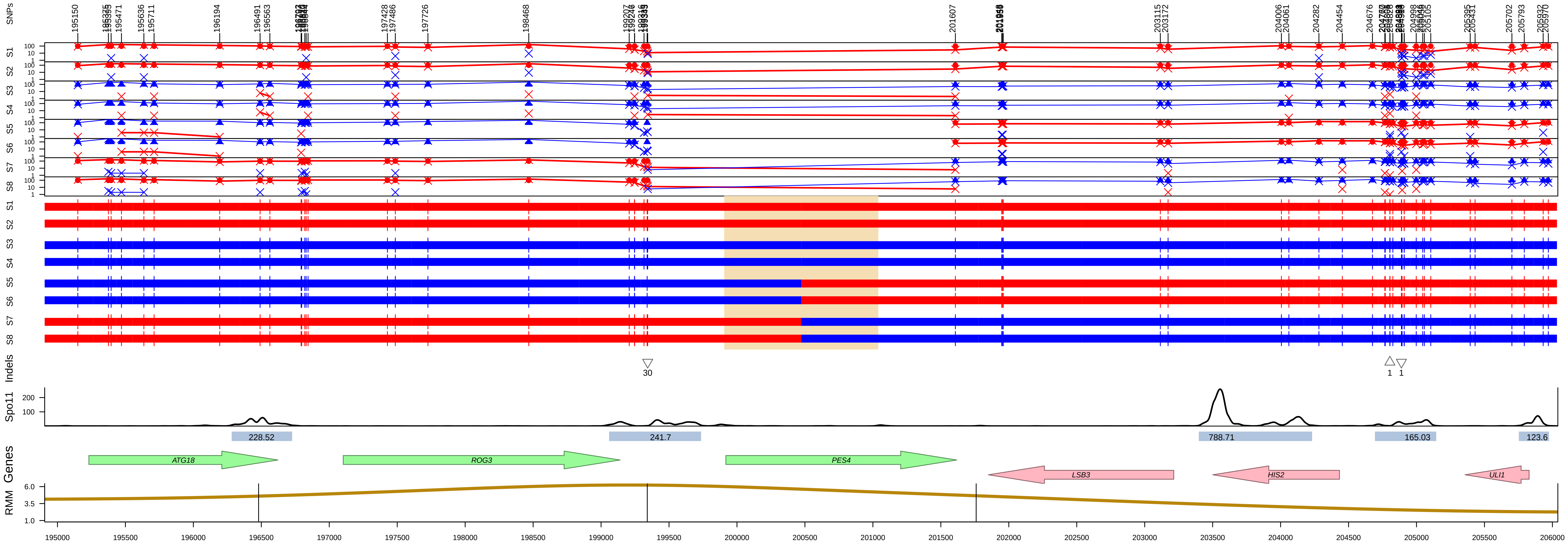

Genotype=TCMN6 Id=32 Chromo=6 Type=No Strand Transfer
Estimated: DSBs=1, COs=1, NCOs=0
Pattern=(4:4aCO)
Mid Len=1134bp Midpoint=200473 Variants=0
Red=S288c, Blue=SK1

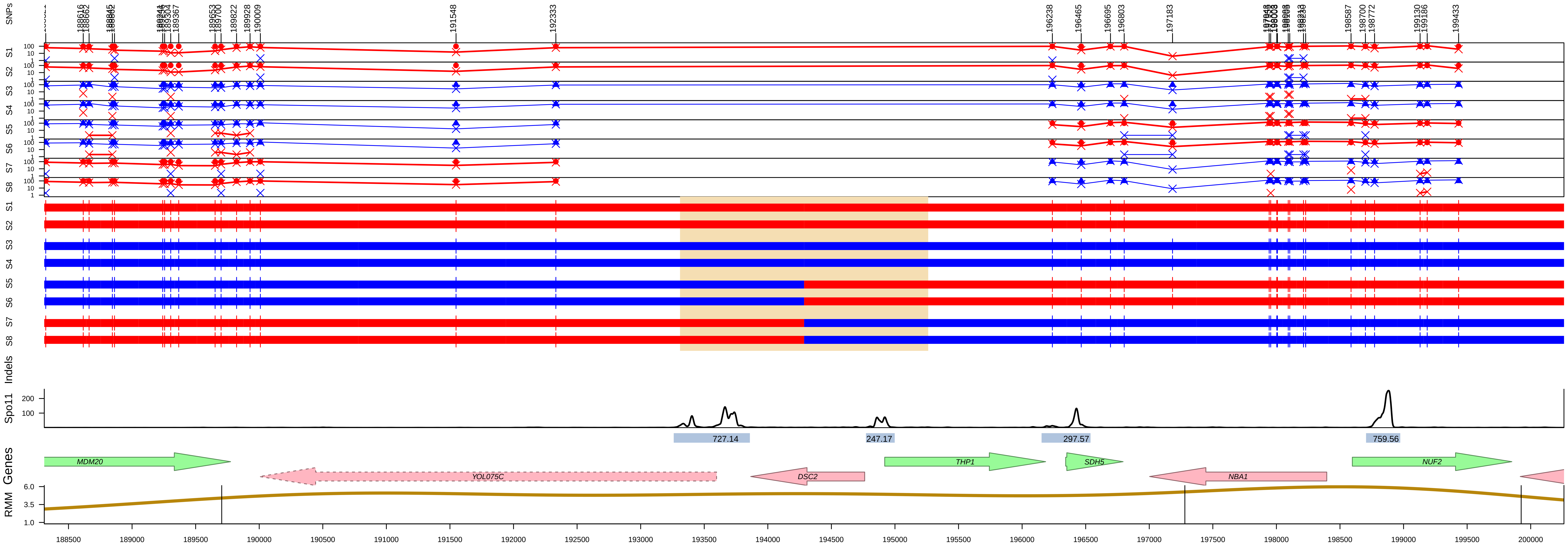

Genotype=TCMN6 Id=111 Chromo=15 Type=No Strand Transfer
Estimated: DSBs=1, COs=1, NCOs=0
Pattern=(4:4aCO)
Mid Len=1952bp Midpoint=194285.5 Variants=0
Red=S288c, Blue=SK1

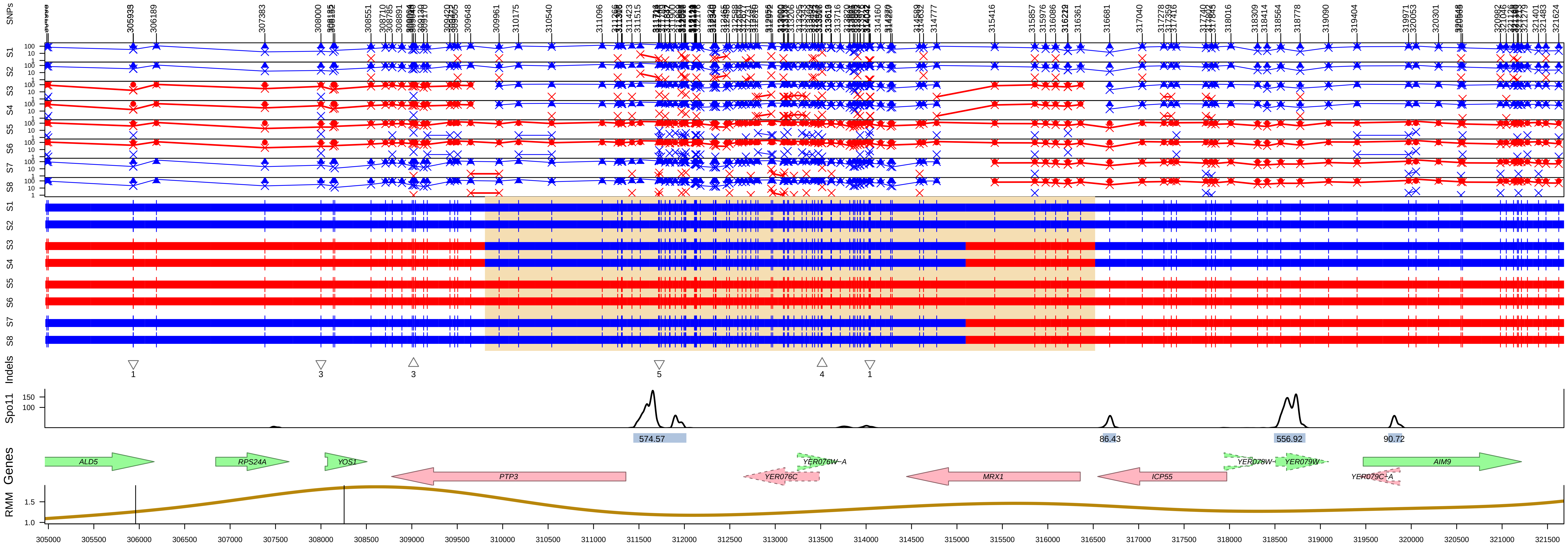

Genotype=TCMN6 Id=27 Chromo=5 Type=One−Sided Bidirectional
Estimated: DSBs=1, COs=1, NCOs=0
Pattern=(2:6)_(6:2)_(4:4aCO)
Mid Len=6716bp Midpoint=313162.75 Variants=78
Red=S288c, Blue=SK1

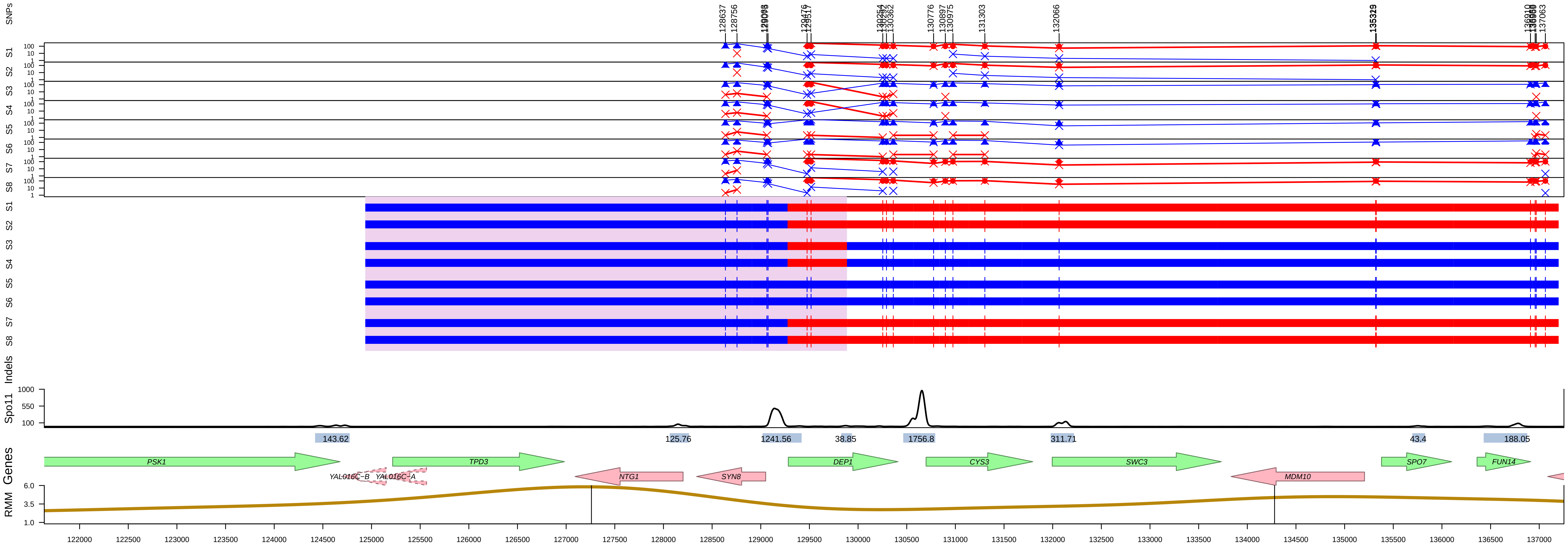

Genotype=TCMN6 Id=1 Chromo=1 Type=dNCO
Estimated: DSBs=2, COs=0, NCOs=2
Pattern=(0:8)_(6:2)_(4:4)
Mid Len=4950bp Midpoint=127410.75 Variants=6
Red=S288c, Blue=SK1

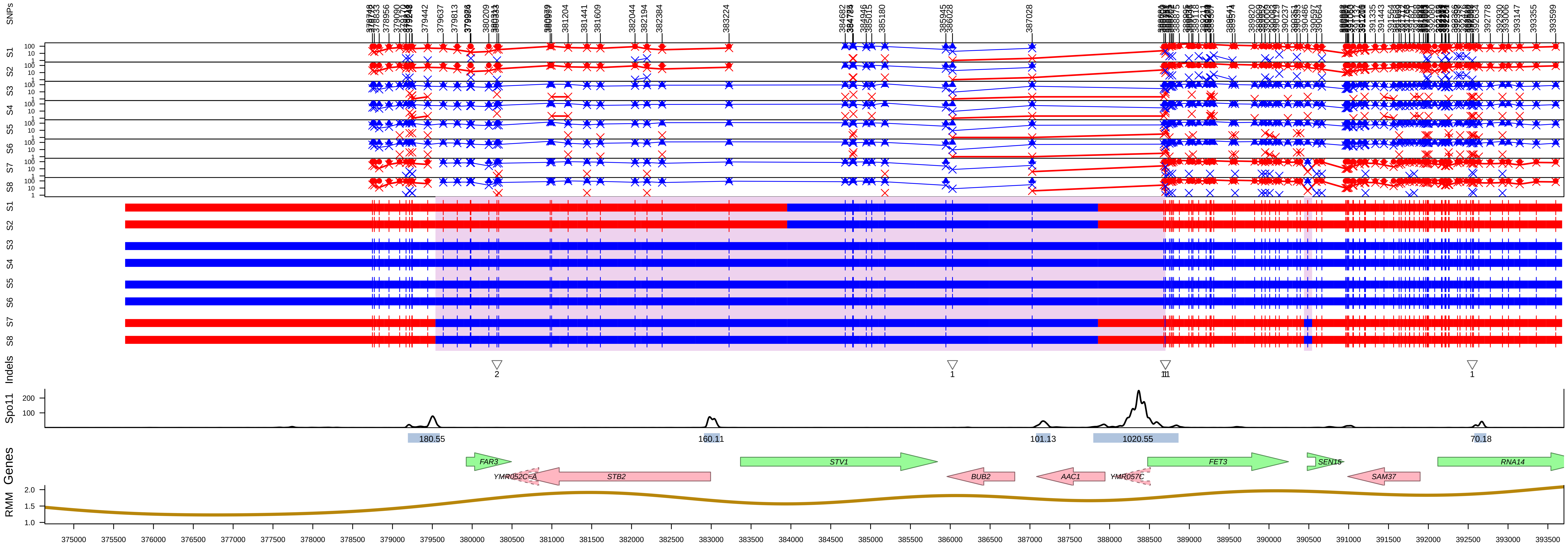

Genotype=TCMN6 Id=85 Chromo=13 Type=dNCO
Estimated: DSBs=2, COs=0, NCOs=2
Pattern=(2:6)_(0:8)_(4:4)_(2:6)_(4:4)
Mid Len=9162bp Midpoint=384120.25 Variants=26
Red=S288c, Blue=SK1

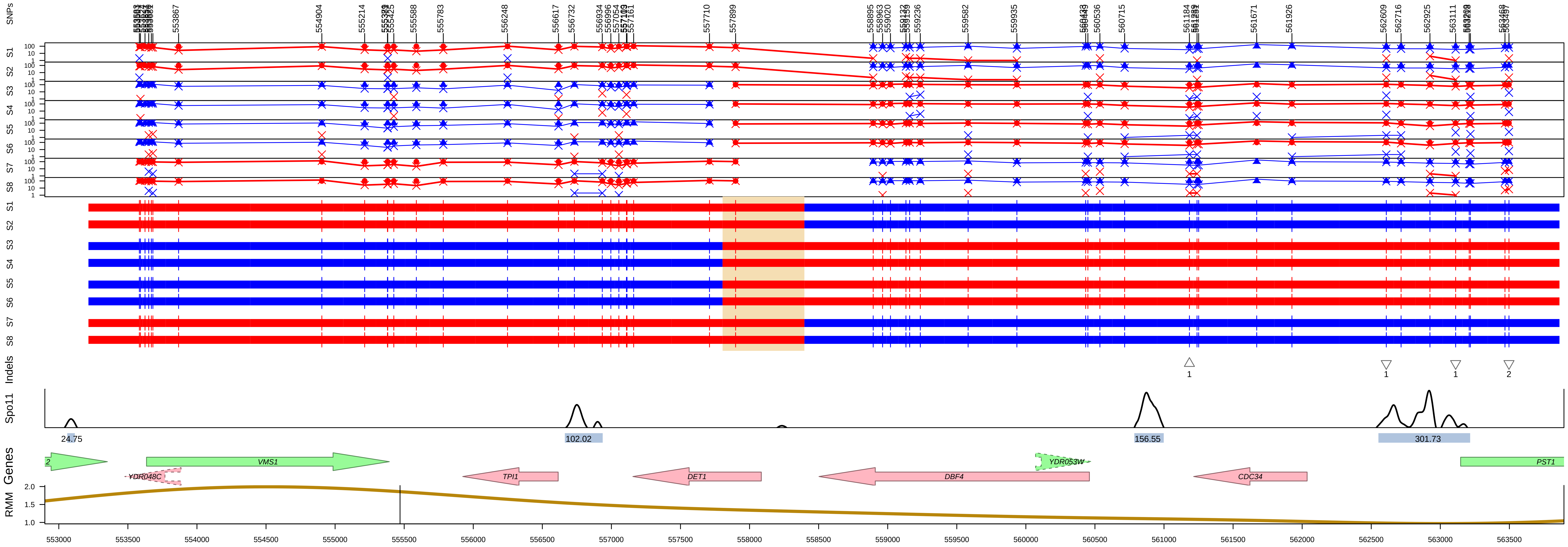

Genotype=TCMN6 Id=23 Chromo=4 Type=dCO
Estimated: DSBs=2, COs=2, NCOs=0
Pattern=(8:0)_(4:4aCO)
Mid Len=592bp Midpoint=558100.75 Variants=1
Red=S288c, Blue=SK1

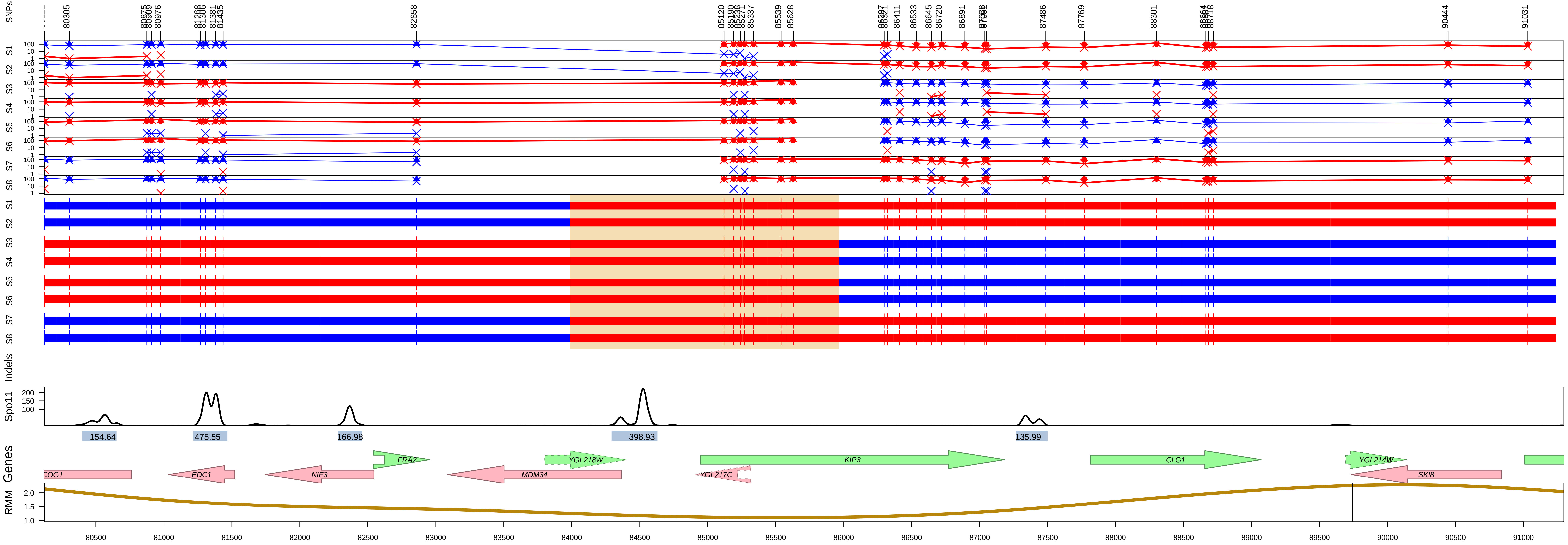

Genotype=TCMN6 Id=36 Chromo=7 Type=dCO
Estimated: DSBs=2, COs=2, NCOs=0
Pattern=(8:0)_(4:4aCO)
Mid Len=1974bp Midpoint=84975.75 Variants=7
Red=S288c, Blue=SK1

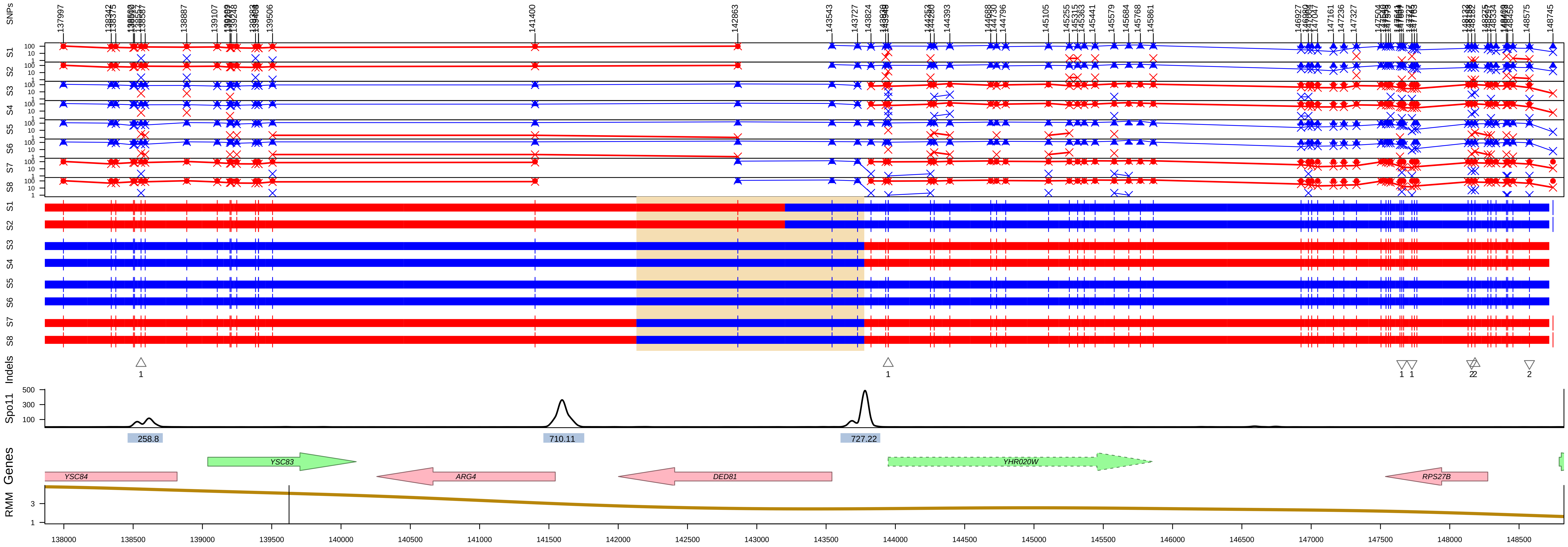

Genotype=TCMN6 Id=50 Chromo=8 Type=>2 Chr CO
Estimated: DSBs=2, COs=1, NCOs=1
Pattern=(2:6)_(0:8)_(4:4aCO)
Mid Len=1644bp Midpoint=142953.5 Variants=3
Red=S288c, Blue=SK1

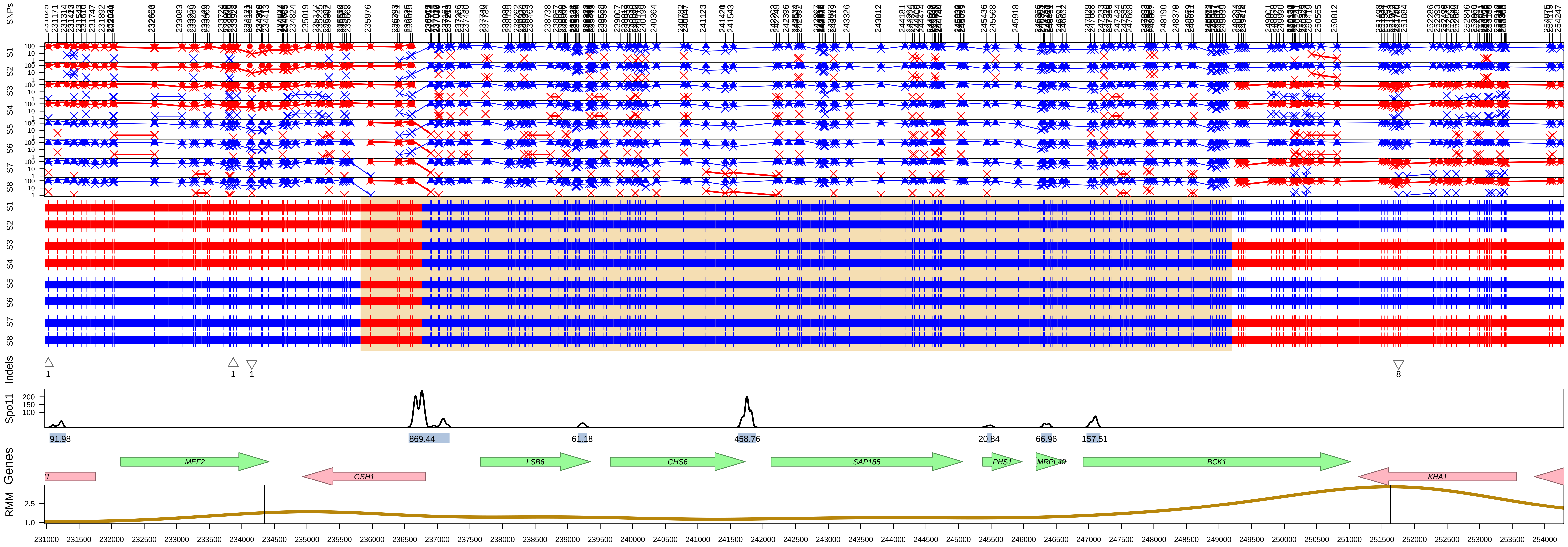

Genotype=TCMN6 Id=62 Chromo=10 Type=>2 Chr CO
Estimated: DSBs=2, COs=1, NCOs=1
Pattern=(8:0)_(0:8)_(4:4aCO)
Mid Len=13374bp Midpoint=242509.25 Variants=124
Red=S288c, Blue=SK1

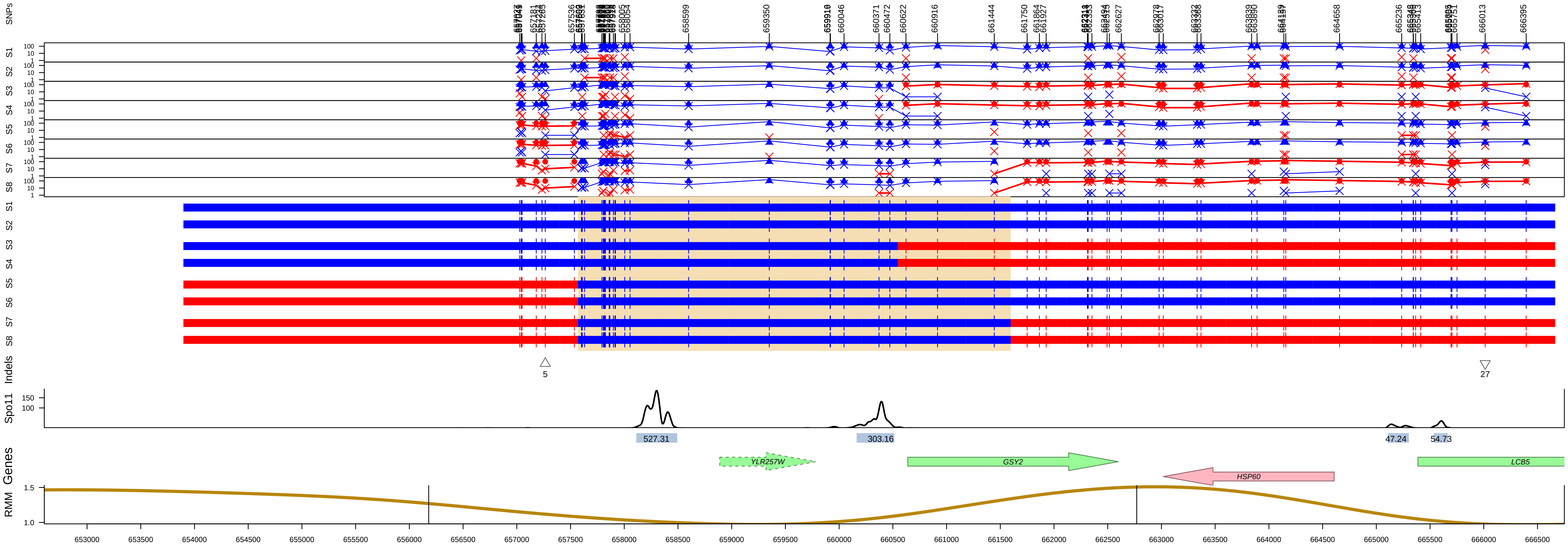

Genotype=TCMN6 Id=79 Chromo=12 Type=>2 Chr CO
Estimated: DSBs=2, COs=1, NCOs=1
Pattern=(0:8)_(2:6)_(4:4aCO)
Mid Len=4028bp Midpoint=659583 Variants=27
Red=S288c, Blue=SK1

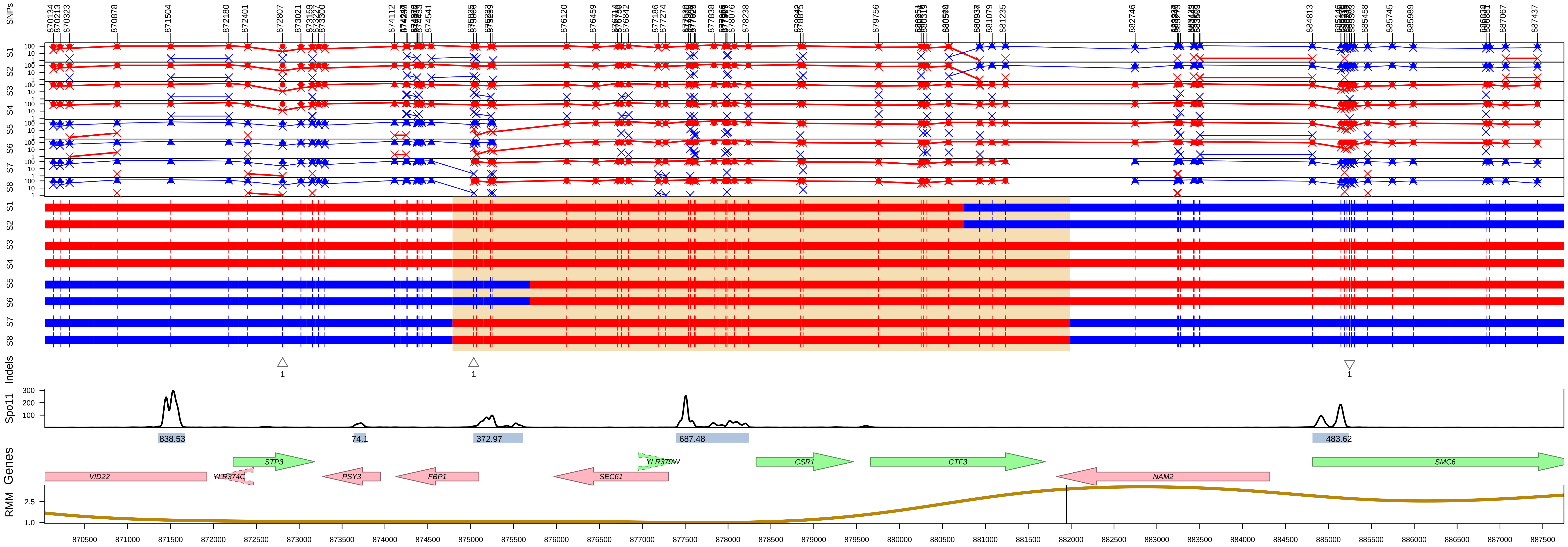

Genotype=TCMN6 Id=82 Chromo=12 Type=>2 Chr CO
Estimated: DSBs=2, COs=1, NCOs=1
Pattern=(6:2)_(8:0)_(6:2a)_(4:4aCO)
Mid Len=7202bp Midpoint=878389.25 Variants=35
Red=S288c, Blue=SK1

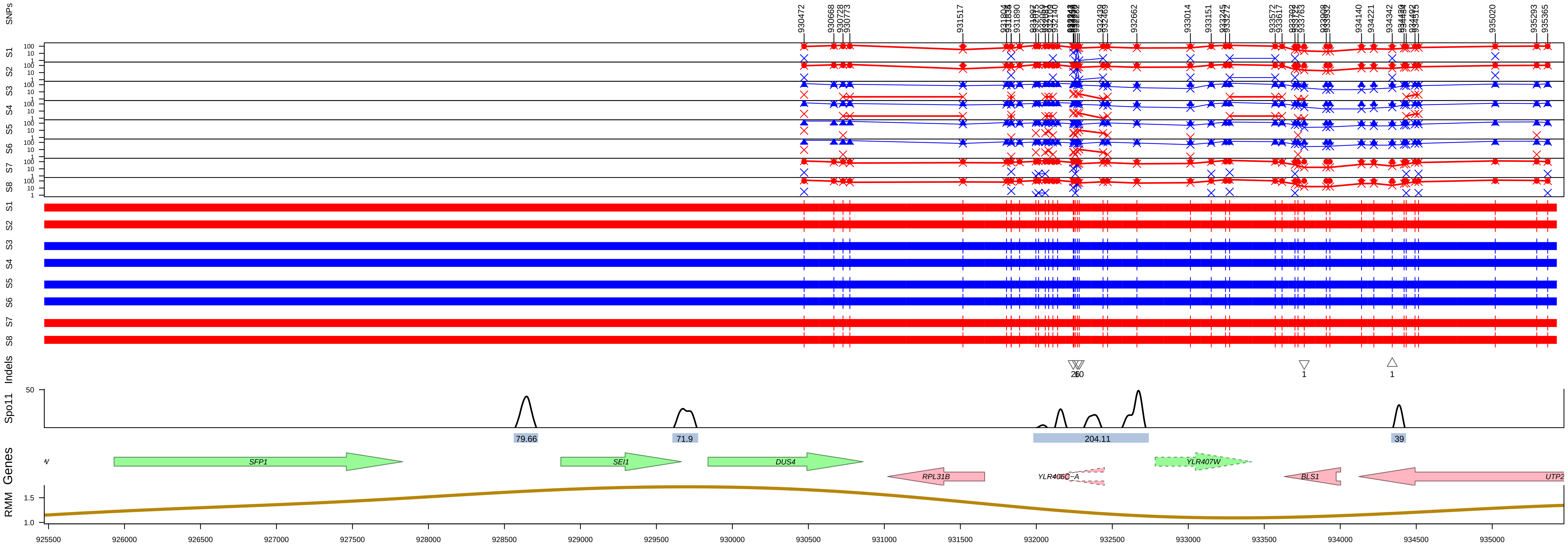

Genotype=TCMN6 Id=83 Chromo=12 Type=dCO
Estimated: DSBs=2, COs=2, NCOs=0
Pattern=(4:4aCO)
Mid Len=19018bp Midpoint=911454 Variants=0
Red=S288c, Blue=SK1

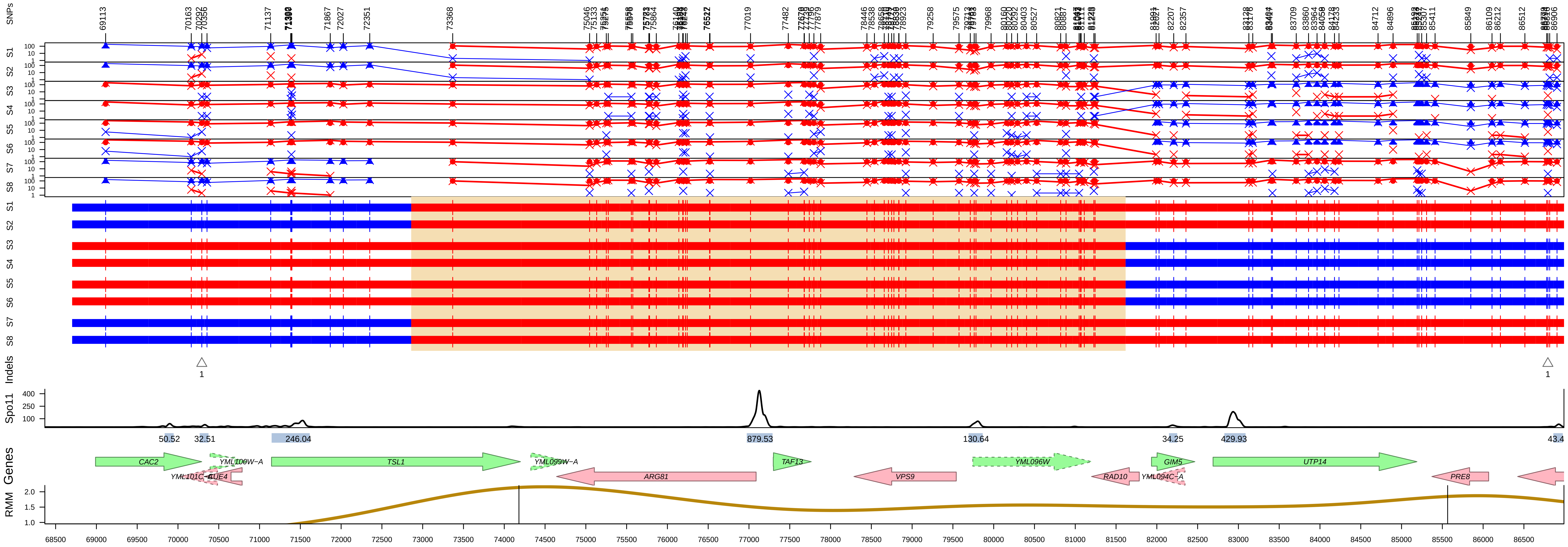

Genotype=TCMN6 Id=84 Chromo=13 Type=dCO
Estimated: DSBs=2, COs=2, NCOs=0
Pattern=(8:0)_(4:4aCO)
Mid Len=8758bp Midpoint=77238.25 Variants=52
Red=S288c, Blue=SK1

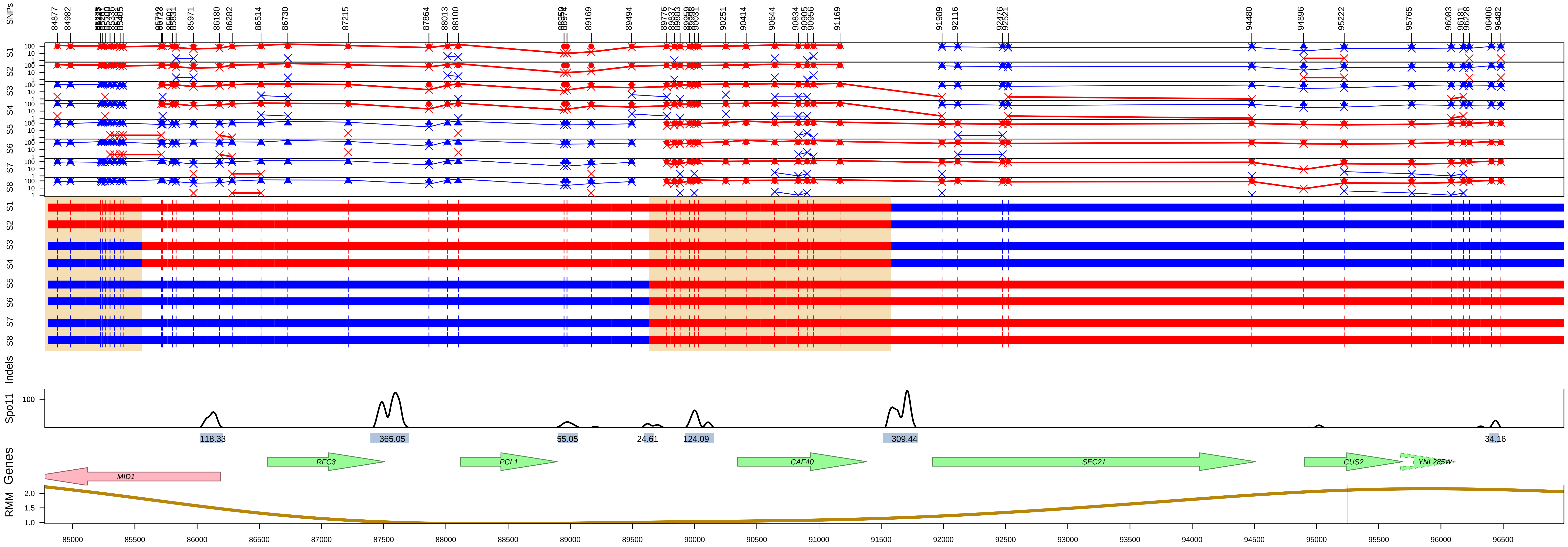

Genotype=TCMN6 Id=95 Chromo=14 Type=dCO
Estimated: DSBs=2, COs=2, NCOs=0
Pattern=(8:0)_(4:4aCO)
Mid Len=1944bp Midpoint=90607 Variants=13
Red=S288c, Blue=SK1

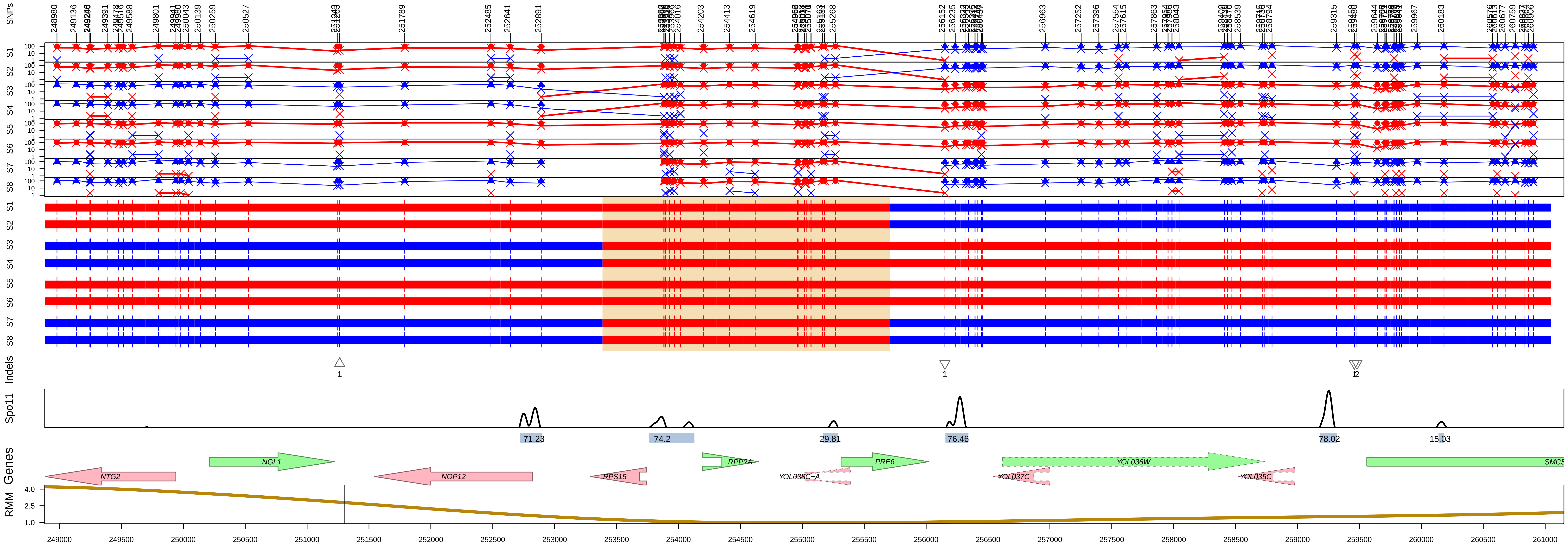

Genotype=TCMN6 Id=112 Chromo=15 Type=dCO
Estimated: DSBs=2, COs=2, NCOs=0
Pattern=(8:0)_(4:4aCO)
Mid Len=2324bp Midpoint=254548.25 Variants=19
Red=S288c, Blue=SK1

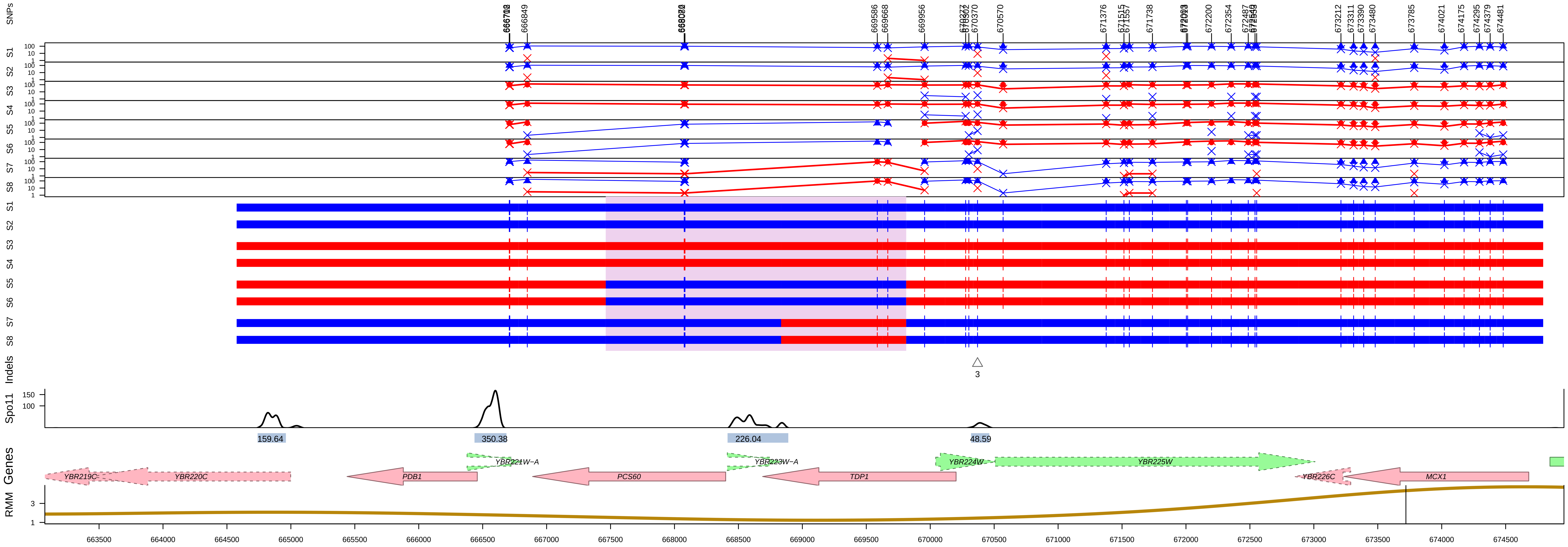

Genotype=TCMN6 Id=13 Chromo=2 Type=dNCO
Estimated: DSBs=2, COs=0, NCOs=2
Pattern=(2:6)_(4:4aCO)_(4:4CO)
Mid Len=2350bp Midpoint=668637.25 Variants=4
Red=S288c, Blue=SK1

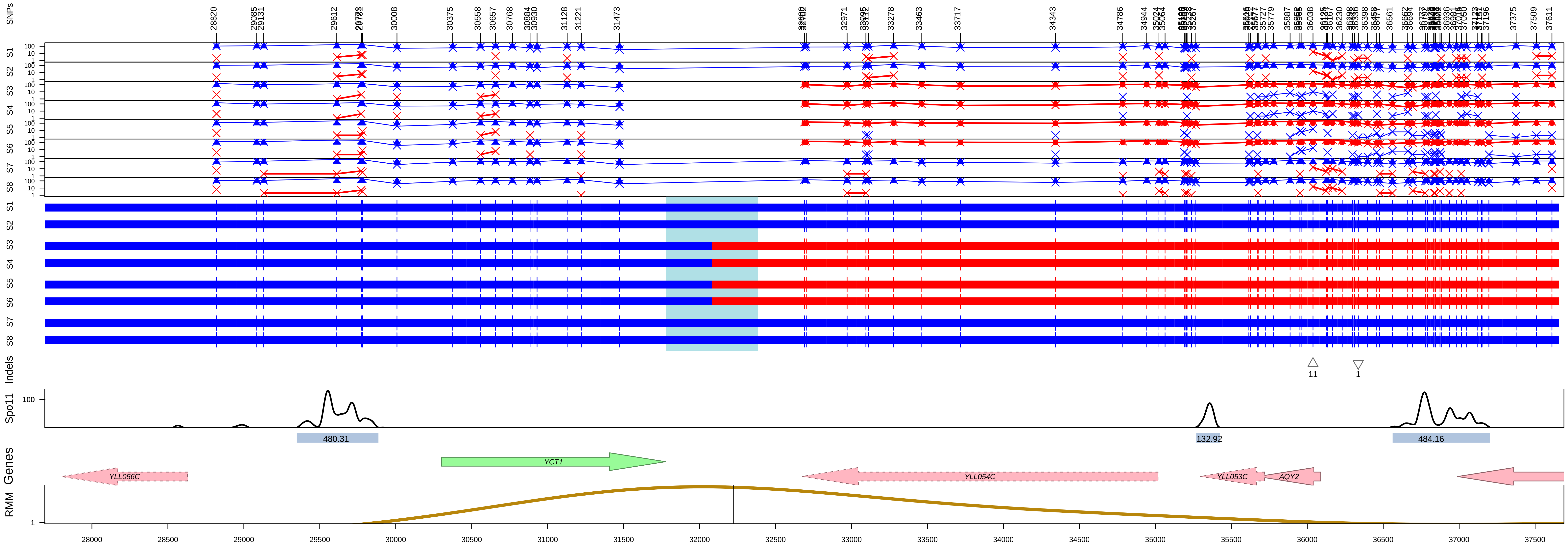

Genotype=TCMN6 Id=210 Chromo=12 Type=Unknown
Estimated: DSBs=1, COs=U, NCOs=U
Pattern=
Mid Len=608bp Midpoint=32081.5 Variants=0
Red=S288c, Blue=SK1
