# Supplementary material for: Separable roles of the DNA damage response kinase Mec1ATR and its activator Rad24RAD17 during meiotic recombination
Source: PLoS Genet. 2024 Dec 9;20(12):e1011485. doi: 10.1371/journal.pgen.1011485 (PMC11658708; doi:10.1371/journal.pgen.1011485)
Supplement: Attachment — Submitted filename: 2024 PLOS Genetics reviewer responses Mec1 paper.pdf [file pgen.1011485.s023.pdf]

Author responses to reviewer comments: PGENETICS-D-24-00817

Reviewer comments are in black
Author responses are in blue
Line numbers refer to version without tracked changes

AE's comments:
L26 "and has less effect on spore viability"

We have corrected this oversight (line 26)

L213-216 Almost all of the skew is in the first 40kb from the telomere, whereas my impression is that EARs are substantially larger. Is the increase uniform across chromosomes, or is it possible that a few chromosomes are dominating the signal? Perhaps this is a subtelomere thing, not an EAR thing?

Thank you for drawing these points to our attention. Whilst previously characterised effects at EARs may indeed span beyond 40 kb, the strength of such effects (for example in average Hop1 binding) are strongest at ~40 kb from the telomere (Subramanian et al 2019, Fig 2b and 2d; attached). We have rephrased this section to weaken the assertion that it is specifically driven by EARs and not an independent effect of chromosome ends (Lines 216-218).

https://www.ncbi.nlm.nih.gov/pubmed/30814509

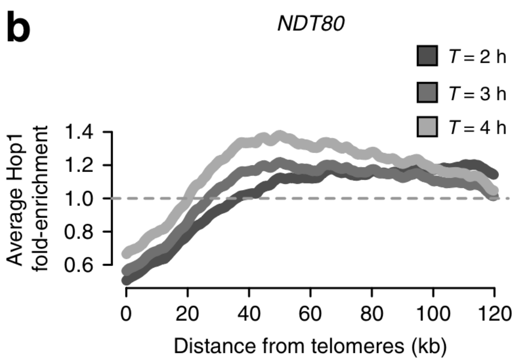

Secondly, we have now included an additional supplementary figure (Fig S4) that tests for any chromosome-specific effects. Notably, we observe highly reproducible effects at all chromosome ends in any particular genotype, indicating that the differences are not driven by any particular chromosome. We have included this point in the revised text (Lines 219-222 and Lines 259-262).

L1375 and ff please proof the reference list for capitalization and italicization uniformity

References are imported by our reference manager from PubMed, but this does not seem to include italics information. We have manually amended these.

Table S1 The spore viability distributions don't add up to 100%. What do these numbers represent? This table would benefit from a more extensive legend.

Thank you for pointing out this error which arose in the viability pattern numbers during preparation of the aggregated table. All numbers errors have been corrected (all now add up to 100% as expected). We have also updated the legend as suggested (Line 1386-1396).

Figures general In Table S1, viability is reported for several arrest periods for ndt80AR. Which of this arrest periods were used for the mapping experiments? This should be stated in figure legends or in a prominent place in the results section.

We have added this information (8 hours) to the legend of Table S1, and to the Methods (Line 1396 and 908).

Statistics In addition to stating what error bars represent, please indicate in each figure legend how many replicates were performed when error bars indicate standard error. If < 3 replicates were performed, then error bars should denote range.

We have made the suggested adjustments. In instances where we do not provide error bars, we have plotted the individual data points on top of the bars.

Reviewer #1: The edits to the manuscript have largely addressed the issues I raised in my initial review. The problems with ascertainment bias obviously persist but the authors were very careful in highlighting the associated caveats throughout the manuscript.

We thank the reviewer for their supportive comments and are pleased we have addressed their original concerns.

Reading through the revised manuscript, I only noticed a couple of things:

1. One item that needs to be addressed is missing statistical analyses. In particular the results shown in Figure 2 are discussed at length regarding increases and decreases but, at least in some of the panels (e.g. 2C), it is not clear how significant these differences are. In addition, in figures where statistical analyses were performed (e.g. 4, 5…), p value thresholds should be corrected for multiple hypothesis testing. If such correction was already performed, please indicate it in the methods and the figure legends.

We have added P values in the text for the results of the tests performed in figure 2 (Lines 199, 203, 204 and 212).

In the process of recalculating P values, we noticed errors in the statistical tests for differences between quantiles of event lengths (figures 5, 8, S7). We apologise for this error and have corrected the tests. Notably, the difference in NCO lengths between wild type and *ndt80AR* is no longer significant, but differences in CO lengths remain significant. This has been noted on line 445.

We have corrected for multiple testing using the Benjamini-Hochberg method. The results of our analyses do not qualitatively change after correction. As requested, we have noted the use of

such correction in the methods section (Lines 1113-1114) and in the legends of relevant figures

2. The other thing I was wondering about is the increased cell-to-cell variation, particularly for COs for some of the mutants. The authors propose that this heterogeneity may be related to asynchronous meiotic induction (line 200-202). I wonder whether the variation may instead be related to temporal differences in homolog engagement/chromosome synapsis among chromosomes. This seems testable because one would expect that excess COs will be non-randomly distributed among chromosomes, with some chromosomes receiving substantially more COs than others, but the affected chromosomes would differ from cell to cell. The author note later in the text that E0 chromosomes also lack detectable NCOs, which would seem consistent with such chromosome-by-chromosome effects. If detected, these effects would also impact the conclusions about the randomization of inter-crossover distances in Figure 7.

1. This is an interesting question. To test this idea, we initially looked at the variation in CO density per chromosome. CO densities were normalised by dividing by the total number of COs per meiosis in order to allow variances to be directly compared, and then plotted against the rank order of chromosome size. This demonstrated significant variation between chromosomes, with shorter chromosomes generally displaying both greater densities and potentially greater variance, which appeared greater in some genotypes (e.g. *mec1-mn msh2*Δ).

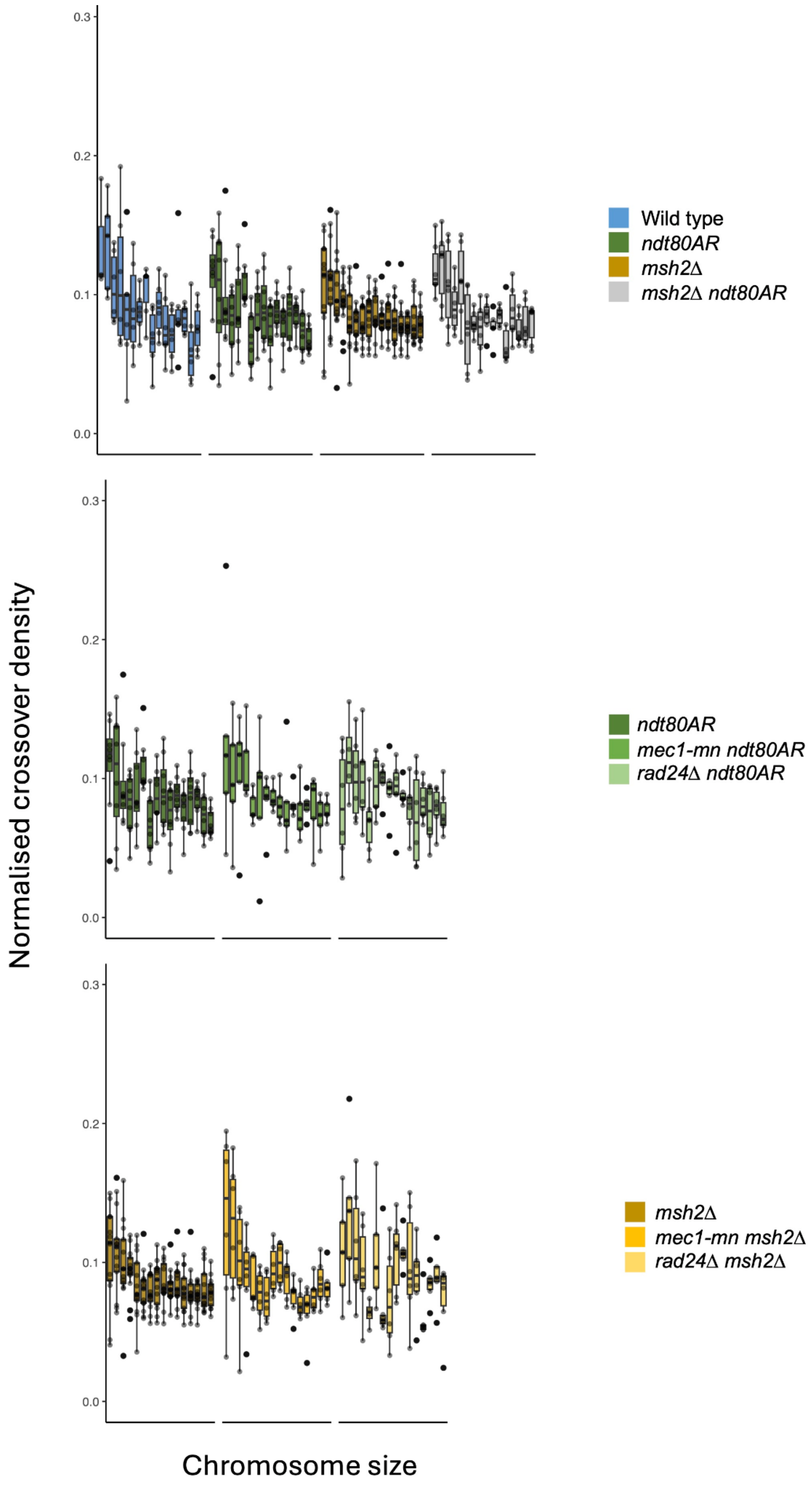

Normalised crossover density
0.3
0.2
0.1
0.0
Wild type
ndt80AR
msh2Δ
msh2Δ ndt80AR
ndt80AR
mec1-mn ndt80AR
rad24Δ ndt80AR
msh2Δ
mec1-mn msh2Δ
rad24Δ msh2Δ
Chromosome size

2. To simplify this analysis we summarised these variances by plotting variance per chromosome in each genotype. Although differences between genotypes are present, it is difficult to determine what is driving these effects genetically because, even though we have normalised for the number of COs, it seems probable that variation in CO number may still be influencing variation in CO densities due to sampling-type effects.

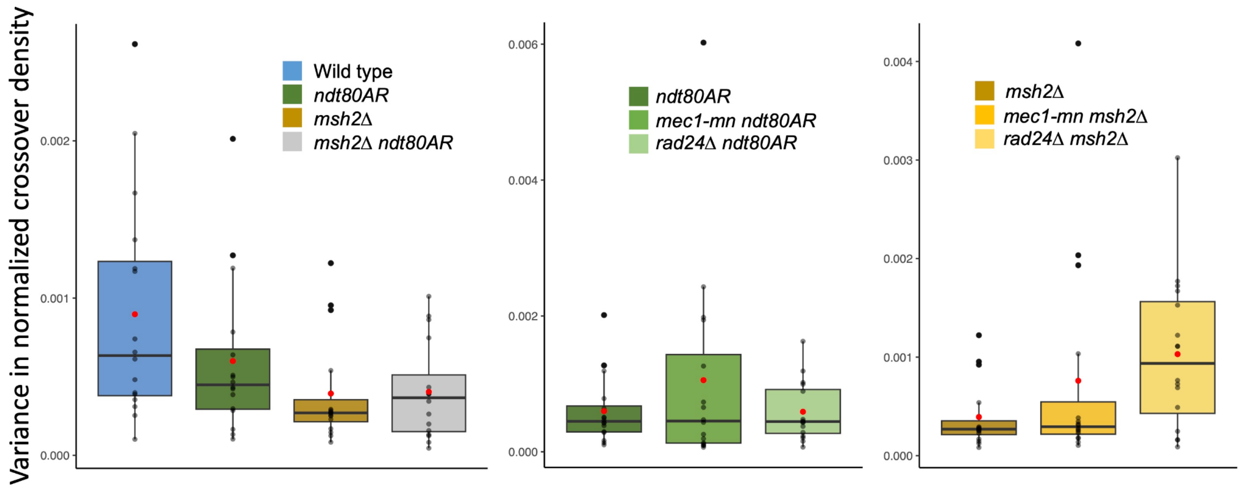

3. Finally, to directly investigate if variations in CO number influence the degree of variance in CO density between chromosomes, we plotted variance in densities against CO number and stratified by genotype. No clear trend was observed, although some genotypes did appear to display a weak negative trend, as might be expected if increased CO number reduces variance in CO density (contrary to the reviewer's suggestion).

Upon fitting linear models, as expected from our visual inspection, we found relatively low values of R-squared, suggesting that crossover number does not substantially contribute to variance in CO density. Moreover, the P values for such models were consistently above the threshold for significance, so we cannot confidently conclude that there is a relationship between the number of COs and variance in CO density across chromosomes. In the one instance where correlation was significant (*msh2 ndt80AR, P* = 0.0489), the correlation was negative (slope = -0.0016), which is counter to the reviewer's suggestion.

What the reviewer suggests is possible, however our data do not support this. Further replicate datasets would be necessary to test this relationship more thoroughly given the very weak potential relationships observed. We have included these analyses in this rebuttal for information, but do not consider it necessary to further expand the manuscript by their inclusion.

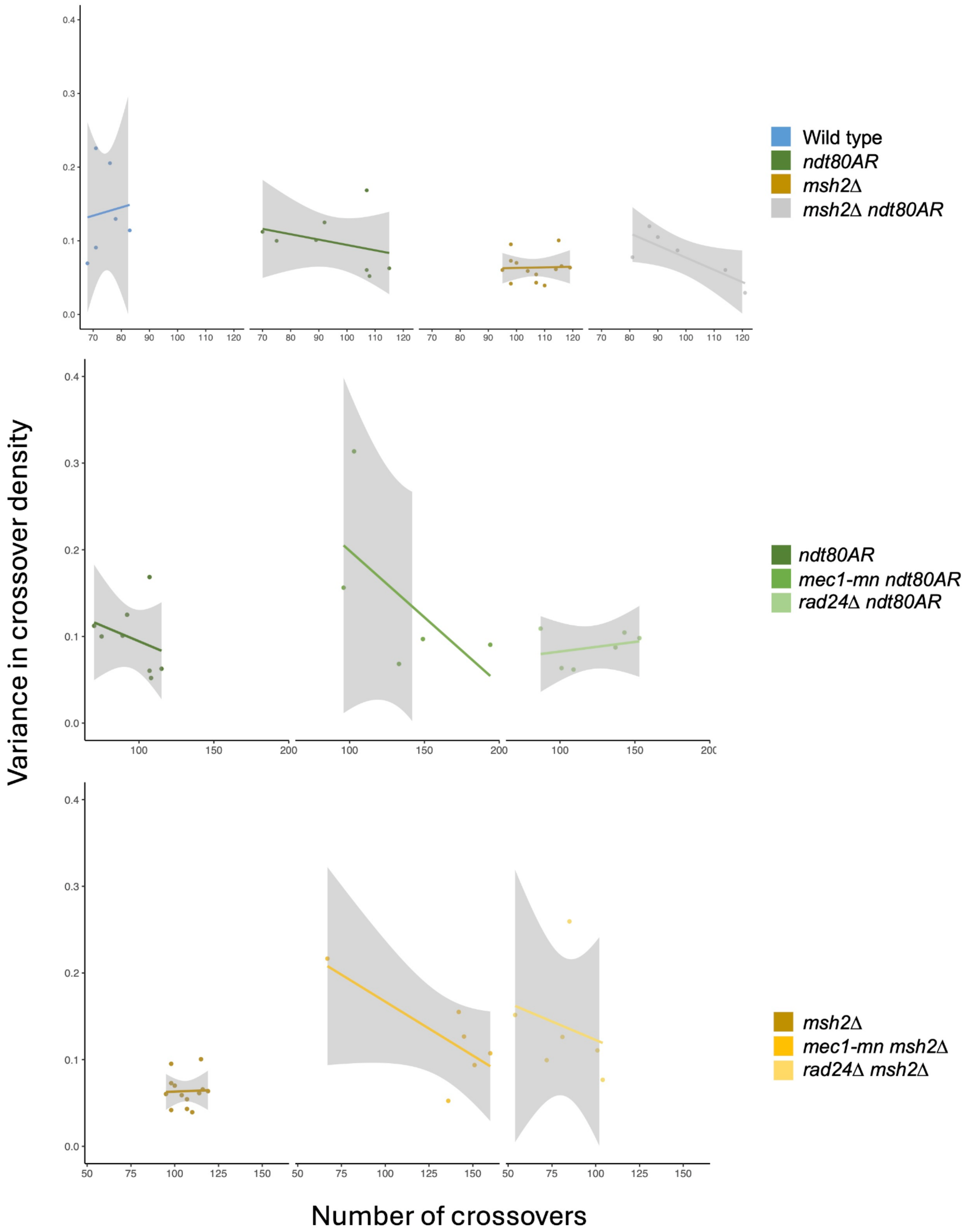

Wild type
ndt80AR
msh2Δ
msh2Δ ndt80AR
ndt80AR
mec1-mn ndt80AR
rad24Δ ndt80AR
msh2Δ
mec1-mn msh2Δ
rad24Δ msh2Δ
Variance in crossover density
Number of crossovers

Reviewer #2: The authors have thoroughly addressed the concerns by reviewers and associate editor. I have only some minor points that the authors may want to fix:
L160: normal prophase I length

We have made this correction (Line 161).

Fig. 3A: Could you please move the legend into Fig 3A, so it is closer to the dots? Direct genotype labeling of the dots with increased E0 chromosomes might be an alternative. As it stands now, it is really challenging for the reader to find the right label in the list, even there are only 4-5 genotypes that result in increased E0 events.

We have moved the legend, placing it between Fig 3A and B

L407: isn't dHJ migration a subset of JM migration? Maybe: "JM migration, including, but not limited to dHJ branch migration"

We have made this adjustment (Lines 424-425)

L513 & L526 & L724: Direct evidence for the role of Mec1 in homolog bias was provided by Joshi et al (2015; PMID: 25661491), and should be cited here. Synergistic effects of Mec1 and Pch2 on homolog bias further address the issue of redundancy in Hop1 activation (see L724).

We have added the suggested reference and adjusted the text in Line 531. We have also added this point to the discussion (Lines 751-753).

Fig. 7A-E: Please bring the legend (Simulation/Experimental) further down into the Figure and increase the font size. Reading on the screen, it's easy for the legend to get cut off, and this reader thought they have to figure out for themselves what red and blue curves stand for.

We have made the suggested adjustments (Fig 7)

L576: Brown et al (2013; PMID: 23316435) should be cited which also reported the surprising lack of mlh3D effects on spore viability in an mms4 mutant background.

We have adjusted the text on Lines 596-597 and added the suggested citation

L605: Please rewrite this rather convoluted sentence.

We have rewritten this sentence (Lines 628-630)

Fig. 8E,F: Please refer the reader to the appropriate (supplemental) diagram for explanation of the y-axes: "Average 2-nonsis NCO per meiosis" and "% of NCOs that are 2-nonsis". "2-nonsis" is not explained anywhere in the main manuscript, so only the true aficionados will be able to understand this figure.

We have referenced the appropriate supplementary figures in the legend for figure 8.

L654: Please improve readability of this sentence: “due to increase…due to failure”

We have rewritten this sentence (Lines 682-684)

Reviewer #3: To clarify each function of the 9-1-1 clamp loader Rad24 and Mec1ATR, which are involved in DNA damage response, in controlling the meiotic recombination processes, the authors used a novel approach, deep sequencing of tetrads or octads after PMS in spores derived from S288C/SK1 hybrid diploids and in a mismatch repair-deficient background. Although many of the results merely obtained confirm those already reported, the novel methodology described above is interesting, even if there is room for further improvement, and is expected to provide valuable insights that will provide opportunities for future applications in various research fields.

On the other hand, there have already been many reports showing that mismatch repair deficiency has a significant impact on the biochemical reaction of homologous recombination. Furthermore, this paper does not fully explain what occurs during meiotic recombination in the msh2Δ strain. Therefore, previously, I pointed out that the results obtained by meiotic recombination in the genetic background of msh2Δ do not necessarily coincide with what actually occurs in wild-type strains, and that the experimental results should be interpreted with caution. In this revision, this point is mentioned more appropriately in the text, and it has been improved. However, since the significant increase in spore viability of mec1-mn and rad24Δ strains in the msh2Δ background was accompanied by large alternations in the CO/NCO balance, we should avoid making detailed mention of the CO control analysis (Fig. 3), which used these CO/NCO ratios as parameters. Above all, this paper shows a specific contribution of MSH2 to CO interference (L549-551), which may lead to a recombination reaction that differs from that in the wild-type strain. Furthermore, the data from the rad24Δ sml1Δ strain, which represents a sample in which the six chromosomes are miraculously precisely distributed during meiosis I without COs, raises serious concerns regarding the generalizability of the observations. As a result, the paper, especially the first half, requires assumptions to interpret the data, making it difficult to generalize and understand what the data truly shows.

On the other hand, this method has successfully analyzed unusual meiotic recombination derived from multiple DSBs and DSB resection tract length in the DDR mutants. I agree the importance of making the data obtained public, but since making definitive claims of novel ideas based on data whose reproducibility has not been confirmed can lead to confusion. Authors should separate clear conclusions based on the experimental results from uncertain interpretations based on assumptions and consider keeping discussion of the latter more modest and simpler, or excluding discussion of data that is difficult to generalize to know the function of DDR factors in meiotic recombination.

We appreciate the considered appraisal of our revised study. We have sought to address these concerns in our responses to the points highlighted below.

Major points:
1. L240-242: Rather than mec1 deficiency resulting in greater recombination diversity, could this result be due to the possibility that tetrads selected for viability in mec1-mn cells survive by various means?

Whilst this may be possible, this sentence only describes the change we see in the data relative to the control and avoids unnecessary speculation for which we have no evidence.

2. L245-248: Although the statistical significance of the difference is unclear, at least in the mec1-nm ndt80AR cells, recombination near the telomere appears to be higher than in the wild type. Also, in rad24Δ, recombination appears to be higher than in the wild type up to 40 kb from the telomeres (Fig. S3C). Please check the data. Therefore, the conclusion is an overstatement, I think.

We have revised this section and the analysis of the dataset to now include both telomere ends and to stratify by chromosome (Fig S4 as suggested by the AE). The similarity between what happens at each chromosome end within a given genotype gives us confidence that these effects are real, and that *rad24*Δ and *mec1-mn* behave differently with respect to the relative distribution of recombination towards chromosome ends. Specifically, Mec1 appears to have only a minor role in the enhancement of recombination that occurs towards chromosome ends upon prophase extension, whereas Rad24 has a larger role that is independent of prophase extension (Lines 253-259).

While it is the case that *rad24*Δ *ndt80AR* has higher recombination than the wild type at up to 40 kb from chromosome ends, we infer that this is due to the effect of prophase extension which increases recombination at telomere ends independently of Rad24.

3. L253-254: I don't understand the logic behind this conclusion. In the ndt80AR strain, Ndt80 is ectopically expressed at a later time than usual, so Mec1-dependent inhibition of Ndt80 does not occur at the time of Ndt80 recovery as in the wild-type. (Are there any data showing that inhibition occurs in the ndt80AR?) Rather, since the recombination frequency is even higher in the ndt80AR mec1-mn than in the ndt80AR, a function of Mec1 that is not dependent on Ndt80 should be considered.

We have clarified this section to avoid confusion (Lines 266-271). We agree that our data support a role for Mec1 (in DSB trans interference) that is independent of Ndt80.

4. L318-320: Isn't this explanation only valid under the assumption that the amount of DSBs in each strain is constant? The definition of CO homeostasis is that the frequency of COs per bivalent remains constant despite fluctuations in the amount of DSBs. Since NCO varies with the number of DSBs [1], please provide evidence that the amount of CO per NCO can be used as a criterion for CO homeostasis even when the amount of DSBs is not constant among the strains. At least, as you have discussed, there is a possibility that bias is being applied at the time of selecting surviving spores in rad24Δ. In other words, it is thought that complex events are occurring in each mutant background, so unless the analysis is performed in combination with the spo11 hypomorph mutations, it is difficult to simply consider the function of DDR factors in CO homeostasis with this method.

We assume that the sum of COs and NCOs is proportional to the number of total DSBs in each meiosis, and therefore the ratio of COs to NCOs is representative of CO homeostasis. We have

made an additional clarification of this in the text (Line 333). Such an assumption does not rely on the number of DSBs being constant. In fact our conclusions rely on the total CO+NCO to vary in order to observe these negative trends.

5. L549-551: More even spacing than in the wild type? What specific situation are you thinking of?

We have clarified that this is in comparison to the wild type (Line 569).

6. L633-634: Although its relationship with ZIP3 is still unclear, RAD24 has been reported to function upstream of ZIP1 in meiotic recombination. The rad24 mutation may bypass ZIP3 function upstream [2]. Analysis of the zip3 rad24 msh2 triple mutant is required to reach this conclusion.

We have acknowledged this point (Lines 659-661).

Minor points:
7. L233: Please briefly explain the rationale for using the sml1Δ mutation.

The purpose of using the *sml1*Δ mutation was to increase spore viability in the *rad24*Δ strain.

This was noted on Line 154.
