# Supplementary material for: Separable roles of the DNA damage response kinase Mec1ATR and its activator Rad24RAD17 during meiotic recombination
Source: PLoS Genet. 2024 Dec 9;20(12):e1011485. doi: 10.1371/journal.pgen.1011485 (PMC11658708; doi:10.1371/journal.pgen.1011485)
Supplement: Attachment — Submitted filename: 2018 PLoS Genetics Reviewer Comments.pdf [file pgen.1011485.s022.pdf]

Reviewer/Editor comments in **BLACK**, author responses in **BLUE**.

Associate Editor's comments:

1. Most important, the issue of bias due to selection needs to be addressed. As pointed out by reviewer #1, there is a real concern that the selection for complete tetrads, especially when crossover frequencies are low and/or the population is non-uniform, could distort measured outcomes, especially with regards to "class 1" versus "class 2" crossovers, interference, etc. For example, zip4 mutants were reported to display negative interference (Tsubouchi/Roeder, 2006)--later shown to be spurious when coincidence between unlinked intervals on nonhomologous chromosomes was shown also to display negative interference (Chen/Fung 2008). This is also a concern, to my mind, when cells are held at pachytene for varying amounts of time, such as is likely to occur with the NDT80-AR allele. My reasoning is that, in a standard liquid sporulation, even SK1/SK1 cells initiate meiosis over a ca. 2 hour window, and thus some cells will be accumulating crossovers for as much as 2h longer than others at the time of release from pachytene arrest. This may also be the case if there is a greater-than-normal variance in the duration of meiosis I prophase in rad24 or mec1 mutants. In both cases, selection for 4 viable spores may end up selecting for cells that have spent more time in meiosis I prophase, and thus have accumulated more DSBs and potentially altered class1:class2 crossover levels, for example. Alternatively, (as I think you mention), loss of DNA damage response may result in a loss of homeostatic constraints on DSB levels and spacing, which in turn may lead to greater variance in the number of events per cell, etc. etc.

I don't have any easy suggestions for addressing these issues, but one way to start (at least to evaluate how much of an issue it is) would be to present the tetrad data in toto--in other words, spore viability patterns as well as numbers; thus, my previous request. At the very least, this should allow the reviewers/readers to evaluate for themselves how much of a concern this is. In cases where spore viability is truly bad, you might want to consider sequencing a reasonable number of spore colonies chosen randomly from tetrads where there were *not* four viable spores, to ascertain whether or not the four spore-viable tetrads are exceptional with regards to crossover numbers. Regardless of whether they are or are not, I think that a more nuanced interpretation of crossover numbers, crossover types, and crossover spacing may be called for, one that more completely incorporates the uncertainties articulated above. I want to emphasize that I personally think that these concerns really do have the potential to compromise the certainty of conclusions, and call for being more cautious in their interpretation.

(BTW, you might want to think about this with regards your recent bioRxiv manuscript on mismatch correction and crossover interference, but that is not the consideration here.)

We thank the editor for raising these important points and allowing us an opportunity to address them, which we have tackled in 3 ways: 1) As suggested, we have provided a table of the spore viability patterns for each strain (Table S1). 2) We have added an emphasised section at the beginning of the results section to make it clear that there are likely to be observational biases

introduced due to the selection for rare 4-spore viable meioses (Page 5). 3) To better display the variability between meioses that the editor discusses, we have revised Figure 2 so that in addition to presenting averages, the individual frequencies of COs and NCOs are presented for each meiosis (as grey dots). We also included a stacked bar here so that the relative fraction of “complex” events can be compared between mutants and controls. These values for each meiosis are also represented in a new summary table (Table S2).

2. One should keep in mind that msh2Δ mutants abrogate at least three activities, each of which could have impact on COs, NCOs, multiple events, spacing, etc. etc. Please forgive me if the following is obvious to you. First, loss of MMR not only limits the "destruction" of heteroduplex, but also reduces generation of recombinogenic lesions by colliding MMR excision tracts or ssDNA within single excision tracts (see Borts and Haber, 1987, 1990. Second, abrogation of mismatch recognition will reduce heteroduplex rejection; "rejected" events that would form IH COs could be recycled into NCOs, intersister events, or even class 2 COs—thus, reducing rejection could change the overall spectrum of events observed in hybrid crosses. Finally, Msh2 also participates in flap cleavage (with Rad1/Rad10, Msh3, Saw1 etc.) that is mismatch recognition-independent (and can be separated by msh2 separation-of-function alleles from MMR), and we really don't know to what extent Msh2-dependent flap-cleavage shapes the recombination landscape, even in completely homozygous non-hybrid strains. Nothing to be done about this experimentally, but again it would be useful if the presentation and discussion of the impact of msh2 mutation were a bit more nuanced in acknowledging the potential contribution of all 3 of these.

As suggested, we have expanded the discussion of the known roles of Msh2 during HR on page 7.

3. Code. This one is unambiguous: PLOS editorial policy requires that all underlying data and code be deposited in an independent, openly-accessible public repository (i.e. GitHub, Figshare, etc.), ideally in a form that is commented sufficiently that an informed practitioner could make use of and evaluate it. "Available on request" is not accepted, nor is referring to an unevaluated preprint acceptable, either. I know that this is going to place a considerable burden on your co-authors, but my understanding is that it is non-negotiable. Please see PLOS Data Availability Policy (links above) for more information.

All code used for data acquisition and analysis is available from a public GitHub repository at: https://github.com/Neale-Lab/OctadRecombinationMapping This information is referenced in the text (Line 1088-1091).
All spreadsheet files and tables that underpin the figures are included as a supplement and are named with a reference to their use in each of the main figures. Data plotted in main and supplementary figures has also been included in a single excel file for ease of access.

4. WT and msh2Δ strain data. I could not figure out from the paper whether these data were from new tetrads or incorporated previous data from Martini et al and Marsolier-Kergoat et al. Could you either please clarify in the text, or forgive my oversight?

All presented data are newly-derived datasets generated in the SK1-S288c hybrid. We have clarified this in the Methods section (Line 853). Wild-type and *msh2Δ* data from Martini show broadly similar features in terms of CO and NCO counts, but were not included due to the underlying microarray files being incompatible with the various steps of the event calling pipeline used here which accepts FASTQ files as input. The four *msh2Δ* meiotic datasets from Marsolier-Kergoat are also distinct from the nine *msh2Δ* meioses collected and analysed here, and were not aggregated within the analyses that we have presented.

5. Despite reviewers' response to the question, in my opinion underlying data is not available in the current version. Again, PLOS data availability policy is that all underlying data need to be publicly accessible and available. I interpret this as meaning that the numerical data used to generate each an every graph in the paper (I know, there are *many) need to be reported, either in supplementary tables included with the publication (i.e. multisheet excel file) or in similar tables deposited at an openly-accessible public repository, etc. etc. Again, I know that this will create a lot of work, but it is non-negotiable.

As indicated above, we have made all raw data, scripts, and data tables available.

And then the array of minor comments (please forgive me if they are duplications with the reviewers' comments). Numbers refer to line numbers.

a. 44-45. One of the potential differences is that Mec1 is central to Hop1 phosphorylation, while Rad24 is not (I think...). Could this explain differences seen between the two?

Perhaps yes. We have included this possibility within the discussion (Line 526-528).

b. 81-83. I think that it would appropriate to cite Nancy Hollingsworth's latest papers in support of this--they address the issue much more directly.

We have amended the text and included citations to the Hollingsworth lab (Line 83-87).

c. 130-134. Since Stephen Gray's experiments were done with SK1/SK1 strains, at the minimum, a qualifier to the effect that, if Mec1 protein degradation is similar in hybrid strains...should be added. Could this also explain differences between mec1-mn and rad24∆?

We have amended the text (Line 136-139), and include a discussion of the potential reason for the difference between mec1-mn and rad24∆ (Line 717-719).

d. 208-210. Please explain why a greater increase in NCOs than in COs results from CO homeostasis. Couldn't it also result from increased heteroduplex tract lengths?

This is a very useful point that we hadn't considered. We have added this possibility to the text (Line 328-332).

e. 211-212. Meaning of this sentence is not clear. Also, in Figure S3, it would be useful to include a line for wild-type in all panels. It looks to me that mec1-mn ndt80-AR is still skewed towards the telomere, relative to wild-type.

We have added the WT line as suggested, and simplified the text to improve clarity (Line 246-249).

f. 219. Interhomolog recombination frequency (not rate).

Corrected.

g. 281-283. I do not follow the logic of this statement--given the negligible variance in terms of number of recombination events per cell for wt or sml1∆, how can CO homeostasis be assessed? And, by reporting CO/NCO ratios, rather than CO/total, aren't you effectively doubling the variance, since COs and NCOs are dependent variables?

We agree with this statement and have clarified the text in this section as suggested (Line 319-323), and also removed the trend lines for WT and *sml*∆ in Fig 3D (previously Fig 2G). Regarding the second point: This may be true, but it seems that assessing how the CO:NCO ratio changes with event count is the relevant statistic to present when assessing the potential effect of homeostasis. E.g. We believe this figure asks the question: "What is the relative proportion of COs vs NCOs when recombination frequency is low, mid, high?".

h. 329-334. Criteria that distinguish a "meiotic" 8:0 from a "mitotic" 8:0 are not terribly clear--I assume that the former must have associated heteroduplex.

Correct. This was described in the methods, but we have additionally clarified this within the text (Line 390).

The fact that there are many more such double events in ndt80-AR than in msh2Δ would suggest that MMR-induced secondary events are making a major contribution. What happens in ndt80-AR msh2Δ?

We have added the value for msh2Δ ndt80AR to Fig 4F, which is intermediate between msh2Δ and ndt80AR suggesting a contribution of both MMR-induced secondary events, and prophase arrest upon the frequency of such events. We have made note of this on line 391-394

For the multiple events reported in Figure 3, can you make an assessment of the observed/expected ratio for each type (i.e. dCO, dNCO, etc)? That, rather than significance of difference from wild-type, seems to me the important metric to consider.

Our results indicate that the difference in event number does not correlate with altered proportions of mixed type events. Therefore, the change in proportions is independent of the number of events. We have updated our manuscript to note this (line 396-399).

i. 354-360. Need to consider mismatch rejection followed by reinvasion as another explanation. The greater effect for NCOs than COs may be more consistent with this than with correction.

We have included this possibility (Line 425-427).

j. 388. This may be confusing to the reader. I think that you mean that the number of octads needed was too great to impose on poor Margaret! Later in the paragraph, Jasvinder Ahuja should be included on the personal communication.

We have clarified this sentence and included the citation to Jasvinder's published work (Lines 460-464 and 466).

k. 405-411. I don't think that the numbers are additive here, so I would urge caution.

These numbers were not added - both classes show the change.

l. 413-415. Could these be 2x DSBs?

This is possible, but not the simplest explanation. We have added this possibility to the text (Line 491-493).

m. 494-501. I am a big fan of bioRxiv, but I am not comfortable with the use of an unreviewed bioRxiv manuscript as justification of the validity of the modeling approach. Please also see reviewer #1 comment 2.

In response to this comment and others, we have decided to remove this section of the manuscript and all mentions of the unreviewed bioRxiv manuscript, which is currently undergoing revision.

n. 560-568, Figure 6H. Please include spore viability of ndt80-AR SK1/SK1 strains in this figure.

We have added spore viabilities as Fig 7J

o. 632-635. In the absence of external validation of the mixed modeling approach, this argument is circular and should be dropped.

In response to this comment and others, we have removed this text.

p. Figure 2. Please consider splitting into 2 figures, which might help with the issues with the legend in panel E.

As suggested, we have split Fig 2 E-G into a separate figure (now Fig 3). As a result all subsequent figure numbers are incremented by one.

Figure 5, legend--should be "illustrated in FigS7 and S8", I believe.

We apologise for this oversight and have amended the text and Figure legend (Line 1170).

q. Tables: in my printout, the shading in the tables makes some cells difficult to read. Please remove the shading unless it adds meaning—as in Table S5 (which lacks an explanation for colors).

We have revised the tables as suggested. We have amended the legend for Table S5 (now Table S4).

Reviewer's Responses to Questions

Comments to the Authors:
Please note here if the review is uploaded as an attachment.

Reviewer #1: Several canonical DNA damage response (DDR) factors also impact the control of meiotic recombination. This manuscript analyzes the effects of two conserved DDR factors

Mec1(ATR) and Rad24(RAD17) on recombination in budding yeast by sequencing the meiotic products either as tetrads or as octads. The authors show that inactivation of the two factors (through depletion of Mec1 and deletion of Rad24) causes a variety of changes in the patterns of meiotic recombination. Some of the changes are shared between the two mutants, including loss of spatial regulation, consistent with Rad24 being one of the activators of Mec1. In addition, loss of Rad24 shares fundamental features with mutants lacking the crossover factor Zip3, in line with observations from the Shinohara lab showing that Rad24 has a Mec1-independent role in loading Zip3 onto chromosomes. The authors also find evidence for a role of Mec1 in suppressing meiotic DNA double strand breaks at allelic sites in trans. The authors present several interesting approaches for inferring heteroduplexes from tetrad data and present a mixed model approach for extracting the proportion of class I and class II crossovers. The paper is well written and the analysis provides an intriguing view of the roles of two conserved DDR factors in regulating meiotic recombination that is expected to be of substantial interest two the field.

We thank the reviewer for their supportive and useful comments.

1. One clear caveat of the approach taken here is the need for complete tetrads as well as hybrid genomes. This appears to be a minor issue for the analysis of mec1-mn mutants, but the authors report major problems in recovering fully viable tetrads from rad24 mutants from the hybrid strains, possibly because Rad24 helps meiotic cells deal with heteroduplexes. To some extent this is the nature of the beast, and the authors make a good point that biases produced by the low spore viability will presumably make the observable phenotypes less severe. I think this argument is absolutely valid when looking at the patterns of recombination, such as event lengths or multi-chromatid events. However, I think the authors need to take greater care in particular when talking about the overall levels of CO events of these mutants (e.g. line 668). The rad24 mutation in particular shows opposite effects depending on whether wild-type (sml1) cells are analyzed or whether the analysis involved rescue by msh2 or extended prophase arrest. This opposite outcome complicates interpretation when considering the wild-type situation.

In response to these comments and those of the Associate Editor (see comment/response #1 above), we have: 1) Provided a table of the raw spore viability patterns for each strain (Table S1). 2) Added an emphasised section in the results section to make it clear that there are likely to be observational biases introduced due to the selection for rare spore-spore viable meioses (Line 222-225). 3) Toned down and added additional caveats when comparing frequency differences (e.g. Line 326-328). We hope these changes will satisfy the reviewer and prove useful for the general reader.

2. I think the mixed model for dissecting the contributions of class-I and class-II crossovers has the potential to be quite powerful. What I am missing is some form of validation. It would be important to test this model on mutants that have been better characterized for their effects on the two crossover classes, instead of simply assuming that the proposed ratios are correct. I am

particularly struck by the final two paragraphs of the results section, in which the authors find that rad24 mutants are not dependent on Mus81 even though their model predicted an excess of class II crossovers. There are multiple possibilities that could explain this observation. It could indicate a role for additional resolvases, although their mus81 mlh3 mutant would argue against this. It could also mean that crossovers in rad24 mutants are neither class I nor class II. But I think another possibility is that the model is not sufficient. For example, the model appears to treat the genome uniformly but the authors also show surprising regional effects on crossover formation, in particular in the msh2 and ndt80AR backgrounds. If these regional effects cause regional differences in the positioning of crossovers, then a mixed model assuming a uniformity across the genome is likely insufficient. I think without further validation, the discussion of these results needs to be toned down substantially.

In light of these comments and others, we have decided to remove discussion of mixed models and all references to the unpublished BioRxiv manuscript. As pointed out by the reviewers, these results are unvalidated. We will be revising this manuscript at a later date.
Instead, we have provided a comparison between each genetic background and a random simulation, which simply involves generating random inter-crossover distances from a gamma distribution with an alpha value of 1 (explained in revised methods in more detail, revised Fig7A-H). Though we see changes in CO distribution that are independent of the number of crossovers, this does not provide direct insight into the proportions of interfering and non-interfering crossovers, so we have dropped this point from the text. It only suggests that CO interference has overall stronger or weaker influence in specific backgrounds, which could either be due to differences in proportions of interfering and non-interfering crossovers or due to interference becoming stronger or weaker. The results and discussion have been updated to account for both explanations. We thank the reviewers for these comments.

3. Along the lines of the previous point, the authors argue that Figure S10E (rad24 ndt80AR) shows a single, completely random component, but the curve appears to have too much structure for this. Why is there a bump around 10kb? It seems more likely that a more complex model, maybe including a third population, may be needed.

In addition to the above changes we have clarified the text when describing the *rad24* dataset to state that it shifts towards that of a random distribution (Line 562-564). Though the *rad24* ICD distribution is visibly different from the random distribution, the two distributions are not significantly statistically different ($P = 0.399$), therefore we cannot confidently conclude that the *rad24* ICD distribution is a non-random distribution. Whilst we cannot say with absolute certainty, we favour the view that the bump observed is likely to be due to stochastic variation within the dataset.

4. Some of the effects reported here are remarkably similar to recent analyses of pch2 mutants by the Nishant group (Charkraborty et al G3 2017). These parallels should be discussed.

We have added a section in the discussion with comparisons to the *pch2* mutation (Line 721-726).

5. Please provide all the code used in these analyses. It is not possible to recapitulate the presented findings if the code is not made available. Most journals nowadays require that the scripts be deposited on github or a related repository, and the authors should adhere to this standard instead of repeatedly referring to "in-house" scripts (e.g. VariantCaller, event calling [line 879], RecombineSim, GMM,…).

As indicated in responses to the Editor above, all scripts are now available on Github at https://github.com/Neale-Lab/OctadRecombinationMapping (Line 1088-1091).

Additional comments, typos:
1. Could some of the milder phenotypes of mec1-mn compared to rad24 be due to incomplete depletion (in particular in the early stages of recombination)? This could be mentioned, unless the authors have evidence that argues otherwise.

We have added this possibility to the results discussion (Line 136-139).

2. Line 248-255: This paragraph is very hard to read because the topic switches mid-paragraph from rad24 to mec1. I suggest separating these thoughts into two paragraphs.

For brevity we have decided to delete this paragraph since, in retrospect, we think it is challenging to infer what effects there may be on the frequency of intersister recombination from this assay.

3. Figure 2A: The lines indicating fold differences are not properly aligned with the bars. Also, there is an unnecessary tick on the "2.1x" line in panel 2D.

To avoid clutter, these numbers have been removed from the figure.

4. Figure 2E, G,H: I suggest including a legend on all panels. It is not easy to look back and forth between the panels for the legend. Also, the arrow indicating increasing crossovers makes me feel like the order of the strains should be reversed so the arrow is not pointing down. Finally, please improve the visibility of the light colors. In particular the yellow in 2D, H is very hard to see on the white background.

The suggested changes to Figure 2 have been implemented (now figure 3A, C, D, E).

5. Figure 2F: Please include all chromosomes in this graph and in the analysis. A lack of non-exchange chromosomes in the dataset is still data and should be considered.

All chromosomes have been added to the graph.

6. Line 416: events
7. Lines 465 and 489: unformatted references

We have made the suggested corrections

8. Lines 511-513: It is not intuitive to think about a 0.7-fold decrease. I suggest putting these numbers as %

9. Lines 626-632 are very hard to read. Also, is there are contradiction between the effect of rad24 and rad24ndt80 on skewing in these two sentences?

We thank the reviewers for these suggestions. Based on other comments from reviewers, we have decided to remove this section on mixture modeling given its reliance on unpublished methodology.

10. Some of the colors used in the bar graphs are very pale and are barely visible above background in the legends. I suggest adding a black frame around the boxes in the legend like it is done for the bars.

As suggested, we have added a black frame to the boxes in the legend.

11. Figure S5E, F, G: To orient the reader, please highlight the positions of the relevant segments in the images.

We have highlighted these segments as suggested.

Reviewer #2: The manuscript entitled "Separable role of the DNA damage response kinase Mec1(ATR) and its activator Rad24(RAD17) within the regulation of meiotic recombination" is a nice detailed examination of two important components of the DNA damage response during meiosis. The study using genome-wide analysis of SNPs to dissect out the changes in recombination signatures/types to investigate at what level these proteins affect recombination regulation. Although many of the findings were not novel and rather confirmatory, two findings are worthwhile. Their analysis to separate out Class I and Class II crossovers is compelling and shows how CO interference is affected differently in the two mutants indicating additional separable roles for the proteins. The analysis of the pseudo octad is unique and clever allowing the authors to really separate out whether a particular recombination category occurs from a single or multiple DSBs which allows them to tease out from their analysis whether the regulation occurs at the CO or DSB level. The logic for the general flow of the paper is clearly laid out. However there are some points where clarification is needed.

Specific comments:

Lines 263-268: 6 E0's in rad24sml, but there's low CO (40% of wt), so it seem obvious that E0s occur.

While this is a valid observation, our results suggest that there are more E0s in rad24 sml than expected from chance alone, given the number of COs observed. Therefore the low number of COs does not fully explain the number of E0s observed. See also our answer below.

50% of rad24 ndt80 msh2 but 10% of mec1 ndt80 msh2 have E0s and that this could be explained by less CO assurance in rad24. But rad24 msh2 has fewer COs, Can you separate out the data and show that it's not only because of fewer COs in that mutant. And a frequency of E0 for wt should be given.

We have added a new figure (figure 3) to show differences in the number of E0s between each genotype.

277 paragraph on CO homeostasis. This might not be worthwhile including since you are measuring CO homeostasis where there is a big selection for viability, those with more CO's (given the same number of DSBs) are more likely to live.

As well as clarifying the text in this section, we have added a comment that mentions the potential skew towards higher CO fraction due to selection for viability when event count decreases (line 222-225).

~333 There needs to be some clarification about what the loss of trans-inhibition of DSBs is doing. The loss of trans-inhibition of DSBs can explain loss of CO assurance in rad24, but there's a similar loss of trans inhibition in mec1, but a greater loss of CO assurance.

Our revised analysis finds no significant loss of CO assurance in mec1 but suggests that there is a loss in rad24 (figure 3B).

~527 if the loss of trans-inhibition in mec1 provides all the DSBs for the extra events why isn't it providing additional DSBs for extra events for rad24. What do you think is happening in the rad24 case?

Based on other comments from reviewers, we have decided to remove this section on mixture modelling entirely given its reliance on unpublished methodology.

Figure 5:
Although the categories are better explained in a Llorente review, to make the categories more understandable for the reader of this manuscript it would be good to incorporate the below changes since it is a crucial part in the argument:

Fig5BC:
Each category should have a corresponding drawing

These drawings are now provided in the supplementary material, figures S5 and S6. We have added references to these figures in the figure caption.

FigS5:
Show recombination intermediates to explain the dNCO, dCO and CO+NCO with 8:0 and 7:1 segments

We have added these in the new figure S6.

I would explicitly in the text say that you excluded premeiotic 8:0. You can infer that by the fact you only include the 8:0 regions with associated hDNA tracts but that is too hard for the typical reader.

We have now noted this in the methods section (line 995-996)

Fig5DEF and FigS8 :
When talking about the hDNA types, it would help to have drawings of the corresponding recombination intermediates.

We have added these in the new figures S11 and S12. These have already been described in Marsolier-Kergoat et al. 2018. In particular, the drawings for Fig 5DEF are exactly the same as Fig 3 from that study. The other new drawings are adaptations of other models and concepts proposed in the same paper.

It would also be interesting to see the different types of hDNA signatures associated with the corresponding resolution pathway.

We have linked the new supplementary figures described above to each corresponding outcome in the figure legends, using the same titles in each case and referring to the corresponding figures in the figure legends.

Reviewer #3: The authors categorized the various pathways of meiotic recombination in detail by combining the nucleotide sequence polymorphism between S288C strain and SK1 strain of budding yeast with msh2 mutation and ndt80AR. As a result, they clarified the common functions and different functions in meiotic recombination control of Mec1 kinase and Rad24 clamp loader, which are necessary factors for the DNA damage response (DDR). Common functions like trans-DSB interference, hyper-DSB resection etc. were clarified. On the other hand, there are differences in function in CO interference. Mec1 mainly functions through trans DSB suppression, whereas Rad24 functions in class I CO formation indirectly via Zip3 loading.

This paper looked at the molecular mechanisms behind differences in Mec1 and Rad24 function by detailed comparison, using a new method. Although the novelty of the final model is not high,

I think that the paper contains important findings to understand molecular functions of meiotic CO control. However, the following comments need to be answered.

Major comments

1. P5 L153 – 155. The authors mentioned as "… the loss of the DNA damage response checkpoint causes an early exit from meiotic prophase." with a citation showing NDT80 expressed earlier in DDR mutants. However, according to a previous report from the Shinohara group (Genetics, 2003), DDR mutants show a delay in meiotic divisions in response to a delay in the formation of meiotic recombinants. In the hybrid rad24 strains used here, is discoordination between meiotic recombination and meiosis progression observed? Authors should also consider possible effects of reduced expression of NDT80 in the ndt80AR strains.

The discoordination between *NDT80* expression and entry into the meiotic divisions in DDR mutants is certainly intriguing and suggests that Ndt80 alone is insufficient to drive chromosome segregation. In terms of our observations, we can confidently state that delaying expression of *NDT80* rescues the acute spore viability defects present in the *rad24*Δ strain, and this is independent of diploids being hybrids or non-hybrids (Fig 1C, S2B, Table S1 and Gray et al Open Biology 2013). We have revised this portion of the text (Lines 164-166) to refer to encompass the possibility that the ndt80AR system may both extend prophase and/or suppress early expression of Ndt80 that arises in DDR mutants. Because recombination maps are assembled from individual meioses, we are unable to determine the precise time or extent of Ndt80 expression that occurred in the individual meioses that we characterise.

2. P6 L205 – 212. It is suggested that CO homeostasis in mec1-mn is maintained, due to the slight difference between the increased rate of COs and the increased rate of NCOs when recombination is increased in the msh2 or ndt80AR background. However, this is indirect evidence and not enough to lead to a strong conclusion. This suggestion should be removed or not be made so strongly.

We agree and have removed this statement about crossover homeostasis.

3. P13 L444-452. I cannot accept the authors' logic in this section. Even though a sufficient amount of recombination between homologous chromosomes is observed, this is not evidence that further inter-sister recombination cannot be suppressed. I think that it is an overstatement to say that the mec1-mn mutant has no deficiency in inter-homolog bias by this logic, because inter sister recombination is not directly studied. This part should be eliminated.

As suggested, we have removed this section

4. Relating to the above question, DDR mutant strains are known to cause increased ectopic recombination. Did you detect any (inter-homolog) ectopic recombination using this analysis method?

This is an excellent idea, and something we have been very eager to follow up. So far we have approached this by attempting to identify chimeric read pairs that originate from two chromosomes—indicative of an ectopic recombination event. However, the number of detected events is low and doesn't seem to correlate with genotype. We also are aware that the inherent bias towards viable spores and the relatively short reads that were used to generate these data limits our ability to detect ectopic recombination events. Thus at this stage we feel these analyses are too preliminary to report.

5. Overall, the influence of the msh2 mutation, in particular the effect on recombination tract and the effect on stronger CO interference (Fig. 9SD) cannot be overlooked. Since this is an important point affecting the interpretation of the issue, authors should explain the effect of msh2 mutation on meiotic recombination more carefully.

Following this useful point, we have updated the interpretations and discussion of datasets collected in *msh2*Δ background, for example, Line 422-430, throughout pages 13-14, Lines 544-555, and 765-791.

Minor comments
1. P8, L285. Fig 1A shows strategy of the analysis. Is there any appropriate reference?

This statement (And the erroneous reference to Fig 1A) has been deleted in the revision.

Have all data underlying the figures and results presented in the manuscript been provided?
Large-scale datasets should be made available via a public repository as described in the *PLOS Genetics* data availability policy, and numerical data that underlies graphs or summary statistics should be provided in spreadsheet form as supporting information.

Reviewer #1: Yes

Reviewer #2: Yes

Reviewer #3: Yes
